# Fluorinated Glycan Frameshifts: Automated Synthesis Expedites the Study of Glycan‐Protein Interactions by ^19^F‐BioNMR

**DOI:** 10.1002/anie.8014647

**Published:** 2026-02-08

**Authors:** James Suri, Christina Jordan, Charlotte S. Teschers, Kristina Schlangen, Simon H. Rüdisser, Alvar D. Gossert, Ryan Gilmour

**Affiliations:** ^1^ Institute For Organic Chemistry University of Münster Münster Germany; ^2^ Department of Biology ETH Zürich Zürich Switzerland; ^3^ Department of Biomolecular Systems Max Planck Institute For Colloids and Interfaces Potsdam Germany

**Keywords:** automated glycan assembly, fluorine, molecular recognition, NMR spectroscopy, stereoselectivity

## Abstract

Given the prominence of ^19^F‐bioNMR in structural research, fluorinated glycan frameshifts hold enormous potential in studying carbohydrate‐protein interactions. To contribute to this field, the synthesis of selectively C‐2 fluorinated glycans related to the O3b antigen of *Klebsiella pneumoniae* is disclosed, and their interactions with the lectin Concanavalin A (ConA) are interrogated spectroscopically. Automated glycan assembly (AGA) was employed to expedite construction in which the C(sp^3^)‐F bond was leveraged to control stereoselectivity of α‐mannosylation. Subsequent ^19^F‐BioNMR analysis of binding to ConA allowed determination of the respective IC_50_ and *K*_D_ values; this revealed a conspicuous frameshift‐dependency in which one pattern dominated. Collectively, this study advocates for the strategic utilisation of the C(sp^3^)‐F bond in the design, construction, and analysis of probes to interrogate ubiquitous mannose‐binding lectins with therapeutic relevance.

## Graphical abstract

Given the prominence of ^19^F‐bioNMR in structural research, fluorinated glycans hold enormous potential in glycomimetic frameshift design. However, challenges associated with the stereocontrolled synthesis of these disruptive actors continue to frustrate advances. To address this, the automated synthesis of selectively C‐2 fluorinated glycans related to the O3b antigen of *Klebsiella pneumoniae* is disclosed, and their interactions with the lectin Concanavalin A (ConA) are interrogated spectroscopically.

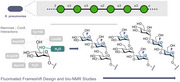

## 1 Introduction

Glycan structural diversity manifests itself in a “*continuum of function*” that is unrivalled in Nature [1, 2, 3, 4, 5, 6, 7]. Whilst many biopolymers are encoded at the genome level by processes that can be emulated in the laboratory [8, 9, 10], glycan assembly is not constrained by templated synthesis. Removing this limitation provides remarkable latitude in the generation of linear and branched structures that permeate all aspects of biological recognition. The evolutionary success of glycans is reflected by Laine´s venerable calculation of the theoretical number of isomers of a reducing hexasaccharide; this revealed a trillion permutations (1.05 x 10^12^)! [11] Despite the potential of glycans in the development of novel therapeutics, the juxtaposition between biological ubiquity and clinical translation remains conspicuous [12, 13, 14, 15, 16, 17, 18, 19, 20].

Impediments include the absence of an iterative biological glycosylation blueprint, such that reconciling this disparity is contingent on effective strategies to expedite the stereocontrolled synthesis of well‐defined glycans [21, 22]. The inherent hydrolytic vulnerability of glycosidic linkages in vivo and in vitro must be considered in molecular design [23, 24], and effective structural biology approaches are vital to facilitate the study of strategic protein‐carbohydrate interactions [25, 26]. The latter issue is particularly urgent given the notoriously weak nature of these orchestrating events, and the consequences this has for biomedical applications [27, 28, 29]. Fluorinated glycans are disruptive in tackling this multifaceted challenge, where remarkable changes in structure, physicochemistry and reactivity originate from OH to F exchange [30, 31, 32, 33, 34, 35, 36, 37, 38, 39, 40, 41]. In particular, locating the C(sp^3^)‐F substituent adjacent to the anomeric centre ensures a powerful steering group to regulate the stereochemical outcome of chemical glycosylation [42, 43, 44, 45, 46, 47, 48, 49, 50, 51], such that the newly formed C(sp^3^)‐O bond is forged according to a modified Anh‐Eisenstein induction model (approach of the nucleophile 90° to the σ*_C‐F_ orbital, which itself is coplanar with the π*_C = O(+)_ orbital) [52, 53, 54]. Enhanced electronegativity, accompanied by fluorination, mitigates the hydrolytic liability of the product glycan [23, 24, 55] and simultaneously provides a sensitive NMR‐active probe with minimal steric disruption [56, 57, 58, 59, 60, 61, 62, 63, 64].

**FIGURE 1:**
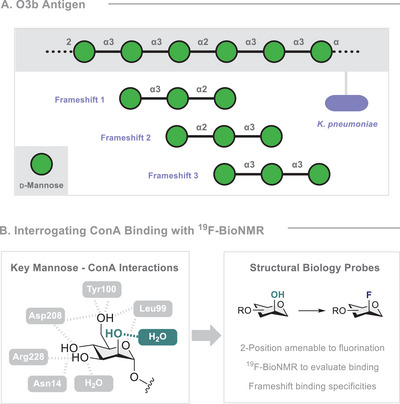
(A) O3b antigen of *K. pneumoniae* and possible frameshifts of the repeating unit. (B) Known interactions between mannose and ConA and why they inform the decision to generate 2‐deoxy‐2‐fluoro analogues for ^19^F‐BioNMR analyses.

**FIGURE 2:**
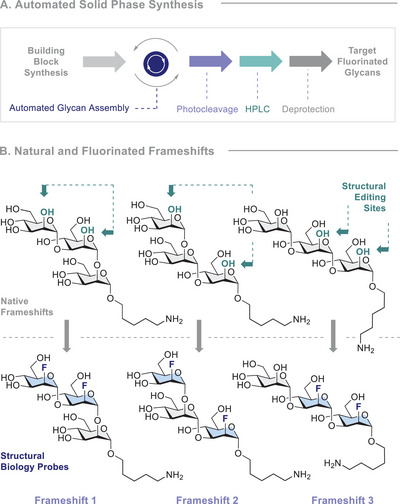
(A) General overview of AGA in this work. (B) The structures of the frameshifts that were synthesised in this study.

Importantly, strategic fluorination enables key hydrogen‐bonding interactions in glycan‐protein complexes to be systematically interrogated: [65] this has recently been validated in complex scenarios such as the GM1–cholera toxin [66] and Le^a^–LecB [67] complexes. The potential of fluorinated glycan motifs in biomedicine is further reflected by their success as vaccine leads [68], where site‐selective fluorination provides a handle to confer hydrolytic stability and/or enhance immunogenicity [69, 70, 71, 72]. Cognisant of the physicochemical variances that distinguish F‐glycans from their native counterparts, and the varying biological functions elicited by antigen frameshifts [65, 73, 74, 75], an opportunity exists to generate an integrated approach to studying F‐glycan‐protein interactions expedited by automated glycan assembly (AGA) [76, 77, 78, 79, 80, 81, 82, 83, 84, 85]. With Laine´s hexamer calculation in mind [11], the hexameric section of the O3b antigen of *Klebsiella pneumoniae* emerged as a particularly pressing target for investigation (Figure 1A). Increasing resistance to antibiotic treatments [86] and the absence of an approved vaccine [87] renders the O3b antigen clinically relevant. Moreover, the repeating α‐linked trimannoside [88, 89] can be expressed as three different frameshifts, rendering it ideally suited for this investigation. Given the ubiquity of mannose‐binding lectins (e.g., DC‐SIGN and langerin) [90], the key interactions of mannosides and Concanavalin A (ConA), were examined (Figure 1B, left). ConA, a plant lectin derived from the jack bean (*Canavalia ensiformis*), has previously been used to identify carbohydrate structures in various cell types, including malignant cells [91]. It recognises α‐linked glycans, particularly glucose and mannose; it exhibits high specificity for trimannosides in particular; this motif is common to high‐mannose‐type *N*‐linked glycoproteins. ConA forms a tetramer at pH 7, with each monomer possessing one carbohydrate‐binding site [92]. According to Goldstein and colleagues, the equatorial orientations of the hydroxyl groups at C3 and C4, and the hydroxymethyl group at C5, are essential for binding [93, 94]. Since the C2 position of the monosaccharide occupying the main binding site is bound to a water molecule and does not engage in hydrogen bonding with the protein [95, 96, 97], it is ideally suited to molecular editing with fluorine. Furthermore, a hydrophobic region adjacent to the binding site led us to postulate that fluorination might lead to notable improvements in the IC_50_ values. To test this hypothesis, it was envisaged that fluorine could be leveraged throughout the AGA and structural ^19^F‐BioNMR studies to allow native and fluorinated frameshifts of the O3b antigen of *K. pneumoniae* to be compared (Figures 2A and 2B).

## 2 Results and Discussion

**SCHEME 1:**
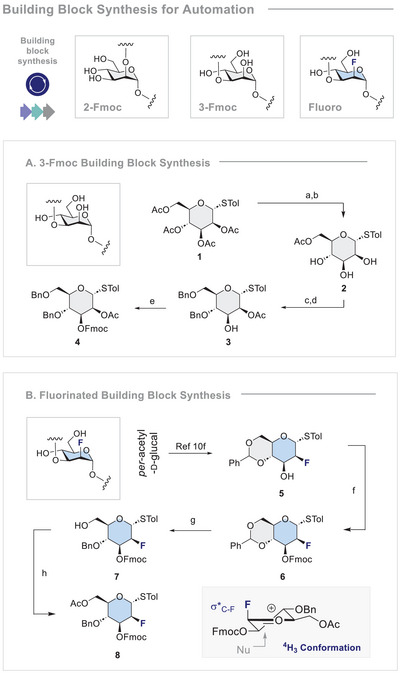
Building Block Synthesis for Automation. **A**. Synthesis of BB **4**. Conditions: a) NaOMe, MeOH, rt, o/n; b) AcCl, 2,4,6‐collidine, ‐40°C, 30 min, 61% (2 steps); c) CH_3_C(OMe)_3_, *p*‐TsOH, DMF, rt, 3 h, then NaH, BnBr, 0°C, 1.5 h, then H_2_O, NaH, BnBr, 0°C, o/n; d) HCl, EtOAc, rt, o/n, 74% (2 steps); e) FmocCl, pyridine, DCM, rt, o/n, 95%. **B**. Synthesis of BB **8**. **B** Conditions: f) FmocCl, pyridine, DCM, rt, o/n, 93%; g) BH_3_•THF, TMSOTf, DCM, 0°C, 72 h, 63%; h) Ac_2_O, pyridine, DCM, rt, o/n, quantitative. Insert: The ^4^H_3_ conformation of the corresponding oxocarbenium ion leading to the α‐product is shown.

To prepare the target trisaccharide frameshifts outlined in Figure 2B, it was envisaged that the synthesis could be simplified to three building blocks (BBs) (Scheme 1, top).

For this particular investigation, in which translation to an automated platform would be validated, thiodonors were selected on account of their stability and widespread application (please see the Supporting Information for the synthesis of the 2‐Fmoc BB). The synthesis of a suitably protected non‐fluorinated BB for the 3‐linkage began with thiodonor **1** (Scheme 1A). Zemplén deprotection and selective acetylation of the 6‐OH position proceeded smoothly to yield triol **2**. A one‐pot, sequential reaction followed by acid hydrolysis transformed **2** into the partially‐protected compound **3,** bearing a free 3‐OH [98]. Finally, an efficient Fmoc protection step completed the preparation of BB **4** (30% over 7 steps from d‐mannose).

Construction of the key fluorinated building block to forge the 3‐linkage was achieved via a multi‐step campaign starting from *per*‐acetylated d‐glucal (Scheme 1B). Benzylidene acetal **5** was synthesised according to the literature, followed by Fmoc protection to generate the fully protected scaffold **6**. Regioselective benzylidene ring opening with borane proved effective in generating the primary alcohol **7**, and this position could then be acetylated to complete the synthesis of BB **8** (10% over eight steps). In this particular case, the choice of protecting groups was deliberate to favour the ^4^H_3_ conformation during the glycosylation to generate the α‐product [42]. The α‐configuration at C1 of **8** was established by ^19^F NMR analysis: (^2^ *J*_HF_ 50.0 Hz, ^3^ *J*_Fax‐Hax_ 28.1 Hz, ^3^ *J*_Fax‐Heq_ 14.4 Hz) (please see the ESI).

**FIGURE 3:**
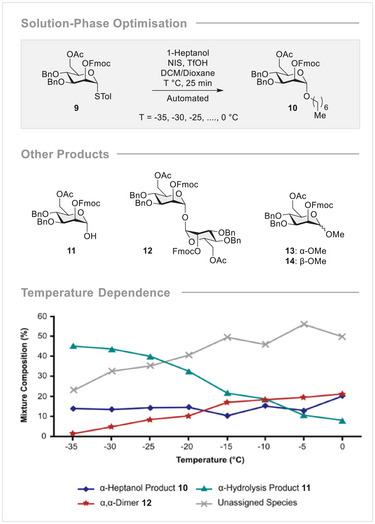
Solution‐phase glycosylation optimisation of BB **9** to 1‐heptanol. Percentages calculated are based on integrals of all HSQC anomeric cross‐peaks (85 ppm—102 ppm and 6.40 ppm – 4.40 ppm) representing 100%. The 8 eq. donor excess means that 12.5% of the mixture composition represents quantitative product formation.

Having the key building blocks in hand, attention was then turned to determining optimal solution‐phase glycosylation conditions that could then be translated to an automated paradigm. Owing to the complexity of automated synthesis, this step‐wise approach was deemed prudent to delineate possible factors affecting yield and stereoselectivity. Initially, a range of temperatures was surveyed (see the Supporting Information) [99], which then informed the conditions for test glycosylations under automated conditions with a commercial Glyconeer 2.1^®^ instrument. For this purpose, 1‐heptanol was selected to mimic the primary alcohol of the aminopentanol linker used in solid‐phase synthesis. The results for BB **9** (the 2‐Fmoc BB) are summarised in Figure 3.

In a departure from solution‐phase behaviour, the formation of the desired product **10** was accompanied by several side products: where possible, these species were isolated and characterised as being the hydrolysis product **11**, the 1,1‐dimer **12**, and OMe adducts **13** and **14**. Their identification was based on comparing crude ^1^H,^13^C‐HSQC spectra to standards that were prepared independently. It is important to note that compounds **13** and **14** resulted from contamination of the instrument during washing steps, and their formation was eliminated by simply altering the standard washing cycles. Dimer **12** is an indicator of hydrolysis during glycosylation, whereas lactol **11** is formed during the aqueous reaction workup from excess activated donor. As such, lactol **11** serves as a measure for the efficiency of donor activation and donor stability under the reaction conditions. Therefore, the reaction was optimised for a maximum content of **10** (8 equivalents of **9** means 12.5% maximum content of **10** in the mixture) and **11** in the crude reaction mixture. The results of this solution‐phase study indicated that α‐selectivity dominates and is insensitive to temperature. Furthermore, the yield of **10** proved to be quantitative at all temperatures, thereby building confidence for AGA studies. As can be seen from Figure 3, donor stability was highest at the lowest temperatures, so that the temperature range ‐35 to ‐25°C was chosen as a starting point for solid phase synthesis.

Efficiently transferring the reaction conditions to the solid phase can be challenging [100], and this case was no exception, with conditions that had been effective for the solution phase synthesis (i.e., using 8 eq. donor) giving low yields on resin (Table 1, entry 1). Using a double glycosylation cycle [83] increased the yield, albeit not to a satisfactory extent (entry 2). At a higher glycosylation temperature (−20 to 0°C), the reaction proceeded more efficiently (entry 4). Finally, upon increasing the number of equivalents and raising the temperature, quantitative conversion could be attained (entry 6). The glycosylation of BB **4** was trialled with the same conditions as used for BB **9**, and this led to effective conversion. An analogous solution phase study was carried out for fluorinated BB **8** with heptanol and revealed that **8** was fully activated at ‐30°C, and that the activated donor **8** was stable at temperatures up to 10°C (for additional details see the Supporting Information). At higher temperatures, TfOH‐mediated cleavage of the 4‐OBn group was observed, so the ‐10 to 10°C range was chosen for glycosylation of BB **8**.

**TABLE 1:** Optimisation of the glycosylation of BB **9** to the resin‐bound linker in the Glyconeer 2.1^®^.

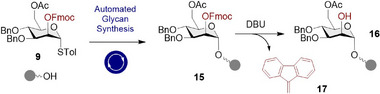				
Entry	T_1_ ^a^ (°C)	T_2_ ^b^ (°C)	Eq.	Yield^c^ (%)
1	−35	−25	8	57
2	−35	−25	2 × 5	70
3	−20	0	6	52
4	−20	0	8	74
5	−20	0	2 × 5	91
**6**	−**20**	**0**	**2 × 8**	**Quant**.

^a^ Incubation time at T_1_ was 300 s.

^b^ Incubation time at T_2_ was 1200 s.

^c^ Yield calculated using UV quantification of Fmoc cleavage.

### 2.1 Trisaccharide Synthesis

**FIGURE 4:**
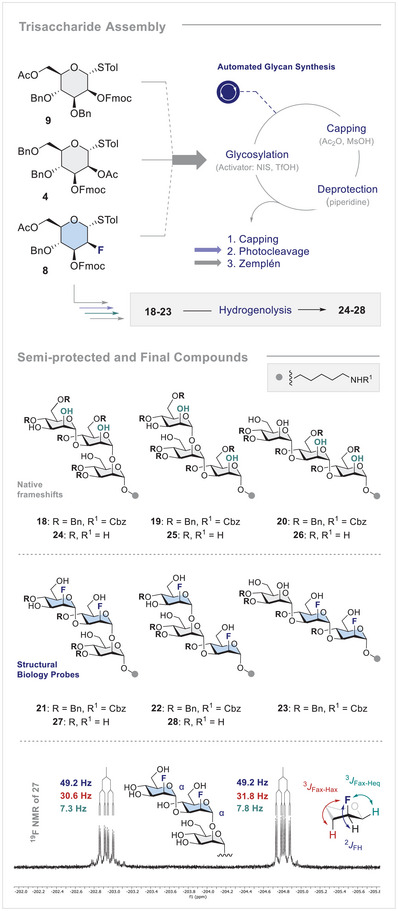
Automated synthesis of trisaccharides, including post‐automation manipulations. Synthesised semi‐protected and fully‐deprotected trisaccharides. Bottom: ^19^F NMR analysis of compound **27**.

To access the target trisaccharides required for ^19^F‐biobi‐oNMR studies, the BBs (**4**, **8** and **9**) were assembled with the aid of an automated platform (Figure 4) [51, 76, 77, 78, 79, 80, 81, 82, 83, 84, 85] with photocleavage being performed in batch. Although a flow system was examined to expedite synthesis [101], higher levels of efficiency could be obtained in batch [102]. This observation is noteworthy in light of observations that the (linear vs. branched) structure of glycans often leads to significant differences in photocleavage efficiency [103]. Following cleavage from the resin, Zemplén deprotection was performed. Size exclusion HPLC enabled the separation of the complex mixtures that arose due to the absence of purification steps during the automated synthesis cycles. At this stage, the pure semi‐protected glycans (**18‐23**) could be characterised (please see the Supporting Information for full details). Standard hydrogenolysis with Pd/C and H_2_ afforded fully deprotected trisaccharide **27** in which the anomeric configurations could be unequivocally assigned by ^19^F NMR spectroscopy (^2^ *J*_HF_ 49.2 and 49.2 Hz, ^3^ *J*_Fax‐Hax_ 30.6 and 31.8 Hz, ^3^ *J*_Fax‐Heq_ 7.3 and 7.8 Hz). It is interesting to note that for the other frameshifts, these deprotection conditions proved ineffective. This is likely due to aggregate formation, which is well documented [104]. Sonication was successfully deployed to disrupt these aggregates, enabling access to fully deprotected trisaccharide frameshifts (**24‐26, 28**) in just 6 h with yields ranging from 41–76%. The lack of a terminal 2‐F‐Man meant **23** was not fully‐deprotected or used for subsequent ^19^F‐BioNMR studies. However, its inclusion in the automated synthesis is instructive. The final yields of the fully deprotected compounds ranged from 5–10% over 10 steps, based on initial resin loading. Using the optimised conditions from automation as a reference point, trisaccharide **28** could be prepared manually in solution in 4% yield over seven steps, whereas the automated synthesis delivered compound **28** in 7% yield over ten steps. An interesting comparison arose from an attempted manual synthesis of a protected analogue of **23**. Complications, including side reactions and degradation, hampered the isolation of the final product. In contrast, the automated synthesis of semi‐protected **23** proved facile (13% over 10 steps).

### 2.2 ^19^F‐BioNMR Analysis

**FIGURE 5:**
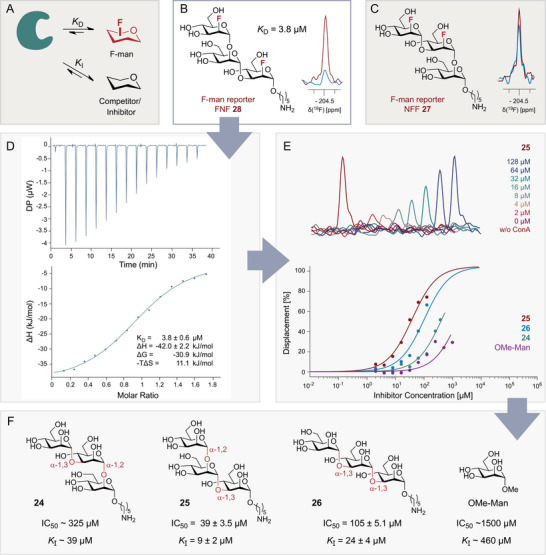
(A) Basic scheme of a competition binding assay based on observation of a fluorinated reporter molecule (red) in the presence of a non‐fluorinated competitor (black). The two F‐mannosides that were tested as potential reporters for the establishment of binding assays are shown in (B) and (C). To test their suitability, the reduction of the ^19^F NMR signal of the free mannoside (red, 13 µM) was investigated after the addition of ConA (blue, 7.5 µM). **FNF 28** showed a strong binding response (B) suitable for a binding assay, in contrast to **NFF 27**, which was very weakly bound by ConA (C). For **FNF 28**, the affinity was determined by ITC (D). Competition experiments were carried out using **FNF 28** as a reporter molecule and increasing concentrations of competitors. Example experimental data of **FNF 28** and **25** as a competitor is given. (E) IC_50_ values were fitted using the signal of free **FNF 28** as full displacement and the internal control TFE for normalisation of signal intensities. For **24** and OMe‐Man, IC_50_ values were determined from the displacement observed in the last points, as insufficient displacement was observed at the highest employed concentration. (F) IC_50_ and *K*_I_ values for the competitors tested, with their respective structures.

In order to explore how varying trisaccharide connection patterns impact ConA binding, a ^19^F‐reporter assay was established (Figure 5A). To that end, two potential ^19^F reporter molecules, which contain different fluorination motifs, were tested: **27** and **28**, termed **NFF** and **FNF** for convenience. Herein, **FNF 28** clearly showed a stronger binding response than **NFF 27** (Figures 5B,C).

For this compound, a significantly reduced signal after ConA addition was observed, due to changes in *T*_2_ relaxation in fast exchange. To determine the affinity of the reporter, NMR titration experiments were carried out, which indicated a *K*_D_ value of ≤ 7 µM, just at the border of the assay window. To obtain a more precise value, isothermal titration calorimetry (ITC) was performed, which yielded a *K*_D_ value of 3.8 ± 0.6 µM (Figure 5D, see Supporting Information for details). With **FNF 28** as a suitable ^19^F reporter, competition experiments were set up. Accounting for its relatively high affinity—which makes displacement by weak competitors challenging—the concentration of **FNF 28** was minimised to 13 µM, the lowest level compatible with the sensitivity limits of the NMR instrument.

For a comparison with monosaccharides, we also explored α‐OMe‐mannose as a competitor in addition to the synthesised trimannosides. For a quantitative analysis, IC_50_ values were fitted by using a four‐parameter equation, where the minimal and maximal displacement values were derived from the data signal of free **FNF 28** (maximum) and **FNF 28** in the presence of ConA (minimum). A cooperativity value of 1 was assumed, such that the only parameter fitted was the IC_50_ (Figure 5E, see Supporting Information for details). Notably, in these titrations, for OMe‐mannose and **24**, only incomplete displacement was achieved even at the highest employed concentrations of 1 and 0.4 mM, respectively. Therefore, here, the displacement observed for the last points was used to calculate the IC_50_ value. Using the determined *K*_D_ value of the reporter of 3.8 µM, *K*_I_ values were calculated from the IC_50_ values, as reported in Figure 5F.

In this competition assay with the non‐fluorinated mannosides a clear affinity ranking could be established with **25 **> **26 **> **24 **> OMe‐Man. From the relative affinities, SAR on the influence of OH–F substitutions and the different glycan linkages can be derived. Compound **25** and the reporter **FNF 28** itself had the highest affinity. Interestingly, compound **25**, which is the non‐fluorinated analogue of **FNF 28**, has a *K*_I_ value of 9 µM: this indicates that **25** has a weaker binding affinity than **FNF 28** itself. Since **FNF 28** was employed at a concentration of 13 µM in the assay, equipotency would have been reached at an IC_50_ value of 13 µM for **25**. This is a rare example in which fluorination of a glycan does not lead to a loss in affinity, establishing bioisosterism. To rationalise the tolerance of fluorination, it is instructive to examine the reported X‐ray structures of ConA in complex with different mannosides (PDB 1CVN, 1I3H, and 5CNA) [96].

The structures of di‐ and trimannosides (PDB 1I3H and 1CVN, respectively) have different glycan linkages, and notably, the trimannoside is branched and not linear as in this work. In the trimannoside structure 1CVN, the central mannose unit serves as the reducing end and is connected to two terminal mannoses via α‐1,3 and α‐1,6‐linkages. Our data is too limited to model the linear trimannosides with their many degrees of freedom based on the structure, and due to the different glycan linkages at least one mannose must adopt a different relative orientation. However, in all published structures, the terminal mannose binds in the same well‐defined way in the deep end of the binding pocket of ConA. In the trimannoside (PDB 1CVN), the α‐1,6‐linked mannose is located within the actual binding site of ConA, and the remaining mannose units are part of the so‐called “extended binding pocket”.

One can therefore expect that the terminal mannose of the trimannosides produced in this work binds in this canonical way. In this case, the bioisosterism of the introduced fluorine might be rationalised by the fact that the hydroxyl at the C2 position does not engage in any hydrogen bonding interactions, such that no penalty should emerge from this replacement. In light of the slightly higher affinity of the fluorinated variant, it is interesting to note that the binding pocket has a partially hydrophobic character. The residue Leu99 and two tyrosines (Tyr100, Tyr12) line one side of the lower binding pocket such that the higher hydrophobicity of the mannose seems beneficial. In contrast, for compound **NFF 27**, essentially no binding was detectable, and its IC_50_ must lie above 1 mM as no displacement of **FNF 28** at this concentration was observed (data not shown). One reason for the weakened binding can be attributed to the unfavourable α‐1,2 linkage. However, the same difference in topology is present between compounds **25** and **24**, where the impact is only a 4‐fold reduction in affinity. Therefore, it appears that the fluorine introduced at the C2 position of the central mannose is detrimental for binding. This seems rational on account of the absence of a hydrogen bond to water observed in the parent structure.

From the 3–8‐fold lower *K*_I_ values of **24** and **26** compared to **25**, and the much lower binding response of the reporter candidate **NFF 27** compared to **FNF 28**, it is evident that an α‐1,3 connection in the first linkage is beneficial for the interaction with ConA. Further, it can be concluded that the α‐1,2 topology of the second linkage is more favourable than an α‐1,3 linkage. However, the extended binding pocket seems to be quite tolerant to different glycan topologies, since α‐1,2, α‐1,3, and α‐1,6 linkages can be accommodated, albeit with different affinities.

The overall SAR that derives from the binding data can therefore be summarised in the following way: (i) an α‐1,2 linkage between the second and third mannose is more favourable than an α‐1,3 topology, while an α‐1,2 linkage between the first and second mannose reduces affinity 3‐fold, (ii) a OH to F replacement at the C2 position of the central mannose is incompatible with binding, and (iii) OH to F replacement at the C2 position of the mannose in the deep binding pocket acts as a bioisostere, or might even enhance affinity.

## 3 Conclusions

In conclusion, AGA enabled the establishment of a robust reporter assay for the characterisation of ConA binders that can be extrapolated to mannose‐binding proteins in a broader sense. The fluorinated frameshift **FNF 28** has been identified as a highly soluble reporter ligand with a relatively high affinity. Consequently, meaningful *K*_I_ values can be derived even for strong binders in the low µM range. The utility of the assay was demonstrated by exploring the structure‐activity‐relationship (SAR) of linear trimannosides, where fluorine‐editing and differential glycan linkages revealed the importance of individual OH groups, optimal glycan topology, and localised modulation of hydrophobicity. This study contributes to the design, construction, and analysis of probes to interrogate ubiquitous mannose‐binding lectins.

## Conflicts of Interest

The authors declare no conflicts of interest.

## Supporting information




**Supporting File 1**: anie71365‐sup‐0001‐SuppMat.pdf.

## Data Availability

The data that support the findings of this study are available from the corresponding author upon reasonable request.
