# Supplementary material for: Fluorinated Glycan Frameshifts: Automated Synthesis Expedites the Study of Glycan‐Protein Interactions by 19F‐BioNMR
Source: Angew Chem Int Ed Engl. 2026 Feb 8;65(12):e8014647. doi: 10.1002/anie.8014647 (PMC12990967; doi:10.1002/anie.8014647)
Supplement: Supplementary file 1 — Supporting File 1: anie71365‐sup‐0001‐SuppMat.pdf. [file ANIE-65-e8014647-s001.pdf]

Supporting Information

for

Fluorinated Glycan Frameshifts: Automated Synthesis Expedites the Study of Glycan-Protein Interactions by ^{19}F-BioNMR

James Suri,[a] Christina Jordan,[a,b] Charlotte S. Teschers,[a,c] Kristina Schlangen,[a] Simon H. Rüdisser,[b] Alvar D. Gossert,*[b] and Ryan Gilmour*[a]

[a] Dr. J. Suri, Dr. C. Jordan, Dr. C. S. Teschers, K. Schlangen, Prof. Dr. R. Gilmour
Institute for Organic Chemistry, University of Münster, Corrensstraße 36, 48149, Münster (Germany)
E-mail: ryan.gilmour@uni-muenster.de

[b] Dr. C. Jordan, Dr. S. H. Rüdisser, Dr. A. D. Gossert
Department of Biology, ETH Zürich, Hönggerbergring 64, Zürich (Switzerland)
E-mail: alvar.gossert@biol.ethz.ch

[c] Dr. C. S. Teschers
Department of Biomolecular Systems, Max Planck Institute for Colloids and Interfaces, Am Mühlenberg 1, 14476 Potsdam (Germany)

Contents

General Information 3
Automation: General Information 5
Building Block Synthesis 8
Synthesis of Standards 18
Glycosylation Optimisation 21
Automated Glycan Synthesis 27
Solution-Phase Trisaccharide Synthesis 45
BioNMR: Materials and Methods, and SAR 57
NMR Spectra 59
References 141

General Information

Chemicals were purchased as reagent grade and used without further purification unless otherwise stated. NIS was recrystallised from benzene/1,4-dioxane. Dry solvents were obtained from a custom-built chromatographic drying apparatus. For column chromatography, the stationary phase was SiO_2 (40-63 µm, VWR Chemicals) unless otherwise specified. Purification solvents were of technical grade and distilled with a rotary evaporator before use. Flash chromatography was performed with Büchi FlashPure ID silica cartridges in a Büchi Pure C-850 FlashPrep system using UV and ELSD detection. 10% Pd/C from Sigma-Aldrich was pretreated before use, as previously described.[1]

Reactions were monitored via thin layer chromatography (TLC), using aluminium foil plates pre-coated with SiO_2-60 F254 (Merck). Visualisation of compounds on the TLC plates was achieved with either a UV lamp (254 nm) or using ceric ammonium molybdate (CAM) stain. Removal of solvents was achieved using a rotary evaporator (~10 mbar, 44 °C). Further drying was achieved with high vacuum (~10^{-2} mbar) at room temperature with the option to freeze-dry with liquid nitrogen. NMR spectra were measured by the service department at the Institute of Organic Chemistry, University of Münster. Spectra were measured using Bruker Avance Neo 400, Agilent DD2 500, and Agilent DD2 600 spectrometers at room temperature, unless otherwise specified. Chemical shifts are referenced to the residual solvent peak.[2] Multiplicities are abbreviated as: singlet (s), doublet (d), triplet (t), and multiplet (m). Multiple assignments for a single reported signal are separated by a comma (,). When a specific assignment could not be made, the possible assignments are separated by a forward slash (/). Unknown compounds were assigned using ^{1}H,^{1}H-gCOSY, ^{1}H,^{13}C-gHSQC, ^{1}H,^{13}C-gHMBC, ^{1}H,^{1}H-NOESY and ^{1}H,^{1}H-TOCSY NMR spectra. For oligosaccharides, in certain cases, ^{1}H,^{13}C-gHSQCADTOXY was also used. Variable temperature NMR (VT-NMR) and ^{19}F,^{19}F{^{1}H}-NOESY/EXSY NMR were used to confirm cases of rotational isomerism. In certain cases, to localise fluorine atoms to their specific sugar, ^{19}F,^{13}C-gc2hsqcse was employed. To confirm configurations at anomeric centres in fluorinated sugars, coupling constants from ^{19}F-NMR spectra were used. For non-fluorinated sugars, the anomeric $^{1}J_{CH}$ couplings from the proton coupled ^{13}C spectrum or proton coupled ^{1}H,^{13}C-HSQC spectrum were used. Mass spectra were measured by the MS service at the Institute of Organic Chemistry, University of Münster. HRMS-ESI spectra were measured with Thermo Fisher Orbitrap Velos Pro, Thermo Fisher LTQ Orbitrap XL, and Thermo Fisher Exploris 120 Electrospray Orbitrap spectrometers. MALDI-MS spectra were measured with an Autoflex Speed MALDI-TOF spectrometer. IR spectra were recorded using a Shimadzu IRSpirit with QATR-S and selected absorption bands are reported in wavenumbers (cm^{-1}). Specific optical rotation was recorded using a Jasco P-2000 polarimeter. Melting points were determined using a Büchi B-545 apparatus. Sonication was achieved with a Bandelin Sonorex RK 255 without deliberate heating, however, upon prolonged use the temperature of the water bath was found to reach a maximum of 60 °C.

Automated glycan synthesis was accomplished with a Glyconeer® 2.1 synthesiser operated using GlycoSoft software. Temperature control was facilitated by a Huber Unistat 705 cryostat. DCM and 1,4-dioxane were used dry, whereas MeOH, THF and DMF were HPLC grade. Linker-functionalised Merrifield resin was prepared as previously described.[3] Building blocks were co-evaporated with toluene thrice before being dried under high vacuum for a minimum of three hours and dissolved in dry DCM. All reagent solutions were prepared freshly under argon before a week of syntheses. The activator solutions were kept at 2 °C during the syntheses, the other solvents were kept at room temperature. All syntheses were carried out on a 12.5 µmol scale, with respect to acceptor sites on the resin. Photocleavage of the glycan chain from the resin was carried out in batch using a 365 nm

LED placed 2 cm below a 5 mL RBF containing the resin-bound sugar in DCM (2 mL). The solution was stirred for 8 hours at room temperature then filtered. Size exclusion purifications were performed with a YMC-Pack Diol 300, 150 × 8.0 mm, 3 µm column in an Agilent Infinity 1260 HPLC.

Automation: General Information

Reagent Solutions

All reagent solutions were prepared under a protective argon atmosphere.

Building block solution: building block (**4**, **8** or **9,** 0.10 mmol) in dry DCM (1 mL).

Acid wash solution: TMSOTf (450 µL) in dry DCM (40 mL). Kept at 2 °C during the synthesis.

Activator solution: recrystallised NIS (1.35 g) and TfOH (150 µL) in dry DCM/1,4-dioxane (2/1 v/v, 40 mL). Kept at 2 °C during the synthesis.[a]

Fmoc deprotection solution: piperidine (20 mL) in DMF (80 mL).

Pre-capping solution: pyridine (4 mL) in DMF (36 mL).

Capping solution: acetic anhydride (4 mL) and methanesulfonic acid (800 µL) in dry DCM (36 mL).

Automated Synthesis Modules

Module A: Resin Swelling

DCM (2 mL) swells the resin (12.5 µmol) in the reaction vessel (shielded from light) at room temperature for 30 minutes. The resin is then washed with DMF, THF, and DCM (3 × 2 mL, 25 seconds per wash). All reagent lines are washed and primed.

Module B: Acid Wash

The resin is swollen in DCM (2 mL) and cooled to -20 °C. The acid wash solution (1 mL) is added and incubated (2 × 90 seconds). The reaction vessel is drained and the resin is washed with DCM (2 mL, 25 seconds).

Module C: Thioglycoside Glycosylation Single

Two temperatures (T_1, T_2) and times (t_1, t_2) are chosen by the user for each glycosylation. DCM (2 mL) is added to the reaction vessel. The vessel is cooled/warmed to T_1 then the reaction vessel is drained. Building block solution (1 mL) is added followed by the dropwise addition of activator solution (1 mL). The reaction vessel contents are incubated at T_1 for t_1. Then the vessel is warmed to T_2 and incubated for t_2. The reaction vessel is then drained and the resin is washed with DCM/1,4-dioxane (1/1, v/v) (2 × 2 mL, 25 seconds per wash) and DCM (2 × 2 mL, 25 seconds per wash).

Module D: Thioglycoside Glycosylation Double

Two temperatures (T_1, T_2) and times (t_1, t_2) are chosen by the user for each glycosylation. DCM (2 mL) is added to the reaction vessel. The vessel is cooled/warmed to T_1 then the reaction vessel is drained.

[a] The comparatively high concentration of TfOH is required to obtain reproducible results with the commercial Glyconeer 2.1® instrument, despite negatively impacting BB stability at higher temperatures. Similar observations have been made by other groups working with this instrument and have been disclosed as verbal communication on the Glyconner User Meeting in April 2019 in Potsdam, Germany.

Building block solution (1 mL) is added followed by the dropwise addition of activator solution (1 mL). The reaction vessel contents are incubated at T_1 for t_1. Then the vessel is warmed to T_2 and incubated for t_2. The reaction vessel is then drained and the resin is washed with DCM/1,4-dioxane (1/1, v/v) (2 × 2 mL, 25 seconds per wash) and DCM (2 × 2 mL, 25 seconds per wash). Building block solution (1 mL) is added followed by the dropwise addition of activator solution (1 mL). The reaction vessel contents are incubated at T_1 for t_1. Then the vessel is warmed to T_2 and incubated for t_2. The reaction vessel is then drained and the resin is washed with DCM/1,4-dioxane (1/1, v/v) (2 × 2 mL, 25 seconds per wash) and DCM (2 × 2 mL, 25 seconds per wash).

Module E: Acidic Capping

At 25 °C, the resin is washed with DMF (3 × 2 mL, 25 seconds per wash). Pre-capping solution (2 mL) is added and incubated (1 minute). The reaction vessel is drained and the resin is washed with DCM (3 × 2 mL, 25 seconds per wash). Capping solution (2 mL) is added and incubated (20 minutes). The reaction vessel is drained and the resin is washed with DCM (3 × 2 mL, 25 seconds per wash).

Module F: Fmoc Deprotection

At 25 °C, the resin is washed with DMF (3 × 2 mL, 60 seconds per wash). Fmoc deprotection solution (2 mL) is added and the mixture is incubated (5 minutes). The reaction vessel is drained and the resin is washed with DMF (3 × 2 mL, 60 seconds per wash then 1 × 15 mL) then DCM (5 × 2 mL, 60 seconds per wash).

Post Automation Procedures

Method A: Photocleavage

Photocleavage of the glycan chain from the resin was carried out in batch using a 365 nm LED placed 2 cm below a 5 mL RBF containing the resin-bound sugar in DCM (2 mL). The solution was stirred for 8 hours at room temperature then filtered and the solvent was removed *in vacuo*.

Method B: Methanolysis

The impure trisaccharide was dissolved in DCM/MeOH (1/1, v/v, 2 mL) and NaOMe (10 mg) was added. The mixture was stirred at rt for 10 h then neutralised with Amberlyst^{TM}[H^+]. The solvent was removed *in vacuo*.

Method C: HPLC SEC Purification

A SEC column (YMC-Pack Diol 300, 150 × 8.0 mm, 3 µm) was equipped on an Agilent Infinity 1260 HPLC. Samples were dissolved in ^{i}PrOH (300 µL) and purified in multiple runs (100 µL injections with dilution when needed, to avoid damage from the HPLC taking in air). Fractions were combined based on MS. The following gradient was used with hexane and ^{i}PrOH:

Time (min)	Hexane (%)	^{i}PrOH (%)
0	98	2
2	98	2
16	90	10
36	90	10

45	88	12
75	88	12
96	80	20
130	80	20

Method D: Hydrogenolysis

The trisaccharide was dissolved in EtOAc/^{t}BuOH/H_2O (2/1/1, v/v/v, 2 mL) and pretreated Pd/C (same mass as sugar) was added. The flask was equipped with a H_2 balloon and stirred o/n. The mixture was filtered through a celite plug and the celite was washed with the same solvent mixture. Freeze drying yielded the pure product.

Method E: Hydrogenolysis with Sonication

The trisaccharide was dissolved in ^{t}BuOH/H_2O (2/1, v/v, 2 mL) and pretreated Pd/C (40 mg) was added. The flask was equipped with a H_2 balloon and sonicated for 6 h. The mixture was filtered through a celite plug and the celite was washed with the same solvent mixture. Freeze drying yielded the pure product.

Quantification of Resin Loading

Resin was functionalised as previously described.[4] The resin loading was quantified according to a literature known method.[5] Shielded from light, the functionalised Merrifield resin (78 mg, 35 µmol) was swollen in DCM (1 mL) for 1 h. Pyridine (80 µL) and FmocCl (80 mg, 0.31 mmol) were added and the mixture was stirred for 22 h. The resin was washed with DCM (6 × 1 mL) to remove excess FmocCl. A solution of DBU in DMF (2 %v/v, 2 mL) was added and the reaction stirred for 2.5 h before filtration. An aliquot (160 µL) of the filtrate was diluted to a total volume of 10 mL with MeCN. A UV/vis spectrum was measured and the absorbance at 294 nm and 304 nm to calculate the resin loading (Equation 1). The final loading value was the mean of both wavelengths over 3 independent experiments and was calculated as 0.23 mmol g^{-1}. The same method was used to obtain yields when Fmoc-protected sugars were glycosylated to the resin.

Equation 1: *Abs$_\lambda$ = Absorbance at λ nm, V = Volume, D = Dilution Factor, ε$_\lambda$ = Molar absorption coefficient at λ nm, m = Mass of resin, l = Path length.*

$$Fmoc\ loading_\lambda\ (mmol\ g^{-1}) = \frac{\mathrm{Abs}_\lambda \times V(L) \times D}{\varepsilon_\lambda\ (L\ mmol^{-1}\ cm^{-1}) \times m_{resin}\ (g) \times l\ (cm)}$$

$$\varepsilon_{294} = 8794\ L\ mol^{-1}\ cm^{-1} \qquad \varepsilon_{304} = 7624\ L\ mol^{-1}\ cm^{-1}$$

Building Block Synthesis

4-Methylphenyl 2,3,4,6-tetra-*O*-acetyl-1-thio-α-D-mannopyranoside (1)

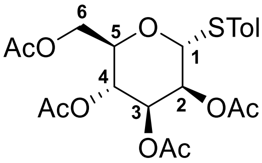

D-Mannose (2.70 g, 15.0 mmol, 1.0 eq.) was dissolved in pyridine (25 mL) then Ac_2O (25 mL) was added and the mixture was stirred at rt for 15 h. The mixture was concentrated *in vacuo* and diluted with DCM before being washed with sat. aqueous $NaHCO_3$ (2 × 15 mL) then aqueous HCl (1 M, 2 × 15 mL). The organic fraction was dried over $MgSO_4$ and the solvent was removed *in vacuo* and co-evaporated with toluene twice to yield a crude yellow syrup. The crude syrup and *p*-thiocresol (2.29 g, 18.4 mmol, 1.2 eq.) were dissolved in dry DCM (70 mL) under argon. The mixture was cooled to 0 °C before BF_3•Et_2O (5.69 mL, 46.1 mmol, 3.0 eq.) was added. The mixture was stirred for 15 h and allowed to warm to rt. The reaction was quenched by the addition of sat. aqueous $NaHCO_3$ followed by the addition of small portions of solid $NaHCO_3$ over several hours. Extraction with DCM (3×) and drying over $MgSO_4$ followed. Concentration *in vacuo* yielded a crude residue that was purified by column chromatography (SiO_2, CyH/EtOAc 9/1) to yield **1** (4.84 g, 71%) as a pale-yellow syrup.

R_f (CyH/EtOAc 2/1) 0.33; **$[\alpha]_D^{23}$** +97.5 (c = 2.0, $CHCl_3$); **^{1}H NMR** (599 MHz, $CDCl_3$) δ 7.39 – 7.36 (m, 2H, Tol), 7.13 – 7.11 (m, 2H, Tol), 5.50 – 5.48 (m, 1H, H-C2), 5.42 – 5.40 (m, 1H, H-C1), 5.33 – 5.31 (m, 2H, H-C3, H-C4), 4.57 – 4.53 (m, 1H, H-C5), 4.29 (dd, *J* = 12.2, 5.9 Hz, 1H, H-C6), 4.10 (dd, *J* = 12.2, 2.4 Hz, 1H, H-C6), 2.33 (s, 3H, Tol), 2.14 (s, 3H, Ac), 2.07 (s, 3H, Ac), 2.06 (s, 3H, Ac), 2.01 (s, 3H, Ac) ppm; **^{13}C NMR** (151 MHz, $CDCl_3$) δ 170.5 (C=O Ac), 169.9 (C=O Ac), 169.8 (C=O Ac), 169.7 (C=O Ac), 138.4 (Tol), 132.6 (Tol), 130.0 (Tol), 128.8 (Tol), 86.0 (C1), 70.9 (C2), 69.42 (C5), 69.36 (C3/C4), 66.4 (C3/C4), 62.5 (C6), 21.1 (Tol), 20.9 (Ac), 20.69 (Ac), 20.67 (Ac), 20.6 (Ac) ppm; **IR** (ATR) ṽmax / cm-1 2944 (w), 1745 (s), 1492 (w), 1435 (w), 1366 (m), 1217 (s), 1108 (m), 1050 (s), 976 (m), 809 (m), 752 (m); **HRMS** (ESI) m/z $[M+Na]^+$ calcd for $C_{21}H_{26}O_9SNa^+$ 477.1195; found 477.1182. Spectroscopic data are in accordance with the literature.[6]

4-Methylphenyl 2,3:4,6-di-*O*-benzylidene-1-thio-α-D-mannopyranoside (S1)

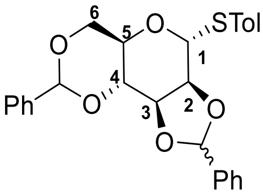

To a solution of **1** (8.90 g, 19.6 mmol, 1.0 eq.) in MeOH (150 mL) was added NaOMe (0.42 g, 7.83 mmol, 0.4 eq.) and the mixture was stirred at rt for 15 h. The mixture was neutralised with Amberlyst™[H^+] before concentration *in vacuo* and co-evaporation with toluene twice. The residue was suspended with NEt_3 (32.8 mL, 235 mmol, 12.0 eq.) in dry DCM (60 mL). The mixture was cooled under argon to 0 °C before the addition of TMSCl (14.9 mL, 118 mmol, 6.0 eq.). The mixture was stirred for 15 h and allowed to warm to rt before being quenched with water. The aqueous layer was extracted with DCM (×2), washed with brine and dried over $MgSO_4$. Concentration *in vacuo* yielded a yellow syrup that was then dried under high vacuum. The resulting syrup was dissolved in dry MeCN (150 mL) and cooled under argon to 0 °C before the addition of benzaldehyde (3.88 mL, 38.4 mmol, 2.1 eq.) followed by TMSOTf (0.33 mL, 1.83 mmol, 0.1 eq.). The mixture was stirred for 30 min and quenched with NEt_3 (1 mL). Filtration of the precipitate and washing with cold MeCN and pentane, followed by drying under high vacuum yielded **S1** as a mixture of isomers (7.69 g, 85%, *exo*/*endo*, 1/0.15) as an amorphous white solid.

R_f (CyH/EtOAc 4/1) 0.67; **^{1}H NMR** (599 MHz, CD_2Cl_2) δ 7.56 – 7.15 (m, 16.10, Ar), 6.28 (s, 1H, C*H*Ph dioxolane benzylidene *exo*), 5.97 (s, 0.15H, C*H*Ph dioxolane benzylidene *endo*), 5.85 (d, *J* = 0.6 Hz, 0.15H, H-C1 *endo*), 5.79 (d, *J* = 0.6 Hz, 1H, H-C1 *exo*), 5.64 (s, 1H, C*H*Ph dioxane benzylidene *exo*), 5.53 (s, 0.15H, C*H*Ph dioxane benzylidene *endo*), 4.66 (dd, *J* = 8.2, 5.3 Hz, 1H, H-C3 *exo*), 4.53 – 4.48 (m, 0.30H, H-C2 *endo*, H-C3/4 *endo*), 4.41 (dd, *J* = 5.2, 0.5 Hz, 1H, H-C2 *exo*), 4.33 (td, *J* = 10.0, 5.2 Hz, 1H, H-C5 *exo*), 4.29 (td, *J* = 10.0, 5.2 Hz, 0.15H, H-C5 *endo*), 4.23 (dd, *J* = 10.4, 5.2 Hz, 1H, H-C6 *exo*), 4.19 (dd, *J* = 10.4, 5.3 Hz, 0.15H, H-C6 *endo*), 3.98 (dd, *J* = 9.9, 8.3 Hz, 1H, H-C4 *exo*), 3.84 – 3.76 (m, 1.15H, H-C6 *exo*, H-C3/4 *endo*), 3.70 (t, *J* = 10.3 Hz, 0.15H, H-C6 *endo*), 2.36 (s, 0.45H, Tol *endo*), 2.35 (s, 3H, Tol *exo*) ppm; **^{13}C NMR** (151 MHz, CD_2Cl_2) δ 139.22 (Tol *endo*), 139.18 (Tol *exo*), 139.1 (Ph dioxolane *exo*), 137.90 (Ph dioxane *endo*), 137.85 (Ph dioxane *exo*), 137.4 (Ph dioxolane *endo*), 133.83 (Tol *endo*), 133.79 (Tol *exo*), 130.39 (Tol *endo*), 130.37 (Tol *exo*), 129.9 (Ph *endo*), 129.7 (Ph *exo*), 129.54 (Ph *exo*), 129.46 (Ph *endo*), 129.2 (Tol *exo*), 129.1 (Tol *endo*), 128.9 (Ph *endo*), 128.8 (Ph *exo*), 128.7 (Ph *exo*), 128.6 (Ph *endo*), 126.9 (Ph *endo*), 126.67 (Ph *exo*), 126.65 (Ph *endo*), 126.5 (Ph *exo*), 104.5 (dioxolane benzylidene *endo*), 103.5 (dioxolane benzylidene *exo*), 102.4 (dioxane benzylidene *exo*), 102.1 (dioxane benzylidene *endo*), 85.6 (C1 *exo*), 85.1 (C1 *endo*), 81.2 (C3/4 *endo*), 79.1 (C2 *endo*), 77.8 (C4 *exo*), 76.5 (C2 *exo*), 75.8 (C3 *exo*), 74.4 (C3/4 *endo*), 68.93 (C6 *exo*), 68.88 (C6 *endo*), 61.97 (C5 *endo*), 61.96 (C5 *exo*), 21.30 (Tol *endo*), 21.29 (Tol *exo*) ppm; **IR** (ATR) ṽmax / cm-1 3220 (w), 2904 (w), 1492 (w), 1377 (m), 1217 (w), 1091 (s), 1022 (s), 981 (s), 912 (m), 883 (m), 855 (w), 809 (m), 752 (s), 695 (s), 643 (m); **HRMS** (ESI) m/z $[M+Na]^+$ calcd for $C_{27}H_{26}O_5SNa^+$ 485.1399; found 485.1394. Spectroscopic data are in accordance with literature.[7]

4-Methylphenyl 3-*O*-benzyl-4,6-*O*-benzylidene-1-thio-α-D-mannopyranoside (S2)

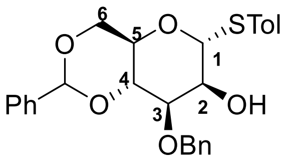

Dibenzylidene **S1** (8.56 g, 18.5 mmol, 1.0 eq.) was dissolved in DCM (120 mL) and cooled to -40 °C before the addition of DIBAL-H (1.0 M in Tol, 55.5 mL, 55.5 mmol, 3.0 eq.) under argon. The mixture was stirred for 12 h, allowed to warm to rt and then quenched by the cautious addition of water (10 mL) and aqueous NaOH (2 M, 2 mL) and filtered through $MgSO_4$. The retentate was crushed with DCM and re-filtered twice. After removal of the solvent *in vacuo*, column chromatography (SiO_2, CyH/EtOAc 9/1) afforded **S2** (5.80 g, 67%) as a colourless syrup. Regioisomer **S3** (0.91 g, 11%) was also isolated as a white solid.

R_f (CyH/EtOAc 4/1) 0.41; **m.p.** 134.7 °C; **$[\alpha]_D^{22}$** +137.3 (c = 0.5, DCM); **^{1}H NMR** (500 MHz, CD_2Cl_2) δ 7.53 – 7.48 (m, 2H, Ph), 7.44 – 7.29 (m, 10H, Ph, Tol, Bn), 7.19 – 7.14 (m, 2H, Tol), 5.62 (s, 1H, C*H*Ph), 5.49 (d, *J* = 1.2 Hz, 1H, H-C1), 4.85 (d, *J* = 11.7 Hz, 1H, CH_2Ph), 4.74 (d, *J* = 11.7 Hz, 1H, CH_2Ph), 4.35 (td, *J* = 9.9, 4.9 Hz, 1H, H-C5), 4.30 (dt, *J* = 3.1, 1.4 Hz, 1H, H-C2), 4.18 (dd, *J* = 10.3, 4.9 Hz, 1H, H-C6), 4.15 (t, *J* = 9.5 Hz, 1H, H-C4), 3.96 (dd, *J* = 9.6, 3.4 Hz, 1H, H-C3), 3.83 (t, *J* = 10.3 Hz, 1H, H-C6), 2.84 (d, *J* = 1.6 Hz, 1H, 2-OH), 2.35 (s, 3H, Tol) ppm; **^{13}C NMR** (126 MHz, CD_2Cl_2) δ 138.7 (Ar), 138.5 (Ar), 138.2 (Ar), 133.1 (Ar), 130.3 (Ar), 129.9 (Ar), 129.3 (Ar), 128.8 (Ar), 128.6 (Ar), 128.32 (Ar), 128.30 (Ar), 126.6 (Ar), 102.0 (CHPh), 88.9 (C1), 79.3 (C4), 76.3 (C3), 73.3 (CH_2Ph), 71.5 (C2), 68.9 (C6), 64.9 (C5), 21.3 (Tol) ppm; **IR** (ATR) ṽmax / cm-1 3421 (br), 2922 (w), 1493 (m), 1455 (s), 1377 (w), 1270 (w), 1099 (s), 1018 (m), 809 (m), 748 (m), 699 (m), 668 (w); **HRMS** (ESI) m/z $[M+Na]^+$ calcd for $C_{27}H_{28}O_5SNa^+$ 487.1555; found 487.1550. Spectroscopic data are in accordance with literature.[7]

4-Methylphenyl 2-*O*-benzyl-4,6-*O*-benzylidene-1-thio-α-D-mannopyranoside (S3)

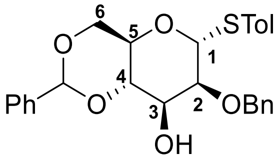

A solution of dibenzylidene **S1** (4.07 g, 8.80 mmol, 1.0 eq.) in dry DCM (100 mL) was cooled to -40 °C under argon then DIBAL-H (1.2 M in toluene, 22.0 mL, 26.4 mmol, 3.0 eq.) was added. The mixture was stirred for 3 h, allowed to warm to rt and then quenched by the cautious addition of H_2O and aqueous NaOH (2 M) and filtered through $MgSO_4$. The retentate was crushed with DCM and re-filtered twice. After removal of the solvent *in vacuo*, column chromatography (SiO_2, CyH/EtOAc 9/1) afforded **S3** (2.60 g, 64%) as a colourless syrup.

R$_f$ (CyH/EtOAc 4/1) 0.51; **m.p.** 112.0 °C; **[α]$_D^{22}$** +154.57 (c = 1.0, DCM); **^{1}H NMR** (599 MHz, CD_2Cl_2) δ 7.54 – 7.49 (m, 2H, Ar), 7.44 – 7.32 (m, 10H, Ar), 7.20 – 7.16 (m, 2H, Ar), 5.58 (s, 1H, C*H*Ph), 5.55 – 5.54 (m, 1H, H-C1), 4.73 (d, *J* = 11.5 Hz, 1H, CH_2Ph), 4.65 (d, *J* = 11.5 Hz, 1H, CH_2Ph), 4.32 (ddd, *J* = 10.2, 9.4, 5.0 Hz, 1H, H-C5), 4.20 (dd, *J* = 10.2, 4.9 Hz, 1H, H-C6), 4.12 – 4.06 (m, 2H, H-C2, H-C3), 3.96 (t, *J* = 9.5 Hz, 1H, H-C4), 3.82 (t, *J* = 10.3 Hz, 1H, H-C6), 2.50 (d, *J* = 8.2, Hz, 1H, 3-OH), 2.37 (s, 3H, Tol) ppm; **^{13}C NMR** (151 MHz, $CDCl_3$) δ 138.7 (Ar), 138.1 (Ar), 138.0 (Ar), 133.1 (Ar), 130.3 (Ar), 130.2 (Ar), 129.5 (Ar), 129.0 (Ar), 128.6 (Ar), 128.52 (Ar), 128.49 (Ar), 126.7 (Ar), 102.4 (CHPh), 87.1 (C1), 80.6 (C2), 80.0 (C4), 73.5 (CH_2Ph), 69.4 (C3), 68.8 (C6), 65.1 (C5), 21.3 (Tol) ppm; **IR** (ATR) ṽmax / cm-1 3490 (w), 3031 (w), 2870 (w), 1492 (m), 1452 (m), 1383 (m), 1303 (m), 1274 (m), 1211 (m), 1153 (m), 1090 (s), 1039 (s), 959 (m), 809 (m), 740 (s), 700 (s), 672 (m), 637 (m) ; **HRMS** (ESI) m/z $[M+Na]^+$ calcd for $C_{27}H_{28}O_5SNa^+$ 487.1555; found 487.1549.

4-Methylphenyl 3-*O*-benzyl-4,6-*O*-benzylidene-2-*O*-fluorenylmethoxycarbonyl-1-thio-α-D-mannopyranoside (S4)

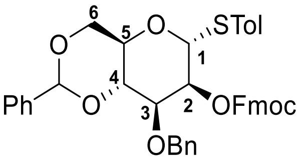

To a solution of **S2** (3.17 g, 6.82 mmol, 1.0 eq.) and FmocCl (3.52 g, 13.6 mmol, 2.0 eq.) in DCM (70 mL) was added pyridine (5.50 mL, 68.2 mmol, 10.0 eq.) and the solution was stirred at rt for 4 h. Aqueous HCl (1 M, 35 mL) was added and the aqueous layer was extracted with DCM (3 × 20 mL). The combined organic layers were washed with brine and dried over $MgSO_4$ before the solvent was removed *in vacuo*. Purification by column chromatography (SiO_2, 2% EtOAc in CyH) yielded **S4** (4.76 g, quant.) as a white foam.

R$_f$ (CyH/EtOAc 9/1) 0.43; **m.p.** 66.8 °C; **[α]$_D^{24}$** +74.1 (c = 0.5, DCM); **^{1}H NMR** (599 MHz, CD_2Cl_2) δ 7.81 – 7.78 (m, 2H, Fmoc), 7.64 – 7.60 (m, 2H, Fmoc), 7.53 – 7.49 (m, 2H, Fmoc), 7.44 – 7.24 (m, 14H, Ph, Fmoc, Bn, Tol), 7.19 – 7.15 (m, 2H, Tol), 5.67 (s, 1H, C*H*Ph), 5.48 (d, *J* = 1.4 Hz, 1H, H-C1), 5.43 (dd, *J* = 3.4, 1.4 Hz, 1H, H-C2), 4.74 – 4.71 (m, 2H, C*H$_2$*Ph), 4.50 (dd, *J* = 10.6, 6.9 Hz, 1H, CH_2 Fmoc), 4.45 (dd, *J* = 10.6, 7.2 Hz, 1H, CH_2 Fmoc), 4.37 (td, *J* = 9.8, 4.8 Hz, 1H, H-C5), 4.28 (t, *J* = 7.0 Hz, 1H, CH Fmoc), 4.22 (dd, *J* = 10.3, 4.9 Hz, 1H, H-C6), 4.16 (t, *J* = 9.7 Hz, 1H, H-C4), 4.05 (dd, *J* = 9.9, 3.4 Hz, 1H, H-C3), 3.89 (t, *J* = 10.3 Hz, 1H, H-C6), 2.35 (s, 3H, Tol) ppm; **^{13}C NMR** (151 MHz, CD_2Cl_2) δ 154.9 (C=O Fmoc), 143.9 (Fmoc), 143.7 (Fmoc), 141.73 (Fmoc), 141.69 (Fmoc), 139.2 (Tol), 138.4 (Ph), 138.1 (Ph), 133.3 (Tol), 130.4 (Tol), 129.5 (Tol), 129.4 (Ph), 128.7 (Bn), 128.6 (Ph), 128.31 (Fmoc), 128.28 (Fmoc), 128.09 (Bn), 128.08 (Bn), 127.62 (Fmoc), 127.60 (Fmoc), 126.6 (Ph), 125.5 (Fmoc), 125.4 (Fmoc), 120.5 (Fmoc), 102.1 (CHPh), 87.7 (C1), 78.9 (C4), 75.9 (C2), 74.6 (C3), 72.9 (CH_2Ph), 70.5 (CH_2 Fmoc), 68.7 (C6), 65.6 (C5), 47.2 (CH Fmoc), 21.3 (Tol) ppm; **IR** (ATR) ṽmax / cm-1 3039 (w), 2866 (w), 1747 (s), 1493 (w), 1451

(m), 1384 (m), 1256 (s), 1100 (s), 1017 (m), 959 (m), 806 (m), 740 (s), 697 (s); **HRMS** (ESI) m/z $[M+Na]^+$ calcd for $C_{42}H_{38}O_7SNa^+$ 709.2236; found 709.2231.

4-Methylphenyl 3,4-di-*O*-benzyl-2-*O*-fluorenylmethoxycarbonyl-1-thio-α-D-mannopyranoside (S5)

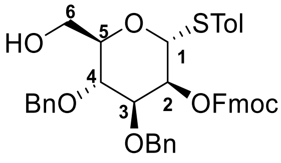

Under argon, a solution of **5** (7.15 g, 10.4 mmol, 1.0 eq.) in dry DCM (120 mL) was prepared and cooled to 0°C. BH_3•THF (1M in THF, 31.2 mL, 31.2 mmol, 3.0 eq.) was added followed by TMSOTf (0.56 mL, 3.12 mmol, 0.3 eq.) and the solution was stirred for 15 h, allowed to warm to rt. The reaction was quenched by the dropwise addition of H_2O (20 mL). The mixture was extracted with DCM and the organic layer was washed with brine, dried over $MgSO_4$ before the solvent was removed *in vacuo*. Purification by column chromatography (SiO_2, CyH/EtOAc 9/1) afforded **6** (6.63 g, 93%) as a white foam.

R_f (CyH/EtOAc 4/1) 0.41; **m.p.** 128 °C; **$[\alpha]_D^{23}$** +78.8 (c = 0.5, DCM); **^{1}H NMR** (599 MHz, $CDCl_3$) δ 7.79 – 7.76 (m, 2H, Fmoc), 7.64 – 7.59 (m, 2H, Fmoc), 7.43 – 7.21 (m, 16H, Fmoc, Bn, Tol), 7.16 – 7.12 (m, 2H, Fmoc), 5.50 (dd, *J* = 1.7, 0.6 Hz, 1H, H-C1), 5.46 (dd, *J* = 2.8, 1.7 Hz, 1H, H-C2), 4.98 (d, *J* = 10.9 Hz, 1H, C*H₂*Ph), 4.78 (d, *J* = 11.4 Hz, 1H, C*H₂*Ph), 4.70 (d, *J* = 10.9 Hz, 1H, C*H₂*Ph), 4.64 (d, *J* = 11.4 Hz, 1H, C*H₂*Ph), 4.46 (dd, *J* = 10.4, 7.3 Hz, 1H, CH_2 Fmoc), 4.34 (dd, *J* = 10.4, 7.9 Hz, 1H, CH_2 Fmoc), 4.28 – 4.22 (m, 2H, CH Fmoc, H-C5), 4.03 – 3.96 (m, 2H, H-C3, H-C4), 3.90 – 3.81 (m, 2H, H-C6), 2.34 (s, 3H, Tol), 1.79 (dd, *J* = 7.6, 5.8 Hz, 1H, C6-OH) ppm; **^{13}C NMR** (151 MHz, $CDCl_3$) δ 154.8 (C=O Fmoc), 143.6 (Fmoc), 143.3 (Fmoc), 141.5 (Fmoc), 141.4 (Fmoc), 138.6 (Tol), 138.3 (Bn), 137.7 (Bn), 133.1 (Tol), 130.2 (Tol), 129.3 (Tol), 128.6 (Bn), 128.5 (Bn), 128.2 (Bn), 128.11 (Bn), 128.06 (Bn), 128.03 (Fmoc), 128.02 (Fmoc), 127.96 (Bn), 127.31 (Fmoc), 127.30 (Fmoc), 125.5 (Fmoc), 125.3 (Fmoc), 120.18 (Fmoc), 120.17 (Fmoc), 86.5 (C1), 78.5 (C3), 75.6 (CH_2Ph), 74.5 (C4), 74.4 (C2), 73.1 (C5), 72.1 (CH_2Ph), 70.4 (Fmoc CH_2), 62.2 (C6), 46.8 (Fmoc CH), 21.3 (Tol) ppm; **IR** (ATR) ṽmax / cm-1 2926 (w), 1747 (s), 1494 (w), 1452 (m), 1386 (w), 1259 (s), 1101 (s), 1028 (m), 969 (w), 807 (w), 758 (m), 741 (s), 698 (m); **HRMS** (ESI) m/z $[M+Na]^+$ calcd for $C_{42}H_{40}O_7SNa^+$ 711.2392; found 711.2391.

4-Methylphenyl 6-*O*-acetyl-3,4-di-*O*-benzyl-2-*O*-fluorenylmethoxycarbonyl-1-thio-α-D-mannopyranoside (9)

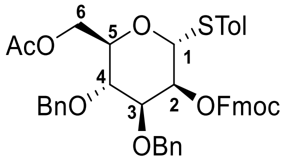

To a solution of **S5** (2.01 g, 2.92 mmol, 1.0 eq.) in DCM (30 mL) was added Ac_2O (1.66 mL, 17.5 mmol, 6.0 eq.) and pyridine (2.35 mL, 29.2 mmol, 10.0 eq.) and the mixture was stirred at rt for 15 h. The solution was diluted with DCM (150 mL) before the addition of sat. aqueous $NaHCO_3$ (4 mL) and then water (25 mL). The solution was separated and the organic layer was washed with aqueous HCl (1M, 25 mL). The organic layer was then washed with brine and dried over $MgSO_4$ before the solvent was removed *in vacuo* to give a colourless oil which crystallised upon standing. Purification by column chromatography (SiO_2, CyH/EtOAc 6/1) afforded **9** (1.92 g, 90%) as a white foam.

R_f (CyH/EtOAc 4/1) 0.57; **m.p.** 113.0 °C; **$[\alpha]_D^{23}$** +52.3 (c = 1.0, $CHCl_3$); **^{1}H NMR** (599 MHz, $CDCl_3$) δ 7.79 – 7.76 (m, 2H, Fmoc), 7.63 – 7.59 (m, 2H, Fmoc), 7.43 – 7.26 (m, 16H, Tol, Fmoc, Bn), 7.14 – 7.10 (m,

2H, Tol), 5.52 (d, *J* = 1.6 Hz, 1H, H-C1), 5.44 (dd, *J* = 3.1, 1.7 Hz, 1H, H-C2), 4.96 (d, *J* = 10.8 Hz, 1H, C*H₂*Ph), 4.79 (d, *J* = 11.3 Hz, 1H, C*H₂*Ph), 4.66 – 4.61 (m, 2H, C*H₂*Ph), 4.47 – 4.40 (m, 2H, H-C5, CH_2 Fmoc), 4.38 – 4.32 (m, 3H, CH_2 Fmoc, H-C6), 4.26 (t, *J* = 7.6 Hz, 1H, CH Fmoc), 4.01 (dd, *J* = 9.2, 3.1 Hz, 1H, H-C3), 3.89 (t, *J* = 9.5 Hz, 1H, H-C4), 2.33 (s, 3H, Tol), 2.04 (s, 3H, Ac) ppm; **^{13}C NMR** (151 MHz, $CDCl_3$) δ 170.9 (C=O Ac), 154.7 (C=O Fmoc), 143.6 (Fmoc), 143.3 (Fmoc), 141.5 (Fmoc), 141.4 (Fmoc), 138.5 (Tol), 138.0 (Bn), 137.6 (Bn), 132.9 (Tol), 130.1 (Tol), 129.4 (Tol), 128.6 (Bn), 128.6 (Bn), 128.3 (Bn), 128.2 (Bn), 128.09 (Ar), 128.07 (Ar), 128.05 (Ar), 128.0 (Ar), 127.30 (Fmoc), 127.29 (Fmoc), 125.4 (Fmoc), 125.3 (Fmoc), 120.22 (Fmoc), 120.18 (Fmoc), 86.2 (C1), 78.6 (C3), 75.5 (CH_2Ph), 74.4 (C4), 74.2 (C2), 72.1 (CH_2Ph), 70.9 (C5), 70.4 (CH_2 Fmoc), 63.5 (C6), 46.8 (CH Fmoc), 21.3 (Tol), 20.9 (Ac) ppm; **IR** (ATR) ṽmax / cm-1 2953 (w), 1743 (s), 1495 (w), 1452 (m), 1385 (w), 1367 (w), 1259 (s), 1103 (s), 1024 (m), 970 (w), 807 (w), 758 (m), 741 (s), 699 (m); **HRMS** (ESI) m/z $[M+Na]^+$ calcd for $C_{44}H_{42}O_8SNa^+$ 753.2498; found 753.2494.

4-Methyphenyl 6-*O*-acetyl-1-thio-α-D-mannopyranoside (2)

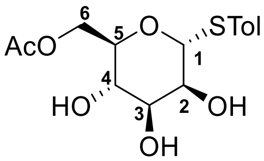

To a solution of **1** (1.59 g, 3.49 mmol, 1.0 eq.) in MeOH (50 mL) was added NaOMe (0.08 g, 1.40 mmol, 0.4 eq.) and the mixture was stirred at rt for 15 h. The mixture was neutralised with Amberlyst™$[H^+]$ before concentration *in vacuo* and co-evaporation with toluene (2×). A solution of the residue in freshly distilled 2,4,6-collidine (9.29 mL, 69.8 mmol, 20.0 eq.) was cooled to -40 °C under argon. After the dropwise addition of AcCl (0.30 mL, 4.19 mmol, 1.2 eq.) the mixture was stirred for 30 minutes before being quenched with methanol and left to warm to rt. The mixture was co-evaporated with toluene and the solvent was removed under reduced pressure before column chromatography (SiO_2, EtOAc/MeOH/H_2O 9/1/0 → 27/2/1). Crystallisation from EtOAc yielded **2** (0.70 g, 61.1%) as white needles.

R_f (EtOAc/MeOH/H_2O 8.5/1/0.5) 0.52; **m.p.** 133.7 °C; **$[α]_D^{23}$** +184.5 (c = 0.5, MeOH); **^{1}H NMR** (500 MHz, MeOD) δ 7.41 – 7.38 (m, 2H, Tol), 7.17 – 7.12 (m, 2H, Tol), 5.34 (d, *J* = 1.5 Hz, 1H, H-C1), 4.41 – 4.35 (m, 1H, H-C6), 4.28 – 4.21 (m, 2H, H-C5, H-C6), 4.07 (dd, *J* = 2.9, 1.6 Hz, 1H, H-C2), 3.71 – 3.65 (m, 2H, H-C3, H-C4), 2.32 (s, 3H, Tol), 2.00 (s, 3H, Ac) ppm; **^{13}C NMR** (126 MHz, MeOD) δ 172.7 (C=O Ac), 139.0 (Tol), 133.5 (Tol), 131.8 (Tol), 130.8 (Tol), 90.3 (C1), 73.4 (C2), 73.1 (C3), 72.9 (C5), 69.1 (C4), 65.1 (C6), 21.1 (Tol), 20.8 (Ac) ppm; **IR** (ATR) ṽmax / cm-1 3369 (br), 2921 (w), 1740 (m), 1493 (m), 1245 (m), 1101 (s), 1070 (s), 965 (w), 841 (w), 800 (m); **HRMS** (ESI) m/z $[M+Na]^+$ calcd for $C_{15}H_{20}O_6SNa^+$ 351.0873; found 351.0868. Spectroscopic data are in accordance with literature.[8,9]

4-Methyphenyl 2-*O*-acetyl-4,6-di-*O*-benzyl-1-thio-α-D-mannopyranoside (3)

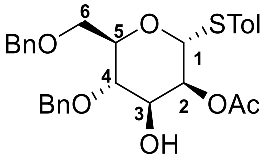

To a solution of **2** (0.85 g, 2.59 mmol, 1.0 eq.) in dry DMF (1 mL) was added $CH_3CH(OMe)_3$ (2.60 mL, 20.7 mmol, 8.0 eq.) then *p*-TsOH•H_2O (0.7 mg, 0.008 mmol, 0.003 eq.). The mixture was stirred for 3 h before the addition of NaH (60% in oil, 0.62 g, 15.5 mmol, 6.0 eq.) and cooling of the mixture to 0 °C. Benzyl bromide (0.92 mL, 7.77 mmol, 3.0 eq.) was added dropwise and the mixture was stirred for 1.5 h. After the addition of H_2O (0.02 mL), NaH (0.62 g, 15.5 mmol, 6.0 eq.) and DMF (1.6 mL) were added and the mixture was cooled to 0 °C. Benzyl bromide (0.46 mL, 3.89 mmol, 1.5 eq.)

was added dropwise and the reaction was stirred for 15 h before being slowly quenched with methanol (2.5 mL). The mixture was washed with aqueous LiCl (5%) before being extracted with EtOAc. The organic fraction was stirred with aqueous HCl (1 M) for 15 h then quenched with sat. aqueous $NaHCO_3$. The organic layer was washed with brine and dried over $MgSO_4$ before the solvent was removed *in vacuo*. Flash chromatography (SiO_2, CyH/EtOAc 2/1 → 3/2) yielded **3** (0.97 g, 74%) as a yellow syrup.

R_f (CyH/EtOAc 4/1) 0.20; **$[\alpha]_D^{23}$** +106.7 (c = 1.0, $CDCl_3$); **^{1}H NMR** (500 MHz, $CDCl_3$) δ 7.38 – 7.25 (m, 12H, Tol, Bn), 7.07 – 7.04 (m, 2H, Tol), 5.48 (d, *J* = 1.6 Hz, 1H, H-C1), 5.35 (dd, *J* = 3.5, 1.6 Hz, 1H, H-C2), 4.78 (d, *J* = 11.1 Hz, 1H, C*H*$_2$Ph), 4.69 (d, *J* = 11.9 Hz, 1H, C*H*$_2$Ph), 4.61 (d, *J* = 11.1 Hz, 1H, C*H*$_2$Ph), 4.49 (d, *J* = 11.9 Hz, 1H, C*H*$_2$Ph), 4.33 (ddd, *J* = 9.7, 4.1, 2.2 Hz, 1H, H-C5), 4.12 (dd, *J* = 9.3, 3.5 Hz, 1H, H-C3), 3.90 – 3.83 (m, 2H, H-C6, H-C4), 3.74 (dd, *J* = 11.0, 2.0 Hz, 1H, H-C6), 2.31 (s, 3H, Tol), 2.14 (s, 3H, Ac)ppm; **^{13}C NMR** (126 MHz, $CDCl_3$) δ 170.8 (C=O Ac), 138.3 (Bn), 138.2 (Bn), 138.0 (Tol), 132.5 (Tol), 129.98 (Tol), 129.95 (Tol), 128.7 (Bn), 128.5 (Bn), 128.1 (Bn), 128.02 (Bn), 128.01 (Bn), 127.8 (Bn), 86.5 (C1), 76.3 (C4), 75.1 (CH_2Ph), 74.1 (C2), 73.6 (CH_2Ph), 72.3 (C5), 71.1 (C3), 69.0 (C6), 21.24 (Tol), 21.20 (Ac) ppm; **IR** (ATR) ṽmax / cm-1 3469 (w), 3030 (w), 2866 (w), 1741 (s), 1494 (m), 1454 (m), 1372 (m), 1232 (s), 1099 (s), 1086 (s), 1075 (s), 1046 (s), 1028 (s), 1018 (s), 970 (m), 811 (m), 734 (s), 698 (s); **HRMS** (ESI) m/z $[M+Na]^+$ calcd for $C_{29}H_{32}O_6SNa^+$ 531.1812; found 531.1812. Spectroscopic data re in accordance with literature.[9]

4-Methyphenyl 2-*O*-acetyl-4,6-di-*O*-benzyl-3-*O*-fluorenylmethoxycarbonyl-1-thio-α-D-mannopyranoside (4)

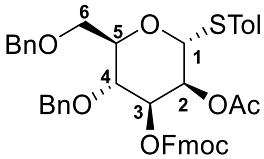

A solution of **3** (0.77 g, 1.51 mmol, 1.0 eq.) and FmocCl (0.78 g, 3.02 mmol, 2.0 eq.) in DCM (30 mL) was stirred at rt for 15 h before being washed with 1M aqueous HCl and extracted with DCM. The organic fraction was washed with brine and dried over $MgSO_4$. After the solvent was removed *in vacuo*, column chromatography (SiO_2, 2% EtOAc in CyH) yielded **4** (1.05 g, 95%) as an amorphous white solid.

R_f (CyH/EtOAc 4/1) 0.46; **m.p.** 52.6 °C; **$[\alpha]_D^{22}$** +77.3 (c = 1.0, $CHCl_3$); **^{1}H NMR** (500 MHz, $CDCl_3$) δ 7.79 – 7.75 (m, 2H, Fmoc), 7.64 – 7.56 (m, 2H, Fmoc), 7.43 – 7.19 (m, 16H, Fmoc, Bn, Tol), 7.09 – 7.05 (m, 2H, Tol), 5.63 (dd, *J* = 3.3, 1.7 Hz, 1H, H-C2), 5.49 (d, *J* = 1.7 Hz, 1H, H-C1), 5.21 (dd, *J* = 9.7, 3.3 Hz, 1H, H-C3), 4.78 (d, *J* = 11.0 Hz, 1H, C*H*$_2$Ph), 4.70 (d, *J* = 11.9 Hz, 1H, C*H*$_2$Ph), 4.56 (d, *J* = 11.0 Hz, 1H, C*H*$_2$Ph), 4.53 – 4.47 (m, 2H, C*H*$_2$Ph, CH_2 Fmoc), 4.42 (ddd, *J* = 9.8, 4.3, 1.8 Hz, 1H, H-C5), 4.38 (dd, *J* = 10.5, 7.7 Hz, 1H, CH_2 Fmoc), 4.28 (t, *J* = 7.3 Hz, 1H, CH Fmoc), 4.13 (t, *J* = 9.8 Hz, 1H, H-C4), 3.89 (dd, *J* = 11.0, 4.2 Hz, 1H, H-C6), 3.73 (dd, *J* = 11.0, 1.9 Hz, 1H, H-C6), 2.32 (s, 3H, Tol), 2.17 (s, 3H, Ac) ppm; **^{13}C NMR** (126 MHz, $CDCl_3$) δ 170.2 (C=O Ac), 154.3 (C=O Fmoc), 143.7 (Fmoc), 143.3 (Fmoc), 141.5 (Fmoc), 141.4 (Fmoc), 138.23 (Bn), 138.16 (Tol), 138.0 (Bn), 132.6 (Tol), 130.0 (Tol), 129.6 (Tol), 128.53 (Ar), 128.48 (Ar), 128.04 (Ar), 127.99 (Ar), 127.98 (Ar), 127.93 (Ar), 127.86 (Ar), 127.8 (Ar), 127.31 (Fmoc), 127.29 (Fmoc), 125.3 (Fmoc), 125.2 (Fmoc), 120.19 (Fmoc), 120.17 (Fmoc), 86.2 (C1), 76.7 (C3), 75.3 (CH_2Ph), 73.6 (CH_2Ph), 73.3 (C4), 72.5 (C5), 71.5 (C2), 70.4 (CH_2 Fmoc), 68.7 (C6), 46.8 (CH Fmoc), 21.3 (Tol), 21.1 (Ac) ppm; **IR** (ATR) ṽmax / cm-1 3035 (w), 2923 (w), 1751 (s), 1495 (w), 1451 (m), 1374 (w), 1226 (s), 1262 (s), 1101 (s), 1051 (s), 971 (w), 739 (s); **HRMS** (ESI) m/z $[M+Na]^+$ calcd for $C_{44}H_{42}O_8SNa^+$ 753.2493; found 753.2481.

1,3,4,6-Tetra-*O*-acetyl-2-deoxy-2-fluoro-α-D-mannopyranoside (S6)

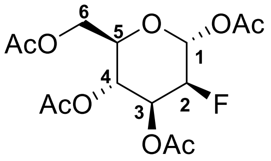

D-Glucal (25.0 g, 91.83 mmol, 1.0 eq.) was dissolved in acetone (410 mL) and water (82 mL) before Selectfluor® (39.0 g, 110 mmol, 1.2 eq.) was added. The mixture was stirred at rt for 48 h before concentration *in vacuo*. The aqueous residue was extracted with DCM (3×) and concentrated *in vacuo* to yield a colourless syrup that was dissolved in pyridine (300 mL) before the addition of Ac_2O (34.0 mL, 367 mmol, 4.0 eq.) then DMAP (1.12 g, 9.18 mmol, 0.1 eq.). The mixture was stirred for 15 h at rt then quenched with aqueous sat. $NaHCO_3$ and extracted with DCM (3×). A crude residue was obtained after concentration *in vacuo* that was purified by column chromatography (SiO_2, CyH/EtOAc 8/1) to yield **S6** (12.75 g, 40%) as colourless crystals.

R_f (CyH:EtOAc 4:1) 0.28; **m.p.** 84.2 °C; **$[\alpha]_D^{21}$** +49.4 (c = 1.0, DCM); **^{1}H** NMR (500 MHz, $CDCl_3$) δ 6.27 (dd, *J* = 6.5, 2.1 Hz, 1H, H-C1), 5.41 (td, *J* = 10.2, 1.0 Hz, 1H, H-C4), 5.26 (ddd, *J* = 27.9, 10.2, 2.6 Hz, 1H, H-C3), 4.75 (dt, *J* = 48.7, 2.4 Hz, 1H, H-C2), 4.28 (dd, *J* = 12.5, 4.5 Hz, 1H, H-C6), 4.12 (dd, *J* = 12.5, 2.4 Hz, 1H, H-C6), 4.05 (ddd, *J* = 10.1, 4.5, 2.4 Hz, 1H, H-C5), 2.17 (s, 3H, Ac), 2.11 (s, 3H, Ac), 2.09 (s, 3H, Ac), 2.05 (s, 3H, Ac) ppm; **^{13}C NMR** (126 MHz, $CDCl_3$) δ 170.8 (C=O), 170.4 (C=O), 169.4 (C=O), 168.2 (C=O), 90.3 (d, *J* = 31.1 Hz, C1), 86.1 (d, *J* = 182.1 Hz, C2), 70.9 (C5), 69.6 (d, *J* = 17.0 Hz, C3), 65.3 (d, *J* = 1.3 Hz, C4), 61.9 (C6), 21.0 (Ac), 20.85 (Ac), 20.84 (Ac), 20.7 (Ac) ppm; **^{19}F NMR** (470 MHz, $CDCl_3$) δ -203.84 (dddt, *J* = 48.7, 27.9, 6.5, 0.9 Hz) ppm; **IR** (neat) ṽmax / cm-1 2961 (w), 1747 (s), 1436 (w), 1373 (m), 1218 (s), 1150 (m), 1092 (w), 1058 (w), 1022 (m), 980 (m), 896 (w), 803 (w), 682 (w); **HRMS** (ESI) m/z $[M^+Na]^+$ calcd for $C_{14}H_{19}FO_9Na^+$ 373.0905; found 373.0905. Spectroscopic data are in accordance with literature.[10]

4-Methylphenyl 3,4,6-tri-*O*-acetyl-2-deoxy-2-fluoro-1-thio-α-D-mannopyranoside (S7)

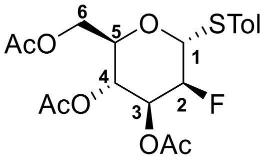

A solution of **S6** (6.53 g, 18.6 mmol, 1.0 eq.) and *p*-thiocresol (2.78 g, 22.4 mmol, 1.2 eq.) in dry DCM (70 mL) was cooled to 0 °C under argon. Boron trifluoride diethyl etherate (6.90 mL, 55.9 mmol, 3.0 eq.) was added cautiously and the mixture was allowed to warm to room temperature while stirring for 48 h. The reaction was quenched by the addition of sat. aqueous $NaHCO_3$ followed by the addition of small portions of solid $NaHCO_3$ over several hours. The aqueous layer was extracted with DCM (3 × 50 mL) and the combined organic fractions were washed with brine and dried over $MgSO_4$. The solvent was removed under reduced pressure. Purification by column chromatography (SiO_2, CyH/EtOAc 9/1) yielded **S7** (4.96 g, 64%) as a yellow syrup.

R_f (CyH:EtOAc 9:1) 0.53; **$[\alpha]_D^{22}$** +157.0 (c = 1.0, $CHCl_3$); **^{1}H NMR** (599 MHz, $CDCl_3$) δ 7.40 – 7.36 (m, 2H, Tol), 7.17 – 7.12 (m, 2H, Tol), 5.57 (dd, *J* = 14.4, 1.8 Hz, 1H, H-C1), 5.38 (t, *J* = 10.1 Hz, 1H, H-C4), 5.22 (ddd, *J* = 28.2, 10.1, 2.5 Hz, 1H, H-C3), 5.04 (ddd, *J* = 49.9, 2.5, 1.9 Hz, 1H, H-C2), 4.51 (ddd, *J* = 10.2, 5.4, 2.3 Hz, 1H, H-C5), 4.30 (dd, J = 12.3, 5.4 Hz, 1H, H-C6), 4.11 (dd, J = 12.3, 2.3 Hz, 1H, H-C6), 2.34 (s, 3H, Tol), 2.11 (s, 3H, Ac), 2.07 (s, 3H, Ac), 2.07 (s, 3H, Ac). ppm; **^{13}C NMR** (151 MHz, $CDCl_3$) δ 170.6 (C=O), 170.1 (C=O), 169.5 (C=O), 138.7 (Tol), 132.6 (Tol), 130.1 (Tol), 128.4 (Tol), 88.3 (d, *J* = 188.8 Hz, C2), 85.9 (d, *J* = 22.4 Hz, C1), 70.2 (d, *J* = 17.6 Hz, C3), 69.5 (C5), 66.0 (d, *J* = 1.3 Hz, C4), 62.2 (C6), 21.1 (Tol), 20.70 (Ac), 20.68 (Ac), 20.6 (Ac) ppm; **^{19}F NMR** (564 MHz, $CDCl_3$) δ -189.72 (ddd, *J* = 49.9, 28.1, 14.4 Hz) ppm; **IR** (neat) ṽmax / cm-1 2940 (w), 1746 (s), 1494 (m), 1436 (w), 1369 (s), 1225 (s), 1104 (s),

1061 (s), 1018 (w), 977 (w), 917 (w), 860 (w), 811 (w), 795 (w), 771 (w); **HRMS** (ESI) m/z [M$^+$Na]$^+$ calcd for $C_{19}H_{23}FO_7SNa^+$ 437.1046; found 437.1034. Spectroscopic data are in accordance with literature.[4]

4-Methylphenyl 4,6-*O*-benzylidene-2-deoxy-2-fluoro-1-thio-α-D-mannopyranoside (5)

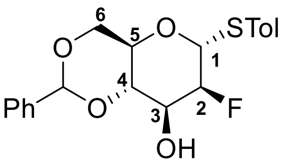

A solution of **S7** (4.90 g, 11.8 mmol, 1.0 eq.) and NaOMe (0.26 g, 4.73 mmol, 0.4 eq.) in MeOH (80 mL) was stirred at rt for 14 h. The pale-yellow solution was neutralised with Amberlyst^{TM}[H$^+$] and the solvent was removed *in vacuo* before co-evaporation with toluene. The resulting white amorphous solid was dissolved in MeCN (100 mL) to which was added benzaldehyde dimethyl acetal (7.10 mL, 47.2 mmol, 4.0 eq.) and *p*-toluenesulfonic acid (0.20 g, 1.18 mmol, 0.1 eq.). The suspension was stirred at rt for 48 h. The yellow solution was neutralised with NEt_3 and the solvent was removed under reduced pressure. Column chromatography (SiO_2, 5% EtOAc in CyH) yielded **5** (3.10 g, 70%) as a white foam.

R$_f$ (CyH/EtOAc 4/1) 0.31; **m.p.** 155.5 °C; **[α]$_D$^{22}** +305.1 (c = 1.0, $CHCl_3$); **^{1}H NMR** (599 MHz, $CDCl_3$) δ 7.54 – 7.50 (m, 2H, Ph), 7.43 – 7.35 (m, 5H, Ar Ph, Tol), 7.18 – 7.13 (m, 2H, Tol), 5.61 (s, 1H, C*H*Ph), 5.57 (dd, *J* = 15.2, 1.6 Hz, 1H, H-C1), 5.06 (ddd, *J* = 49.4, 2.7, 1.6 Hz, 1H, H-C2), 4.34 (td, *J* = 9.8, 4.9 Hz, 1H, H-C5), 4.27 (dd, *J* = 10.3, 5.0 Hz, 1H, H-C6), 4.15 (dddd, *J* = 27.0, 10.0, 5.5, 2.7 Hz, 1H, H-C3), 3.99 (td, *J* = 9.7, 1.7 Hz, 1H, H-C4), 3.84 (t, *J* = 10.2 Hz, 1H, H-C6), 2.46 (d, *J* = 5.5 Hz, 1H, 3-OH), 2.35 (s, 3H, Tol) ppm; **^{13}C NMR** (126 MHz, $CDCl_3$) δ 138.8 (Tol), 137.1 (Ph), 132.9 (Tol), 130.3 (Tol), 129.5 (Ph), 129.0 (Tol), 128.5 (Ph), 126.4 (Ph), 102.5 (CHPh), 91.3 (d, *J* = 186.7 Hz, C2), 87.2 (d, *J* = 23.5 Hz, C1), 79.1 (d, *J* = 2.1 Hz, C4), 68.7 (d, *J* = 17.9 Hz, C3), 68.5 (C6), 64.8 (C5), 21.3 (Tol) ppm; **^{19}F NMR** (470 MHz, CDCl3) δ -190.06 (ddd, *J* = 49.6, 26.9, 15.4 Hz) ppm; **IR** (ATR) ṽmax / cm-1 3358 (w), 2952 (w), 1713 (m), 1641 (w), 1494 (m), 1381 (m), 1278 (m), 1219 (m), 1197 (m), 1098 (s), 1073 (s), 1037 (s), 1021 (s), 1000 (s), 976 (s), 914 (m), 859 (s), 811 (s), 794 (s), 775 (s), 749 (s), 699 (s), 675 (s); **HRMS** (ESI) m/z [M+Na]$^+$ calcd for $C_{20}H_{21}FO_4SNa^+$ 399.1042; found 399.1035. Spectroscopic data are in accordance with literature.[4]

4-Methylphenyl 4,6-*O*-benzylidene-3-*O*-fluorenylmethoxycarbonyl-2-deoxy-2-fluoro-1-thio-α-D-mannopyranoside (6)

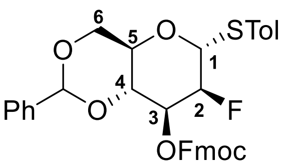

To a solution of **5** (3.10 g, 8.24 mmol, 1.0 eq.) and FmocCl (4.26 g, 16.5 mmol, 2.0 eq.) in DCM (70 mL) was added pyridine (6.65 mL, 82.4 mmol, 10.0 eq.). The resulting solution was stirred at rt for 15 h. Aqueous HCl (1 M) was added to neutralise the solution before extraction with DCM (3 × 20 mL). The combined organic fractions were washed with brine and dried over $MgSO_4$ and the solvent was removed *in vacuo*. Column chromatography (SiO_2, 2% EtOAc in CyH) yielded **6** (4.58 g, 93%) as a white foam.

R$_f$ (CyH:EtOAc 4:1) 0.56; **m.p.** 67.7 °C; **[α]$_D$^{22}** +153.9 (c = 1.0, MeOH); **^{1}H NMR** (500 MHz, CD_2Cl_2) δ 7.83 – 7.76 (m, 2H, Fmoc), 7.64 – 7.56 (m, 2H, Fmoc), 7.49 – 7.45 (m, 2H, Ph), 7.45 – 7.34 (m, 7H, Fmoc, Tol, Ph), 7.30 (td, *J* = 7.5, 1.2 Hz, 1H, Fmoc), 7.26 (td, *J* = 7.5, 1.1 Hz, 1H, Fmoc), 7.22 – 7.16 (m, 2H, Tol), 5.61 (s, 1H, CHPh), 5.58 (dd, *J* = 15.0, 1.5 Hz, 1H, H-C1), 5.20 – 5.12 (m, 2H, H-C2, H-C3), 4.52 (dd, *J* = 10.6, 7.0 Hz, 1H, CH_2 Fmoc), 4.48 (dd, *J* = 10.6, 7.0 Hz, 1H, CH_2 Fmoc), 4.46 – 4.40 (m, 1H, H-C5), 4.32 –

4.24 (m, 2H, CH Fmoc, H-C6), 4.21 (t, *J* = 9.8 Hz, 1H, H-C4), 3.87 (t, *J* = 10.3 Hz, 1H, H-C6), 2.35 (s, 3H, Tol) ppm; **^{13}C NMR** (126 MHz, CD_2Cl_2) δ 154.6 (C=O Fmoc), 143.8 (Fmoc), 143.6 (Fmoc), 141.70 (Fmoc), 141.68 (Fmoc), 139.5 (Tol), 137.6 (Ph), 133.4 (Tol), 130.5 (Tol), 129.6 (Ph), 128.8 (Tol), 128.6 (Ph), 128.3 (Fmoc), 127.6 (Fmoc), 126.7 (Ph), 125.43 (Fmoc), 125.41 (Fmoc), 120.5 (Fmoc), 102.4 (CHPh), 89.4 (d, *J* = 188.5 Hz, C2), 87.4 (d, *J* = 23.2 Hz, C1), 76.2 (d, *J* = 1.9 Hz, C4), 73.5 (d, *J* = 17.1 Hz, C3), 70.6 (CH_2 Fmoc), 68.7 (C6) 65.6 (C5), 47.1 (CH Fmoc), 21.3 (Tol) ppm; **^{19}F NMR** δ -188.44 – -188.74 (m) ppm; **IR** (neat) ṽmax / cm-1 3032 (w), 2911 (w), 1726 (s), 1468 (w), 1419 (m), 1274 (m), 1234 (s), 1218 (s), 1064 (s), 976 (m), 951 (s), 927 (s), 871 (w), 774 (w), 701 (m), 669 (s), 653 (s); **HRMS** (ESI) m/z $[M^{+}Na]^{+}$ calcd for $C_{35}H_{31}FO_6SNa^{+}$ 621.1718; found 621.1717. Spectroscopic data are in accordance with literature.[4]

4-Methylphenyl 4-*O*-benzyl-3-*O*-fluorenylmethoxycarbonyl-2-deoxy-2-fluoro-1-thio-α-D-mannopyranoside (7)

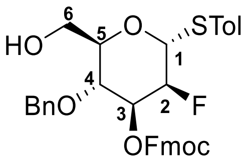

Benzylidene **6** (4.56 g, 7.62 mmol, 1.0 eq.) was dissolved in dry DCM (80 mL) under argon and cooled to 0 °C. Borane (1 M in THF, 22.9 mL, 22.9 mmol, 3.0 eq.) was added, followed by TMSOTf (0.41 mL, 2.29 mmol, 0.3 eq.) and the resulting colourless solution was stirred for 72 h at 0 °C. The reaction was quenched by the cautious addition of H_2O. The aqueous layer was extracted with DCM (2 × 20 mL) and the combined organic fractions were washed with brine and dried over $MgSO_4$ before the solvent was removed *in vacuo*. Column chromatography (SiO_2, CyH/EtOAc 9/1) yielded **7** (2.90 g, 63%) as a white foam.

R_f (CyH/EtOAc 4/1) 0.41; **m.p.** 47.3 °C; **$[\alpha]_D^{22}$** +136.7 (c = 1.0, DCM); **^{1}H NMR** (599 MHz, $CDCl_3$) δ 7.80 – 7.75 (m, 2H, Fmoc), 7.65 – 7.59 (m, 2H, Fmoc), 7.43 – 7.39 (m, 2H, Fmoc), 7.39 – 7.36 (m, 2H, Tol), 7.35 – 7.27 (m, 7H, Fmoc, Bn), 7.17 – 7.13 (m, 2H, Tol), 5.55 (dd, *J* = 14.5, 1.9 Hz, 1H, H-C1), 5.22 – 5.08 (m, 2H, H-C2, H-C3), 4.83 (d, *J* = 11.1 Hz, 1H, C*H*$_2$Ph), 4.70 (d, *J* = 11.1 Hz, 1H, C*H*$_2$Ph), 4.51 – 4.41 (m, 2H, CH_2 Fmoc), 4.31 – 4.22 (m, 2H, H-C5, CH Fmoc), 4.12 (t, *J* = 9.9 Hz, 1H, H-C4), 3.89 – 3.80 (m, 2H, H-C6), 2.35 (s, 3H, Tol) ppm; **^{13}C NMR** (151 MHz, $CDCl_3$) δ 154.5 (C=O Fmoc), 143.4 (Fmoc), 143.3 (Fmoc), 141.47 (Fmoc), 141.45 (Fmoc), 138.8 (Tol), 137.7 (Bn), 133.1 (Tol), 130.3 (Tol), 128.8 (Tol), 128.7 (Bn), 128.2 (Bn), 128.1 (Bn, Fmoc), 127.37 (Fmoc), 125.3 (Fmoc), 125.2 (Fmoc), 120.3 (Fmoc), 88.8 (d, *J* = 186.9 Hz, C2), 86.2 (d, *J* = 22.0 Hz, C1), 77.1 (d, *J* = 17.6 Hz, C3), 75.5 (CH_2Ph), 73.2 (C5), 72.6 (d, *J* = 1.3 Hz, C4), 70.5 (CH_2 Fmoc), 61.7 (C6), 46.8 (CH Fmoc), 21.3 (Tol) ppm; **^{19}F NMR** (470 MHz, $CDCl_3$) δ -189.96 (ddd, *J* = 49.9, 28.1, 14.3 Hz) ppm; **IR** (ATR) ṽmax / cm-1 3454 (w), 2925 (w), 1750 (s), 1493 (m), 1451 (m), 1260 (s), 1087 (s), 1100 (s), 968 (m), 799 (m), 760 (m), 739 (s); **HRMS** (ESI) m/z $[M+Na]^{+}$ calcd for $C_{35}H_{33}FO_6SNa^{+}$ 623.1874 ; found 623.1870. Spectroscopic data in accordance with literature.[4]

4-Methylphenyl 6-*O*-acetyl-4-*O*-benzyl-3-*O*-fluorenylmethoxycarbonyl-2-deoxy-2-fluoro-1-thio-α-D-mannopyranoside (8)

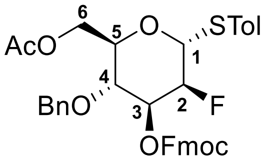

To a solution of **17** (0.10 g, 0.17 mmol, 1.0 eq.) and pyridine (0.14 mL, 1.70 mmol, 10.0 eq.) in DCM (2 mL) was added Ac_2O (0.07 mL, 0.68 mmol, 4.0 eq.). The mixture was stirred at rt for 15 h before the addition of sat. aqueous $NaHCO_3$. The organic layer was washed with aqueous HCl (1 M), then brine and dried over $MgSO_4$ before the solvent was removed under reduced pressure. Column chromatography (SiO_2, CyH/EtOAc 9/1) yielded **8** (0.11 g, quant.) as a white foam.

R_f (CyH/EtOAc 4/1) 0.54; **m.p.** 47.0 °C; **$[\alpha]_D^{22}$** +167.1 (c = 0.5, DCM); **^{1}H NMR** (500 MHz, $CDCl_3$) δ 7.80 – 7.75 (m, 2H, Fmoc), 7.65 – 7.59 (m, 2H, Fmoc), 7.43 – 7.26 (m, 11H, Tol, Bn, Fmoc), 7.15 – 7.11 (m, 2H, Tol), 5.56 (dd, *J* = 14.4, 1.7 Hz, 1H, H-C1), 5.22 – 5.04 (m, 2H, H-C2, H-C3), 4.81 (d, *J* = 11.0 Hz, 1H, CH_2Ph), 4.61 (d, *J* = 11.1 Hz, 1H, CH_2Ph), 4.53 – 4.41 (m, 3H, H-C5, CH_2 Fmoc), 4.36 – 4.31 (m, 2H, H-C6), 4.28 (t, *J* = 7.3 Hz, 1H, CH Fmoc), 4.01 (t, *J* = 9.9 Hz, 1H, H-C4), 2.34 (s, 3H, Tol), 2.04 (s, 3H, Ac) ppm; **^{13}C NMR** (126 MHz, $CDCl_3$) δ 170.8 (C=O Ac), 154.4 (C=O Fmoc), 143.3 (Fmoc), 143.2 (Fmoc), 141.49 (Fmoc), 141.47 (Fmoc), 138.7 (Tol), 137.4 (Bn), 132.9 (Tol), 130.2 (Tol), 128.8 (Tol), 128.7 (Bn), 128.3 (Bn), 128.2 (Bn), 128.14 (Fmoc), 128.13 (Fmoc), 127.4 (Fmoc), 125.3 (Fmoc), 125.2 (Fmoc), 120.3 (Fmoc), 88.6 (d, *J* = 187.2 Hz, C2), 86.0 (d, *J* = 22.1 Hz, C1), 77.3 (d, *J* = 17.7 Hz, C3), 75.4 (CH_2Ph), 72.8 (C4), 70.8 (C5), 70.5 (CH_2 Fmoc), 63.0 (C6), 46.8 (CH Fmoc), 21.3 (Tol), 20.9 (Ac) ppm; **^{19}F NMR** (470 MHz, $CDCl_3$) δ -190.09 (ddd, *J* = 50.3, 28.4, 14.4 Hz) ppm; **IR** (ATR) ṽmax / cm-1 2912 (w), 1749 (w), 1452 (w), 1261 (m), 1237 (m), 1089 (s), 879 (s), 741 (s), 626 (s); **HRMS** (ESI) m/z $[M+Na]^+$ calcd for $C_{37}H_{35}FO_7SNa^+$ 665.1980; found 665.1977.

Synthesis of Standards

Heptyl 6-*O*-acetyl-3,4-di-*O*-benzyl-2-*O*-fluorenylmethoxycarbonyl-α-D-mannopyranoside (10)

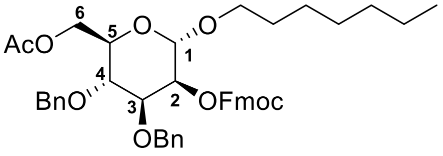

A solution of **9** (74.0 mg, 0.10 mmol, 1.0 eq.) in dry DCM (1.5 mL) was prepared under argon and cooled to -20 °C after freshly powdered dry-rite was added. NIS (28.3 mg, 0.13 mmol, 1.25 eq.) was added followed by TfOH (4.5 µL, 0.05 mmol, 0.5 eq.). The mixture was left to stir for 20 mins then allowed to warm to rt over 30 mins before being quenched with sat. aqueous $Na_2S_2O_3$. The mixture was extracted with DCM (3×) and the organic fractions were combined and washed with brine before being dried over $MgSO_4$. Evaporation of the solvent *in vacuo* afforded the crude product that was purified using column chromatography (SiO_2, CyH/EtOAc 9/1) to yield **10** (47 mg, 74%) as a colourless syrup.

Rf (CyH/EtOAc 4/1) 0.60; **$[\alpha]_D^{23}$** +10.3 (c = 2.0, $CHCl_3$); **^{1}H NMR** (599 MHz, $CDCl_3$) δ 7.80 – 7.76 (m, 2H, Ar), 7.66 – 7.61 (m, 2H, Ar), 7.44 – 7.39 (m, 2H, Ar), 7.37 – 7.32 (m, 4H, Ar), 7.32 – 7.28 (m, 5H, Ar), 7.27 – 7.21 (m, 3H, Ar), 5.21 (dd, *J* = 3.3, 1.9 Hz, 1H, H-C2), 4.98 – 4.91 (m, 2H, H-C1, C*H₂*Ph), 4.78 (d, *J* = 11.2 Hz, 1H, C*H₂*Ph), 4.65 – 4.59 (m, 2H, C*H₂*Ph), 4.46 (dd, *J* = 10.4, 7.3 Hz, 1H, CH_2 Fmoc), 4.40 – 4.31 (m, 3H, H-C6, CH_2 Fmoc), 4.26 (t, *J* = 7.6 Hz, 1H, CH Fmoc), 4.04 (dd, *J* = 8.8, 3.2 Hz, 1H, H-C3), 3.91 – 3.81 (m, 2H, H-C4, H-C5), 3.68 (dt, *J* = 9.6, 6.8 Hz, 1H, OCH_2), 3.44 (dt, *J* = 9.6, 6.6 Hz, 1H, OCH_2), 2.08 (s, 3H, Ac), 1.62 – 1.54 (m, 2H, CH_2), 1.35 – 1.28 (m, 8H, CH_2), 0.92 – 0.88 (m, 3H, CH_3) ppm; **^{13}C NMR** (151 MHz, $CDCl_3$) δ 171.0 (C=O Ac), 154.9 (C=O Fmoc), 143.7 (Fmoc), 143.4 (Fmoc), 141.5 (Fmoc), 141.4 (Fmoc), 138.1 (Bn), 138.0 (Bn), 128.6 (Ar), 128.5 (Ar), 128.3 (Ar), 128.1 (Ar), 128.0 (Ar), 127.9 (Ar), 127.29 (Ar), 127.28 (Ar), 125.5 (Ar), 125.3 (Ar), 120.20 (Ar), 120.16 (Ar), 97.6 (C1), 78.4 (C3), 75.5 (CH_2Ph), 74.2 (C4), 72.7 (C2), 72.0 (CH_2Ph), 70.4 (CH_2 Fmoc), 69.9 (C5), 68.4 (OCH_2), 63.5 (C6), 46.8 (CH Fmoc), 31.9 (CH_2), 29.5 (CH_2), 29.2 (CH_2), 26.2 (CH_2), 22.8 (CH_2), 21.0 (Ac), 14.2 (CH_3) ppm; **IR** (ATR) ṽmax / cm-1 2921 (m), 2359 (m), 1745 (s), 1452 (m), 1263 (s), 1079 (m), 740 (m), 700 (w); **HRMS** (ESI) m/z $[M+Na]^+$ calcd for $C_{44}H_{50}O_9Na^+$ 745.3353; found 745.3348.

6-*O*-Acetyl-3,4-di-*O*-benzyl-2-*O*-fluorenylmethoxycarbonyl-α-D-mannopyranose (11)

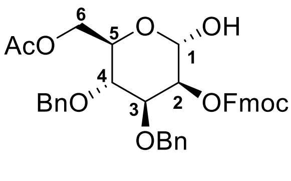

11 was isolated as a colourless syrup during the purification of multiple solution-phase glycosylations that had used **9** as a donor. Reported as the α-product below, however, the NMR also showed small traces of β-product

R_f (CyH/EtOAc 4/1) 0.10; **^{1}H NMR** (599 MHz, $CDCl_3$) δ 7.79 – 7.76 (m, 2H, Ar), 7.65 – 7.60 (m, 2H, Ar), 7.43 – 7.38 (m, 2H, Ar), 7.36 – 7.27 (m, 10H, Ar), 7.25 – 7.21 (m, 2H, Ar), 5.35 (d, *J* = 1.9 Hz, 1H, H-C1), 5.23 (dd, *J* = 3.2, 1.9 Hz, 1H, H-C2), 4.95 (d, *J* = 10.9 Hz, 1H, C*H₂*Ph), 4.77 (d, *J* = 11.4 Hz, 1H, C*H₂*Ph), 4.64 – 4.60 (m, 2H, C*H₂*Ph), 4.46 (dd, *J* = 10.4, 7.3 Hz, 1H, CH_2 Fmoc), 4.41 (dd, *J* = 12.0, 2.2 Hz, 1H, H-C6), 4.33 (dd, *J* = 10.4, 7.9 Hz, 1H, CH_2 Fmoc), 4.30 (dd, *J* = 12.0, 5.1 Hz, 1H, H-C6), 4.26 (t, *J* = 7.5 Hz, 1H, CH Fmoc), 4.12 – 4.08 (m, 2H, H-C3, H-C5), 3.85 (t, *J* = 9.6 Hz, 1H, H-C4), 2.07 (s, 3H, Ac) ppm; **^{13}C NMR** (151 MHz, $CDCl_3$) δ 171.1 (C=O Ac), 154.9 (C=O Fmoc), 143.6 (Fmoc), 143.3 (Fmoc), 141.5 (Fmoc), 141.4 (Fmoc), 138.1 (Bn), 137.9 (Bn), 128.6 (Ar), 128.5 (Ar), 128.3 (Ar), 128.08 (Ar), 128.07 (Ar), 128.0 (Ar), 127.9 (Ar), 127.30 (Ar), 127.28 (Ar), 125.5 (Ar), 125.3 (Ar), 120.22 (Ar),

120.18 (Ar), 92.4 (C1), 77.7 (C3), 75.5 (CH_2Ph), 74.1 (C4), 72.8 (C2), 72.0 (CH_2Ph), 70.4 (CH_2 Fmoc), 70.2 (C5), 63.5 (C6), 46.8 (CH Fmoc), 21.0 (Ac) ppm; **IR** (ATR) ṽmax / cm-1 3427 (w), 2927 (w), 1745 (s), 1452 (m), 1383 (m), 1251 (s), 1073 (s), 1027 (s), 970 (m), 740 (s), 700 (s); **HRMS** (ESI) m/z $[M+Na]^+$ calcd for $C_{37}H_{36}O_9Na^+$ 647.2257; found 647.2254.

6-*O*-Acetyl-3,4-di-*O*-benzyl-2-*O*-fluorenylmethoxycarbonyl-α-D-mannopyranosyl-(1→1)-6-*O*-acetyl-3,4-di-*O*-benzyl-2-*O*-fluorenylmethoxycarbonyl-α-D-mannopyranoside (12)

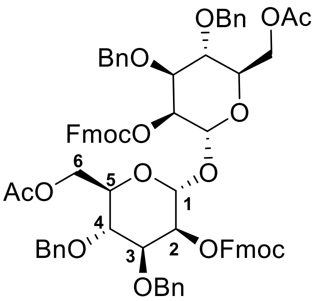

12 was isolated as a colourless syrup during the purification of multiple solution-phase glycosylations that had used **9** as a donor.

R$_f$ (CyH:EtOAc 4:1) 0.27; **$[\alpha]_D^{23}$** +14.8 (c = 0.67, $CHCl_3$); **^{1}H NMR** (599 MHz, $CDCl_3$) δ 7.80 – 7.75 (m, 4H, Ar), 7.64 – 7.58 (m, 4H, Ar), 7.43 – 7.39 (m, 4H, Ar), 7.37 – 7.19 (m, 24H, Ar), 5.24 (d, *J* = 1.9 Hz, 2H, H-C1), 5.04 (dd, *J* = 3.1, 1.9 Hz, 2H, H-C2), 4.97 (d, *J* = 10.7 Hz, 2H, C*H$_2$*Ph), 4.75 (d, *J* = 11.3 Hz, 2H, C*H$_2$*Ph), 4.64 (d, *J* = 10.7 Hz, 2H, C*H$_2$*Ph), 4.61 (d, *J* = 11.3 Hz, 2H, C*H$_2$*Ph), 4.47 (dd, *J* = 10.3, 7.2 Hz, 2H, CH_2 Fmoc), 4.38 (dd, *J* = 11.9, 2.0 Hz, 2H, H-C6), 4.34 – 4.29 (m, 4H, H-C6, C*H$_2$*Ph), 4.25 (t, *J* = 7.6 Hz, 2H, CH Fmoc), 3.91 (dd, *J* = 8.7, 3.1 Hz, 2H, H-C3), 3.89 – 3.81 (m, 4H, H-C4, H-C5), 2.02 (s, 6H, Ac) ppm; **^{13}C NMR** (151 MHz, $CDCl_3$) δ 170.9 (C=O Ac), 154.7 (C=O Fmoc), 143.5 (Fmoc), 143.2 (Fmoc), 141.5 (Fmoc), 141.4 (Fmoc), 137.9 (OBn), 137.6 (OBn), 128.7 (Ar), 128.6 (Ar), 128.4 (Ar), 128.2 (Ar), 128.14 (Ar), 128.12 (Ar), 128.10 (Ar), 128.08 (Ar), 127.3 (Ar), 125.4 (Ar), 125.2 (Ar), 120.3 (Ar), 120.2 (Ar), 93.0 (C1), 77.6 (C3), 75.7 (CH_2Ph), 73.8 (C4), 72.24 (C2), 72.18 (CH_2Ph), 71.0 (C5), 70.5 (CH_2 Fmoc), 63.0 (C6), 46.8 (CH Fmoc), 20.9 (Ac) ppm; **IR** (ATR) ṽmax / cm-1 2950 (w), 2359 (m), 1745 (s), 1452 (w), 1257 (s), 1148 (w), 1102 (w), 1022 (m), 740 (m); **HRMS** (ESI) m/z $[M^+Na]^+$ calcd for $C_{74}H_{70}O_{17}Na^+$ 1253.4511; found 1253.4505.

Methyl 6-*O*-acetyl-3,4-di-*O*-benzyl-2-*O*-fluorenylmethoxycarbonyl-α-D-mannopyranoside (13)

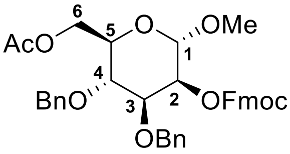

13 was isolated as a colourless syrup during the purification of multiple solution-phase glycosylations that had used **9** as a donor.

R$_f$ (CyH:EtOAc 4:1) 0.34; **$[\alpha]_D^{23}$** +9.3 (c = 0.67, $CHCl_3$); **^{1}H NMR** (599 MHz, $CDCl_3$) δ 7.80 – 7.76 (m, 2H, Ar), 7.65 – 7.59 (m, 2H, Ar), 7.44 – 7.39 (m, 2H, Ar), 7.36 – 7.20 (m, 12H, Ar), 5.20 (dd, *J* = 3.3, 1.8 Hz, 1H, H-C2), 4.94 (d, *J* = 10.9 Hz, 1H, C*H$_2$*Ph), 4.83 (d, *J* = 1.9 Hz, 1H, H-C1), 4.76 (d, *J* = 11.3 Hz, 1H, C*H$_2$*Ph), 4.64 – 4.56 (m, 2H, C*H$_2$*Ph), 4.45 (dd, *J* = 10.4, 7.3 Hz, 1H, CH_2 Fmoc), 4.41 – 4.31 (m, 3H, H-C6, CH_2 Fmoc), 4.26 (t, *J* = 7.6 Hz, 1H, CH Fmoc), 4.04 – 4.00 (m, 1H, H-C3), 3.87 – 3.80 (m, 2H, H-C4, H-C5), 3.38 (s, 3H, OMe), 2.08 (s, 3H, Ac) ppm; **^{13}C NMR** (151 MHz, $CDCl_3$) δ 171.0 (C=O Ac), 154.9 (C=O Fmoc), 143.6 (Fmoc), 143.3 (Fmoc), 141.5 (Fmoc), 141.4 (Fmoc), 138.2 (OBn), 137.9 (OBn), 128.6 (Ar), 128.5 (Ar), 128.3 (Ar), 128.1 (Ar), 128.03 (Ar), 128.02 (Ar), 128.01 (Ar), 127.9 (Ar), 127.30 (Ar), 127.29 (Ar), 125.5 (Ar), 125.3 (Ar), 120.21 (Ar), 120.18 (Ar), 98.6 (C1), 78.3 (C3), 75.4 (CH_2Ph), 74.1 (C4), 72.5 (C2), 71.9 (CH_2Ph), 70.4 (CH_2 Fmoc), 69.8 (C5), 63.5 (C6), 55.2 (OMe), 46.8 (CH Fmoc), 21.0 (Ac) ppm; **IR** (ATR) ṽmax / cm-1 2921 (w), 2365 (w), 1745 (s), 1452 (m), 1263 (s), 1142 (m), 1073 (m), 976 (w), 740 (m); **HRMS** (ESI) m/z $[M+Na]^+$ calcd for $C_{38}H_{38}O_9Na^+$ 661.2414; found 661.2407.

Methyl 6-*O*-acetyl-3,4-di-*O*-benzyl-2-*O*-fluorenylmethoxycarbonyl-β-D-mannopyranoside (14)

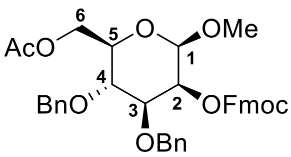

14 was isolated as a colourless syrup during the purification of multiple solution-phase glycosylations that had used **9** as a donor.

R$_f$ (CyH:EtOAc 4:1) 0.16; **[α]$_D$^{23}** -7.0 (c = 0.33, $CHCl_3$); **^{1}H NMR** (599 MHz, $CDCl_3$) δ 7.79 – 7.75 (m, 2H, Ar), 7.69 – 7.63 (m, 2H, Ar), 7.43 – 7.38 (m, 2H, Ar), 7.36 – 7.27 (m, 9H, Ar), 7.24 – 7.21 (m, 3H, Ar), 5.43 (dd, *J* = 3.1, 1.0 Hz, 1H, H-C2), 4.94 (d, *J* = 10.8 Hz, 1H, C*H$_2$*Ph), 4.81 (d, *J* = 11.4 Hz, 1H, C*H$_2$*Ph), 4.62 (d, *J* = 10.8 Hz, 1H, C*H$_2$*Ph), 4.57 (d, *J* = 11.4 Hz, 1H, C*H$_2$*Ph), 4.47 (d, *J* = 1.0 Hz, 1H, H-C1), 4.43 – 4.40 (m, 3H, CH_2 Fmoc, H-C6), 4.34 – 4.28 (m, 2H, CH Fmoc, H-C6), 3.82 (t, *J* = 9.5 Hz, 1H, H-C4), 3.72 (dd, *J* = 9.2, 3.2 Hz, 1H, H-C3), 3.55 (s, 4H, H-C5, OMe), 2.06 (s, 3H, Ac) ppm; **^{13}C NMR** (151 MHz, $CDCl_3$) δ 171.0 (C=O Ac), 155.4 (C=O Fmoc), 143.7 (Fmoc), 143.6 (Fmoc), 141.44 (Fmoc), 141.41 (Fmoc), 138.0 (OBn), 137.5 (OBn), 128.7 (Ar), 128.6 (Ar), 128.4 (Ar), 128.2 (Ar), 128.1 (Ar), 128.02 (Ar), 127.97 (Ar), 127.95 (Ar), 127.29 (Ar), 127.27 (Ar), 125.6 (Ar), 125.5 (Ar), 120.12 (Ar), 120.10 (Ar), 99.8 (C1), 80.3 (C3), 75.5 (CH_2Ph), 74.1 (C4), 73.6 (C5), 72.2 (C2), 71.7 (CH_2Ph), 70.4 (CH_2 Fmoc), 63.5 (C6), 57.4 (OMe), 46.8 (CH Fmoc), 22.8 (Ac) ppm; **IR** (ATR) ṽmax / cm-1 2927 (w), 1739 (w), 1452 (w), 1263 (w), 1217 (m), 746 (s), 666 (m); **HRMS** (ESI) m/z $[M+Na]^+$ calcd for $C_{38}H_{38}O_9Na^+$ 661.2414; found 661.2407.

Glycosylation Optimisation

Temperature of Activation and Decomposition: Building Block 9

Temperatures of activation ($T_{A,}$ lowest temperature at which the building block is partially activated) and decomposition (T_D, lowest temperature where building block is fully activated) were determined using an adapted literature procedure.[11] Though the T_A and T_D of **9** was previously reported, these temperatures are also influenced strongly by factors such as acid concentration.[12] As this work employed an acid concentration differing to that previously published, it was necessary to establish the system-specific T_A and T_D.

A solution of **9** (40 mg, 0.055 mmol, 1.0 eq.) and NIS (33.7 mg, 0.150 mmol, 2.7 eq.) in dry DCM (2 mL) was cooled to the desired temperature (maintained using a cryostat). TfOH (3.75 µL, 0.042 mmol, 0.76 eq.) was added. After 5 minutes, the reaction was quenched by the addition of a solution of pyridine in DMF (1/9 v/v, 1 mL) and sat. aqueous $Na_2S_2O_3$ (10 mL). After warming to rt, the mixture was extracted with DCM (2 × 2 mL) then dried over $MgSO_4$. Filtration and removal of solvent under reduced pressure yielded a crude residue that was co-evaporated twice with toluene and dried under high vacuum. The crude mixtures were analysed via ^{1}H-NMR spectroscopy. The results revealed that for BB **9**: T_A = -50 °C, T_D = -20 °C (Figure S1).

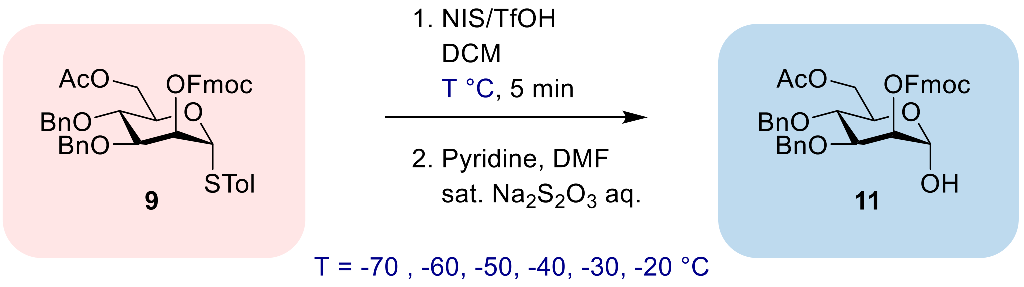

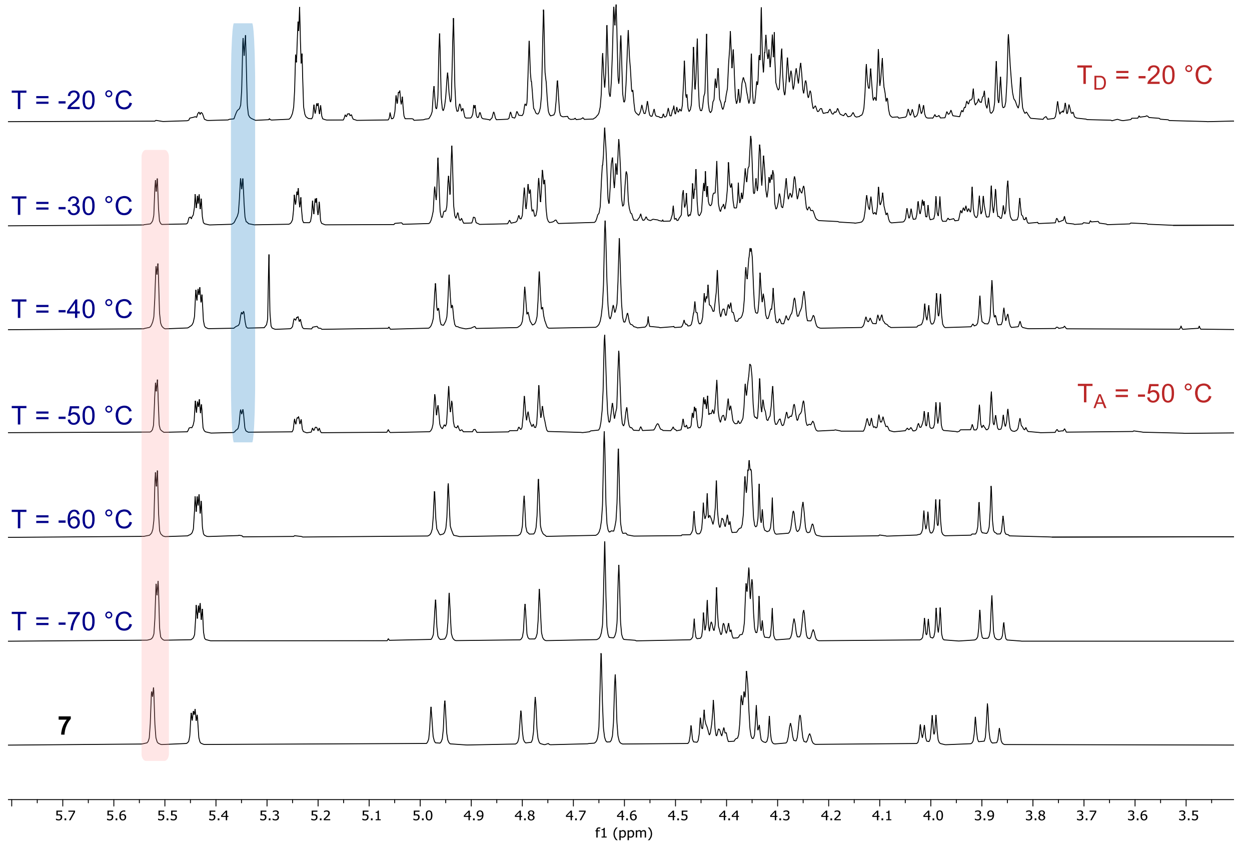

Figure S1. Temperature of activation and decomposition of BB **9** from crude ^{1}H-NMR spectra.

Automated Solution-Phase Glycosylation

A stock solution of dry 1-heptanol in dry DCM was prepared (19.3 µL heptanol in 20 mL dry DCM, 0.795 mg/mL). Building block **9** was co-evaporated three times with toluene and dried under high vacuum with freeze-drying. The heptanol stock solution (1 mL, 6.84 µmol, 1.0 eq.) and building block **9** (40 mg, 54.7 µmol, 8.0 eq.) in dry DCM (1 mL) were added to a vial. This resulted in a 2 mL solution containing heptanol and the building block in a 1/8 ratio. The vials were loaded into the Glyconeer® and a modified synthesis was programmed: an acid wash then the glycosylation step (1200 s at the desired temperature: 0 °C to -35 °C at 5 °C intervals). In the absence of solid-support, the reaction mixture was flushed into a waiting test tube charged with aqueous $Na_2S_2O_3$ to quench the NIS. The mixture was extracted (3× DCM) and washed with brine before being dried over $MgSO_4$ before being concentrated *in vacuo*. NMR spectra were recorded of these crude mixtures and the ^{1}H,^{13}C-HSQC cross-peaks were matched to pure standards and integrated to obtain their relative amounts within mixture (Table S1).

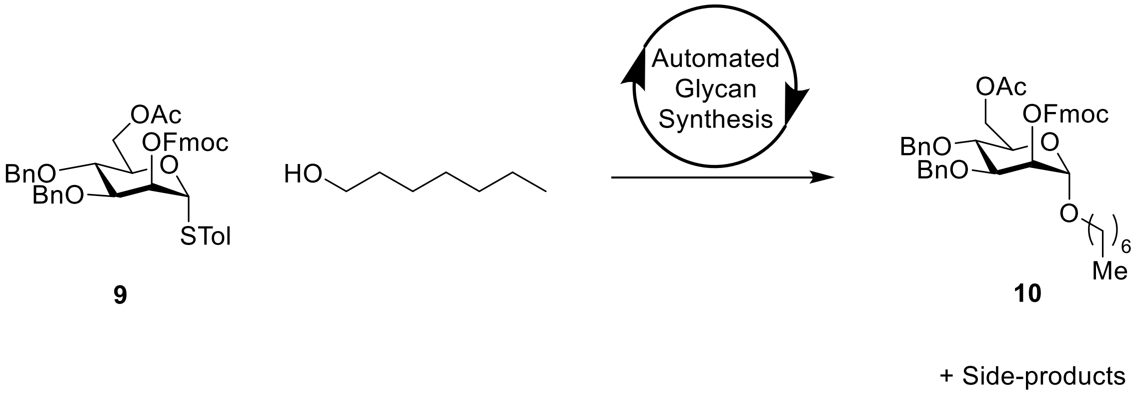

Module	Conditions
B: Acid Wash	
D: Thioglycoside Glycosylation Single	BB **9** (8 eq.), 1-heptanol (1 eq.), X °C for 1200 s

Table S1: *Proportions of various compounds in crude reaction mixture following glycosylation of BB **9** to heptanol. Proportions are relative to the sum of integrals of all cross-peaks in the anomeric region of the ^{1}H,^{13}C-HSQC spectrum, defined as 85 ppm – 102 ppm in the ^{13}C dimension and 6.40 ppm – 4.40 ppm in the ^{1}H dimension.*

	Relative Proportion of Compounds in Crude							
	-35 °C	-30 °C	-25 °C	-20 °C	-15 °C	-10 °C	-5 °C	0 °C
α-Heptanol Product **10**	14.09	13.60	14.52	14.71	10.46	15.36	13.01	20.40
α-Hydrolysis Product **11**	45.45	43.86	40.32	32.68	21.79	18.73	10.66	8.03
α,α-Dimer **12**	1.36	4.82	8.47	10.13	17.21	18.54	19.51	21.37
Unassigned Species	23.18	32.89	35.48	40.85	49.89	46.25	56.18	50.20

Solution Phase Glycosylation: BB 8

The same strategy was applied to survey glycosylation conditions for BB **8** in the temperature range of -40 to 20°C in 10°C intervals. Due to signal overlap, the exact assignment of different species could not be performed. However, the activation and decomposition temperatures could be determined as -30 and 10°C, respectively (Figure S2).

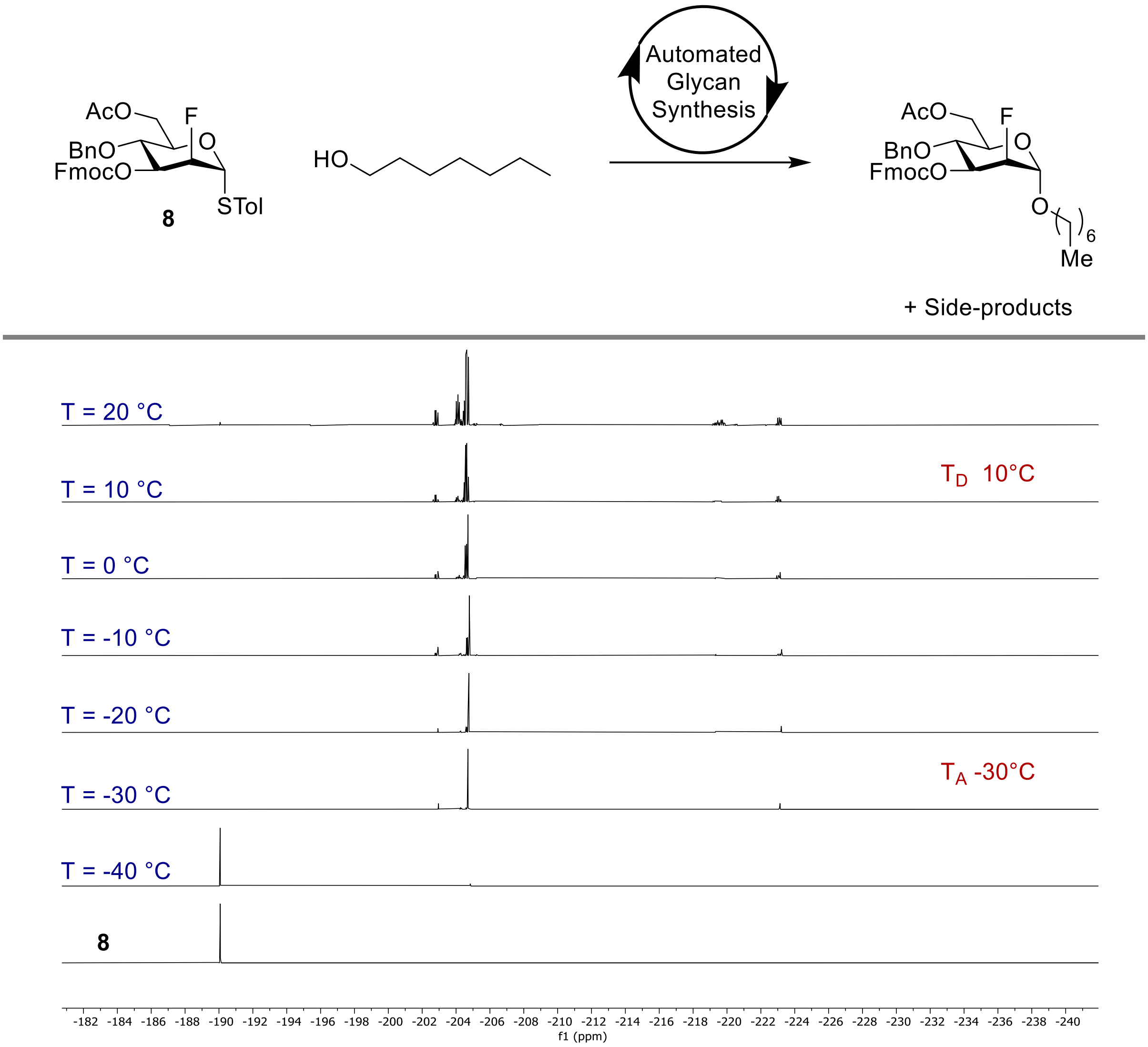

Figure S2. Temperature of activation and decomposition of BB **8** from crude ^{19}F-NMR spectra.

Solid Phase Glycosylation: BB 9

Quantification of Fmoc as described for the quantification of resin loading was used to establish the yields of solid-phase glycosylations (Table S2).

Table S2. *Optimisation of the glycosylation of BB **9** to the resin-bound linker in the Glyconeer 2.1®.*

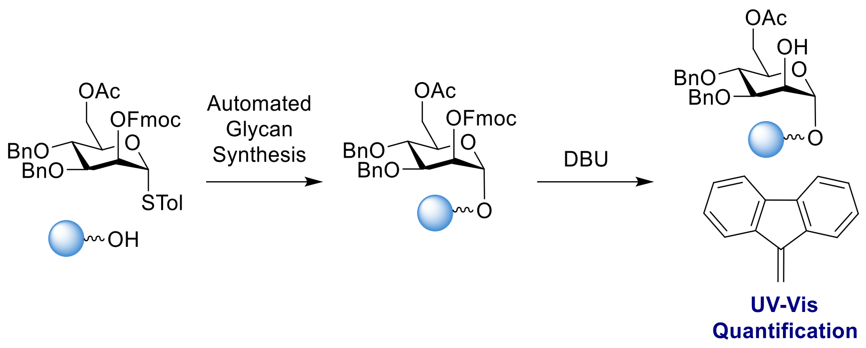

Entry	T_1 (°C)	t_1 (s)	T_2 (°C)	t_2 (s)	Eq.	Loading* (mmol/g)	Yield** (%)
1	-20	300	0	1200	6	0.12	52
2	-20	300	0	1200	8	0.17	74
3	-35	300	-25	1200	8	0.13	57
4	-20	300	0	1200	2 × 5	0.21	91
5	-35	300	-25	1200	2 × 5	0.16	70
6	-20	300	0	1200	2 × 8	0.23	Quant.

*Loading is an average of 304 nm and 294 nm

**Yield calculated based on 0.23 mmol g^{-1} maximum loading

Solid Phase Glycosylation: BB 8

Quantification of Fmoc as described for the quantification of resin loading was used to establish the yields of solid-phase glycosylations (Table S3). It was found that significant 4-OBn cleavage was observed above 10 °C for BB **8**.

Table S3. *Optimisation of the glycosylation of BB **8** to the resin-bound linker in the Glyconeer 2.1®.*

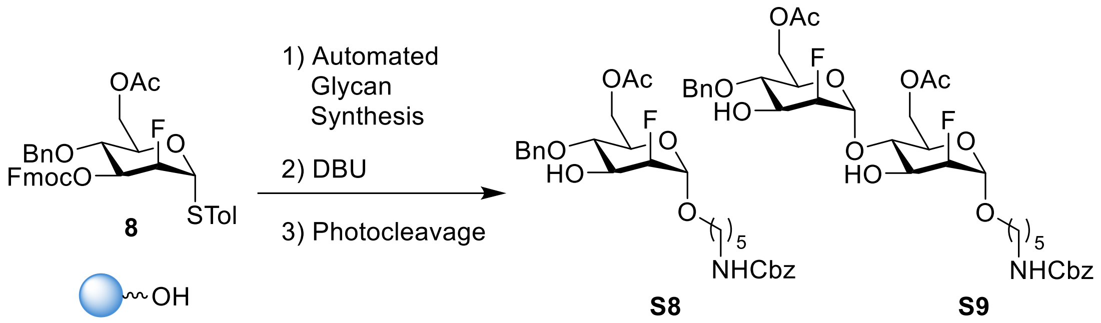

Entry	T_1[a] (°C)	T_2[b] (°C)	Proportion of **S8** signals in ^{19}F-NMR[c] (%)	**S9**/**S8** ratio[d]
1	-30	-30	23	n.d.
2	-20	-20	56	0.01
3	-10	-10	75	0.04
4	0	0	75	0.03
5	10	10	76	0.04
6	0	20	50	0.21

[a] Incubation time at T_1 was 300 s. [b] Incubation time at T_2 was 1800 s. [c] Proportion of ^{19}F peaks from the product cannot be considered a yield as some compounds in the mixture result in multiple ^{19}F peaks, e.g. **S9**. It is still a useful metric to assess the reactivity. [d] Calculated using ^{19}F-NMR.

Automated Glycan Synthesis

_N_-Benzyloxycarbonyl-5-aminopentyl 2,3-di-_O_-acetyl-4,6-di-_O_-benzyl-α-D-mannopyranosyl-(1→3)-2-_O_-acetyl-4,6-di-_O_-benzyl-α-D-mannopyranosyl-(1→2)-6-_O_-acetyl-3,4-di-_O_-benzyl-α-D-mannopyranoside (S10)

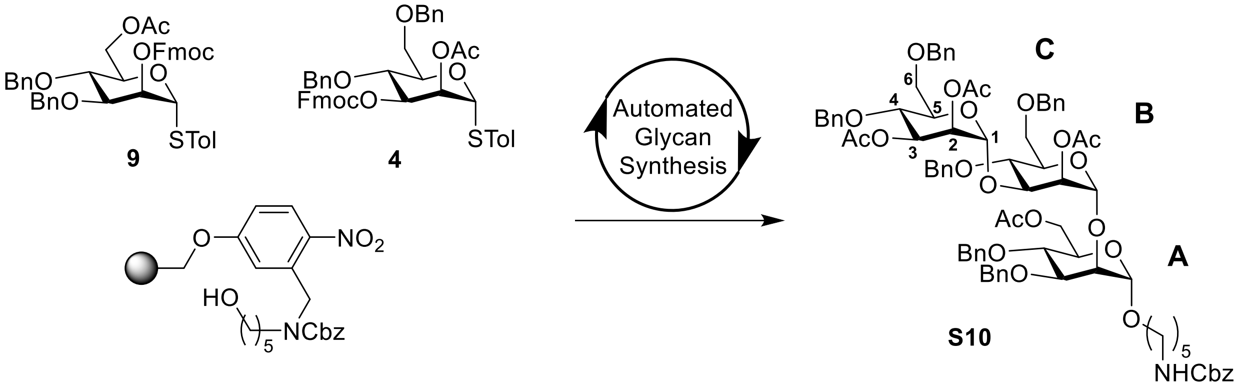

Module	Conditions
A: Resin Swelling	
B: Acid Wash<br>D: Thioglycoside Glycosylation Double<br>E: Acidic Capping<br>F: Fmoc Deprotection	2 × BB **9** (8 eq.), -20 °C for 300 s, 0 °C for 1200 s
B: Acid Wash<br>D: Thioglycoside Glycosylation Single<br>E: Acidic Capping<br>F: Fmoc Deprotection	BB **4** (8 eq.), -20 °C for 300 s, 0 °C for 1200 s
B: Acid Wash<br>D: Thioglycoside Glycosylation Single<br>E: Acidic Capping<br>F: Fmoc Deprotection	BB **4** (8 eq.), -20 °C for 300 s, 0 °C for 1200 s
E: Acidic Capping	

The synthesis was repeated three times on 12.5 µmol scale (based on resin) and the resulting material was combined to enable purification and characterisation. Cleavage from solid support (**Method A** in post-automation procedures) and column chromatography (SiO_2, CyH/EtOAc, 4/1 → 7/3) afforded a mixture (pale yellow syrup) in which the major species was **S10** and characterisation by NMR and MS was possible.

^{1}H NMR (599 MHz, $CDCl_3$) δ 7.40 – 7.12 (m, 35H, Ar), 5.37 – 5.32 (m, 3H, H-C2B, H-C2C, H-C3C), 5.14 (d, J = 1.8 Hz, 1H, H-C1B), 5.09 (s, 2H, CH_2 Cbz), 5.02 (d, J = 1.9 Hz, 1H, H-C1C), 4.92 (d, J = 1.9 Hz, 1H, H-C1A), 4.88 (d, J = 10.8 Hz, 1H, C*H₂*Ph), 4.77 (d, J = 10.6 Hz, 1H, CH_2Ph), 4.68 – 4.61 (m, 4H, CH_2Ph), 4.60 (d, J = 11.4 Hz, 1H, CH_2Ph), 4.56 (d, J = 10.8 Hz, 1H, CH_2Ph), 4.50 (d, J = 11.2 Hz, 1H, CH_2Ph), 4.48 – 4.45 (m, 2H, CH_2Ph), 4.35 – 4.28 (m, 2H, CH_2Ph, H-C6A), 4.21 – 4.16 (m, 2H, H-C6A, H-C3B), 4.08 (t, J = 9.7 Hz, 1H, H-C4C), 4.00 – 3.96 (m, 1H, H-C5B), 3.93 – 3.85 (m, 4H, H-C5C, H-C4B, H-C2A, H-C3A), 3.79 – 3.73 (m, 3H, H-C4A, H-C6B, H-C6C), 3.72 – 3.67 (m, 1H, H-C5A), 3.67 – 3.64 (m, 1H, H-C6B), 3.58 – 3.55 (m, 1H, H-C6C), 3.54 – 3.47 (m, 1H, OCH_2 linker), 3.27 – 3.10 (m, 3H, OCH_2 linker, NCH_2 linker), 2.17 (s, 3H, Ac), 2.08 (s, 3H, Ac), 1.94 (s, 3H, Ac), 1.88 (s, 3H, Ac), 1.53 – 1.42 (m, 4H, CH_2 linker), 1.29 –

1.24 (m, 2H, CH_2 linker) ppm; **^{13}C NMR** (151 MHz, $CDCl_3$) δ 171.2 (C=O Ac), 170.5 (C=O Ac), 170.1 (C=O Ac), 170.0 (C=O Ac), 156.5 (C=O Cbz), 138.47 (Ar), 138.46 (Ar), 138.24 (Ar), 138.20 (Ar), 138.17 (Ar), 137.9 (Ar), 136.8 (Ar), 128.64 (Ar), 128.56 (Ar), 128.53 (Ar), 128.49 (Ar), 128.46 (Ar), 128.45 (Ar), 128.42 (Ar), 128.40 (Ar), 128.38 (Ar), 128.27 (Ar), 128.25 (Ar), 128.2 (Ar), 128.0 (Ar), 127.93 (Ar), 127.91 (Ar), 127.81 (Ar), 127.77 (Ar), 127.74 (Ar), 127.73 (Ar), 127.72 (Ar), 127.68 (Ar), 127.67 (Ar), 127.6 (Ar), 99.9 (C1B), 99.4 (C1C), 98.5 (C1A), 79.2 (C3A), 77.6 (C3B), 76.9 (C2A), 75.5 (CH_2Ph), 75.4 (CH_2Ph), 74.8 (C4B), 74.6 (CH_2Ph), 74.2 (C4A), 73.5 (CH_2Ph), 73.4 (CH_2Ph), 72.6 (C4C), 72.4 (C5C), 72.0 (C2C), 71.9 (CH_2Ph), 71.8 (C5B, C3C), 70.3 (C2B), 69.8 (C5A), 69.0 (C6B), 68.2 (C6C), 67.7 (OCH_2 linker), 66.7 (CH_2 Cbz), 63.3 (C6A), 41.1 (NCH_2 linker), 29.8 (CH_2 linker), 29.1 (CH_2 linker), 23.5 (CH_2 linker), 21.2 (Ac), 20.99 (Ac), 20.96 (Ac), 20.9 (Ac) ppm; **MALDI-MS** m/z $[M+Na]^+$ calcd for $C_{81}H_{93}NO_{22}Na^+$ 1454.61; found 1454.50.

***N*-Benzyloxycarbonyl-5-aminopentyl 4,6-di-*O*-benzyl-α-D-mannopyranosyl-(1→3)-4,6-di-*O*-benzyl-α-D-mannopyranosyl-(1→2)-3,4-di-*O*-benzyl-α-D-mannopyranoside (18)**

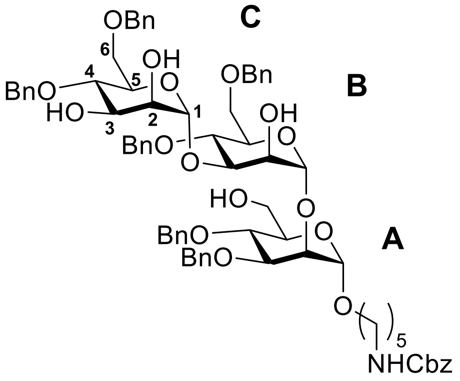

The impure **S10** was subjected to methanolysis (**Method B**) and partially purified with column chromatography (SiO_2, CyH/EtOAc, 4/1 → 1/1). HPLC SEC (**Method C**) afforded pure **18** (7.7 mg, 16% based on resin) as a colourless syrup.

^{1}H NMR (599 MHz, $CDCl_3$) δ 7.35 – 7.17 (m, 35H, Ar), 5.10 – 5.06 (m, 3H, CH_2 Cbz, H-C1B/C), 4.95 (d, *J* = 1.9 Hz, 1H, H-C1B/C), 4.88 (d, *J* = 10.8 Hz, 1H, C*H₂*Ph), 4.84 (d, *J* = 1.9 Hz, 1H, H-C1A), 4.71 – 4.67 (m, 2H, CH_2Ph), 4.66 – 4.64 (m, 2H, CH_2Ph), 4.61 (d, *J* = 10.9 Hz, 1H, CH_2Ph), 4.60 (d, *J* = 12.1 Hz, 1H, CH_2Ph), 4.54 (d, *J* = 11.3 Hz, 1H, CH_2Ph), 4.53 (d, *J* = 12.0 Hz, 1H, CH_2Ph), 4.50 (d, *J* = 12.0 Hz, 1H, CH_2Ph), 4.48 (d, *J* = 12.2 Hz, 1H, CH_2Ph), 4.45 (d, *J* = 11.0 Hz, 1H, CH_2Ph), 4.30 – 4.28 (m, 1H, H-C2B/C), 4.24 – 4.19 (m, 1H, H-C5B/C), 4.00 (dd, *J* = 9.2, 3.1 Hz, 1H, H-C3 B/C), 3.97 – 3.93 (m, 2H, H-C2A, H-C3B/C), 3.93 – 3.90 (m, 1H, H-C2B/C), 3.90 – 3.86 (m, 2H, H-C5B/C, H-C3A), 3.83 – 3.73 (m, 4H, H-C4A, H-C4B/C, H-C6B/C, H-C6A), 3.73 – 3.64 (m, 3H, H-C6B/C, H-C6A), 3.58 – 3.47 (m, 4H, H-C6B/C, H-C5A, H-C4B/C, OCH_2 linker), 3.19 – 3.11 (m, 3H, OCH_2 linker, NCH_2 linker), 1.50 – 1.39 (m, 4H, CH_2 linker), 1.28 – 1.23 (m, 2H, CH_2 linker) ppm; **^{13}C NMR** (151 MHz, $CDCl_3$) δ 156.5 (C=O Cbz), 138.50 (Ar), 138.48 (Ar), 138.4 (Ar), 138.3 (Ar), 138.0 (Ar), 137.3 (Ar), 136.8 (Ar), 128.8 (Ar), 128.7 (Ar), 128.61 (Ar), 128.59 (Ar), 128.58 (Ar), 128.55 (Ar), 128.4 (Ar), 128.3 (Ar), 128.22 (Ar), 128.19 (Ar), 128.11 (Ar), 128.09 (Ar), 128.06 (Ar), 128.0 (Ar), 127.94 (Ar), 127.90 (Ar), 127.82 (Ar), 127.77 (Ar), 127.7 (Ar), 102.1 (C1B/C), 100.5 (C1B/C), 98.9 (C1A), 82.1 (C3B/C), 79.6 (C3A), 76.5 (C4B/C), 75.5 (CH_2Ph), 75.4 (CH_2Ph), 75.2 (C3B/C), 74.9 (CH_2Ph), 74.8 (C4A), 74.3 (C4B/C), 73.7 (CH_2Ph), 73.6 (CH_2Ph), 72.2 (CH_2Ph), 72.1 (C5A, C2A), 72.0 (C5B/C), 71.6 (C2B/C), 71.5 (C5B/C), 69.7 (C6B/C), 69.4 (C2B/C), 69.3 (C6B/C), 67.6 (OCH_2 linker), 66.8

(CH_2 Cbz), 62.4 (C6A), 41.1 (NCH_2 linker), 29.8 (CH_2 linker), 29.2 (CH_2 linker), 23.5 (CH_2 linker) ppm; **MALDI-MS** m/z $[M+Na]^+$ calcd for $C_{73}H_{85}NO_{18}Na^+$ 1286.57; found 1286.63.

5-Aminopentyl α-D-mannopyranosyl-(1→3)-α-D-mannopyranosyl-(1→2)-α-D-mannopyranoside (24)

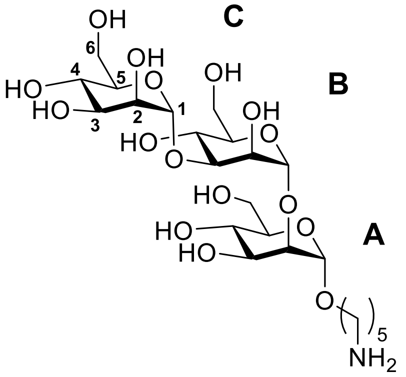

Partially protected trisaccharide **18** (7.7 mg, 6.09 µmol) was subjected to hydrogenolysis (**Method E**) to afford **24** (1.8 mg, 50%, 8% based on resin) as a colourless syrup.

^{1}H NMR (500 MHz, D_2O) δ 5.17 (s, 1H, H-C1), 5.12 (s, 1H, H-C1), 5.04 (s, 1H, H-C1), 4.26 (dd, J = 3.0, 1.8 Hz, 1H, H-C2), 4.10 (dd, J = 3.5, 1.7 Hz, 1H, H-C2), 4.02 – 3.62 (m, 17H, H-C2, H-C3 × 3, H-C4 × 3, H-C5 × 3, H-C6 × 6, OCH_2 linker), 3.61 – 3.54 (m, 1H, OCH_2 linker), 3.08 – 2.98 (m, 2H, NCH_2 linker), 1.79 – 1.61 (m, 4H, CH_2 linker), 1.56 – 1.41 (m, 2H, CH_2 linker) ppm; **^{13}C NMR** (126 MHz, D_2O) δ 102.2 (C1), 102.2 (C1), 98.1 (C1), 78.7 (C2/3/4/5), 77.8 (C2/3/4/5), 73.3 (C2/3/4/5), 72.8 (C2/3/4/5), 70.4 (C2/3/4/5), 70.0 (C2), 69.5 (C2), 67.5 (OCH_2 linker), 67.0 (C2/3/4/5), 66.9 (C2/3/4/5), 66.2 (C2/3/4/5), 61.1 (C6 × 3), 39.3 (NCH_2 linker), 27.8 (CH_2 linker), 26.5 (CH_2 linker), 22.4 (CH_2 linker) ppm; **HRMS** (ESI) m/z $[M+Na]^+$ calcd for $C_{23}H_{43}NO_{16}Na^+$ 612.2480; found 612.2476.

N-Benzyloxycarbonyl-5-aminopentyl 2,3-di-O-acetyl-4,6-di-O-benzyl-α-D-mannopyranosyl-(1→2)-6-O-acetyl-3,4-di-O-benzyl-α-D-mannopyranosyl-(1→3)-2-O-acetyl-4,6-di-O-benzyl-α-D-mannopyranoside (S11)

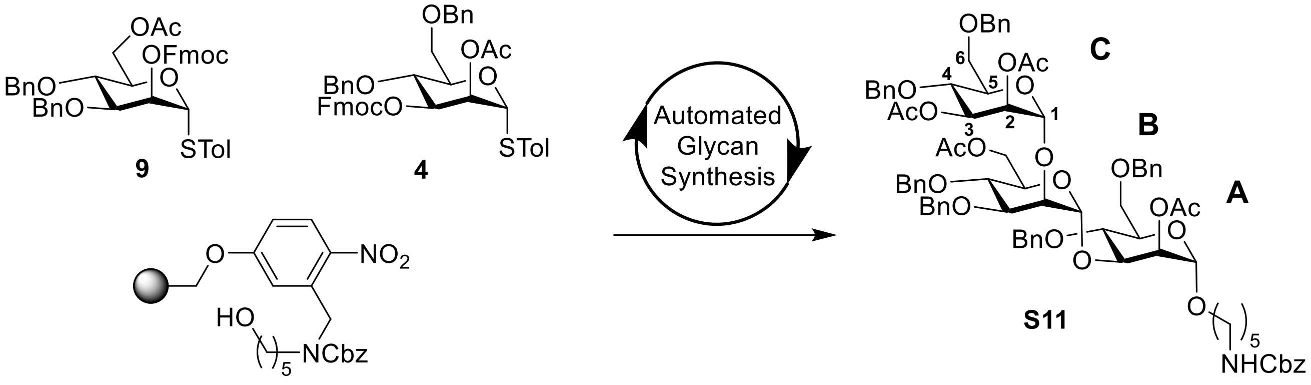

Module	Conditions
A: Resin Swelling	
B: Acid Wash<br>D: Thioglycoside Glycosylation Single<br>E: Acidic Capping<br>F: Fmoc Deprotection	BB **4** (8 eq.), -20 °C for 300 s, 0 °C for 1200 s
B: Acid Wash<br>D: Thioglycoside Glycosylation Single<br>E: Acidic Capping<br>F: Fmoc Deprotection	BB **9** (8 eq.), -20 °C for 300 s, 0 °C for 1200 s
B: Acid Wash<br>D: Thioglycoside Glycosylation Single<br>E: Acidic Capping<br>F: Fmoc Deprotection	BB **4** (8 eq.), -20 °C for 300 s, 0 °C for 1200 s
E: Acidic Capping	

The synthesis was repeated three times on 12.5 µmol scale (based on resin) and the resulting material was combined to enable purification and characterisation. Cleavage from solid support (**Method A**) and column chromatography (SiO_2, CyH/EtOAc, 4/1 → 7/3) afforded a mixture (pale yellow syrup) in which the major species was **S11** and characterisation by NMR and MS was possible.

^{1}H NMR (599 MHz, $CDCl_3$) δ 7.40 – 7.06 (m, 35H, Ar), 5.42 – 5.33 (m, 2H, H-C2C, H-C3C), 5.21 (d, *J* = 1.5 Hz, 1H, H-C1B), 5.10 (dd, *J* = 3.2, 1.8 Hz, 1H, H-C2A), 5.07 (s, 2H, CH_2 Cbz), 4.91 – 4.78 (m, 3H, CH_2Ph, H-C1A, H-C1C), 4.69 – 4.34 (m, 11H, CH_2Ph), 4.24 (dd, *J* = 11.8, 3.7 Hz, 1H, H-C6B), 4.18 (dd, *J* = 12.0, 2.1 Hz, 1H, H-C6B), 4.10 (dd, *J* = 9.8, 3.3 Hz, 1H, H-C3A), 4.00 – 3.75 (m, 6H, H-C5C, H-C4C, H-C4B, H-C2B, H-C3B, H-C4C), 3.75 – 3.58 (m, 6H, OCH_2 linker, H-C6C, H-C6A, H-C5B, H-C5A), 3.45 (d, *J* = 10.9 Hz, 1H, H-C6C), 3.39 – 3.32 (m, 1H, OCH_2 linker), 3.21–3.05 (s, 2H, NCH_2 linker), 2.09 (s, 3H, Ac), 2.05 (s, 3H, Ac), 1.96 (s, 3H, Ac), 1.96 (s, 3H, Ac), 1.59 – 1.42 (m, 4H, CH_2 linker), 1.34 – 1.26 (m, 2H, CH_2 linker) ppm; **^{13}C NMR** (151 MHz, $CDCl_3$) δ 171.4 (C=O Ac), 170.4 (C=O Ac), 170.0 (C=O Ac), 169.6 (C=O Ac), 156.5 (C=O Cbz), 138.4 (Ar), 138.34 (Ar), 138.32 (Ar), 138.31 (Ar), 138.26 (Ar), 138.2 (Ar), 136.9 (Ar), 128.64 (Ar), 128.62 (Ar), 128.61 (Ar), 128.54 (Ar), 128.52 (Ar), 128.47 (Ar), 128.46 (Ar), 128.44 (Ar), 128.40 (Ar), 128.1 (Ar), 128.02 (Ar), 128.01 (Ar), 127.9 (Ar), 127.8 (Ar), 127.7 (Ar), 127.5 (Ar), 127.4 (Ar), 101.2 (C1B), 99.5 (C1C), 97.2 (C1A), 79.6 (C3B), 79.0 (C3A), 76.4 (C2B), 75.3 (Ch_2Ph), 75.2 (Ch_2Ph), 74.9

(Ch_2Ph), 74.6 (C4A), 73.6 (Ch_2Ph), 73.5 (C4B), 73.4 (Ch_2Ph), 73.0 (C4C), 72.4 (C2A), 72.2 (Ch_2Ph), 71.9 (C5C), 71.8 (C3C), 71.5 (C5A), 71.1 (C5B), 70.2 (C2C), 69.0 (C6A), 68.3 (C6C), 68.0 (OCH_2 linker), 66.7 (CH_2 Cbz), 62.7 (C6B), 41.1 (NCH_2 linker), 29.8 (CH_2 linker), 29.1 (CH_2 linker), 23.4 (CH_2 linker), 21.2 (Ac), 21.1 (Ac), 21.0 (Ac), 20.8 (Ac) ppm; **MALDI-MS** m/z $[M+Na]^+$ calcd for $C_{81}H_{93}NO_{22}Na^+$ 1454.61; found 1454.74.

***N*-Benzyloxycarbonyl-5-aminopentyl 4,6-di-*O*-benzyl-α-D-mannopyranosyl-(1→2)-3,4-di-*O*-benzyl-α-D-mannopyranosyl-(1→3)-4,6-di-*O*-benzyl-α-D-mannopyranoside (19)**

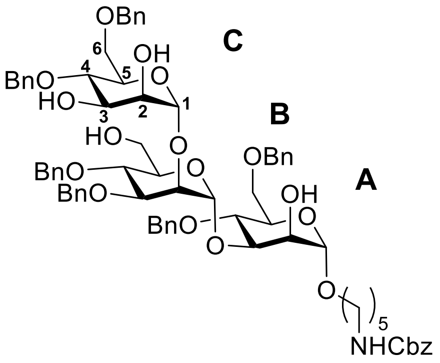

The impure **S11** was subjected to methanolysis (**Method B**) and partially purified with column chromatography (SiO_2, CyH/EtOAc, 4/1 → 2/1). HPLC SEC (**Method C**) afforded pure **19** (3.2 mg, 7% based on resin) as a colourless syrup.

^{1}H NMR (599 MHz, $CDCl_3$) δ 7.35 – 7.27 (m, 25H, Ar), 7.25 – 7.17 (m, 10H, Ar), 5.21 (s, 0.55H, H-C1 rotamer), 5.09 (s, 0.45H, H-C1 rotamer), 5.07 (s, 2H, CH_2 Cbz), 5.02 (s, 0.45H, H-C1 rotamer), 4.97 (s, 0.55H, H-C1 rotamer), 4.88 – 4.83 (m, 1H, CH_2Ph), 4.77 – 4.75 (m, 1H, H-C1), 4.65 – 4.50 (m, 8H, CH_2Ph), 4.48 (d, J = 12.1 Hz, 1H, CH_2Ph), 4.45 (d, J = 12.2 Hz, 1H, CH_2Ph), 4.39 (d, J = 11.3 Hz, 1H, CH_2Ph), 4.07 – 4.04 (m, 1H, H-C2), 4.01 – 3.92 (m, 5H, H-C2, H-C2/3/4/5 × 4), 3.90 (dd, J = 9.1, 2.9 Hz, 1H, H-C3), 3.88 – 3.82 (m, 2H, H-C6, H-C2/3/4/5), 3.79 – 3.74 (m, 1H, H-C2/3/4/5), 3.72 – 3.60 (m, 8H, H-C6 × 4, OCH_2 linker, H-C2/3/4/5 × 3), 3.58 (s, 1H, H-C6), 3.39 – 3.31 (m, 1H OCH_2 linker), 3.16 – 3.07 (m, 2H NCH_2 linker), 1.53 – 1.46 (m, 2H CH_2 linker), 1.45 – 1.39 (m, 2H CH_2 linker), 1.29 – 1.26 (m, 2H, CH_2 linker) ppm; **^{13}C NMR** (151 MHz, $CDCl_3$) δ 138.5 (Ar), 138.4 (Ar), 138.27 (Ar), 138.26 (Ar), 138.23 (Ar), 138.20 (Ar), 138.0 (Ar), 128.58 (Ar), 128.55 (Ar), 128.49 (Ar), 128.47 (Ar), 128.44 (Ar), 128.43 (Ar), 128.37 (Ar), 128.32 (Ar), 128.31 (Ar), 128.1 (Ar), 128.04 (Ar), 127.99 (Ar), 127.95 (Ar), 127.94 (Ar), 127.90 (Ar), 127.86 (Ar), 127.82 (Ar), 127.81 (Ar), 127.78 (Ar), 127.77 (Ar), 127.72 (Ar), 127.66 (Ar), 127.6. (Ar), 101.4 (C1), 100.3 (C1), 99.9 (C1), 81.6 (C2/3/4/5), 79.7 (C3), 76.1(C2/3/4/5), 75.99(C2/3/4/5), 75.4 (CH_2Ph), 75.3 (CH_2Ph), 75.0(C2/3/4/5), 74.9 (CH_2Ph), 74.2(C2/3/4/5), 73.6 (CH_2Ph), 73.5 (CH_2Ph), 73.1(C2/3/4/5), 72.4 (CH_2Ph), 71.72(C2/3/4/5), 71.70(C2/3/4/5), 71.4(C2/3/4/5), 71.1 (C2), 70.3 (C2), 69.1 (C6), 68.9 (C6), 67.8 (OCH_2 linker), 66.7 (CH_2 Cbz), 62.4 (C6), 41.1 (NCH_2 linker), 29.8(CH_2 linker) , 29.0 (CH_2 linker), 23.5 (CH_2 linker) ppm; **MALDI-MS** m/z $[M+Na]^+$ calcd for $C_{73}H_{85}NO_{18}Na^+$ 1286.57; found 1286.23.

5-Aminopentyl α-D-mannopyranosyl-(1→2)-α-D-mannopyranosyl-(1→3)-α-D-mannopyranoside (25)

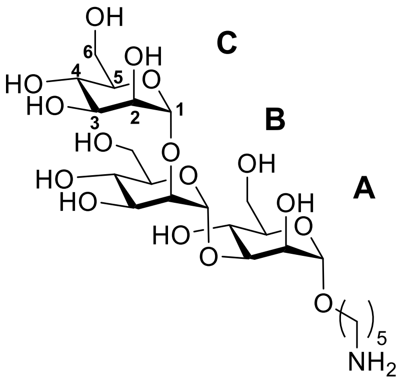

Partially protected trisaccharide **19** (3.0 mg, 2.37 µmol) was subjected to hydrogenolysis (**Method E**) to afford **25** (1.0 mg, 72%, 5% based on resin) as a colourless syrup.

^{1}H NMR (599 MHz, D_2O) δ 5.27 – 5.15 (m, 1H, H-C1 conformers), 4.99 – 4.89 (m, 1H, H-C1 conformers), 4.72 (s, 1H, H-C1), 3.98 (s, 1H, H-C2), 3.97 – 3.94 (m, 2H, H-C2), 3.87 (dd, J = 9.6, 3.3 Hz, 1H, H-C3), 3.85 – 3.69 (m, 5H, H-C3 × 2, H-C6 × 3), 3.68 – 3.47 (m, 10H, H-C4 × 3, H-C5 × 3, H-C6 × 3, OCH_2 linker), 3.46 – 3.40 (m, 1H, OCH_2 linker), 2.89 (s, 2H, NCH_2 linker), 1.64 – 1.48 (m, 4H, CH_2 linker), 1.34 (s, 2H, CH_2 linker) ppm; **^{13}C NMR** (151 MHz, D_2O extracted from the gHSQC spectrum) δ 102.3 (C1 conformers), 100.7 (C1 two conformers), 99.5 (C1), 98.0 (C1 conformers), 78.9 (C3), 78.42 (C2), 78.37 (C3), 73.2 (C4/5), 72.9 (C4/5), 70.3 (C3), 70.0 (C3), 69.9 (C3 conformers), 69.9 (C2), 69.6 (C2), 67.4 (OCH_2 linker), 66.9 (C4/5), 66.7 (C4/5), 66.1 (C4/5), 62.5 (C6), 61.1 (C6), 60.9 (C6), 39.3 (NCH_2 linker), 27.9 (CH_2 linker), 26.5 (CH_2 linker), 22.4 (CH_2 linker) ccoppm; **HRMS** (ESI) m/z $[M+Na]^+$ calcd for $C_{23}H_{43}NO_{16}Na^+$ 612.2480; found 612.2473.

_N_-Benzyloxycarbonyl-5-aminopentyl 2,6-di-_O_-acetyl-3,4-di-_O_-benzyl-α-D-mannopyranosyl-(1→3)-2-_O_-acetyl-4,6-di-_O_-benzyl-α-D-mannopyranosyl-(1→3)-2-_O_-acetyl-4,6-di-_O_-benzyl-α-D-mannopyranoside (S12)

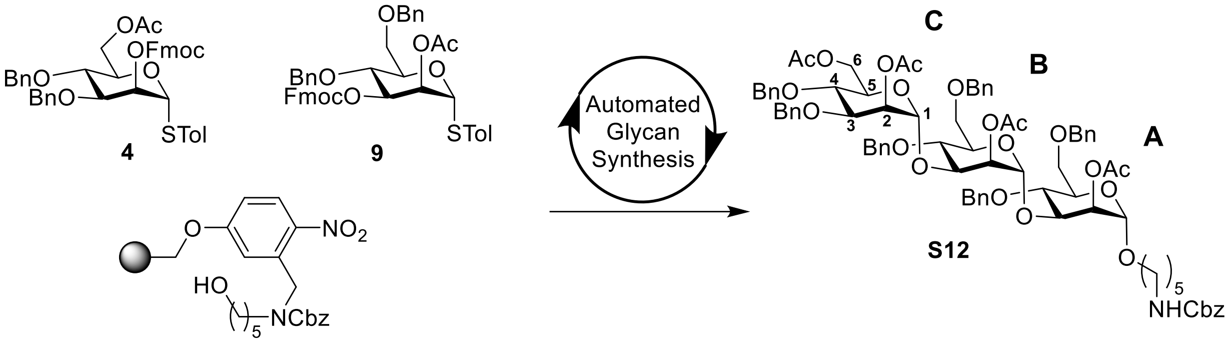

Module	Conditions
A: Resin Swelling	
B: Acid Wash<br>D: Thioglycoside Glycosylation Single<br>E: Acidic Capping<br>F: Fmoc Deprotection	BB **4** (8 eq.), -20 °C for 300 s, 0 °C for 1200 s
B: Acid Wash<br>D: Thioglycoside Glycosylation Single<br>E: Acidic Capping<br>F: Fmoc Deprotection	BB **4** (8 eq.), -20 °C for 300 s, 0 °C for 1200 s
B: Acid Wash<br>D: Thioglycoside Glycosylation Double<br>E: Acidic Capping<br>F: Fmoc Deprotection	2 × BB **9** (8 eq.), -20 °C for 300 s, 0 °C for 1200 s
E: Acidic Capping	

The synthesis was repeated three times on 12.5 µmol scale (based on resin) and the resulting material was combined to enable purification and characterisation. Cleavage from solid support (**Method A**) and column chromatography (SiO_2, CyH/EtOAc, 4/1 → 7/3) afforded a mixture (pale yellow syrup) in which the major species was **S12**, however overlapping peaks and weak signal meant characterisation by NMR was not possible.

MALDI-MS m/z $[M+Na]^+$ calcd for $C_{81}H_{93}NO_{22}Na^+$ 1454.61; found 1454.62.

***N*-Benzyloxycarbonyl-5-aminopentyl 3,4-di-*O*-benzyl-α-D-mannopyranosyl-(1→3)-4,6-di-*O*-benzyl-α-D-mannopyranosyl-(1→3)-4,6-di-*O*-benzyl-α-D-mannopyranoside (20)**

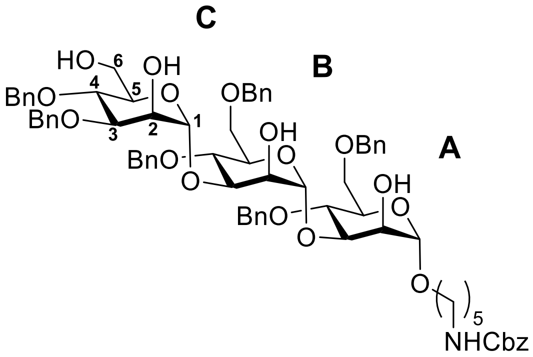

The impure **S12** was subjected to methanolysis (**Method B**) and partially purified with column chromatography (SiO_2, CyH/EtOAc, 4/1 → 1/1). HPLC SEC (**Method C**) afforded pure **20** (6.2 mg, 13% based on resin) as a colourless syrup.

Due to low sample amounts, certain resonances were not visible in the ^{13}C NMR spectrum.

^{1}H NMR (599 MHz, $CDCl_3$) δ 7.37 – 7.14 (m, 35H, Ar), 5.09 – 5.05 (m, 3H, CH_2 Cbz, H-C1C), 5.04 – 5.01 (m, 1H, H-C1B), 4.85 (d, *J* = 11.0 Hz, 1H, C*H*$_2$Ph), 4.75 – 4.47 (m, 10H, H-C1A, C*H*$_2$Ph), 4.44 (d, *J* = 10.9 Hz, 1H, C*H*$_2$Ph), 4.40 (d, *J* = 11.1 Hz, 1H, C*H*$_2$Ph), 4.26 – 4.21 (m, 1H, H-C5B), 4.12 – 4.09 (m, 2H, H-C2A, H-C2B), 4.02 (dd, *J* = 9.0, 3.1 Hz, 1H, H-C3B), 3.97 – 3.93 (m, 2H, H-C2C, H-C3A), 3.93 – 3.88 (m, 1H, H-C5C), 3.87 – 3.83 (m, 1H, H-C3C), 3.80 (t, *J* = 9.4 Hz, 1H, H-C4A), 3.75 – 3.57 (m, 9H, H-C4B, H-C4C, H-C5A, H-C6A, H-C6B, H-C6C, OCH_2 linker), 3.53 (dd, *J* = 10.0, 7.7 Hz, 1H, H-C6B), 3.35 – 3.30 (m, 1H, OCH_2 linker), 3.18 – 3.12 (m, 2H, NCH_2 linker), 1.56 – 1.50 (m, 2H, CH_2 linker), 1.50 – 1.43 (m, 2H, CH_2 linker), 1.36 – 1.26 (m, 2H, CH_2 linker) ppm; **^{13}C NMR** (151 MHz, $CDCl_3$) δ 138.45 (Ar), 138.40 (Ar), 138.2 (Ar), 138.0 (Ar), 137.8 (Ar), 137.6 (Ar), 136.9 (Ar), 128.74 (Ar), 128.71 (Ar), 128.69 (Ar), 128.65 (Ar), 128.64 (Ar), 128.59 (Ar), 128.57 (Ar), 128.5 (Ar), 128.3 (Ar), 128.24 (Ar), 128.19 (Ar), 128.15 (Ar), 128.09 (Ar), 128.06 (Ar), 128.05 (Ar), 128.01 (Ar), 127.97 (Ar), 127.96 (Ar), 127.9 (Ar), 127.7 (Ar), 101.2 (C1B), 101.0 (C1C), 100.3 (C1A), 82.6 (C3C), 81.3 (C3B), 79.9 (C3C), 75.4 (CH_2Ph), 75.34 (CH_2Ph), 75.27 (CH_2Ph), 74.7 (C4B), 74.3 (C4C), 74.0 (C4A) , 73.8 (CH_2Ph), 73.6 (CH_2Ph), 72.6 (C5C), 72.4 (CH_2Ph), 71.8 (C5B), 71.5 (C5A), 70.8 (C2B), 69.59 (C2A), 69.55 (C6B), 69.3 (C6A), 69.0 (C2C), 67.6 (OCH_2 linker), 66.7 (CH_2 Cbz), 62.2 (C6C), 41.1 (NCH_2 linker), 29.9 (CH_2 linker), 29.2 (CH_2 linker), 23.6 (CH_2 linker) ppm; **MALDI-MS** m/z $[M+Na]^+$ calcd for $C_{73}H_{85}NO_{18}Na^+$ 1286.57; found 1286.56.

5-Aminopentyl α-D-mannopyranosyl-(1→3)-α-D-mannopyranosyl-(1→3)-α-D-mannopyranoside (26)

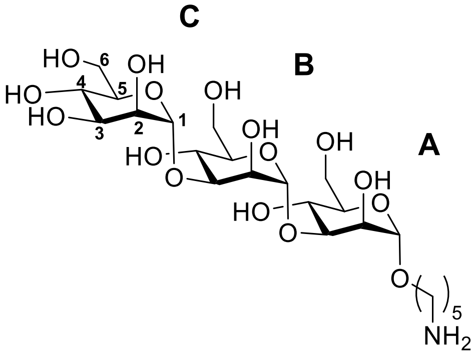

Partially protected trisaccharide **20** (2.5 mg, 2.00 µmol) was subjected to hydrogenolysis (**Method E**) to afford **26** (0.9 mg, 76%, 10% based on resin) as a colourless syrup.

^{1}**H NMR** (599 MHz, $CDCl_3$) δ 5.40 – 5.06 (m, 2H, H-C1 × 2 rotamers), 4.87 – 4.86 (m, 1H, H-C1), 4.27 – 3.55 (m, 20H, H-C2 × 3, H-C3 × 3, H-C4 × 3, H-C5 × 3, H-C6 × 6, OCH_2 linker × 2), 3.06 – 3.01 (m, 2H, NCH_2 linker), 1.75 – 1.64 (m, 4H, CH_2 linker), 1.55 – 1.42 (m, 2H, CH_2 linker). ppm; **HRMS** (ESI) m/z $[M+Na]^+$ calcd for $C_{23}H_{43}NO_{16}Na^+$ 612.2480; found 612.2475.

_N_-Benzyloxycarbonyl-5-aminopentyl 3,6-di-_O_-acetyl-4-_O_-benzyl-2-deoxy-2-fluoro-α-D-mannopyranosyl-(1→3)-6-_O_-acetyl-4-_O_-benzyl-2-deoxy-2-fluoro-α-D-mannopyranosyl-(1→2)-6-_O_-acetyl-3,4-di-_O_-benzyl-α-D-mannopyranoside (S13)

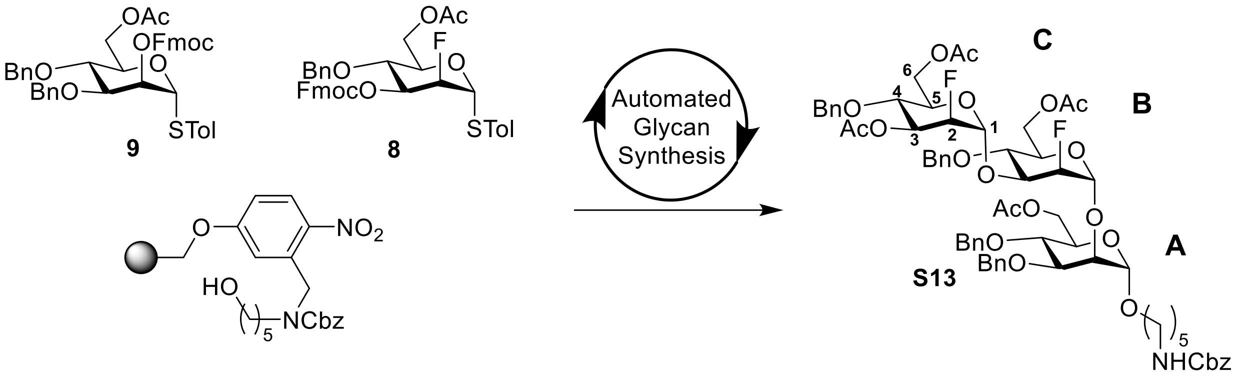

Module	Conditions
A: Resin Swelling	
B: Acid Wash<br>D: Thioglycoside Glycosylation Double<br>E: Acidic Capping<br>F: Fmoc Deprotection	2 × BB **9** (8 eq.), -20 °C for 300 s, 0 °C for 1200 s
B: Acid Wash<br>D: Thioglycoside Glycosylation Double<br>E: Acidic Capping<br>F: Fmoc Deprotection	2 × BB**6** (5 eq.), -10 °C for 300 s, 10 °C for 1800 s
B: Acid Wash<br>D: Thioglycoside Glycosylation Double<br>E: Acidic Capping<br>F: Fmoc Deprotection	2 × BB **8** (5 eq.), -10 °C for 300 s, 10 °C for 1800 s
E: Acidic Capping	

The synthesis was repeated three times on 12.5 µmol scale (based on resin) and the resulting material was combined to enable purification and characterisation. Cleavage from solid support (**Method A** in post-automation procedures) and column chromatography (SiO_2, CyH/EtOAc, 4/1 → 7/3) afforded a mixture (pale yellow syrup) in which the major species was **S13** and characterisation by NMR and MS was possible.

^{1}H NMR (599 MHz, $CDCl_3$) δ 7.38 – 7.27 (m, 25H, Ar), 5.36 (ddd, *J* = 29.3, 9.7, 2.4 Hz, 1H, H-C3C), 5.20 (dd, *J* = 7.0, 2.1 Hz, 1H, H-C1C), 5.11 – 5.07 (m, 3H, H-C1B, CH_2 Cbz), 4.96 (dt, *J* = 49.1, 2.1, 2.1 Hz, 1H, H-C2B), 4.84 (d, *J* = 10.8 Hz, 1H, C*H₂*Ph), 4.78 (d, *J* = 10.8 Hz, 1H, C*H₂*Ph), 4.77 (d, *J* = 1.8 Hz, 1H, H-C1A), 4.76 (dt, *J* = 50.5, 2.2, 2.2 Hz, 1H, H-C2C), 4.72 (d, *J* = 10.8 Hz, 1H, C*H₂*Ph), 4.69 (d, *J* = 11.7 Hz, 1H, C*H₂*Ph), 4.63 (d, *J* = 11.6 Hz, 1H, C*H₂*Ph), 4.62 (d, *J* = 11.3 Hz, 1H, C*H₂*Ph), 4.58 (d, *J* = 10.8 Hz, 1H, C*H₂*Ph), 4.57 (d, *J* = 10.9 Hz, 1H, C*H₂*Ph), 4.35 – 4.28 (m, 2H, H-C6C), 4.28 – 4.23 (m, 2H, H-C6B. H-C6A), 4.24 – 4.20 (m, 2H, H-C6B, H-C5C), 4.17 (dd, *J* = 11.9, 4.2 Hz, 1H, H-C6A), 4.01 (ddd, *J* = 29.7, 9.6, 2.6 Hz, 1H, H-C3B), 3.95 (t, *J* = 9.9, 9.9 Hz, 1H, H-C4C), 3.93 – 3.87 (m, 3H, H-C5B, H-C2A, H-C3A), 3.79 (t, *J* = 9.8 Hz, 1H, H-C4B), 3.74 – 3.65 (m, 2H, H-C5A, H-C4A), 3.62 – 3.57 (m, 1H, OCH_2 linker), 3.36 – 3.31 (m, 1H, OCH_2 linker), 3.21 – 3.15 (m, 2H, NCH_2 linker), 2.10 (s, 3H, Ac), 2.05 (s, 3H, Ac), 1.93 (s, 3H, Ac), 1.92 (s, 3H, Ac). 1.58 – 1.47 (m, 4H, CH_2 linker), 1.37 – 1.28 (m, 2H, CH_2 linker) ppm; **^{13}C NMR** (101 MHz, $CDCl_3$)

δ 170.9 (C=O Ac), 170.8 (C=O Ac), 170.6 (C=O Ac), 170.1 (C=O Ac), 156.5 (C=O Cbz), 137.99 (Ar), 137.97 (Ar), 137.64 (Ar), 137.60 (Ar), 136.74 (Ar), 128.70 (Ar), 128.69 (Ar), 128.65 (Ar), 128.64 (Ar), 128.61 (Ar), 128.5 (Ar), 128.4 (Ar), 128.3 (Ar), 128.23 (Ar), 128.22 (Ar), 128.15 (Ar), 128.1 (Ar), 128.04 (Ar), 128.01 (Ar), 127.9 (Ar), 99.4 (d, *J* = 29.1 Hz, C1C), 98.7 (d, *J* = 28.8 Hz, C1B), 98.6 (C1A), 88.2 (d, *J* = 178.7 Hz, C2B), 87.4 (d, *J* = 178.3 Hz, C2C), 81.4 (d, *J* = 16.9 Hz, C3B), 79.7 (C3A), 75.8 (CH_2Ph, C2A), 75.3 (CH_2Ph), 74.0 (C4A), 73.2 (C4B), 72.9 (d, *J* = 16.9 Hz, C3C), 72.7 (CH_2Ph), 72.5 (C4C), 70.6 (d, *J* = 2.4 Hz, C5C), 70.3 (C5B), 69.9 (C5A), 67.8 (OCH_2, linker), 66.8 (CH_2, Cbz), 63.3 (C6A), 62.9 (C6B), 62.8 (C6C), 41.1 (NCH_2 linker), 29.9 (CH_2 linker), 29.2 (CH_2 linker), 23.5 (CH_2 linker), 21.1 (Ac), 21.0 (Ac), 20.9 (Ac), 20.7 (Ac) ppm; **^{19}F NMR** (564 MHz, $CDCl_3$) δ -202.42 (ddd, J_{FH} = 49.2, 29.5, 7.2 Hz, F-C2B), -203.73 (ddd, J_{FH} = 50.3, 29.3, 7.0 Hz, F-C2C) ppm; **MALDI-MS** m/z $[M+Na]^+$ calcd for $C_{67}H_{79}F_2NO_{20}Na^+$ 1278.51; found 1278.48.

_N_-Benzyloxycarbonyl-5-aminopentyl 4-_O_-benzyl-2-deoxy-2-fluoro-α-D-mannopyranosyl-(1→3)-4-_O_-benzyl-2-deoxy-2-fluoro-α-D-mannopyranosyl-(1→2)-3,4-di-_O_-benzyl-α-D-mannopyranoside (21)

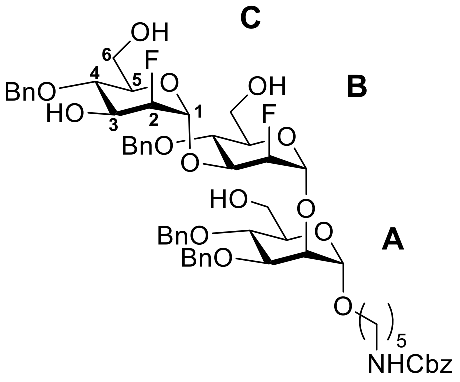

The impure **S13** was subjected to methanolysis (**Method B**) and partially purified with column chromatography (SiO_2, CyH/EtOAc, 4/1 → 1/1). HPLC SEC (**Method C**) afforded pure **21** (8.1 mg, 20% based on resin) as a colourless syrup.

^{1}H NMR (599 MHz, $CDCl_3$) δ 7.38 – 7.27 (m, 25H, Ar), 5.32 – 5.29 (m, 1H, H-C1B), 5.28 (dd, *J* = 8.2, 1.7 Hz, 1H, H-C1C), 5.08 (s, 2H, CH_2 CBz), 4.89 (dt, *J* = 50.0, 2.2 Hz, 1H, H-C2B), 4.87 (d, *J* = 10.8 Hz, 1H, C*H₂*Ph), 4.79 (d, *J* = 11.2 Hz, 1H, C*H₂*Ph), 4.73 (d, *J* = 2.0 Hz, 1H, H-C1A), 4.71 – 4.63 (m, 6H, C*H₂*Ph), 4.60 (dt, *J* = 49.6, 1.9 Hz, 1H, H-C2C), 4.10 (ddd, *J* = 30.4, 9.7, 2.4 Hz, 1H, H-C3B), 4.04 (ddd, *J* = 30.1, 9.4, 2.8 Hz, 1H, H-C3C), 3.98 – 3.96 (m, 1H, H-C2A), 3.92 – 3.89 (m, 2H, H-C3A, H-C4A), 3.89 – 3.82 (m, 4H, H-C4B, H-C5C, H-C6C, H-C6B), 3.78 – 3.76 (m, 2H, H-C6A), 3.74 – 3.70 (m, 3H, H-C5B, H-C6B, H-C6C), 3.62 (t, *J* = 9.6 Hz, 1H, H-C4C), 3.61 – 3.58 (m, 1H, OCH_2 linker), 3.57 – 3.53 (m, 1H, H-C5A), 3.37 – 3.30 (m, 1H, OCH_2 linker), 3.21 – 3.13 (m, 2H, NCH_2 linker), 1.58 – 1.46 (m, 4H, CH_2 linker), 1.36 – 1.29 (m, 2H, CH_2 linker) ppm; **^{13}C NMR** (151 MHz, $CDCl_3$) δ 156.6 (C=O Cbz), 138.5 (Ar), 138.1 (Ar), 137.9 (Ar), 137.5 (Ar), 136.7 (Ar), 128.9 (Ar), 128.80 (Ar), 128.68 (Ar), 128.67 (Ar), 128.6 (Ar), 128.4 (Ar), 128.30 (Ar), 128.27 (Ar), 128.25 (Ar), 128.24 (Ar), 128.21 (Ar), 128.0 (Ar), 127.9 (Ar), 127.8 (Ar), 99.42 (d, *J* = 30.6 Hz, C1C), 98.8 (C1A), 97.80 (d, *J* = 29.8 Hz, C1B), 89.80 (d, J = 174.3 Hz, C2C), 89.02 (d, *J* = 178.1 Hz, C2B), 80.0 (C3A), 77.1 (d, J = 16.7 Hz, C3B), 76.2 (C4C), 75.8 (CH_2Ph), 75.5 (CH_2Ph), 75.4 (CH_2Ph), 74.5 (C4B), 74.3 (C4A), 73.8 (C2A), 73.2 (C5B), 73.1 (C5C), 72.8 (CH_2Ph), 72.4 (C5A), 71.1 (d, *J* = 17.3 Hz, C3C), 67.7 (OCH_2 linker), 66.8 (CH_2 Cbz), 62.1 (C6C), 61.9 (C6B), 61.7 (C6A), 41.1 (NCH_2 linker), 29.9 (CH_2, linker), 29.0 (CH_2, linker), 23.4 (CH_2, linker) ppm; **^{19}F NMR** (564 MHz, $CDCl_3$) δ -203.36 (ddd, *J* = 49.9, 30.3, 6.9 Hz, F-C2B), -205.26 (ddd, *J* = 49.6, 30.2, 8.5 Hz, F-C2C) ppm; **MALDI-MS** m/z $[M+Na]^+$ calcd for $C_{59}H_{71}F_2NO_{16}Na^+$ 1110.46; found 1110.48.

5-Aminopentyl 2-deoxy-2-fluoro-α-D-mannopyranosyl-(1→3)-2-deoxy-2-fluoro-α-D-mannopyranosyl-(1→2)-α-D-mannopyranoside (27)

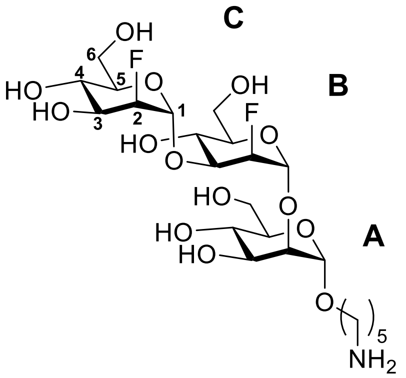

Partially protected trisaccharide **21** (8.1 mg, 7.4 µmol) was subjected to hydrogenolysis (**Method D**) to afford **27** (2.0 mg, 46%, 9% based on resin) as a colourless syrup.

Multiple peaks due to conformational isomers were seen in the ^{19}F-NMR spectrum which were confirmed to show dynamic behaviour with varying temperature. Only the major conformer is reported. Low sample amounts led to the ^{13}C {^{1}H,^{19}F} NMR spectrum being reported as opposed to the coupled spectrum.

^{1}H NMR (599 MHz, D_2O) δ 5.41 (d, *J* = 8.0 Hz, 1H, H-C1C), 5.29 (d, *J* = 7.7 Hz, 1H, H-C1B), 5.12 (dt, *J* = 49.4, 2.1 Hz, 1H, H-C2B), 5.11 (d, *J* = 1.8 Hz, 1H, H-C1A), 4.91 (dt, *J* = 49.2, 2.1 Hz, 1H, H-C2C), 4.09 (ddd, *J* = 30.9, 9.5, 2.6 Hz, 1H, H-C3B), 4.03 (dd, *J* = 3.2, 1.8 Hz, 1H, H-C2A), 4.01 – 3.71 (m, 13H, H-C6A, H-C6B, H-C6C, H-C5B, H-C5C, H-C4B, H-C4C, H-C3A, H-C3C, OCH_2 linker), 3.70 (t, *J* = 9.8 Hz, 1H, H-C4A), 3.66 – 3.62 (m, 1H, H-C5A), 3.61 – 3.54 (m, 1H, OCH_2 linker), 3.05 – 3.00 (m, 2H, NCH_2 linker), 1.76 – 1.64 (m, 4H, CH_2 linker), 1.51 – 1.44 (m, 2H, CH_2 linker) ppm; **^{13}C {^{1}H,^{19}F} NMR** (151 MHz, D_2O) δ 104.0 (C1B, C1C), 102.9 (C1A), 94.3 (C2C), 93.4 (C2B), 84.0 (C2A), 82.7 (C3B), 78.2 (C5C), 78.1 (C5B), 77.7 (C5A), 75.1 (C3A), 74.4 (C3C), 72.5 (OCH_2, linker), 71.8 (C4A), 71.6 (C4C), 70.7 (C4B), 65.81 (C6A), 65.53 (C6C), 65.45 (C6B), 44.3 (CH_2 linker), 32.9 (CH_2 linker), 31.4 (CH_2 linker), 27.3 (CH_2 linker) ppm; **^{19}F NMR** (564 MHz, D_2O) δ -202.93 (ddd, *J* = 49.1, 30.7, 7.4 Hz, F-C2B), -204.81 (ddd, *J* = 49.2, 31.7, 7.6 Hz, F-C2C) ppm; **HRMS** (ESI) m/z $[M+H]^+$ calcd for $C_{23}H_{41}F_2NO_{14}H^+$ 594.2568; found 594.2566.

N-Benzyloxycarbonyl-5-aminopentyl 3,6-di-O-acetyl-4-O-benzyl-2-deoxy-2-fluoro-α-D-mannopyranosyl-(1→2)-6-O-acetyl-3,4-di-O-benzyl-α-D-mannopyranosyl-(1→3)-6-O-acetyl-4-O-benzyl-2-deoxy-2-fluoro-α-D-mannopyranoside (S14)

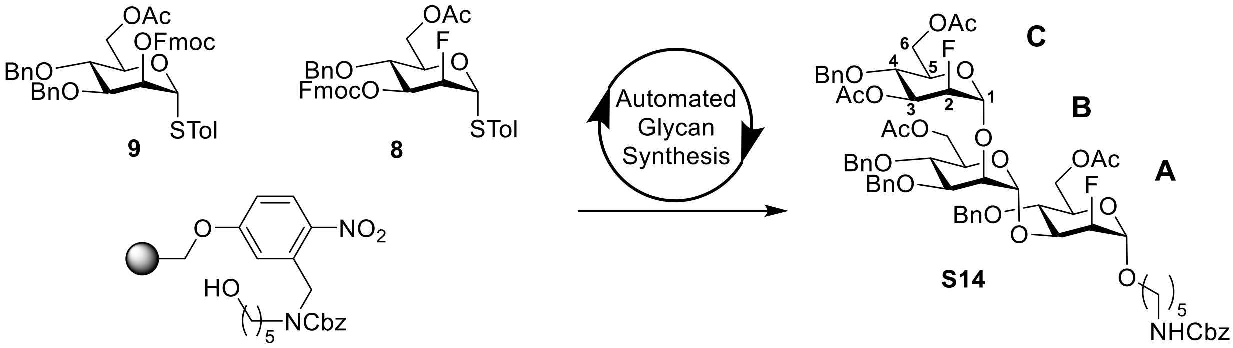

Module	Conditions
A: Resin Swelling	
B: Acid Wash<br>D: Thioglycoside Glycosylation Double<br>E: Acidic Capping<br>F: Fmoc Deprotection	2 × BB **8** (5 eq.), -10 °C for 300 s, 10 °C for 1800 s
B: Acid Wash<br>D: Thioglycoside Glycosylation Double<br>E: Acidic Capping<br>F: Fmoc Deprotection	2 × BB **9** (8 eq.), -20 °C for 300 s, 0 °C for 1200 s
B: Acid Wash<br>D: Thioglycoside Glycosylation Double<br>E: Acidic Capping<br>F: Fmoc Deprotection	2 × BB **8** (5 eq.), -10 °C for 300 s, 10 °C for 1800 s
E: Acidic Capping	

The synthesis was repeated three times on 12.5 µmol scale (based on resin) and the resulting material was combined to enable purification and characterisation. Cleavage from solid support (**Method A**) and column chromatography (SiO_2, CyH/EtOAc, 4/1 → 7/3) afforded a mixture (pale yellow syrup) in which the major species was **S14** and characterisation by NMR and MS was possible.

Due to low sample amounts, certain resonances were not visible in the ^{13}C NMR spectrum.

^{1}H NMR (500 MHz, $CDCl_3$) δ 7.37 – 7.13 (m, 25H, Ar), 5.31 (ddd, *J* = 29.5, 9.6, 2.3 Hz, 1H, H-C3C), 5.12 (d, *J* = 2.0 Hz, 1H, H-C1B), 5.08 (s, 2H, CH_2 Cbz), 4.98 – 4.72 (m, 5H, H-C1C, H-C2C, H-C1A, H-C2A, C*H₂*Ph), 4.72 – 4.65 (m, 2H, C*H₂*Ph), 4.64 – 4.55 (m, 3H, C*H₂*Ph), 4.53 (d, *J* = 11.9, 1H, C*H₂*Ph), 4.46 (d, *J* = 11.1 Hz, 1H, C*H₂*Ph), 4.33 – 4.18 (m, 6H, H-C6A, H-C6B, H-C6C), 4.09 – 3.98 (m, 2H, H-C5B, H-C5C), 3.98 – 3.83 (m, 4H, H-C3A, H-C4B, H-C4C, H-C3B), 3.81 (t, J = 2.4 Hz, 1H, H-C2B), 3.76 – 3.59 (m, 3H, H-C5A, H-C4A, OCH_2 linker), 3.44 – 3.36 (m, 1H, OCH_2 linker), 3.22 – 3.13 (m, 2H, NCH_2 linker), 2.11 (s, 3H, Ac), 2.05 (s, 3H, Ac), 2.01 (s, 3H, Ac), 1.97 (s, 3H, Ac), 1.61 – 1.45 (m, 4H, CH_2 linker), 1.33 (m, 2H, CH_2 linker) ppm; **^{13}C NMR** (101 MHz, $CDCl_3$) δ 171.1 (C=O), 170.9 (C=O), 170.8 (C=O), 169.9 (C=O), 156.5 (Ar), 138.2 (Ar), 138.0 (Ar), 137.7 (Ar), 137.6 (Ar), 136.8 (Ar), 128.8 (Ar), 128.7 (Ar), 128.65 (Ar), 128.62 (Ar), 128.60

(Ar), 128.5 (Ar), 128.3(Ar), 128.24 (Ar), 128.20 (Ar), 128.19 (Ar), 128.0 (Ar), 127.95 (Ar), 127.7 (Ar), 127.6 (Ar), 101.1 (C1B), 99.0 (d, *J* = 29.7 Hz, C1C), 97.2 (d, *J* = 28.8 Hz, C1A), 88.6 (d, *J* = 178.3 Hz, C2A), 87.4 (d, *J* = 178.2 Hz, C2C), 81.0 (d, *J* = 17.4 Hz, C3A), 79.5 (C3B), 77.4 (C2B), 75.6 (CH_2Ph), 75.5 (CH_2Ph), 75.3 (CH_2Ph), 73.9 (C4B), 73.7 (C4A), 72.9 (CH_2Ph, C4C), 72.6 (d, *J* = 16.7 Hz, C3C), 70.7 (C5B), 70.5 (C5C), 69.8 (C5A), 68.2 (OCH_2 linker), 66.7 (CH_2 Cbz), 63.2 (C6A), 63.1 (C6C), 62.8 (C6B), 41.0 (NCH_2 linker), 29.8 (CH_2 linker), 29.1 (CH_2 linker), 23.4 (CH_2 linker), 21.1 (Ac), 21.0 (Ac × 2), 20.8 (Ac) ppm; **^{19}F NMR** (470 MHz, $CDCl_3$) δ -203.32 (ddd, J = 49.4, 29.9, 7.5 Hz, F-C2A), -203.63 (ddd, J = 50.3, 29.6, 6.9 Hz, F-C2C) ppm; **MALDI-MS** m/z [M+Na]$^+$ calcd for $C_{67}H_{79}F_2NO_{20}Na^+$ 1278.51; found 1278.52.

_N_-Benzyloxycarbonyl-5-aminopentyl 4-_O_-benzyl-2-deoxy-2-fluoro-α-D-mannopyranosyl-(1→2)-3,4-di-_O_-benzyl-α-D-mannopyranosyl-(1→3)-4-_O_-benzyl-2-deoxy-2-fluoro-α-D-mannopyranoside (22)

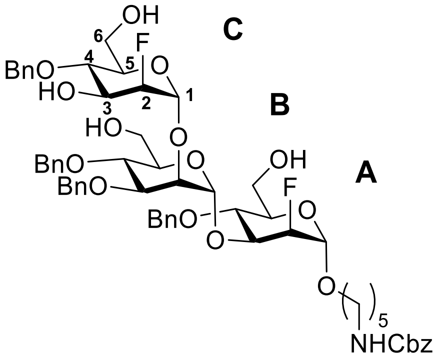

The impure **S14** was subjected to methanolysis (**Method B**) and partially purified with column chromatography (SiO_2, CyH/EtOAc, 2/1). HPLC SEC (**Method C**) afforded pure **22** (7.2 mg, 18% based on resin) as a colourless syrup.

^{1}H NMR (599 MHz, $CDCl_3$) δ 7.39 – 7.19 (m, 25H, Ar), 5.13-5.08 (m, 1H, H-C1B), 5.07 (s, 2H, CH_2 Cbz), 4.93 (dd, *J* = 7.8, 2.0 Hz, 2H, H-C1C), 4.89 – 4.84 (m, 2H, H-C1A, C*H₂*Ph), 4.79 – 4.65 (m, 5H, H-C2A, H-C2C, CH_2Ph), 4.63 (d, *J* = 10.9 Hz, 1H, CH_2Ph), 4.61 – 4.55 (m, 3H, CH_2Ph), 4.05 – 3.82 (m, 4H, H-C3C, H-C5B, H-C3B, H-C3A), 3.82 – 3.56 (m, 13H, H-C5A, H-C4A, H-C6A, H-C6B, H-C6C, H-C5C, H-C4C, H-C4B, H-C2B, OCH_2 linker), 3.42 – 3.37 (m, 1H, OCH_2 linker), 3.21 – 3.13 (m, 2H, NCH_2 linker), 1.60 – 1.46 (m, 4H, CH_2 linker), 1.40 – 1.29 (m, 2H, CH_2 linker) ppm; **^{13}C NMR** (151 MHz, $CDCl_3$) δ 156.7 (C=O Cbz), 138.33 (Ar), 138.28 (Ar), 138.0 (Ar), 136.8 (Ar), 128.78 (Ar), 128.76 (Ar), 128.70 (Ar), 128.65 (Ar), 128.6 (Ar), 128.27 (Ar), 128.25 (Ar), 128.2 (Ar), 128.1 (Ar), 128.0 (Ar), 127.9 (Ar), 127.8 (Ar), 101.2 (C1B), 99.1 (d, *J* = 30.3 Hz, C1C), 97.4 (d, *J* = 28.9 Hz, C1A), 89.7 (d, *J* = 175.4 Hz, C2C), 89.0 (d, *J* = 177.4 Hz, C2A), 79.7 (d, *J* = 17.6 Hz, C3A), 79.4 (C3B), 77.1 (C2B), 75.9 (C4C), 75.6 (CH_2Ph), 75.5 (CH_2Ph), 75.3 (CH_2Ph), 74.5 (C4B), 74.1 (C4A), 73.1 (CH_2Ph), 72.9 (C5B), 72.8 (C5C), 72.2 (C5A), 71.0 (d, *J* = 17.6 Hz, C3C), 68.2 (OCH_2 linker), 66.8 (CH_2 Cbz), 62.0 (C6B, C6C), 61.9 (C6A), 41.1 (NCH_2 linker), 30.0 (CH_2 linker), 29.0 (CH_2 linker), 23.5 (CH_2 linker) ppm; **^{19}F NMR** (470 MHz, $CDCl_3$) δ -203.56 (ddd, *J* = 49.5, 30.0, 7.2 Hz, F-C2A), -205.24 (ddd, *J* = 49.8, 30.2, 7.8 Hz, F-C2C) ppm; **MALDI-MS** m/z [M+Na]$^+$ calcd for $C_{59}H_{71}F_2NO_{16}Na^+$ 1110.46; found 1110.28.

***N*-Benzyloxycarbonyl-5-aminopentyl 2,6-di-*O*-acetyl-3,4-di-*O*-benzyl--α-D-mannopyranosyl-(1→3)-6-*O*-acetyl-4-*O*-benzyl-2-deoxy-2-fluoro-α-D-mannopyranosyl-(1→3)-6-*O*-acetyl-4-*O*-benzyl-2-deoxy-2-fluoro-α-D-mannopyranoside (S15)**

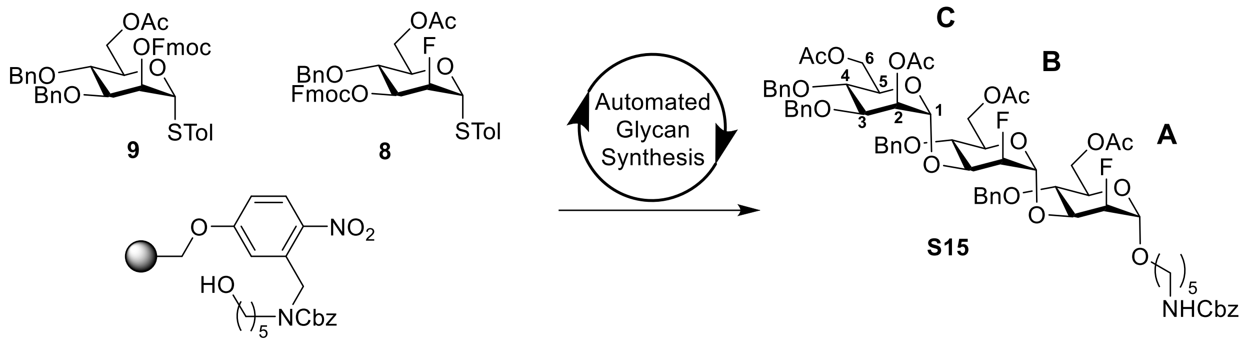

**Module**	**Conditions**
A: Resin Swelling	
B: Acid Wash<br>D: Thioglycoside Glycosylation Double<br>E: Acidic Capping<br>F: Fmoc Deprotection	2 × BB **8** (5 eq.), -10 °C for 300 s, 10 °C for 1800 s
B: Acid Wash<br>D: Thioglycoside Glycosylation Double<br>E: Acidic Capping<br>F: Fmoc Deprotection	2 × BB **8** (5 eq.), -10 °C for 300 s, 10 °C for 1800 s
B: Acid Wash<br>D: Thioglycoside Glycosylation Double<br>E: Acidic Capping<br>F: Fmoc Deprotection	2 × BB **9** (8 eq.), -20 °C for 300 s, 0 °C for 1200 s
E: Acidic Capping	

The synthesis was repeated three times and the resulting material was combined to enable purification and characterisation. Cleavage from solid support (**Method A**) and column chromatography (SiO_2, CyH/EtOAc, 4/1 → 7/3) afforded a mixture (pale yellow syrup) in which the major species was **S15** and characterisation by NMR and MS was possible.

^{1}H NMR (599 MHz, $CDCl_3$) δ 7.45 – 7.12 (m, 25H, Ar), 5.42 (dd, *J* = 3.3, 1.9 Hz, 1H, H-C2C), 5.20 (dd, *J* = 7.4, 2.1 Hz, 1H, H-C1B), 5.10 (d, *J* = 2.0 Hz, 1H, H-C1C), 5.08 (s, 2H, CH_2 Cbz), 4.96 – 4.73 (m, 5H, C*H2*Ph, H-C2A, H-C2B, H-C1A), 4.70 (d, *J* = 10.8 Hz, 1H, CH_2Ph), 4.65 (d, *J* = 11.3 Hz, 1H, CH_2Ph), 4.58 (d, *J* = 10.7 Hz, 1H, CH_2Ph), 4.55 – 4.48 (m, 3H, CH_2Ph), 4.36 – 4.19 (m, 6H, H-C6A, H-C6B, H-C6C), 4.17 – 3.94 (m, 5H, H-C3A, H-C3B, H-C3C, H-C5B, H-C5C), 3.82 – 3.71 (m, 4H, H-C4A, H-C4B, H-C4C, H-C5A), 3.69 – 3.61 (m, 1H, OCH_2 linker), 3.43 – 3.37 (m, 1H, OCH_2 linker), 3.24 – 3.12 (m, 2H, NCH_2 linker), 2.14 (s, 3H, Ac), 2.06 (s, 3H, Ac), 2.04 (s, 3H, Ac), 1.95 (s, 3H, Ac), 1.59 – 1.47 (m, 4H, CH_2 linker), 1.36 – 1.29 (m, 2H, CH_2 linker) ppm; **^{13}C NMR** (151 MHz, $CDCl_3$) δ 170.84 (C=O Ac), 170.76 (C=O Ac), 170.6 (C=O Ac), 170.2 (C=O Ac), 156.5 (C=O Cbz), 138.0 (Ar), 137.7 (Ar), 137.4 (Ar), 137.3 (Ar), 136.7 (Ar), 128.79 (Ar), 128.77 (Ar), 128.63 (Ar), 128.60 (Ar), 128.57 (Ar), 128.4 (Ar), 128.3 (Ar), 128.23 (Ar), 128.22 (Ar), 128.14 (Ar), 128.10 (Ar), 128.05 (Ar), 128.0 (Ar), 100.1 (C1C), 99.4 (d, *J* = 29.5 Hz, C1B), 97.2 (d, *J* = 28.9 Hz, C1A), 88.6 (d, *J*

= 178.4 Hz, C2A), 88.5 (d, *J* = 179.5 Hz, C2B), 80.3 (d, *J* = 17.5 Hz, C3A), 80.2 (d, *J* = 17.6 Hz, C3B), 77.8 (C3C), 75.8 (CH_2Ph), 75.7 (CH_2Ph), 75.4 (CH_2Ph), 74.1 (C4C), 73.7 (C4A), 73.5 (C4B), 72.0 (CH_2Ph), 70.6 (C5C), 70.4 (C5B), 69.8 (C5A), 68.9 (C2C), 68.2 (OCH_2 linker), 66.7 (CH_2 Cbz), 63.4 (C6C), 62.91 (C6B), 62.87 (C6A), 41.0 (NCH_2 linker), 29.9 (CH_2 linker), 29.1 (CH_2 linker), 23.4 (CH_2 linker), 21.1 (Ac), 21.0 (Ac), 20.9 (Ac), 20.8 (Ac) ppm; **^{19}F NMR** (470 MHz, $CDCl_3$) δ -201.97 (ddd, *J* = 49.6, 30.1, 7.5 Hz, F-C2A), -203.43 (ddd, *J* = 49.6, 29.7, 7.1 Hz,F-C2B) ppm; **MALDI-MS** m/z $[M+Na]^+$ calcd for $C_{67}H_{79}F_2NO_{20}Na^+$ 1278.51; found 1278.62.

***N*-Benzyloxycarbonyl-5-aminopentyl 3,4-di-*O*-benzyl-α-D-mannopyranosyl-(1→3)-4-*O*-benzyl-2-deoxy-2-fluoro-α-D-mannopyranosyl-(1→3)-4-*O*-benzyl-2-deoxy-2-fluoro-α-D-mannopyranoside (23)**

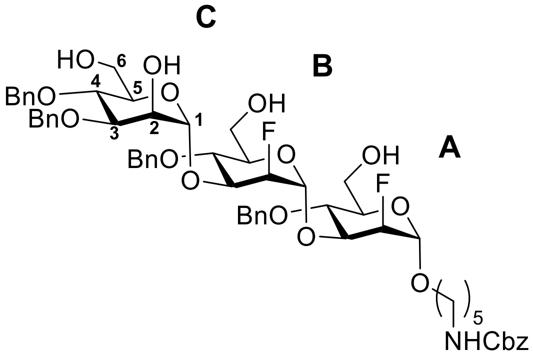

The impure **S15** was subjected to methanolysis (**Method B**) and partially purified with column chromatography (SiO_2, CyH/EtOAc, 2/1 → 1/1). HPLC SEC (**Method C**) afforded pure **23** (5.4 mg, 13% based on resin) as a colourless syrup.

^{1}H NMR (599 MHz, $CDCl_3$) δ 7.42 – 7.21 (m, 25H, Ar), 5.22 (dd, *J* = 7.5, 1.6 Hz, 1H, H-C1B), 5.16 (d, *J* = 1.7 Hz, 1H, H-C1C), 5.09 – 5.06 (m, 2H, CH_2 Cbz), 4.89 – 4.67 (m, 7H, C*H₂*Ph, H-C1A, H-C2A, H-C2B), 4.66 – 4.60 (m, 4H, CH_2Ph), 4.12 – 3.97 (m, 2H, H-C3A, H-C3B), 3.94 (dd, *J* = 3.2, 1.7 Hz, 1H, C-H2C), 3.93 – 3.87 (m, 2H, H-C3C, H-C5B), 3.86 – 3.77 (m, 6H, H-C6B, H-C5C, H-C4A, H-C4B, H-C4C), 3.77 – 3.69 (m, 2H, H-C6A), 3.69 – 3.61 (m, 4H, H-C6C, H-C5A, OCH_2 linker), 3.43 – 3.35 (m, 1H, OCH_2 linker), 3.21 – 3.13 (m, 2H, NCH_2 linker), 1.60 – 1.53 (m, 2H, CH_2 linker), 1.53 – 1.47 (m, 2H, CH_2 linker), 1.38 – 1.31 (m, 2H, CH_2 linker) ppm; **^{13}C NMR** (151 MHz, $CDCl_3$) δ 156.6 (C=O Cbz), 138.3 (Ar), 137.90 (Ar), 137.86 (Ar), 137.71 (Ar), 136.72 (Ar), 128.82 (Ar), 128.76 (Ar), 128.71 (Ar), 128.66 (Ar), 128.6 (Ar), 128.31 (Ar), 128.27 (Ar), 128.25 (Ar), 128.23 (Ar), 128.20 (Ar), 128.18 (Ar), 128.16 (Ar), 128.00 (Ar), 127.97 (Ar), 127.9 (Ar), 101.9 (C1C), 99.6 (d, *J* = 30.4 Hz, C1B), 97.3 (d, *J* = 28.9 Hz, C1A), 89.00 (d, *J* = 177.7 Hz, C2B), 88.98 (d, *J* = 177.7 Hz, C2A) 79.8 (C3C), 79.5 (d, *J* = 16.8 Hz, C3A), 79.1 (d, *J* = 16.7 Hz, C3B), 75.6 (CH_2Ph × 2), 75.4 (CH_2Ph), 74.1 (C4C), 74.0 (C4A), 73.9 (C4B), 72.7 (C5B), 72.5 (CH_2Ph), 72.3 (C5C), 72.2 (C5A), 69.1 (C2C), 68.2 (OCH_2 linker), 66.8 (CH_2 Cbz), 61.9 (C6C), 61.84 (C6A), 61.81 (C6B), 41.0 (NCH_2 linker), 29.8 (CH_2 linker), 29.0 (CH_2 linker), 23.5 (CH_2 linker) ppm; **^{19}F NMR** (470 MHz, $CDCl_3$) δ -202.28 (ddd, J = 49.4, 30.1, 7.4 Hz, F-C2A), -203.53 (ddd, J = 49.2, 29.5, 6.9 Hz, F-C2B) ppm; **MALDI-MS** m/z $[M+Na]^+$ calcd for $C_{59}H_{71}F_2NO_{16}Na^+$ 1110.46; found 1110.35.

Solution-Phase Trisaccharide Synthesis

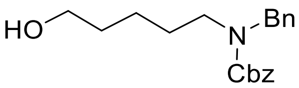

N-(Benzyl)-benzyloxycarbonyl-5-aminopentanol (S16)

Linker **S16** was synthesised as previously described.[13]

^{1}H NMR (599 MHz, $CDCl_3$) δ 7.43 – 7.13 (m, 10H, Ar), 5.24 – 5.13 (m, 2H, CH_2 Cbz), 4.53 – 4.48 (m, 2H, CH_2 NBn), 3.68 – 3.48 (m, 2H, OCH_2), 3.36 – 3.16 (m, 2H, NCH_2), 1.61 – 1.42 (m, 4H, CH_2), 1.40 – 1.19 (m, 2H, CH_2) ppm; **^{13}C NMR** (151 MHz, $CDCl_3$) δ 156.9 (C=O Cbz), 156.4 (C=O Cbz), 138.0 (Ar), 137.0 (Ar), 136.9 (Ar), 128.7 (Ar), 128.6 (Ar), 128.0 (Ar), 127.42 (Ar), 127.35 (Ar), 67.3 (CH_2 Cbz), 62.8 (OCH_2), 50.7 (CH_2 Bn), 50.3 (CH_2 Bn), 47.1 (NCH_2), 46.3 (NCH_2), 32.4 (CH_2), 28.0 (CH_2), 27.6 (CH_2), 23.0 (CH_2) ppm; **IR** (ATR) ṽmax / cm-1 3450 (w), 2933 (w), 1682 (s), 1423 (s), 1366 (m), 1228 (s), 1125 (m), 1073 (m), 913 (m), 729 (s), 695 (s); **HRMS** (ESI) m/z $[M+Na]^+$ calcd for $C_{20}H_{25}NO_3Na^+$ 350.1732; found 350.1724. Spectroscopic data are in accordance with literature.[13]

Fluorinated trisaccharide frameshift **28** was successfully synthesised in a manual solution phase synthesis (Scheme 1).

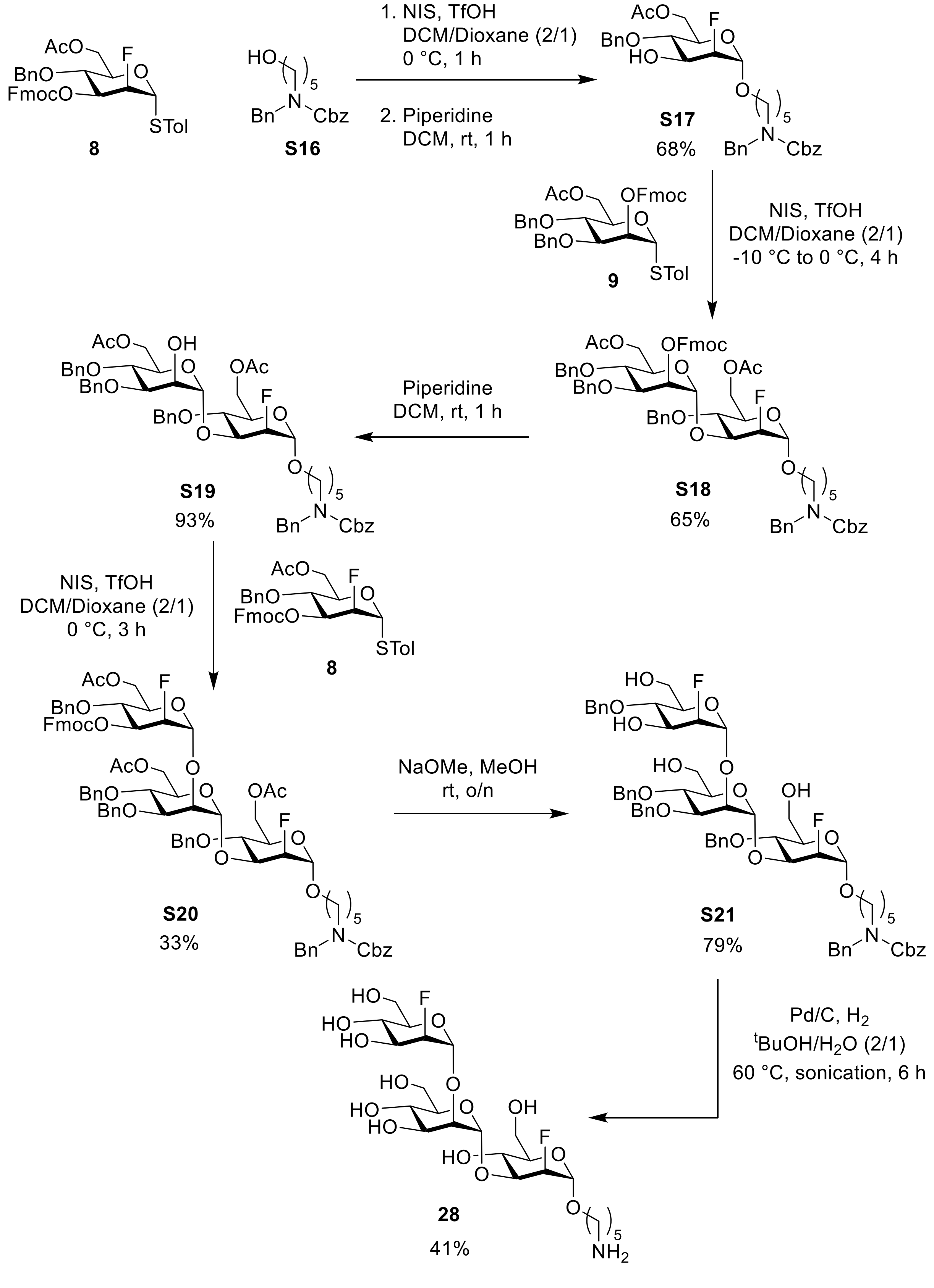

Scheme 1: Overview of route to **28** via manual solution-phase synthesis.

N-(Benzyl)-benzyloxycarbonyl-5-aminopentyl 6-O-acetyl-4-O-benzyl-2-deoxy-2-fluoro-1-thio-α-D-mannopyranoside (S17)

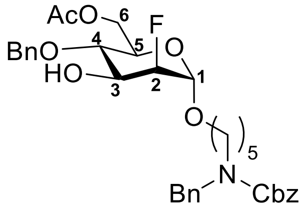

8 (0.20 g, 0.31 mmol, 1.0 eq.) and **S16** (0.15 g, 0.47 mmol, 1.5 eq.) were dissolved in dry DCM (4.10 mL) and dioxane (2.10 mL). Powdered drierite (0.30 g) was added and the mixture was cooled under argon to 0 °C. NIS (87.1 mg, 0.39 mmol, 1.25 eq.) was added before TfOH (14 µL, 0.16 mmol, 0.5 eq.) and the mixture was stirred for 1 h at 0 °C then quenched with aqueous sat. $Na_2S_2O_3$. The mixture was extracted thrice with DCM and dried over $MgSO_4$ before the solvent was removed *in vacuo*. The residue was dissolved in DCM (5 mL) and piperidine (0.5 mL) was added. The mixture was stirred at rt for 1 h then washed with aqueous HCl (1 M) and extracted thrice with DCM. The organic fraction was dried over $MgSO_4$ and the solvent was removed *in vacuo*. Column chromatography (SiO_2, CyH/EtOAc, 4/1) yielded **S17** (134 mg, 68%) as a colourless syrup. Linker- derived side products (**S16a, S16b, S16c**) were also isolated.

Due to low sample amounts, certain resonances were not visible in the ^{13}C NMR spectrum.

R$_f$ (CyH/EtOAc 7/3) 0.27; **^{1}H NMR** (599 MHz, $CDCl_3$) δ 7.31 – 7.13 (m, 14H, Ar), 7.10 – 7.05 (m, 1H, Ar), 5.09 (d, *J* = 14.9 Hz, 2H, CH_2 Cbz), 4.86 – 4.77 (m, 1H, H-C1), 4.75 (d, *J* = 11.1 Hz, 1H, C*H$_2$*Ph), 4.62 – 4.47 (m, 2H, C*H$_2$*Ph, H-C2), 4.40 (d, *J* = 10.3 Hz, 2H, C*H$_2$*Ph NBn), 4.28 (dd, *J* = 12.0, 2.2 Hz, 1H, H-C6), 4.21 (dd, *J* = 12.0, 4.8 Hz, 1H, H-C6), 3.97 – 3.84 (m, 1H, H-C3), 3.72 – 3.64 (m, 1H, H-C5), 3.60 – 3.48 (m, 2H, H-C4, OCH_2 linker), 3.33 – 3.21 (m, 1H, OCH_2 linker), 3.20 – 3.07 (m, 2H, NCH_2 linker), 2.16 (s, 1H, 3-OH), 1.98 (s, 3H, Ac), 1.51 – 1.35 (m, 4H, CH_2 linker), 1.26 – 1.09 (m, 2H, CH_2 linker) ppm; **^{13}C NMR** δ 170.9 (C=O Ac), 138.0 (Ar), 137.9 (Ar), 128.8 (Ar), 128.7 (Ar), 128.6 (Ar), 128.30 (Ar), 128.27 (Ar), 128.1 (Ar), 127.98 (Ar), 127.95 (Ar), 127.5 (Ar), 127.3 (Ar), 97.2 (d, *J* = 29.6 Hz, C1), 89.9 (d, *J* = 174.5 Hz, C2), 75.8 (C4), 75.3 (CH_2Ph OBn), 71.5 (d, *J* = 17.8 Hz, C3), 69.6 (C5), 68.1 (OCH_2 linker), 67.3 (CH_2 Cbz), 63.2 (C6), 50.7 (CH_2Ph NBn), 50.4 (CH_2Ph NBn), 47.2 (NCH_2 linker), 46.2 (NCH_2 linker), 29.1 (CH_2 linker), 28.0 (CH_2 linker), 27.6 (CH_2 linker), 23.5 (CH_2 linker), 21.0 (Ac) ppm; **^{19}F NMR** (564 MHz, $CDCl_3$) δ -206.31 – -206.59 (m) ppm; **HRMS** (ESI) m/z [M+Na]$^+$ calcd for $C_{35}H_{42}FNO_8Na^+$ 646.2792; found 646.2787.

N-(Benzyl)-benzyloxycarbonyl-5-aminopentyl benzyl ether (S16a)

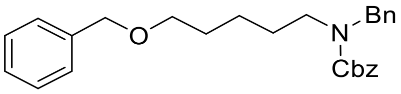

Due to low sample amounts, certain resonances were not visible in the ^{13}C NMR spectrum.

^{1}H NMR (599 MHz, $CDCl_3$) δ 7.43 – 7.14 (m, 15H, Ar), 5.21 – 5.13 (m, 2H, CH_2 Cbz), 4.53 – 4.43 (m, 4H, N*CH₂*Ph, O*CH₂*Ph Bn), 3.49 – 3.35 (m, 2H, OCH_2), 3.32 – 3.15 (m, 2H, NCH_2), 1.68 – 1.44 (m, 4H, CH_2), 1.38 – 1.20 (m, 2H, CH_2) ppm; **^{13}C NMR** (151 MHz, $CDCl_3$) δ 138.8 (Ar), 138.1 (Ar), 137.1 (Ar), 128.7 (Ar), 128.6 (Ar), 128.5 (Ar), 128.1 (Ar), 128.0 (Ar), 127.8 (Ar), 127.7 (Ar), 127.4 (Ar), 73.0 (OCH_2Ph Bn), 70.3 (OCH_2), 67.3 (CH_2 Cbz), 50.6 (CH_2Ph NBn), 50.3 (CH_2Ph NBn), 47.3 (NCH_2), 46.3 (NCH_2), 29.6 (CH_2), 28.1 (CH_2), 27.7 (CH_2), 23.6 (CH_2) ppm; **IR** (ATR) ṽmax / cm-1 2933 (w), 1699 (s), 1492 (m), 1452 (m), 1418 (m), 1360 (m), 1223 (m), 1091 (m), 1027 (m), 735 (s), 695 (s); **HRMS** (ESI) m/z $[M+Na]^+$ calcd for $C_{27}H_{31}NO_3Na^+$ 440.2202; found 440.2197.

N-(Benzyl)-benzyloxycarbonyl-5-aminopentyl 4-methylbenzenesulfinate (S16b)

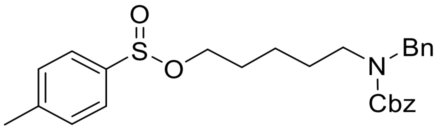

R$_f$ (CyH/EtOAc 7/3) 0.29; **^{1}H NMR** (599 MHz, $CDCl_3$) δ 7.57 (d, J = 7.7 Hz, 2H, Ar), 7.41 – 7.11 (m, 12H, Ar), 5.17 (d, J = 9.8 Hz, 2H, CH_2 Cbz), 4.48 (d, J = 9.4 Hz, 2H, CH_2 Bn), 4.04 – 3.88 (m, 1H, OCH_2), 3.61 – 3.46 (m, 1H, OCH_2), 3.29 – 3.13 (m, 2H, NCH_2), 2.42 (s, 3H, Tol), 1.65 – 1.41 (m, 4H, CH_2), 1.36 – 1.19 (m, 2H, CH_2) ppm; **^{13}C NMR** (151 MHz, $CDCl_3$) δ 156.8 (C=O Cbz), 156.3 (C=O Cbz), 142.8 (Tol), 141.9 (Tol), 138.0 (Bn), 136.94 (Cbz), 136.88 (Cbz), 129.8 (Ar), 128.7 (Ar), 128.6 (Ar), 128.1 (Ar), 128.0 (Ar), 127.4 (Ar), 127.3 (Ar), 125.3 (Ar), 67.3 (CH_2 Cbz), 64.3 (OCH_2), 64.2 (OCH_2), 50.6 (NCH_2), 50.3 (NCH_2), 47.0 (CH_2), 46.1 (CH_2), 29.5 (CH_2), 27.8 (CH_2), 27.3 (CH_2), 23.1 (CH_2), 21.6 (Tol) ppm; **IR** (ATR) ṽmax / cm-1 2839 (w), 1693 (s), 1452 (w), 1223 (w), 1131 (m), 941 (w), 815 (w), 735 (m), 700 (s); **HRMS** (ESI) m/z $[M+Na]^+$ calcd for $C_{27}H_{31}NO_4SNa^+$ 488.1871; found 488.1866.

Di-N-(benzyl)-benzyloxycarbonyl-5-aminopentyl ether (S16c)

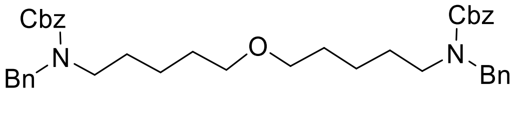

R$_f$ (CyH/EtOAc 4/1) 0.23; **^{1}H NMR** (599 MHz, $CDCl_3$) δ 7.41 – 7.12 (m, 20H, Ar), 5.21 – 5.13 (m, 4H, CH_2 Cbz), 4.53 – 4.46 (m, 4H, *CH₂*Ph NBn), 3.38 – 3.14 (m, 8H, OCH_2, NCH_2), 1.58 – 1.44 (m, 8H, CH_2), 1.37 – 1.20 (m, 4H, CH_2) ppm; **^{13}C NMR** (151 MHz, $CDCl_3$) δ 156.9 (C=O), 156.3 (C=O), 138.1 (Ar), 137.1 (Ar), 137.0 (Ar), 128.7 (Ar), 128.6 (Ar), 128.1 (Ar), 128.0 (Ar), 127.44 (Ar), 127.39 (Ar), 70.8 (OCH_2), 67.3 (CH_2 Cbz), 50.7 (CH_2Ph Bn), 50.4 (CH_2Ph Bn), 47.3 (NCH_2), 46.3 (NCH_2), 29.6 (CH_2), 28.1 (CH_2), 27.7 (CH_2), 23.6

(CH$_2$) ppm; **IR** (ATR) ṽmax / cm-1 2933 (w), 1693 (s), 1452 (m), 1418 (m), 1223 (s), 1091 (s), 1027 (m), 798 (m), 735 (s), 695 (s); **HRMS** (ESI) m/z [M+Na]$^+$ calcd for $C_{40}H_{48}N_2O_5Na^+$ 659.3461; found 659.3458.

***N*-(Benzyl)-benzyloxycarbonyl-5-aminopentyl 6-*O*-acetyl-3,4-di-O-benzyl-2-*O*-fluorenylmethoxycarbonyl-α-D-mannopyranosyl-(1→3)-6-*O*-acetyl-4-*O*-benzyl-2-deoxy-2-fluoro-α-D-mannopyranoside (S18)**

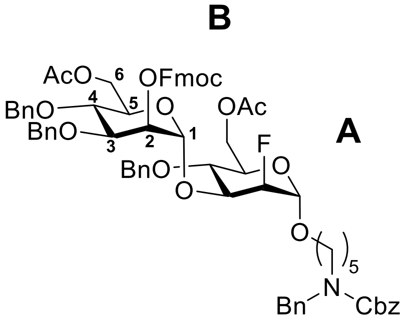

S17 (134 mg, 0.215 mmol, 1.0 eq.) and **9** (236 mg, 0.323 mmol, 1.5 eq.) were dissolved in dry DCM (4.10 mL) and dioxane (2.10 mL) and powdered drierite (0.30 g) was added. The mixture was cooled to -10 °C under argon then NIS (60.5 mg, 0.269 mmol, 1.25 eq.) was added before TfOH (9.5 µL, 0.11 mmol, 0.5 eq.). The mixture was stirred and allowed to warm to 0 °C over 1 h then stirred at 0 °C for 3 h. The mixture was quenched with aqueous sat. $Na_2S_2O_3$. The mixture was extracted thrice with DCM and dried over $MgSO_4$ then the solvent was removed *in vacuo*. Column chromatography (SiO$_2$, CyH/EtOAc, 9/1) afforded **S18** (172 mg, 65%) as a white foam.

^{1}H NMR (599 MHz, CDCl$_3$) δ 7.83 – 7.77 (m, 2H, Ar), 7.67 – 7.58 (m, 2H, Ar), 7.45 – 7.40 (m, 2H, Ar), 7.39 – 7.22 (m, 23H, Ar), 7.22 – 7.13 (m, 4H, Ar), 5.26 (dd, *J* = 3.1, 1.9 Hz, 1H, H-C2B), 5.25 – 5.21 (s, 1H, H-C1B), 5.21 – 5.13 (m, 2H, C*H$_2$*Ph Cbz), 4.97 (d, *J* = 10.8 Hz, 1H, C*H$_2$*Ph OBn), 4.91 – 4.78 (m, 2H, H-C1A, H-C2A), 4.74 (d, *J* = 10.7 Hz, 1H, C*H$_2$*Ph OBn), 4.71 (d, *J* = 11.4 Hz, 1H, C*H$_2$*Ph OBn), 4.65 (d, *J* = 10.8 Hz, 1H, C*H$_2$*Ph OBn), 4.59 (d, *J* = 11.5 Hz, 1H, C*H$_2$*Ph OBn), 4.53 – 4.44 (m, 4H, C*H$_2$*Ph OBn, CH$_2$ Fmoc, C*H$_2$*Ph NBn), 4.41 (dd, *J* = 11.9, 2.2 Hz, 1H, H-C6B), 4.38 – 4.31 (m, 2H, CH$_2$ Fmoc, H-C6B), 4.31 – 4.22 (m, 3H, CH Fmoc, H-C6A), 4.16 (ddd, *J* = 10.0, 5.5, 2.2 Hz, 1H, H-C5B), 4.11 – 3.97 (m, 2H, H-C3A, H-C3B), 3.87 (t, *J* = 9.6 Hz, 1H, H-C4B), 3.81 – 3.72 (m, 2H, H-C4A, H-C5A), 3.66 – 3.51 (m, 1H, OCH$_2$ linker), 3.41 – 3.28 (m, 1H, OCH$_2$ linker), 3.28 – 3.16 (m, 2H, NCH$_2$ linker), 2.06 (s, 3H, Ac), 2.04 (s, 3H, Ac), 1.58 – 1.43 (m, 4H, CH$_2$ linker), 1.31 – 1.16 (m, 2H, CH$_2$ linker) ppm; **^{13}C NMR** (151 MHz) δ 170.9 (C=O Ac), 170.8 (C=O Ac), 156.3 (C=O Cbz), 154.7 (C=O Fmoc), 143.6 (Fmoc), 143.3 (Fmoc), 141.5 (Fmoc), 141.4 (Fmoc), 138.0 (Ar), 137.7 (Ar), 137.5 (Ar), 136.97 (Ar), 136.96 (Ar), 136.9 (Ar), 128.8 (Ar), 128.7 (Ar), 128.64 (Ar), 128.58 (Ar), 128.5 (Ar), 128.33 (Ar), 128.29 (Ar), 128.1 (Ar), 127.98 (Ar), 127.97 (Ar), 127.3 (Ar), 125.4 (Ar), 125.3 (Ar), 120.2 (Ar), 99.8 (C1B), 97.2 (d, J = 30.1 Hz, C1A), 88.6 (d, J = 178.9 Hz, C2A), 80.4 (d, *J* = 17.6 Hz, C3A), 78.0 (C3B), 75.7 (CH$_2$Ph OBn), 75.5 (CH$_2$Ph OBn), 74.1 (C4B), 73.6 (C4A), 72.9 (C2B), 72.1 (CH$_2$Ph OBn), 70.6 (C5B), 70.5 (CH$_2$ Fmoc), 69.8 (C5A), 68.2 (OCH$_2$ linker), 67.3 (CH$_2$Ph Cbz), 63.4 (C6B), 63.0 (C6A), 50.4 (CH$_2$Ph NBn), 47.2 (NCH$_2$ linker), 46.8 (CH Fmoc), 46.2 (NCH$_2$ linker), 29.2 (CH$_2$ linker), 27.6 (CH$_2$ linker), 23.4 (CH$_2$ linker), 21.0 (Ac), 20.9 (Ac) ppm; **^{19}F NMR** (564 MHz, CDCl$_3$) δ -203.45 (ddd, J = 49.9, 29.8, 7.2 Hz) ppm; **HRMS** (ESI) m/z [M+Na]$^+$ calcd for $C_{72}H_{76}FNO_{16}Na^+$ 1252.5046; found 1252.5045.

N-(Benzyl)-benzyloxycarbonyl-5-aminopentyl 6-O-acetyl-3,4-di-O-benzyl-α-D-mannopyranosyl-(1→3)-6-O-acetyl-4-O-benzyl-2-deoxy-2-fluoro-α-D-mannopyranoside (S19)

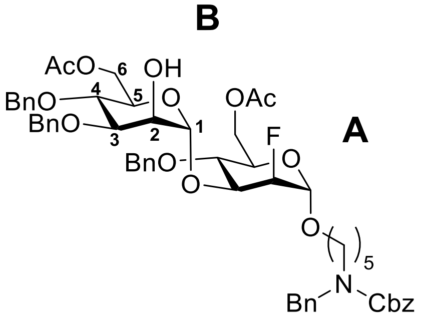

S18 (165 mg, 0.134 mmol, 1.0 eq.) was dissolved in DCM (5 mL) and piperidine (0.5 mL) was added. The mixture was stirred for 1 h then washed with aqueous HCl (1 M) and extracted thrice with DCM. The organic fractions were dried over $MgSO_4$ and the solvent was removed *in vacuo*. Column chromatography (SiO_2, CyH/EtOAc, 3/2) afforded **S19** (126 mg, 93%) as a colourless syrup.

R_f (CyH/EtOAc 7/3) 0.17; **^{1}H NMR** (599 MHz, $CDCl_3$) δ 7.38 – 7.21 (m, 24H, Ar), 7.19 – 7.14 (m, 1H, Ar), 5.20 – 5.14 (m, 2H, CH_2 Cbz), 5.13 (s, 1H, H-C1B), 4.92 – 4.77 (m, 3H, CH_2Ph, H-C2A, H-C1A), 4.70 (d, *J* = 11.6 Hz, 1H, CH_2Ph), 4.67 – 4.62 (m, 2H, CH_2Ph, CH_2Ph), 4.61 (d, *J* = 10.8 Hz, 1H, CH_2Ph), 4.53 – 4.45 (m, 3H, CH_2Ph, CH_2Ph NBn), 4.34 (dd, *J* = 11.9, 2.2 Hz, 1H, H-C6B), 4.30 – 4.23 (m, 3H, H-C6B, H-C6A), 4.15 – 4.09 (m, 1H, H-C5B), 4.04 – 3.95 (m, 2H, H-C3A, H-C2B), 3.94 (dd, *J* = 8.9, 3.3 Hz, 1H, H-C3B), 3.81 – 3.70 (m, 3H, H-C4B, H-C4A, H-C5A), 3.66 – 3.53 (m, 1H, OCH_2 linker), 3.41 – 3.28 (m, 1H, OCH_2 linker), 3.28 – 3.15 (m, 2H, NCH_2 linker), 2.05 (s, 3H, Ac), 2.02 (s, 3H, Ac), 1.59 – 1.43 (m, 4H, CH_2 linker), 1.31 – 1.16 (m, 2H, CH_2 linker) ppm; **^{13}C NMR** (151 MHz, $CDCl_3$) δ 170.9 (C=O Ac), 170.8 (C=O Ac), 156.3 (C=O Cbz), 138.03 – 137.96 (m, Ar), 137.8 (Ar), 137.6 (Ar), 128.74 (Ar), 128.68 (Ar), 128.63 (Ar), 128.61 – 128.52 (m, Ar), 128.3 (Ar), 128.22 (Ar), 128.19 (Ar), 128.1 (Ar), 127.99 (Ar), 127.95 (Ar), 127.5 (Ar), 127.3 (Ar), 101.8 (C1B), 97.3 (d, *J* = 29.0 Hz, C1A), 88.7 (d, *J* = 176.8 Hz, C2A), 80.4 (d, *J* = 16.4 Hz, C3A), 80.0, (C3B), 75.6 (CH_2Ph), 75.3 (CH_2Ph), 74.1 (C4B), 73.7 (C4A), 72.4 (CH_2Ph), 70.0 (C5B), 69.8 (C5A), 69.0 (C2B), 68.2 (OCH_2 linker), 67.3 (CH_2 Cbz), 63.5 (C6B), 63.0 (C6A), 50.7 (CH_2Ph NBn), 50.4 (CH_2Ph NBn), 47.2 (NCH_2 linker), 46.3 (NCH_2 linker), 29.1 (CH_2 linker), 28.0 (CH_2 linker), 27.6 (CH_2 linker), 23.4 (CH_2 linker), 21.0 (Ac), 20.9 (Ac) ppm; **^{19}F NMR** (564 MHz, $CDCl_3$) δ -203.51 (ddd, *J* = 49.3, 30.0, 7.3 Hz) ppm; **HRMS** (ESI) m/z $[M+Na]^+$ calcd for $C_{57}H_{66}FNO_{14}Na^+$ 1030.4365; found 1030.4363.

***N*-(Benzyl)-benzyloxycarbonyl-5-aminopentyl 6-*O*-acetyl-4-*O*-benzyl-3-*O*-fluorenylmethoxycarbonyl -2-deoxy-2-fluoro-α-D-mannopyranosyl-(1→2)-6-*O*-acetyl-3,4-di-*O*-benzyl-α-D-mannopyranosyl-(1→3)-6-*O*-acetyl-4-*O*-benzyl-2-deoxy-2-fluoro-α-D-mannopyranoside (S20)**

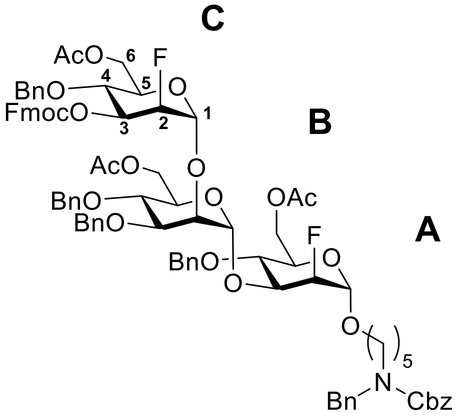

S19 (90.0 mg, 0.089 mmol, 1.0 eq.) and **16** (86 mg, 0.13 mmol, 1.5 eq.) were dissolved in dry DCM (2.05 mL) and dioxane (1.05 mL) then drierite (0.25 g) was added. The mixture was cooled to 0 °C under argon before the addition of NIS (25.1 mg, 0.112 mmol, 1.25 eq.) then TfOH (3.95 µL, 0.0447 mmol, 0.5 eq.). The mixture was stirred at 0 °C for 3 h then quenched with aqueous sat. $Na_2S_2O_3$. The mixture was extracted thrice with DCM and the organic fractions were dried over $MgSO_4$ before the solvent was removed *in vacuo*. Preparative TLC (CyH/EtOAc, 7/3) yielded **S20** (44.4 mg, 33%) as a colourless foam.

R_f (CyH/EtOAc 3/2) 0.62; **^{1}H NMR** (599 MHz, $CDCl_3$) δ 7.79 – 7.74 (m, 2H, Fmoc), 7.64 – 7.59 (m, 2H, Fmoc), 7.42 – 7.38 (m, 2H, Fmoc), 7.37 – 7.14 (m, 32H, Ar), 5.21 – 5.13 (m, 3H, CH_2 Cbz, H-C3C), 5.13 – 5.10 (m, 1H,H-C1B), 5.00 (d, *J* = 6.5 Hz, 1H, H-C1C), 5.00 (dt, *J* = 50.6, 2.2 Hz, 1H, H-C2C), 4.87 – 4.72 (m, 4H, H-C1A, H-C2A, C*H₂*Ph OBn), 4.70 (d, *J* = 11.9 Hz, 1H, CH_2Ph OBn), 4.61 – 4.56 (m, 3H, CH_2Ph OBn), 4.53 (d, *J* = 11.9 Hz, 1H, CH_2Ph OBn), 4.51 – 4.41 (m, 5H, CH_2Ph OBn, CH_2 Fmoc, CH_2Ph NBn), 4.30 – 4.19 (m, 7H, H-C6A, H-C6B, H-C6C, CH Fmoc), 4.07 – 4.03 (m, 1H, H-C5C), 4.02 (ddd, *J* = 10.0, 4.0, 2.2 Hz, 1H, H-C5B), 3.98 – 3.87 (m, 3H, H-C4C, H-C3B, H-C3A), 3.85 (t, *J* = 9.7 Hz, 1H, H-C4B), 3.83 – 3.80 (m, 1H, H-C2B), 3.75 – 3.66 (m, 2H, H-C4A, H-C5A), 3.63 – 3.49 (m, 1H, OCH_2 linker), 3.41 – 3.27 (m, 1H OCH_2 linker), 3.27 – 3.15 (m, 2H NCH_2 linker), 2.05 (s, 3H, Ac), 2.00 (s, 3H, Ac), 1.92 (s, 3H, Ac), 1.57 – 1.42 (m, 4H, CH_2 linker), 1.32 – 1.14 (m, 2H, CH_2 linker) ppm; **^{13}C NMR** (151 MHz, $CDCl_3$) δ 171.1 (C=O Ac), 170.8 (C=O Ac), 170.7 (C=O Ac), 156.3 (C=O Cbz), 154.4 (C=O Fmoc), 143.4 (Fmoc), 143.2 (Fmoc), 141.47 (Fmoc), 141.45 (Fmoc), 138.1 (Ar), 138.00 (Ar), 137.98 (Ar), 137.7 (Ar), 137.4 (Ar), 137.0 (Ar), 128.8 (Ar), 128.7 (Ar), 128.60 (Ar), 128.59 (Ar), 128.5 (Ar), 128.3 (Ar), 128.21 (Ar), 128.20 (Ar), 128.13 (Ar), 128.08 (Ar), 128.02 (Ar), 128.00 (Ar), 127.9 (Ar), 127.7 (Ar), 127.37 (Ar), 127.35 (Ar), 125.23 (Fmoc), 125.16 (Fmoc), 120.3 (Fmoc), 101.1 (C1B), 98.9 (d, *J* = 29.0 Hz, C1C), 97.2 (d, *J* = 28.8 Hz, C1A), 88.6 (d, *J* = 177.4 Hz, C2A), 86.9 (d, *J* = 179.1 Hz, C2C), 80.9 (d, *J* = 17.4 Hz, C3A), 79.6 (C3B), 77.4 (C2B), 76.95 – 76.73 (m, C3C), 75.6 (CH_2Ph OBn), 75.43 (CH_2Ph OBn), 75.41 (CH_2Ph OBn), 73.9 (C4B), 73.7 (C4A), 73.0 (CH_2Ph OBn), 72.5 (C4C), 70.7 (C5B), 70.5 (C5C), 70.4 (CH_2 Fmoc), 69.7 (C5A), 68.3 (OCH_2 linker), 67.3 (CH_2 Cbz), 63.1 (C6C), 63.0 (C6A), 62.9 (C6B), 50.7 (CH_2Ph NBn), 50.4 (CH_2Ph NBn), 47.2 (NCH_2 linker), 46.2 (NCH_2 linker), 46.8 (CH Fmoc), 29.1 (CH_2 linker), 28.0 (CH_2 linker), 27.5 (CH_2 linker), 23.3 (CH_2 linker), 20.98 (Ac), 20.96 (Ac), 20.7 (Ac) ppm; **^{19}F NMR** (563 MHz, $CDCl_3$) δ -203.30 (ddd, *J* = 49.3, 29.7, 7.5 Hz, F-C2A), -203.64 (ddd, *J* = 50.7, 28.9, 6.4 Hz, F-C2C) ppm; **MALDI-MS** m/z $[M+Na]^+$ calcd for $C_{87}H_{93}F_2NO_{21}Na^+$ 1548.61; found 1548.68.

***N*-(Benzyl)-benzyloxycarbonyl-5-aminopentyl 4-*O*-benzyl-2-deoxy-2-fluoro-α-D-mannopyranosyl-(1→2)-3,4-di-*O*-benzyl-α-D-mannopyranosyl-(1→3)-4-*O*-benzyl-2-deoxy-2-fluoro-α-D-mannopyranoside (S21)**

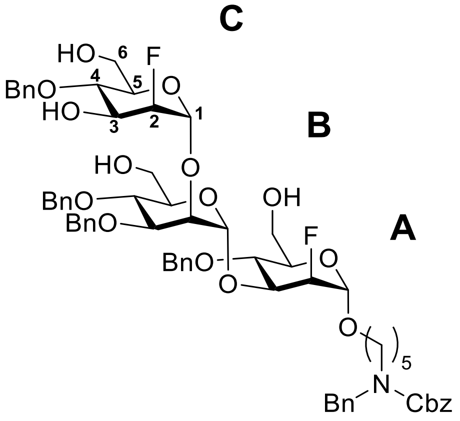

S20 (44 mg, 0.029 mmol, 1.0 eq.) and NaOMe (12 mg, 0.22 mmol, 5.0 eq.) were dissolved in MeOH (3 mL) and stirred at rt for 15 h. The solution was neutralised with Amberlyst™[H$^+$] and filtered. Solvent was removed *in vacuo* to yield a residue that was purified with column chromatography (SiO_2, CyH/EtOAc, 4/1) to yield **S21** (26.8 mg, 79%) as a colourless syrup.

^{1}H NMR (500 MHz, $CDCl_3$) δ 7.39 – 7.12 (m, 30H, Ar), 5.22 – 5.07 (m, 3H, CH_2 Cbz, H-C1B), 5.00 – 4.93 (m, 1H, H-C1A/C), 4.90 – 4.53 (m, 11H, H-C1A/C, H-C2A, H-C2C, C*H$_2$*Ph × 8), 4.53 – 4.45 (m, 2H, C*H$_2$*Ph NBn), 4.07 – 3.88 (m, 3H, H-C2/3/4/5/A/B/C H-C3A, H-C3C), 3.87 – 3.54 (m, 14H, H-C2/3/4/5/A/B/C × 7, H-C6A, H-C6B, H-C6C OCH_2 linker), 3.43 – 3.13 (m, 3H, OCH_2 linker, NCH_2 linker), 1.60 – 1.44 (m, 4H, CH_2 linker), 1.28 – 1.17 (m, 2H, CH_2 linker) ppm; **^{13}C NMR** (126 MHz, $CDCl_3$) δ 156.4 (C=O Cbz), 138.3 (Ar), 138.0 (Ar), 137.9 (Ar), 128.74 (Ar), 128.73 (Ar), 128.67 (Ar), 128.6 (Ar), 128.23 (Ar), 128.21 (Ar), 128.18 (Ar), 128.08 (Ar), 128.06 (Ar), 128.0 (Ar), 127.9 (Ar), 127.8 (Ar), 127.4 (Ar), 127.3 (Ar), 101.2 (C1B), 99.0 (d, *J* = 29.3 Hz, C1A/C), 97.3 (d, *J* = 28.8 Hz, C1A/C), 89.7 (d, *J* = 174.8 Hz, C2A/C), 89.0 (d, *J* = 177.1 Hz, C2A/C), 79.7 (d, *J* = 16.7 Hz, C3A/C), 79.4 (C2/3/4/5/A/B/C), 75.8 (C2/3/4/5/A/B/C), 75.6 (C*H$_2$*Ph), 75.5 (C*H$_2$*Ph), 75.3 (C*H$_2$*Ph), 74.4 (C2/3/4/5/A/B/C), 74.0 (C2/3/4/5/A/B/C), 73.0 (C*H$_2$*Ph), 72.9 (C2/3/4/5/A/B/C), 72.8 (C2/3/4/5/A/B/C), 72.2 (C2/3/4/5/A/B/C), 71.0 (d, *J* = 17.9 Hz, C3A/C), 68.0 (C2/3/4/5/A/B/C), 67.4 (CH_2 Cbz), 61.9 (C6A, C6B, C6C), 50.3 (C*H$_2$*Ph NBn), 47.2 (NCH_2 linker), 46.1 (NCH_2 linker), 29.0 (CH_2 linker), 27.9 (CH_2 linker), 27.5 (CH_2 linker), 23.3 (CH_2 linker) ppm; **MALDI-MS** m/z [M+Na]$^+$ calcd for $C_{66}H_{77}F_2NO_{16}Na^+$ 1200.51; found 1200.61.

5-aminopentyl 2-deoxy-2-fluoro-α-D-mannopyranosyl-(1→2)-α-D-mannopyranosyl-(1→3)-2-deoxy-2-fluoro-α-D-mannopyranoside (28)

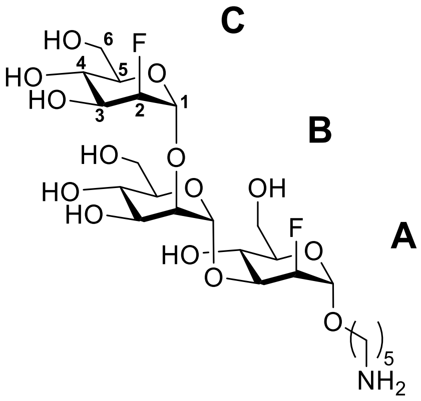

Pretreated Pd/C (40 mg) was added to a solution of **S21** (7 mg, 6 µmol, 1 eq.) in ^{t}BuOH/H_2O (2/1, v/v, 2 mL). The flask was equipped with a H_2 balloon and sonicated for 6 h. The mixture was filtered over celite and washed with the reaction solvent mixture. Freeze drying yielded **28** (1.3 mg, 41%) as a colourless syrup.

^{1}H NMR (599 MHz, D_2O) δ 5.42 (d, *J* = 1.8 Hz, 1H, H-C1B), 5.30 (dd, *J* = 7.5, 2.0 Hz, 1H, H-C1C), 5.10 (dd, *J* = 7.3, 1.9 Hz, 1H, H-C1A), 4.95 (dt, *J* = 49.0, 2.1 Hz, 1H, H-C2A), 4.92 (dt, *J* = 49.5, 2.2 Hz, 1H, H-C2C), 4.17 (dd, *J* = 3.3, 1.8 Hz, 1H, H-C2B), 4.03 – 3.86 (m, 6H, H-C3A, H-C3B, H-C3C, H-C6A, H-C6B, H-C6C), 3.86 – 3.66 (m, 10H, H-C4A, H-C4B, H-C4C, H-C5A, H-C5B, H-C5C, H-C6A, H-C6B, H-C6C, OCH_2 linker), 3.64 – 3.56 (m, 1H, OCH_2 linker), 3.06 – 2.98 (m, 2H, NCH_2 linker), 1.76 – 1.63 (m, 4H, CH_2 linker), 1.53 – 1.42 (m, 2H, CH_2 linker) ppm; **^{13}C NMR** (151 MHz, D_2O) δ 101.2 (C1B), 99.9 (d, *J* = 30.2 Hz, C1C), 97.4 (d, *J* = 29.1 Hz, C1A), 90.4 (d, *J* = 172.4 Hz, C2C), 90.2 (d, *J* = 173.3 Hz, C2A), 79.4 (C2B), 78.4 (d, *J* = 16.8 Hz, C3A), 74.0 (C5C), 73.9 (C5B), 73.3 (C5A), 70.5 (C3B), 70.2 (d, *J* = 17.2 Hz, C3C), 68.5 (OCH_2 linker), 67.5 (C4B), 67.3 (d, *J* = 1.6 Hz, C4C), 66.5 (d, *J* = 1.4 Hz, C4A), 61.6 (C6B), 61.2 (C6C), 61.1 (C6A), 40.0 (NCH_2 linker), 28.6 (CH_2 linker), 27.2 (CH_2 linker), 23.0 (CH_2 linker) ppm; **^{19}F NMR** (564 MHz, D_2O) δ -203.54 (ddd, *J* = 49.3, 30.9, 7.4 Hz, F-C2A), -204.99 (ddd, *J* = 49.5, 31.8, 7.6 Hz, F-C2C) ppm; **HRMS** (ESI) m/z $[M+H]^+$ calcd for $C_{23}H_{41}F_2NO_{14}H^+$ 594.2568; found 594.2568.

Manual solution-phase synthesis of fluorinated frameshift **S22** was attempted but side reactions led to its manual synthesis being abandoned (Scheme 2).

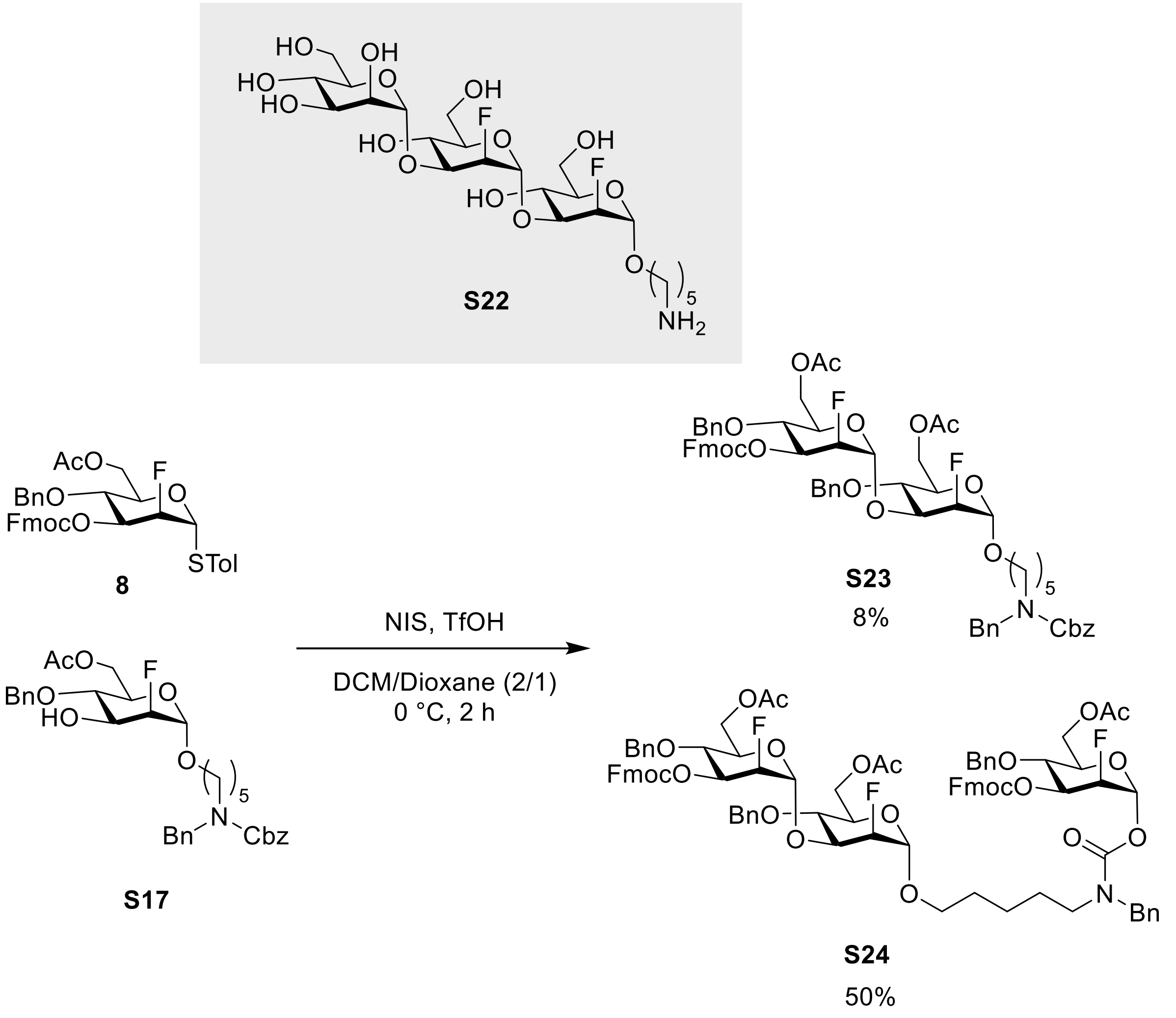

Scheme 2: Fluorinated frameshift **S22**: attempted route.

_N_-(Benzyl)-benzyloxycarbonyl-5-aminopentyl 6-_O_-acetyl-4-_O_-benzyl-3-_O_-fluorenylmethoxycarbonyl -2-deoxy-2-fluoro-α-D-mannopyranosyl-(1→3)-6-_O_-acetyl-4-_O_-benzyl-2-deoxy-2-fluoro-α-D-mannopyranoside (S23)

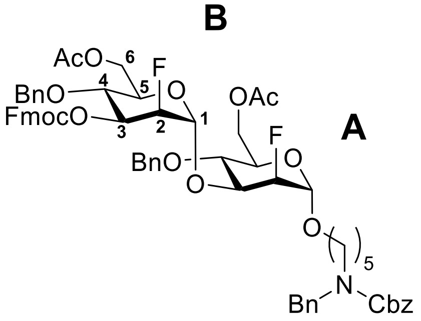

S17 (180 mg, 0.289 mmol, 1.0 eq.) and **8** (371 mg, 0.578 mmol, 2.0 eq.) were dissolved in dry DCM (4.10 mL) and dioxane (2.10 mL) then cooled to 0 °C under argon. NIS (137 mg, 0.607 mmol, 2.1 eq.) was added with drierite (0.30 g) followed by TfOH (12.8 µL, 0.145 mmol, 0.5 eq.) and the mixture was stirred for 2 h at 0 °C then quenched with aqueous sat. $Na_2S_2O_3$. The mixture was extracted thrice with DCM, dried over $MgSO_4$ and the solvent was removed from the organic fractions *in vacuo*. Column chromatography (SiO_2, CyH/EtOAc, 4/1) afforded **S23** (25.2 mg, 8%) as a colourless syrup. A major side product **S24** (227.3 mg, 50%) was isolated as a colourless solid.

Due to low sample amounts, certain resonances were not visible in the ^{13}C NMR spectrum.

R_f (CyH/EtOAc 7/3) 0.30; **^{1}H NMR** (500 MHz, $CDCl_3$) δ 7.80 – 7.75 (m, 2H, Fmoc), 7.64 – 7.59 (m, 2H, Fmoc), 7.43 – 7.38 (m, 2H, Fmoc), 7.38 – 7.13 (m, 22H, Ar), 5.28 – 5.13 (m, 4H, H-C1B, H-C1B, CH_2 Cbz), 4.93 – 4.71 (m, 5H, H-C2A, H-C2B, H-C1A, C*H₂*Ph), 4.62 (d, *J* = 11.1 Hz, 1H, C*H₂*Ph), 4.58 (d, *J* = 10.9 Hz, 1H, C*H₂*Ph), 4.53 – 4.41 (m, 4H, C*H₂*Ph NBn, CH_2 Fmoc), 4.39 (dd, *J* = 12.0, 2.2 Hz, 1H, H-C6B), 4.34 – 4.25 (m, 4H, H-C6B, H-C6A, CH Fmoc), 4.24 – 4.18 (m, 1H, H-C5B), 4.07 – 3.94 (m, 2H, H-C4B, H-C3A), 3.84 – 3.73 (m, 2H, H-C5A, H-C4A), 3.69 – 3.51 (m, 1H, OCH_2 linker), 3.47 – 3.30 (m, 1H, OCH_2 linker), 3.29 – 3.15 (m, 2H, NCH_2 linker), 2.06 (s, 3H, Ac), 2.04 (s, 3H, Ac), 1.64 – 1.44 (m, 4H, CH_2 linker), 1.36 – 1.16 (m, 2H, CH_2 linker) ppm; **^{13}C NMR** (126 MHz, $CDCl_3$) δ 170.9 (C=O Ac), 170.7 (C=O Ac), 154.4 (C=O Fmoc), 143.32 (Fmoc), 143.26 (Fmoc), 141.47 (Fmoc), 141.45 (Fmoc), 138.0 (Ar), 137.6 (Ar), 137.4 (Ar), 128.70 (Ar), 128.69 (Ar), 128.67 (Ar), 128.6 (Ar), 128.24 (Ar), 128.22 (Ar), 128.18 (Ar), 128.13 (Ar), 128.12 (Ar), 128.0 (Ar), 127.4 (Fmoc), 127.3 (Fmoc), 125.23 (Fmoc), 125.19 (Fmoc), 120.3 (Fmoc), 99.4 (d, *J* = 30.1 Hz, C1B), 97.2 (d, *J* = 28.9 Hz, C1A), 88.5 (d, *J* = 176.6 Hz, C2A), 87.0 (d, *J* = 179.3 Hz, C2B), 81.3 (d, *J* = 17.1 Hz, C3A), 76.9 (d, *J* = 16.8 Hz, C3B), 75.8 (CH_2Ph), 75.3 (CH_2Ph), 73.4 (C4A), 72.3 (C4B), 70.48 (C5B), 70.45 (CH_2 Fmoc), 69.8 (C5A), 68.2 (OCH_2 linker), 67.3 (CH_2 Cbz linker), 62.9 (C6A), 62.8 (C6B), 50.7 (CH_2 NBn), 50.4 (CH_2 NBn), 47.2 (NCH_2 linker), 46.8 (Fmoc CH), 46.2 (NCH_2 linker), 29.1 (CH_2 linker), 28.0 (CH_2 linker), 27.6 (CH_2 linker), 23.4 (CH_2 linker), 21.0 (Ac), 20.9 (Ac) ppm; **^{19}F NMR** (470 MHz, $CDCl_3$) δ -203.45 (ddd, *J* = 48.6, 30.1, 7.0 Hz), -203.68 (ddd, *J* = 50.3, 28.7, 6.9 Hz) ppm; **HRMS** (ESI) m/z $[M+Na]^+$ calcd for $C_{65}H_{69}F_2NO_{15}Na^+$ 1164.4533; found 1164.4524.

Side Product (S24)

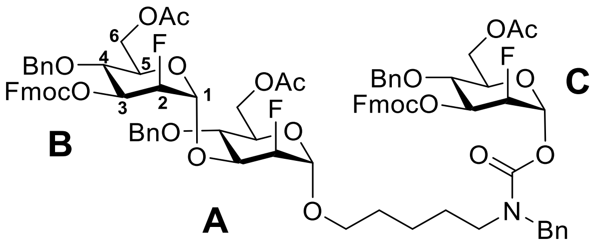

The C3C minor rotamer signal is obscured by chloroform in the ^{13}C NMR spectrum.

R$_f$ (CyH/EtOAc 7/3) 0.17; **^{1}H NMR** (500 MHz, $CDCl_3$) δ 7.80 – 7.74 (m, 4H, Fmoc), 7.65 – 7.57 (m, 4H, Fmoc), 7.44 – 7.38 (m, 4H, Fmoc), 7.36 – 7.16 (m, 24H, Ar), 6.31 (dd, *J* = 6.1, 1.6 Hz, 0.33H, H-C1C minor), 6.25 (dd, *J* = 6.5, 1.9 Hz, 0.66H, H-C1C major), 5.28 – 5.16 (m, 2H, H-C3B, H-C1B), 5.04 (dd, *J* = 27.9, 9.8 Hz, 0.33H, H-C3C minor), 4.98 – 4.67 (m, 7.66H, C*H$_2$*Ph OBn, H-C3C major, H-C1A, H-C2A, H-C2B, H-C2C), 4.66 – 4.23 (m, 15.66, CH Fmoc, CH_2 Fmoc, H-C6C minor, H-C6A, H-C6B, C*H$_2$*Ph NBn, C*H$_2$*Ph OBn), 4.23 – 4.17 (m, 1H, H-C5B), 4.16 – 3.88 (m, 4.64H, H-C4C, H-C5C minor, H-C4B, H-C3A, H-C6C major), 3.84 – 3.71 (m, 2H, H-C4A, H-C5A), 3.69 – 3.61 (m, 0.66H, OCH_2 linker major), 3.61 – 3.47 (m, 1.65H, OCH_2 linker minor, OCH_2 linker major, NCH_2 linker major), 3.45 – 3.36 (m, 0.66H, OCH_2 linker major), 3.37 – 3.30 (m, 0.33H, OCH_2 linker minor), 3.28 – 3.14 (m, 1.99H, NCH_2 linker, H-C5C major), 3.13 – 3.03 (m, 0.33H, NCH_2 linker minor), 2.06 (s, 3H, Ac), 2.04 (s, 3H, Ac), 2.04 (s, 3H, Ac), 1.63 – 1.45 (m, 4H, CH_2 linker), 1.36 – 1.20 (m, 2H, CH_2 linker) ppm; **^{13}C NMR** (126 MHz, $CDCl_3$) δ 170.9 (C=O Ac), 170.73 (C=O Ac), 170.71 (C=O Ac), 154.4 (C=O Fmoc), 153.2 (NC=O), 143.32 (Fmoc), 143.30 (Fmoc), 143.26 (Fmoc), 143.2 (Fmoc), 141.46 (Fmoc), 141.45 (Fmoc), 137.63 (Bn), 137.59 (Bn), 137.5 (Bn), 137.4 (Bn), 137.2 (Bn), 137.1 (Bn), 129.2 (Ar), 128.9 (Ar), 128.69 (Ar), 128.66 (Ar), 128.6 (Ar), 128.40 (Ar), 128.36 (Ar), 128.22 (Ar), 128.19 (Ar), 128.15 (Ar), 128.13 (Ar), 128.11 (Ar), 128.06 (Ar), 128.0 (Ar), 127.9 (Ar), 127.7 (Ar), 127.37 (Ar), 127.35 (Ar), 126.6 (Ar), 125.23 (Fmoc), 125.19 (Fmoc), 125.18 (Fmoc), 125.1 (Fmoc), 120.28 (Fmoc), 120.26 (Fmoc), 99.4 (d, *J* = 29.1 Hz, C1B major), 99.3 (d, *J* = 28.7 Hz, C1B minor), 97.2 (d, *J* = 28.6 Hz, C1A major), 97.1 (d, *J* = 29.2 Hz, C1A minor), 91.6 (d, *J* = 32.0 Hz, C1C minor), 91.3 (d, *J* = 31.4 Hz, C1C major), 88.5 (d, *J* = 178.0 Hz, C2A major), 88.4 (d, *J* = 178.0 Hz, C2A minor), 87.0 (d, *J* = 179.5 Hz, C2B), 86.40 (d, *J* = 180.2 Hz, C2C major), 86.35 (d, *J* = 179.3 Hz, C2C minor), 81.3 (d, *J* = 17.4 Hz, C3A), 76.9 (d, *J* = 16.9 Hz, C3B), 76.7 (d, *J* = 16.9 Hz, C3C major), 75.8 (CH_2Ph OBn), 75.3 (CH_2Ph OBn), 75.1 (CH_2Ph OBn), 73.4 (C4A), 72.3 (C4B), 72.1 (C5C minor), 71.7 (C4C minor), 71.6 (C4C major, C5C major), 70.5 (CH_2 Fmoc), 70.44 (C5B), 69.8 (C5A), 68.2 (OCH_2 linker major), 68.1 (OCH_2 linker minor), 62.91 (C6A/B major), 62.85 (C6A/B/C minor), 62.8 (C6A/B major), 62.7 (C6A/B/C minor), 62.5 (C6A/B/C minor), 62.3 (C6C major), 51.3 (CH_2Ph NBn major), 51.2 (CH_2Ph NBn minor), 48.8 (NCH_2 linker major), 46.82 (NCH_2 linker minor), 46.77 (CH Fmoc), 29.2 (CH_2 linker), 29.1 (CH_2 linker), 27.8 (CH_2 linker), 27.1 (CH_2 linker), 23.6 (CH_2 linker), 23.5 (CH_2 linker), 21.0 (Ac), 20.92 (Ac), 20.88 (Ac) ppm; **^{19}F NMR** (470 MHz, $CDCl_3$) δ -203.34 – -203.93 (m) ppm; **^{19}F{^{1}H} NMR** (470 MHz, $CDCl_3$) δ -203.46 (F-C2A minor), -203.55 (F-C2A major), -203.66 (F-C2B minor), -203.69 (F-C2B major), -203.71 (F-C2C major), -203.82 (F-C2C minor) ppm; **HRMS** (ESI) m/z $[M+Na]^+$ calcd for $C_{88}H_{90}F_3NO_{22}Na^+$ 1592.5804; found 1592.5794.

BioNMR: Materials and Methods, and SAR

NMR reporter assay

NMR samples contained 13 µM FNF **35**, 7.5 µM ConA (InvivoGen CONA_CANCT p81461, lot 9847-46-02), PBS buffer pH 7.4 (Gibco, 10010-015), 10% D_2O and 130 µM TFE. Spectra were recorded at 298 K on an AVIIIHD 600 MHz spectrometer (Bruker) equipped with a QCI-F CryoProbe™ (Bruker) using a ^{19}F pulse-acquire experiment with ^{1}H decoupling. 512 scans were acquired in 22 min for each spectrum and the resulting FIDs were processed with exponential apodization in the Topspin software (TS 4.0.7 Bruker). Signal intensities of the FNF reporter were normalized against TFE and corrected for dilution and for a slight increase of the signal after addition of competitor solution. No correction was performed for the possible non-linear displacement response of the reporter. IC_{50} values were fitted in with python using the familiar four-parameter equation assuming a cooperativity value of n = 1.

$$S = S_{\min} + \frac{S_{\max} - S_{\min}}{1 + \left(\frac{IC_{50}}{[I]}\right)^{n}}$$

Signal intensities S for minimal and maximal displacement values were derived from the data for free FNF (S_{max}) and FNF in presence of ConA (S_{min}). [I] denotes the concentration of inhibitor. The only parameter fitted was the IC_{50}.

K_D determination by isothermal titration calorimetry

ITC experiments were performed on a PEAQ-ITC instrument (Microcal). Concanavalin A (2.3 mg) (InvivoGen CONA_CANCT p81461, lot 9847-46-02) was dissolved in 1 ml PBS (phosphate buffer saline, pH 7.4, gibco 10010-015) and dialysed at room temperature for 2 hours agains ca. 100 ml of PBS buffer using a 3.5 kDA MWCO dialysis membrane (Spectra/Por 132720). The buffer was exchanged once and the sample was dialyzed for another 2 hours. The Concanavalin A concentration was determined by absorbance measurements at 280 nm (MW = 25539.4, theoretical A_{280} = 1.27 for 1 g/l). After dialysis, he Concanavalin A solution was diluted with dialysis buffer to a concentration of 40 µM (momomer) for the ITC experiments.

The stock solution of ligand FNF **35** (in D_2O) was lyophilized for 15 h and subsequently dissolved in dialysis buffer. The concentration of FNF (c(FNF) = 325 µM) was determined by ^{1}H NMR by integrating signals at 2.933 ppm (2H, t), 1.614 ppm and 0.262 ppm (4H, m + t) and 0.126 ppm (2H, t) and referencing to the signal integral of DSS (1000 uM ^{1}H concentration).

ITC experiments were performed with 19 injections of 2.5 ul every 150 s at 25 °C. Raw data were integrated, normalized for the molar concentration, and analyzed using Origin software, according to a 1:1 ConA:FNF ratio binding model. The concentration of Concanavalin A was also fitted to the data, while keeping the ligand concentration at the one determined by NMR and the number of binding sites fixed at N = 1.

The fitting result were:
K_D = 3.83 +/- 0.627 µM,
ΔH = -42.0 +/- 2.18 kJ / mol,
-T ΔS = 11.1 kJ / mol,
ΔG = -30.9 kJ / mol,
Concentration(Concancavalin A) = 35.3 µM

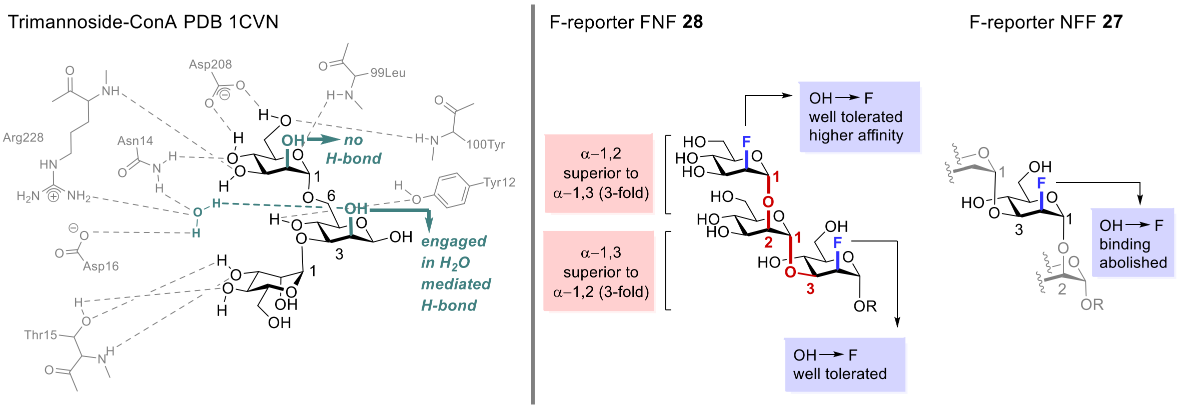

Figure S3. Graphical representation of structure-activity-relationship of the studied trimannosides. (A) interaction network of the branched trimannoside reported in PDB entry 1I3H (J. H. Naismith, R. A. Field, J. Biol. Chem. 1996, 271, 972–976.) and (B) learnings from different affinities of the studied trimannosides.

NMR Spectra

^{1}H NMR, 599 MHz, $CDCl_3$ – Compound 1

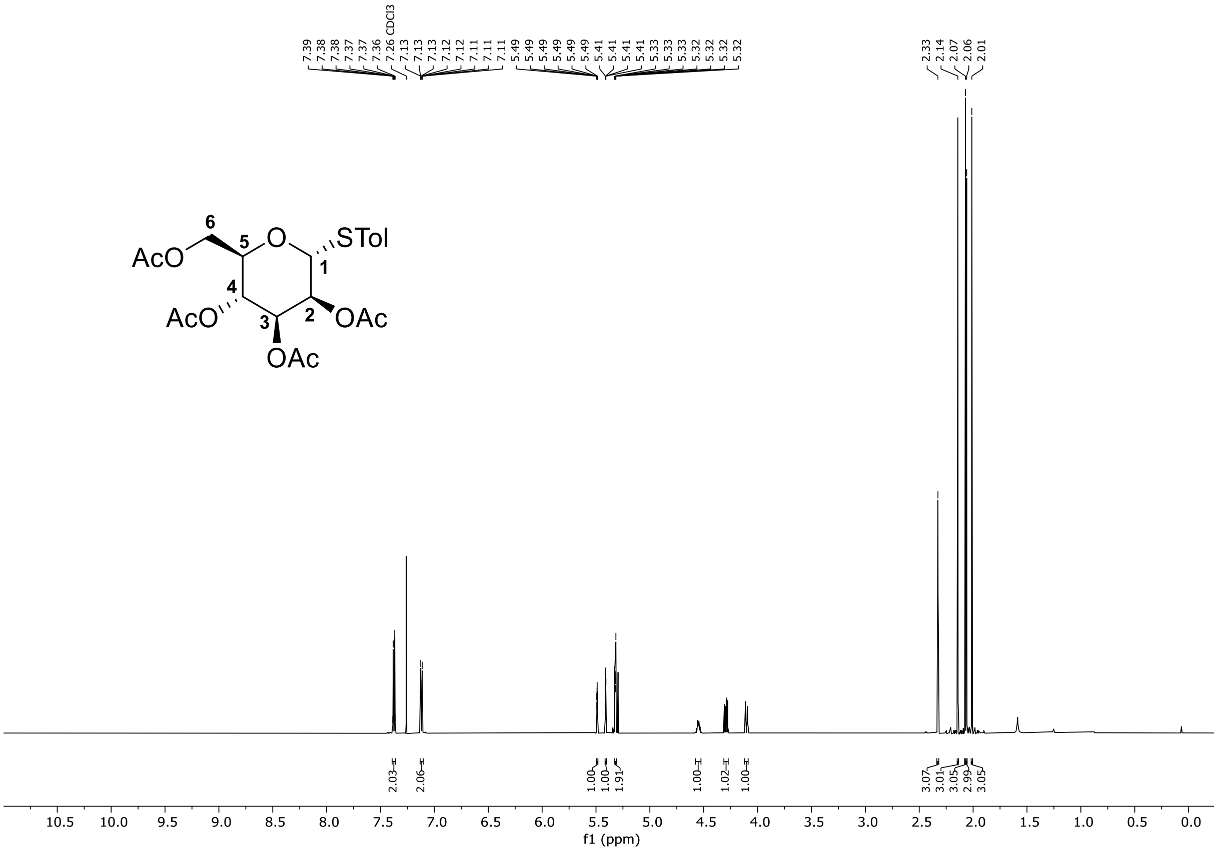

^{13}C NMR, 151 MHz, $CDCl_3$ – Compound 1

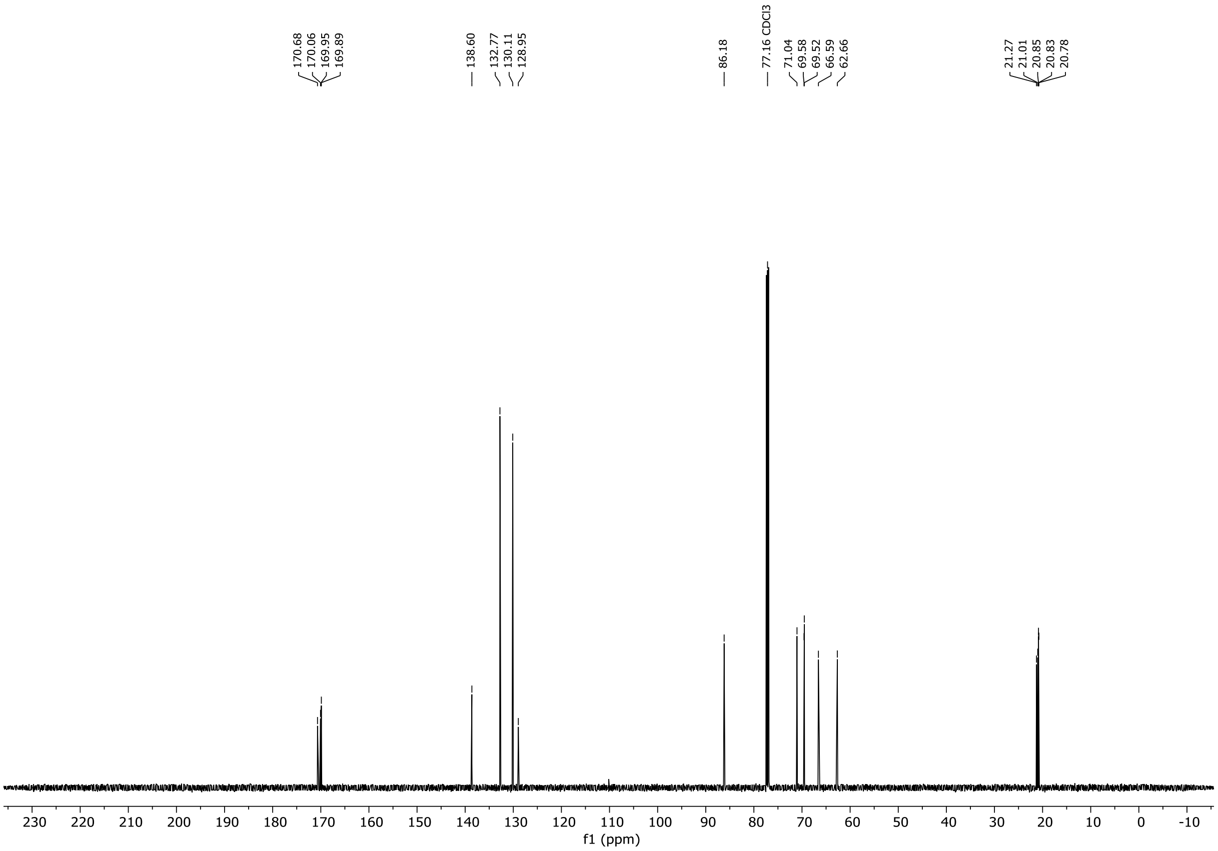

^{1}H NMR, 599 MHz, CD_2Cl_2 – Compound S1

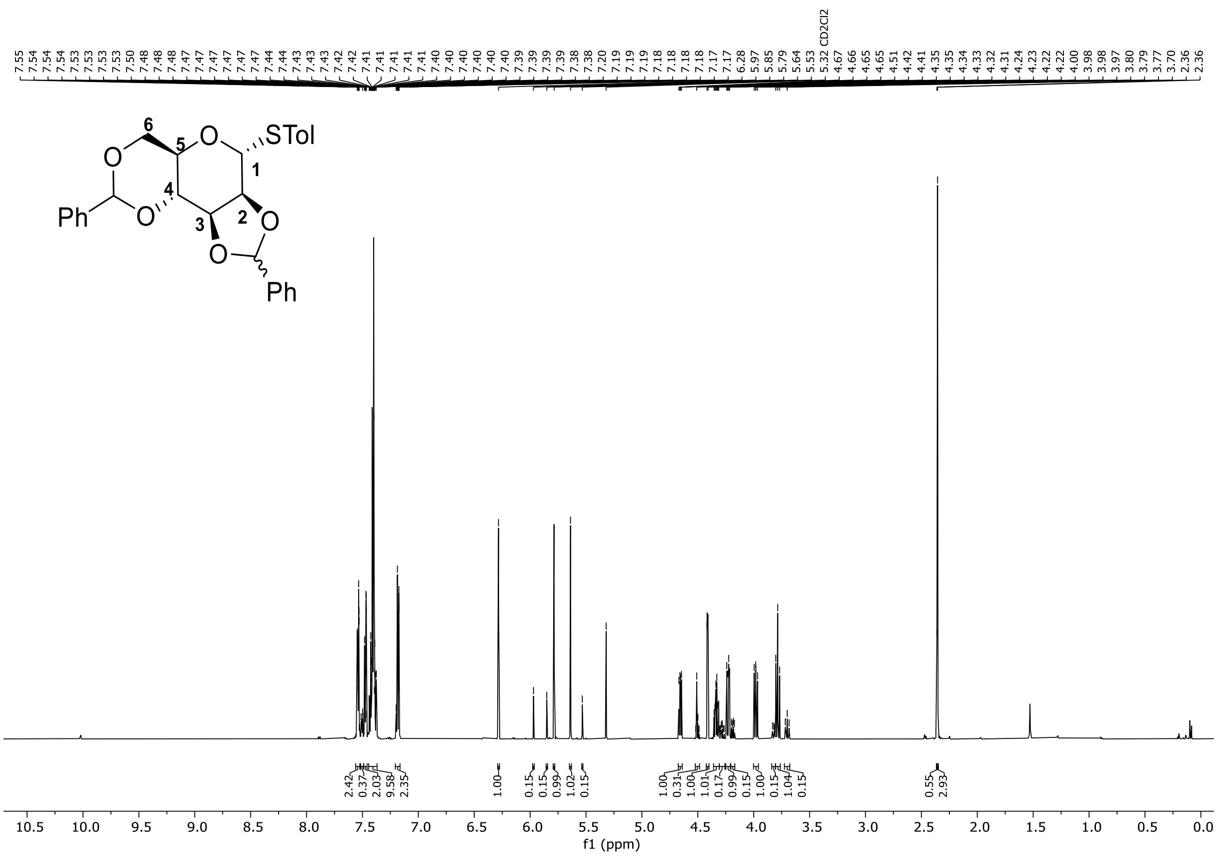

^{13}C NMR, 151 MHz, CD_2Cl_2 – Compound S1

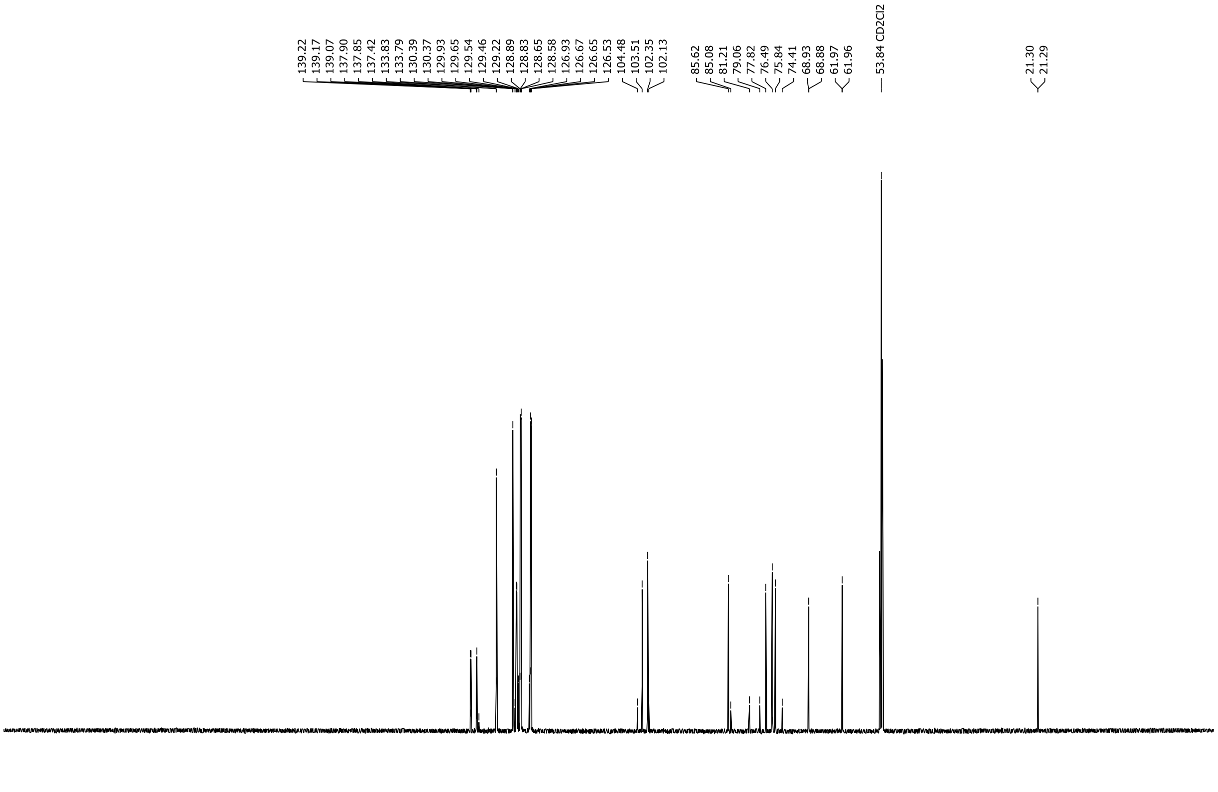

^{1}H NMR, 500 MHz, CD_2Cl_2 – Compound S2

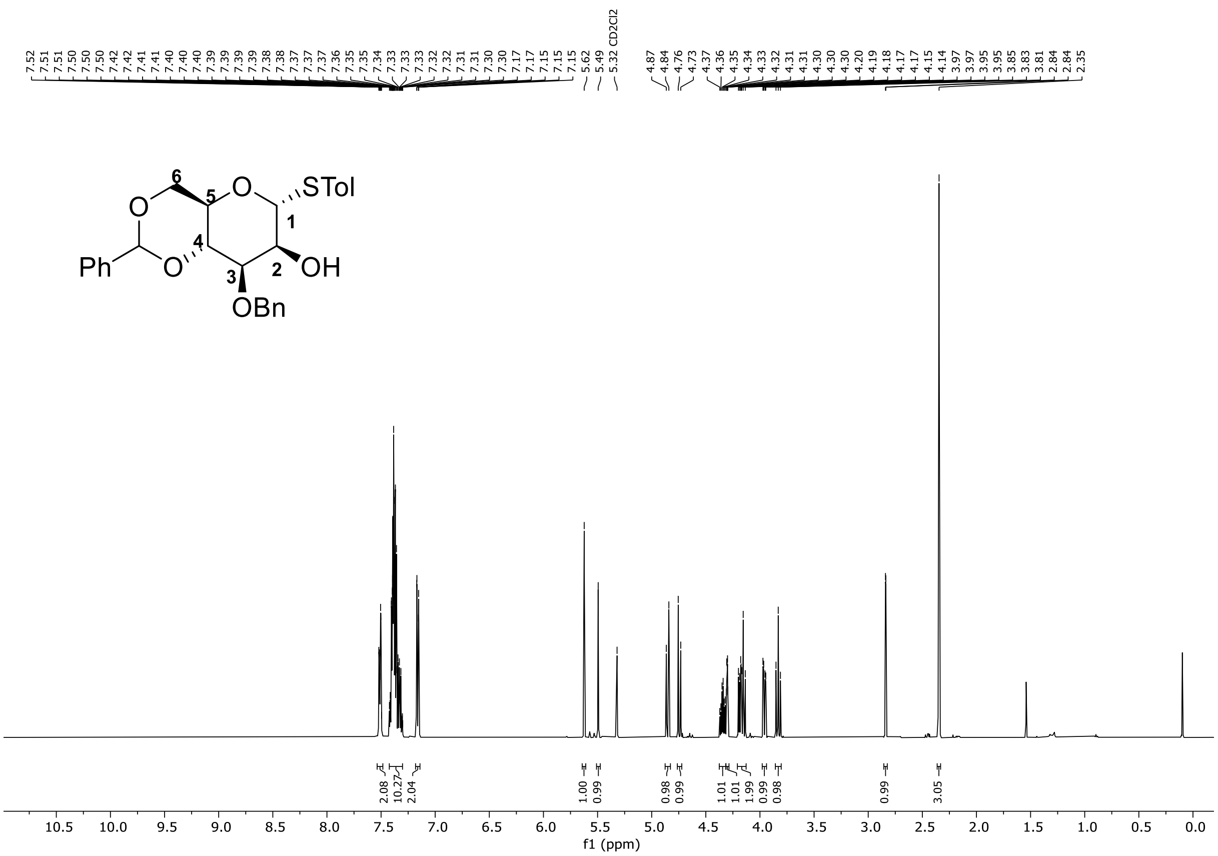

^{13}C NMR, 126 MHz, CD_2Cl_2 – Compound S2

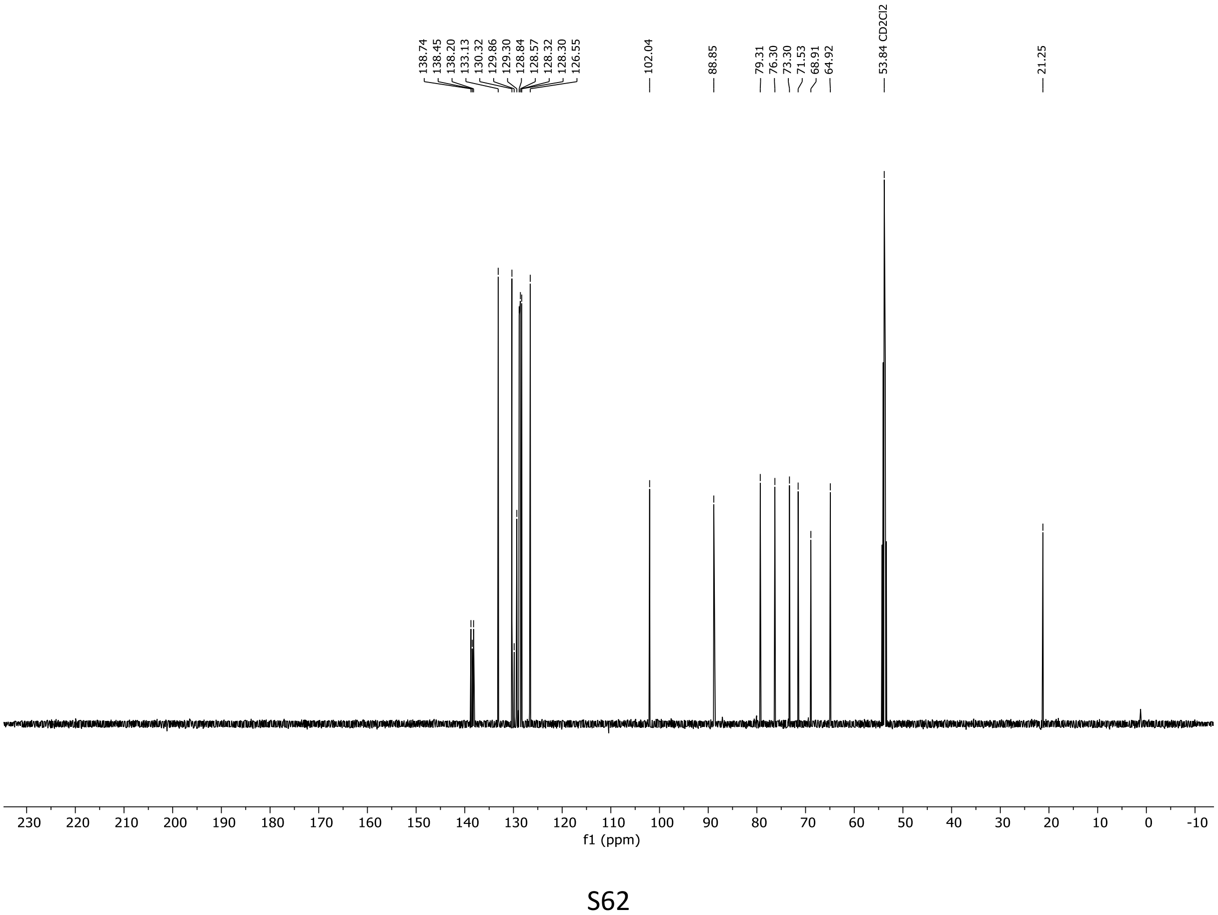

^{1}H NMR, 599 MHz, CD_2Cl_2 – Compound S3

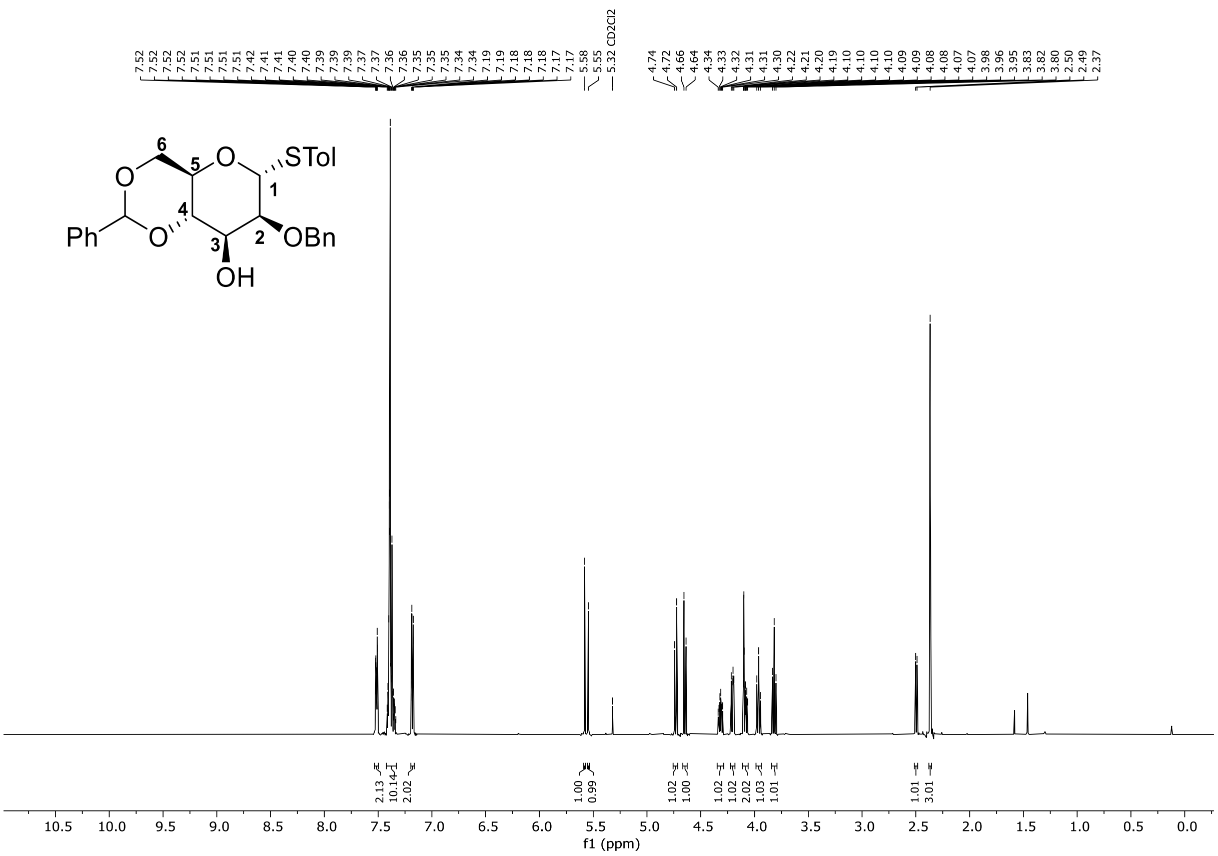

^{13}C NMR, 151 MHz, CD_2Cl_2 – Compound S3

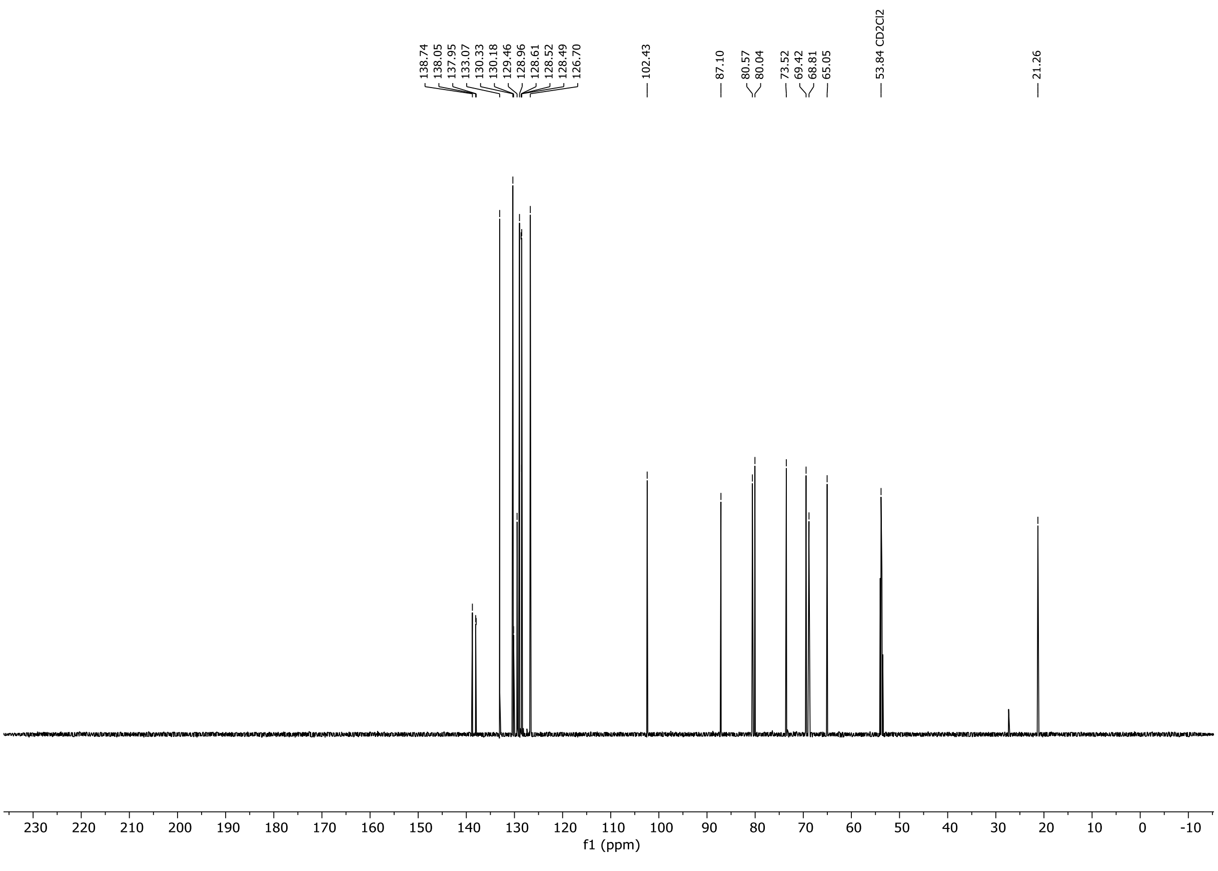

^{1}H NMR, 599 MHz, CD_2Cl_2 – Compound S4

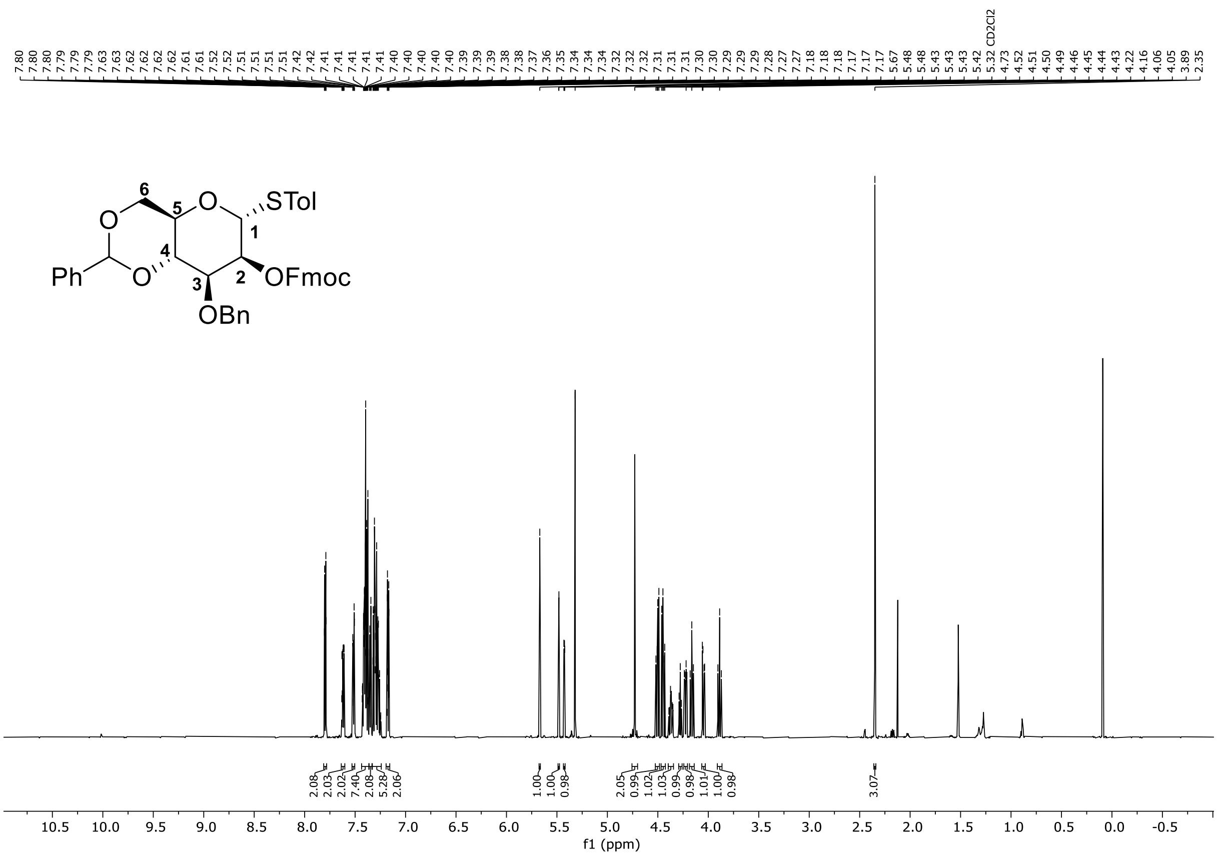

^{13}C NMR, 151 MHz, CD_2Cl_2 – Compound S4

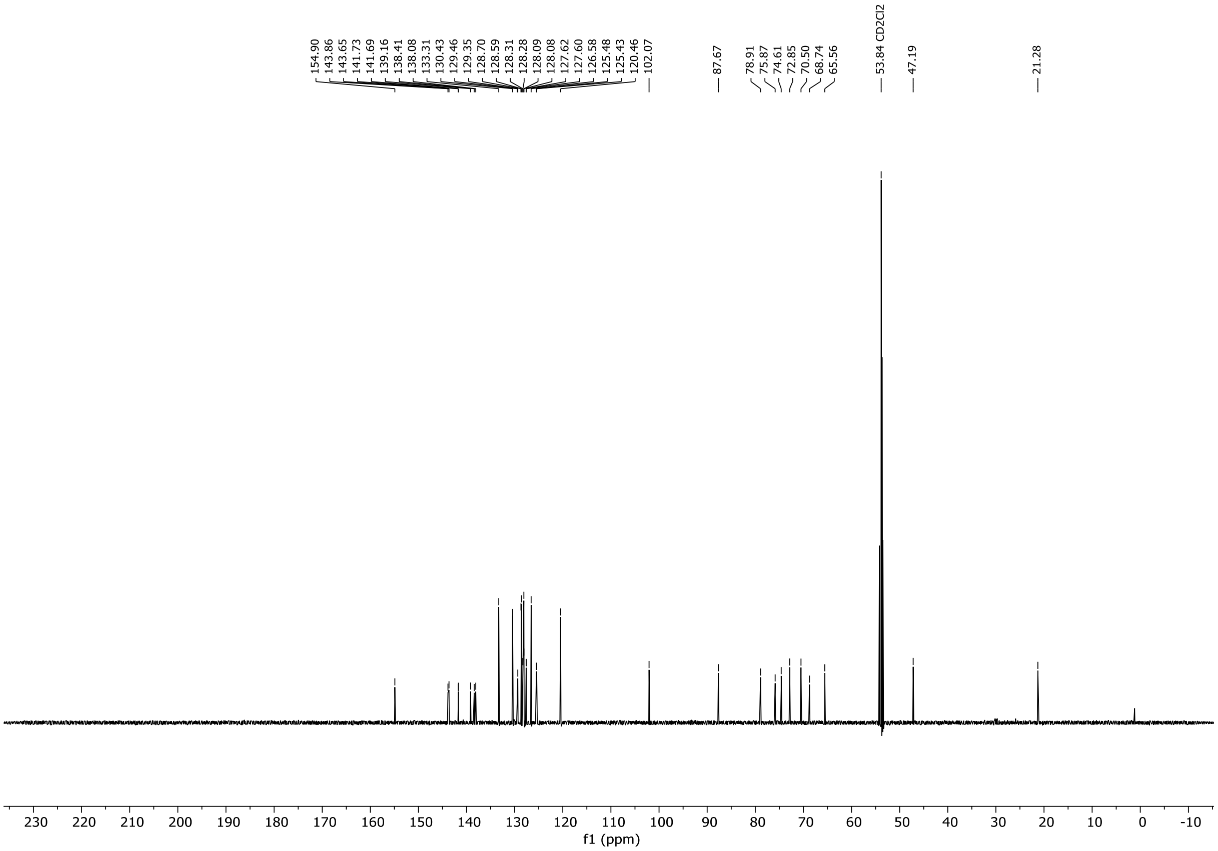

^1H NMR, 599 MHz, $CDCl_3$ – Compound S5

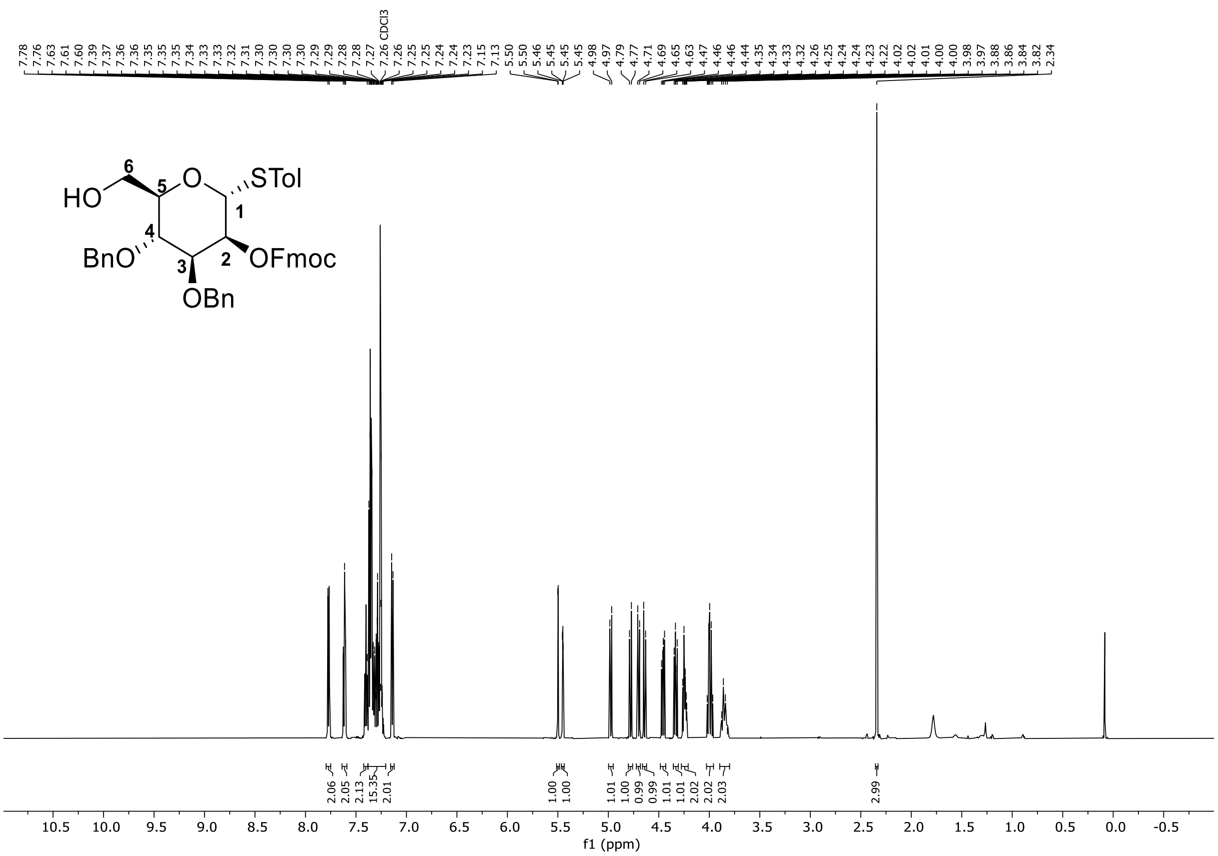

^{13}C NMR, 151 MHz, $CDCl_3$ – Compound S5

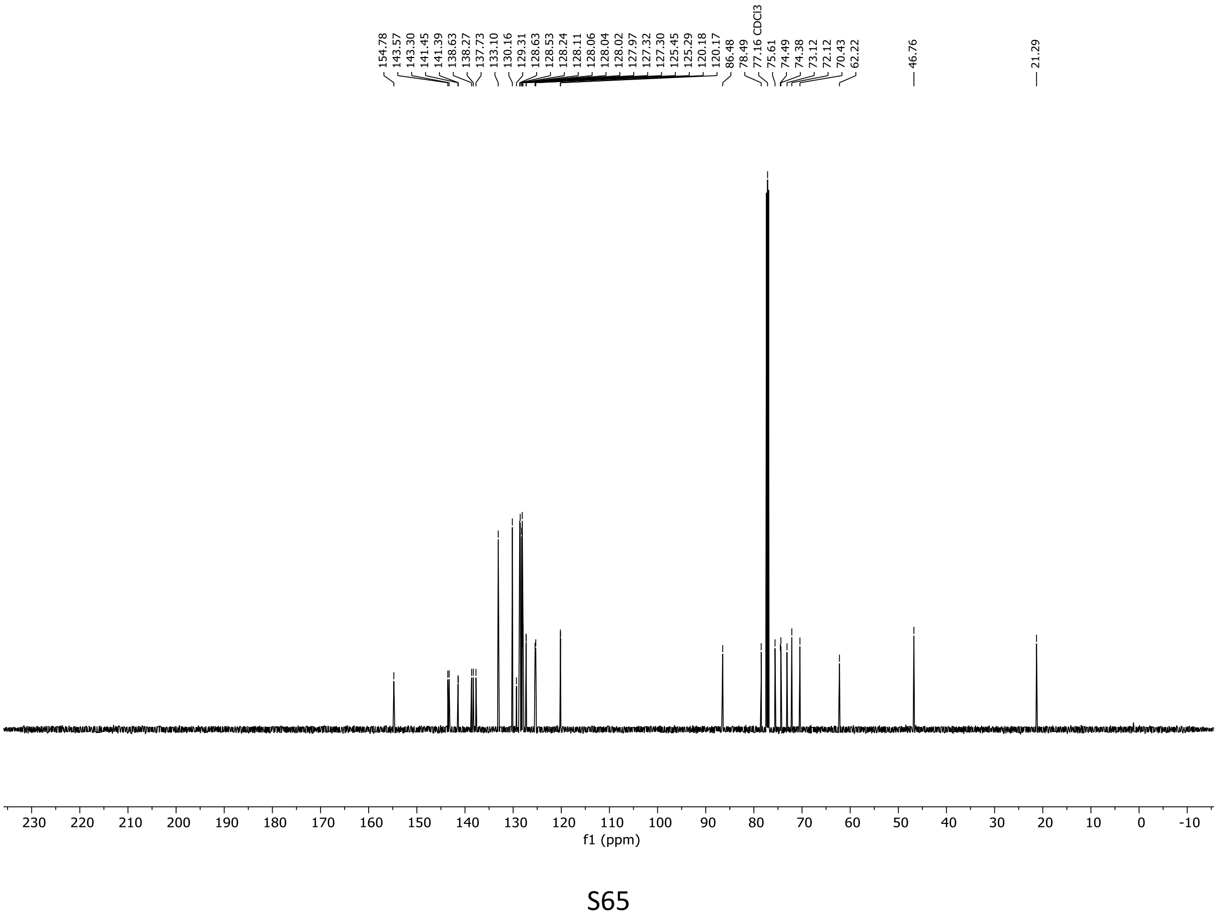

^{1}H NMR, 599 MHz, $CDCl_3$ – Compound 9

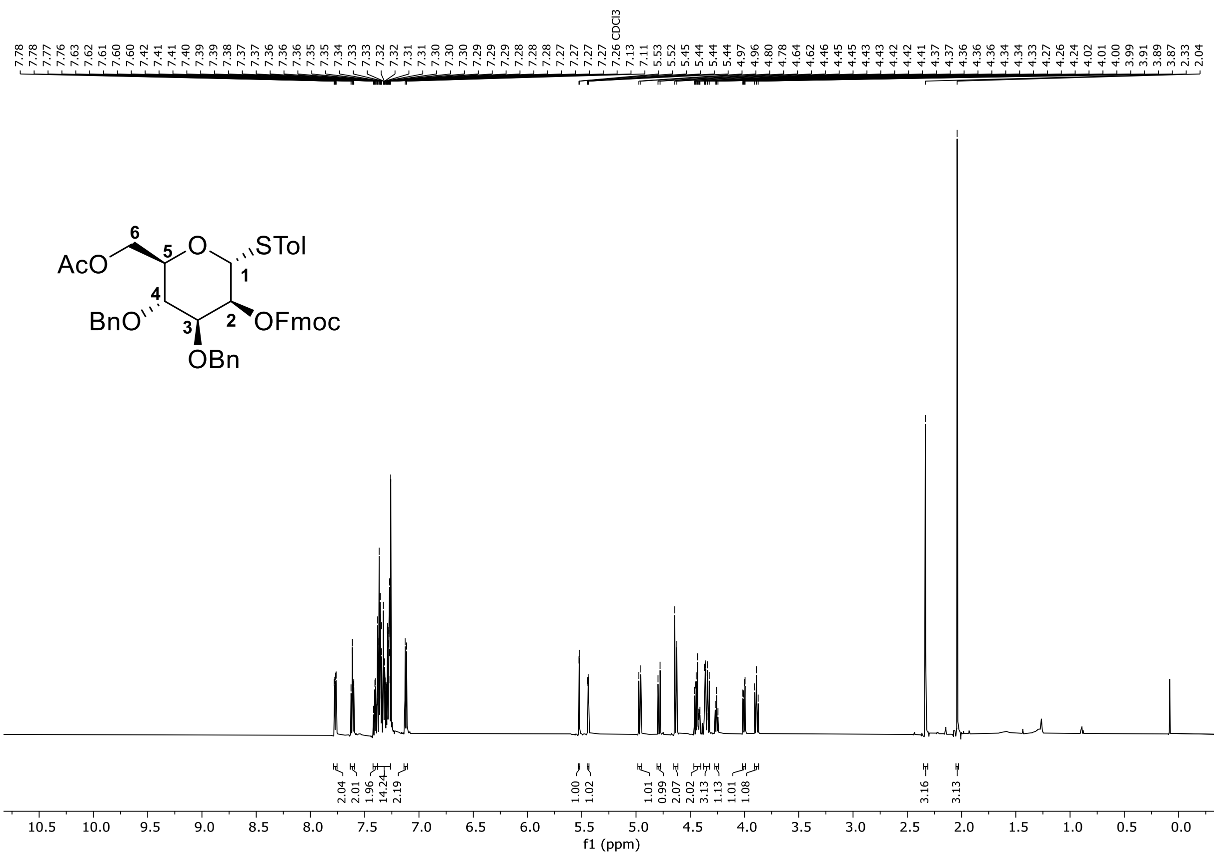

^{13}C NMR, 151 MHz, $CDCl_3$ – Compound 9

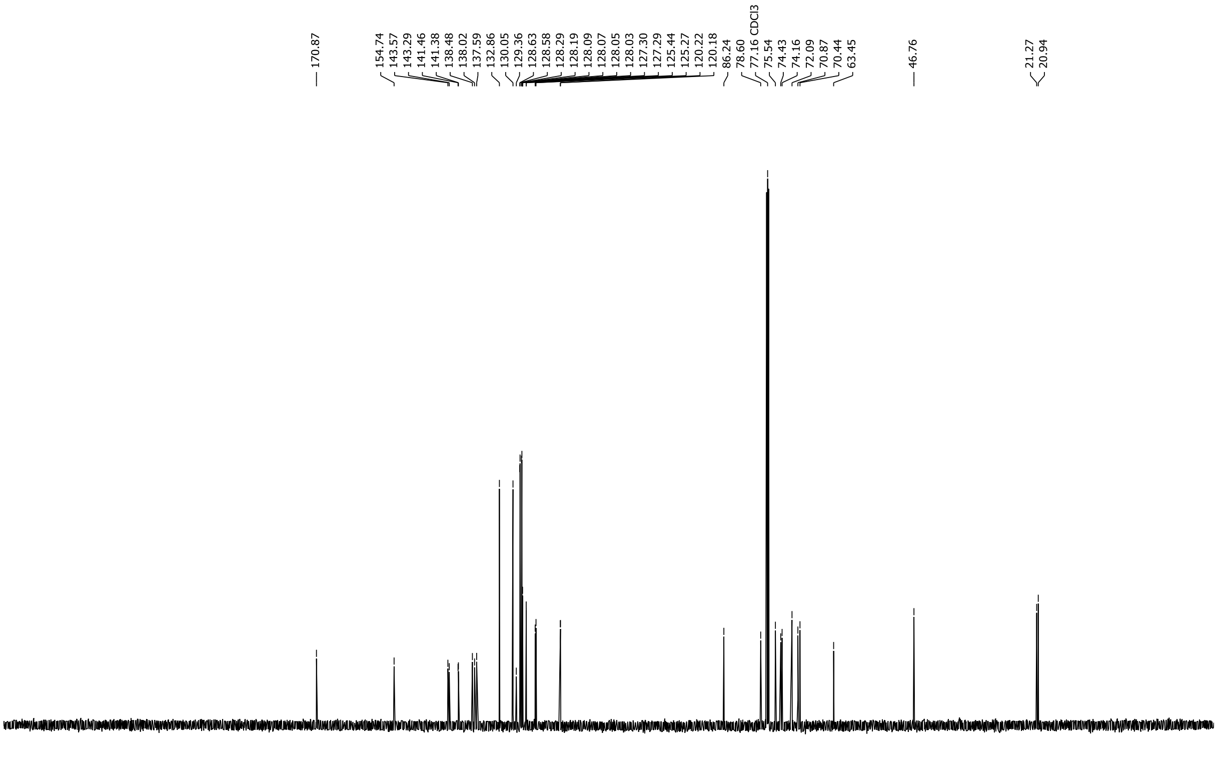

^{1}H NMR, 500 MHz, CD_3OD – Compound 2

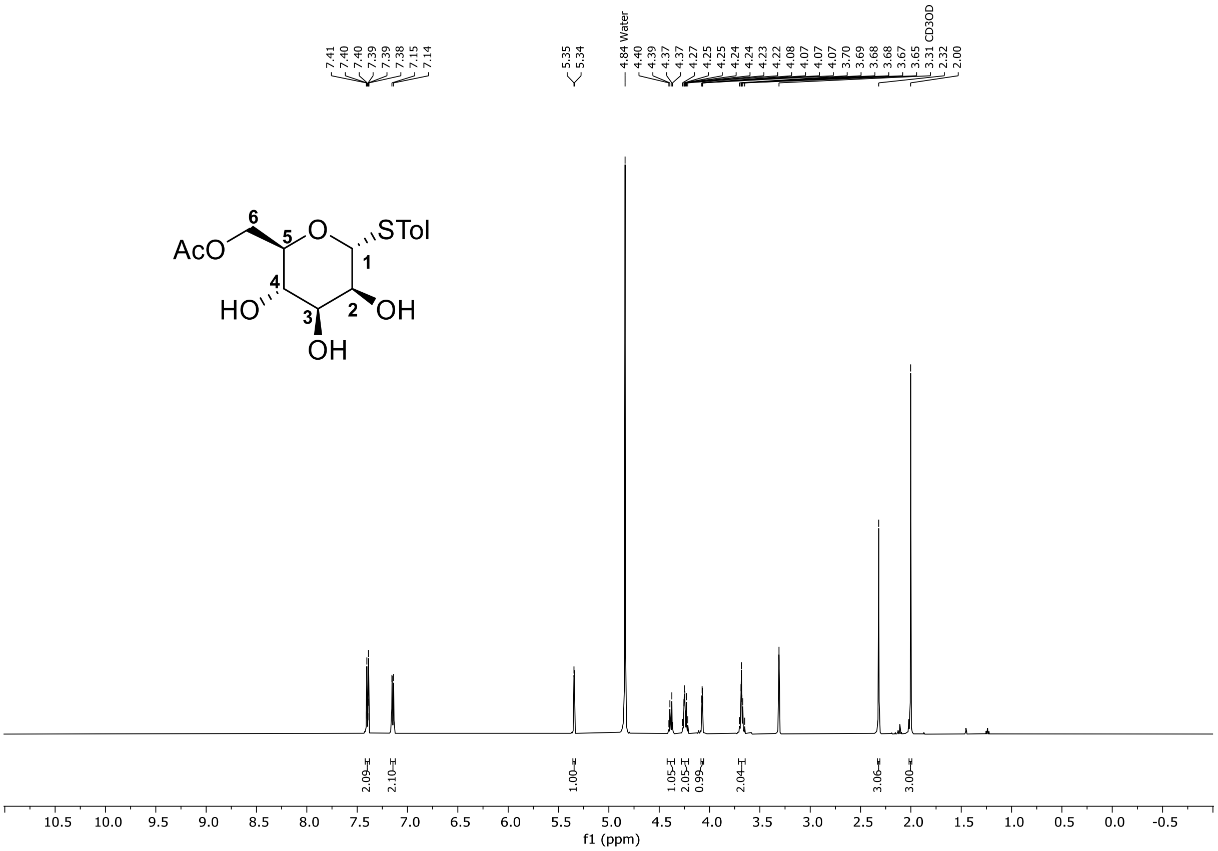

^{13}C NMR, 126 MHz, CD_3OD – Compound 2

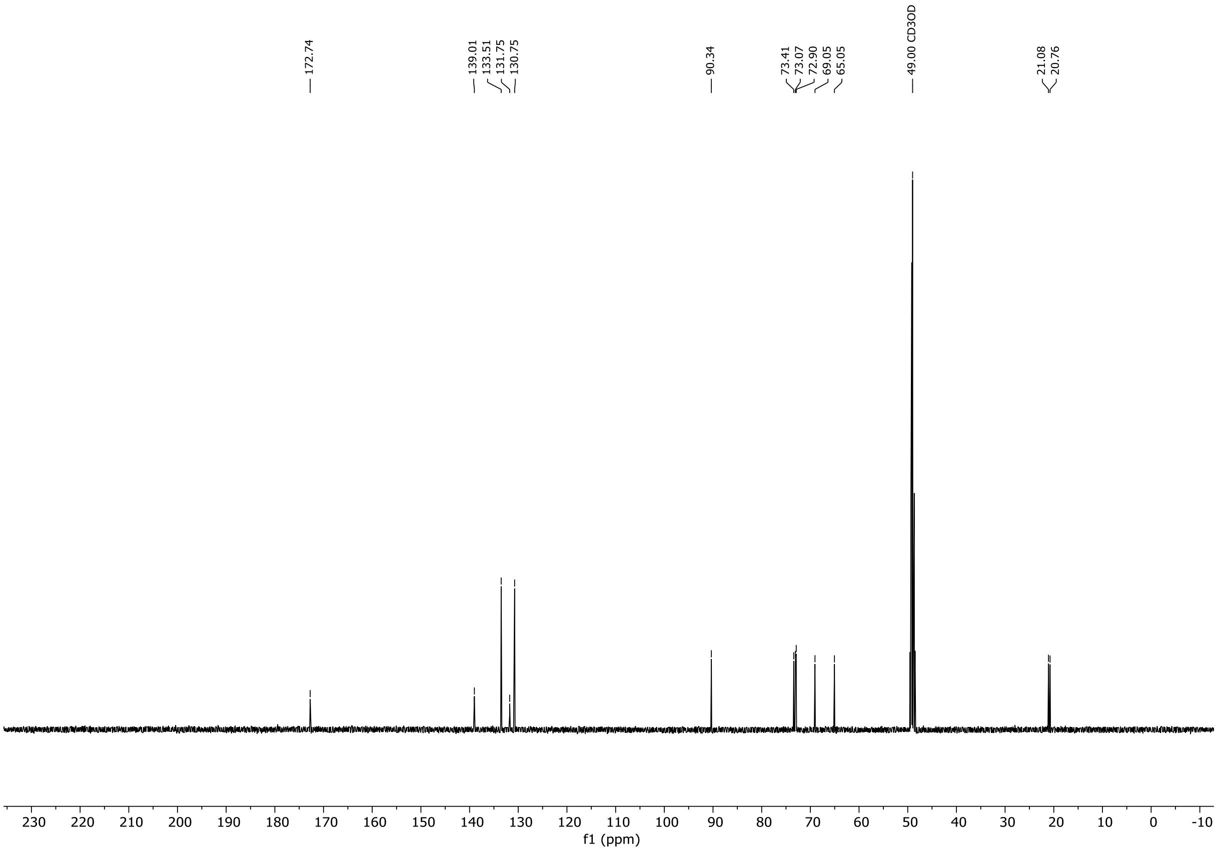

^{1}H NMR, 500 MHz, CDCl$_3$ – Compound 3

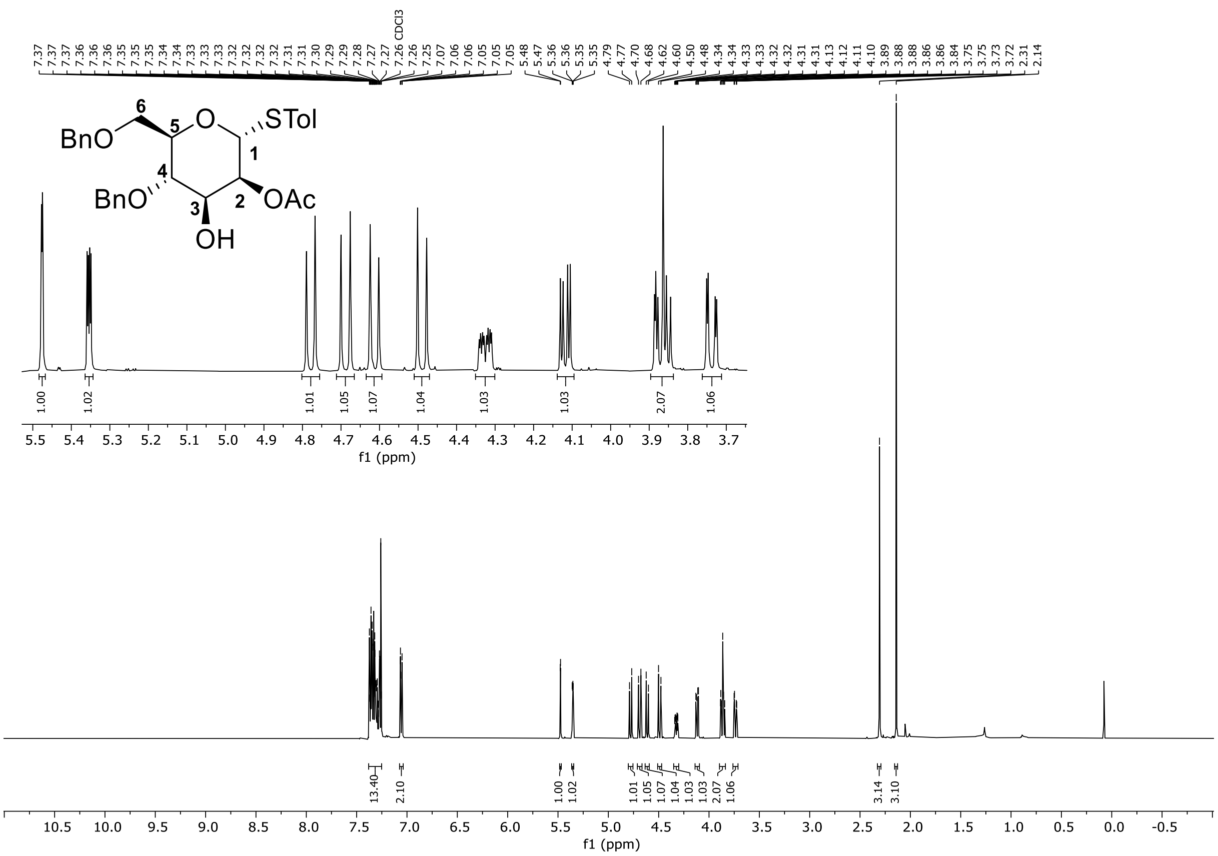

^{13}C NMR, 126 MHz, CDCl$_3$ – Compound 3

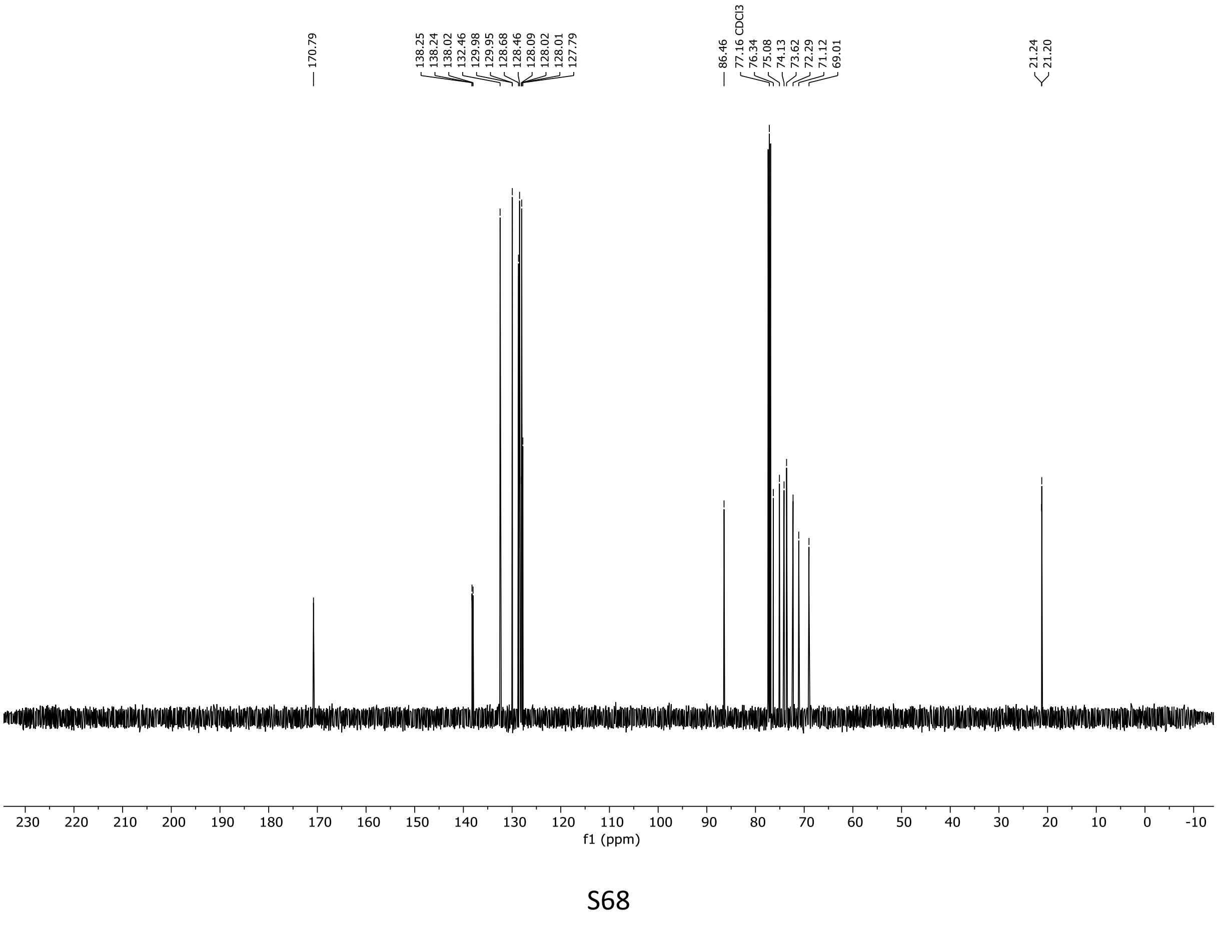

^{1}H NMR, 500 MHz, $CDCl_3$ – Compound 4

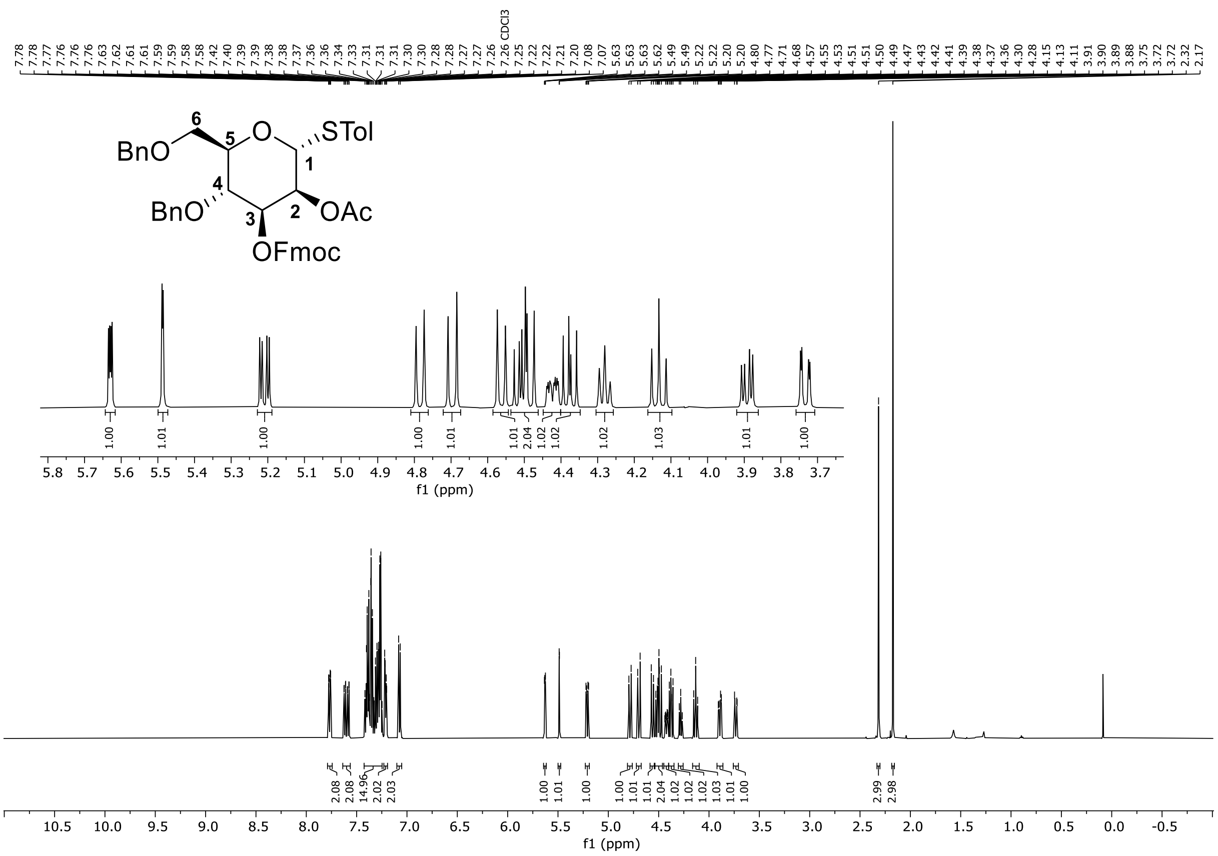

^{13}C NMR, 126 MHz, $CDCl_3$ – Compound 4

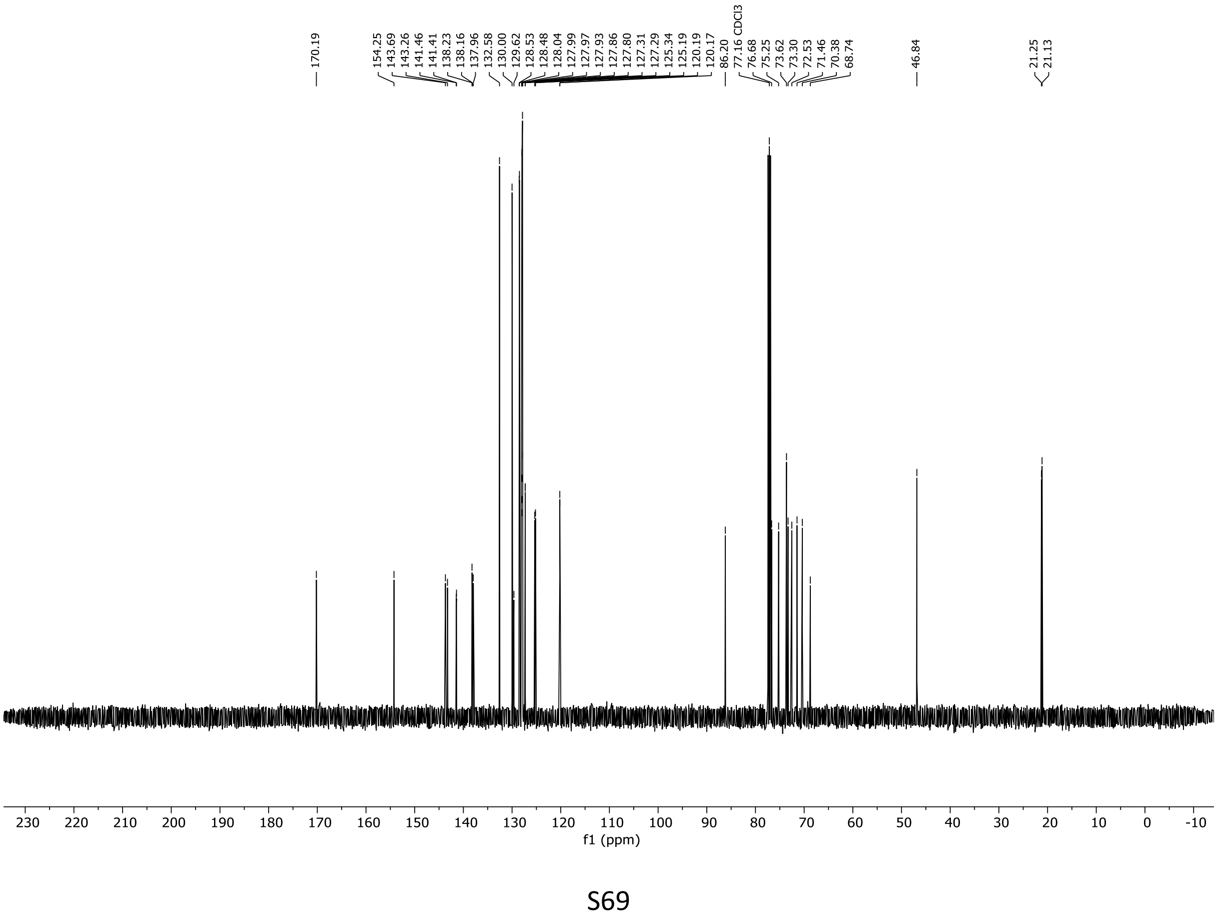

^{1}H NMR, 599 MHz, $CDCl_3$ – Compound S6

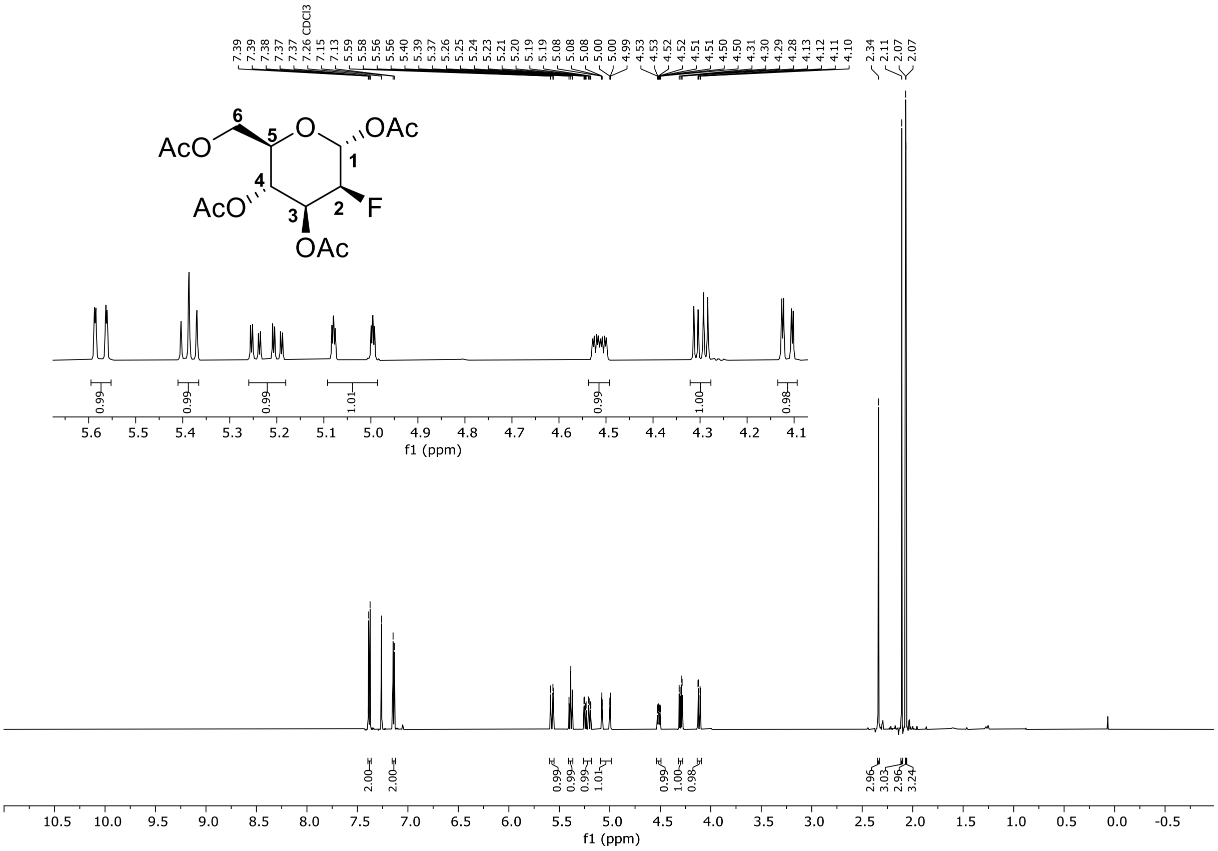

^{13}C NMR, 151 MHz, $CDCl_3$ – Compound S6

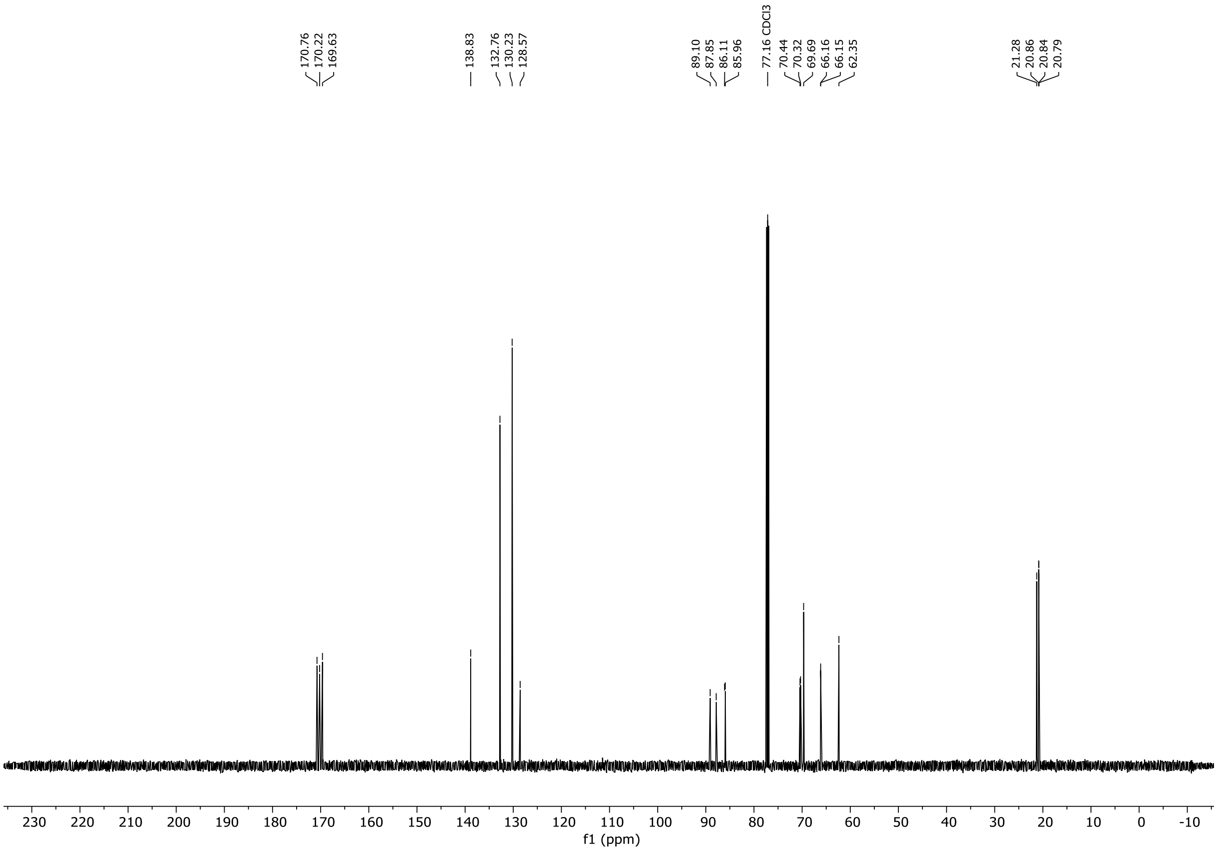

^{19}F NMR, 564 MHz, $CDCl_3$ – Compound S6

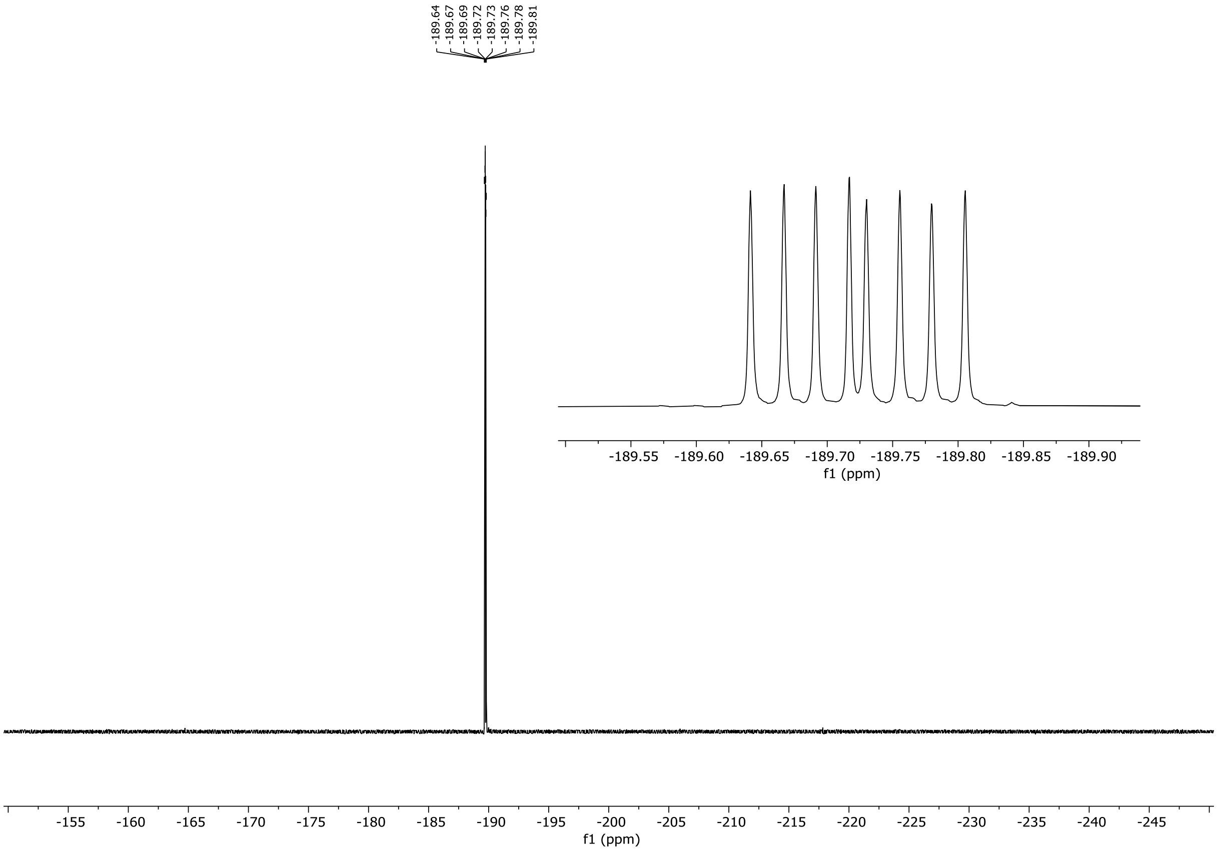

^{1}H NMR, 500 MHz, $CDCl_3$ – Compound S7

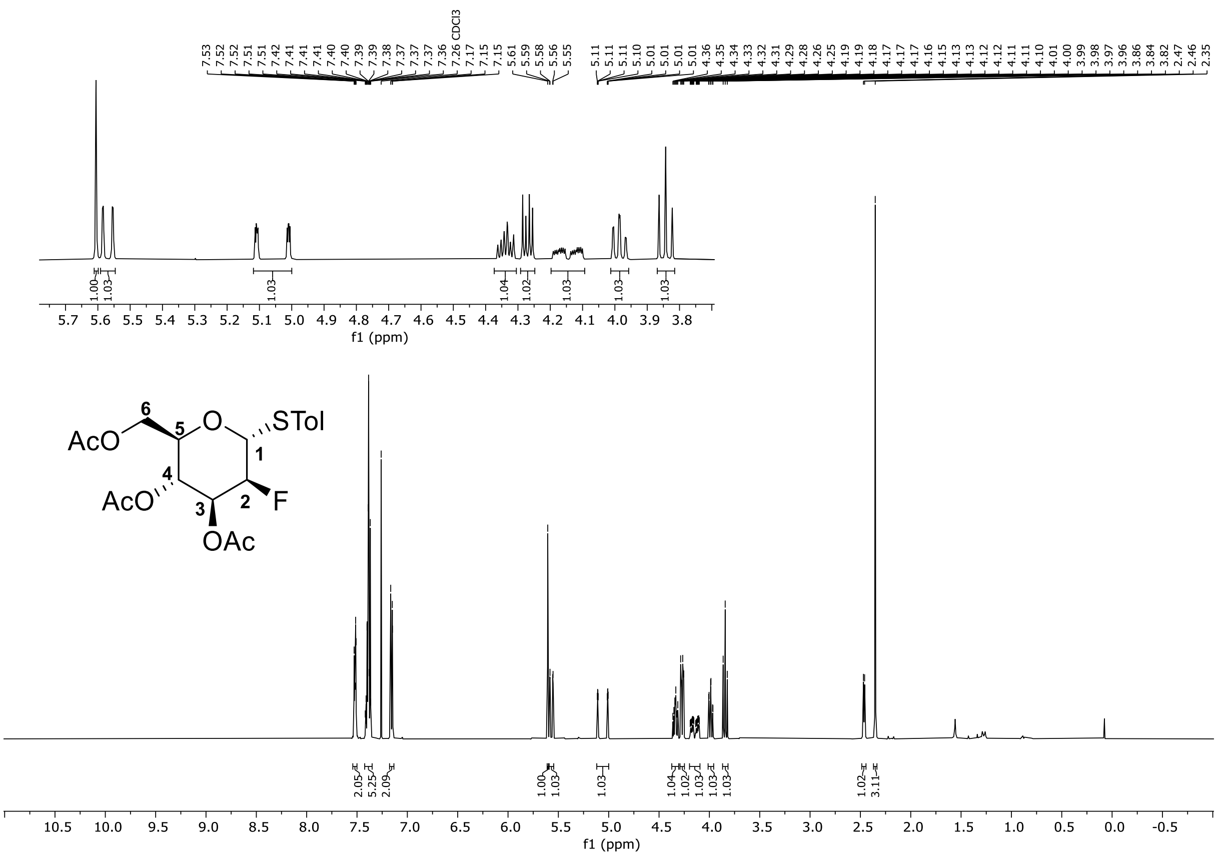

^{13}C NMR, 126 MHz, $CDCl_3$ – Compound S7

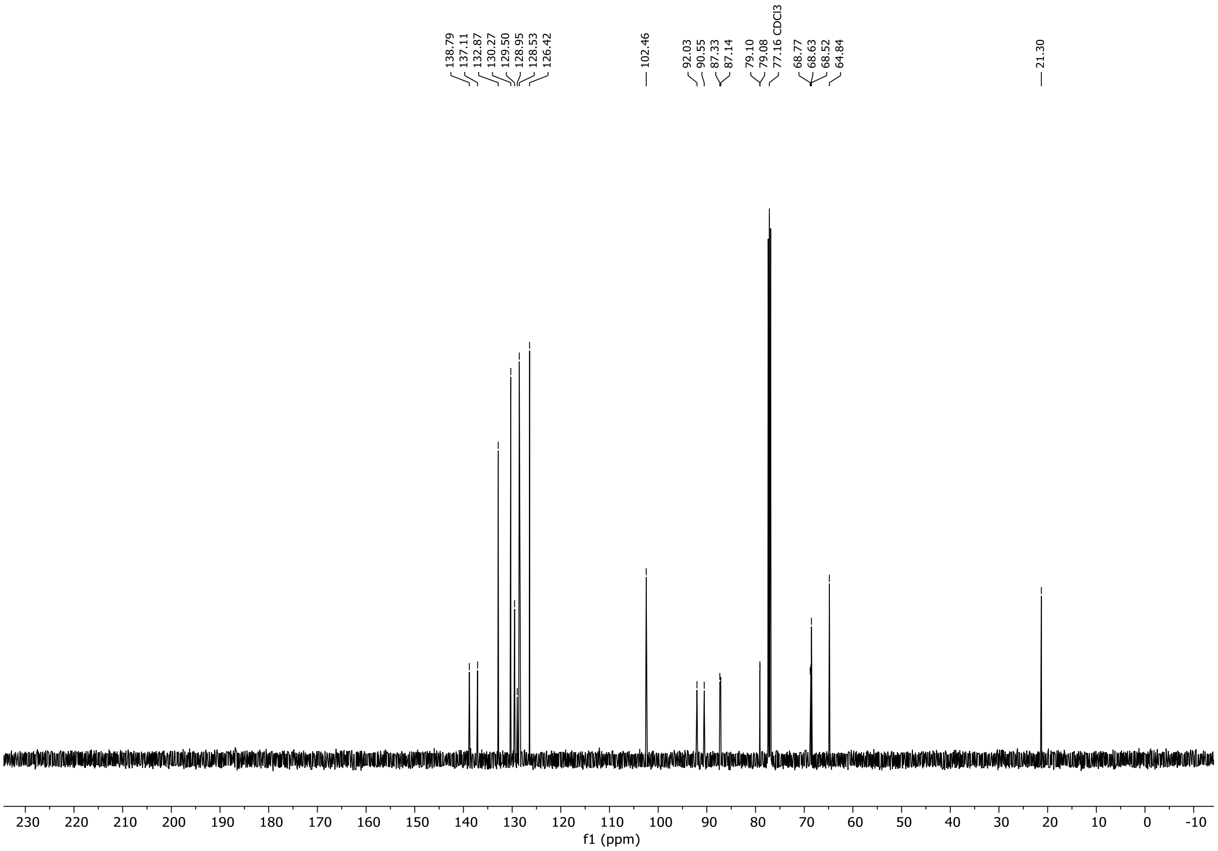

^{19}F NMR, 470 MHz, $CDCl_3$ – Compound S7

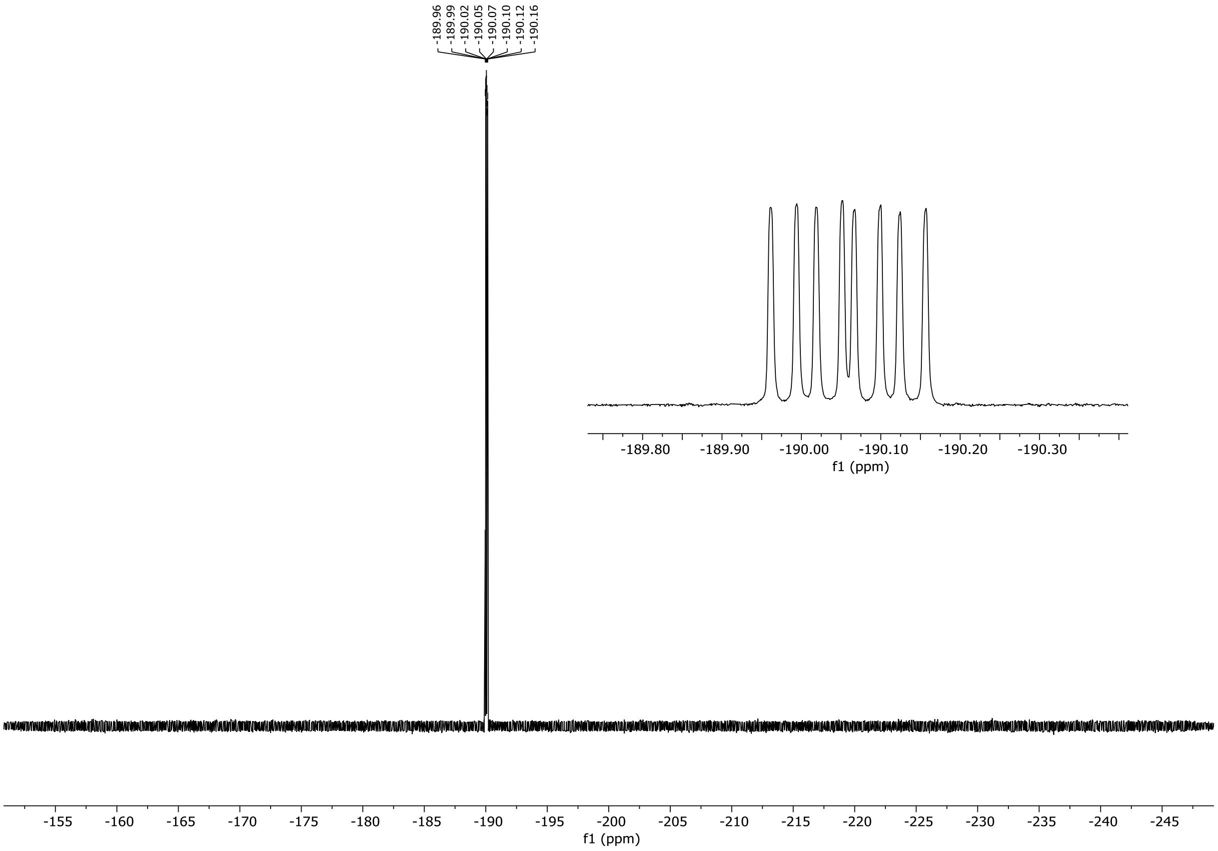

^{1}H NMR, 500 MHz, $CDCl_3$ – Compound 5

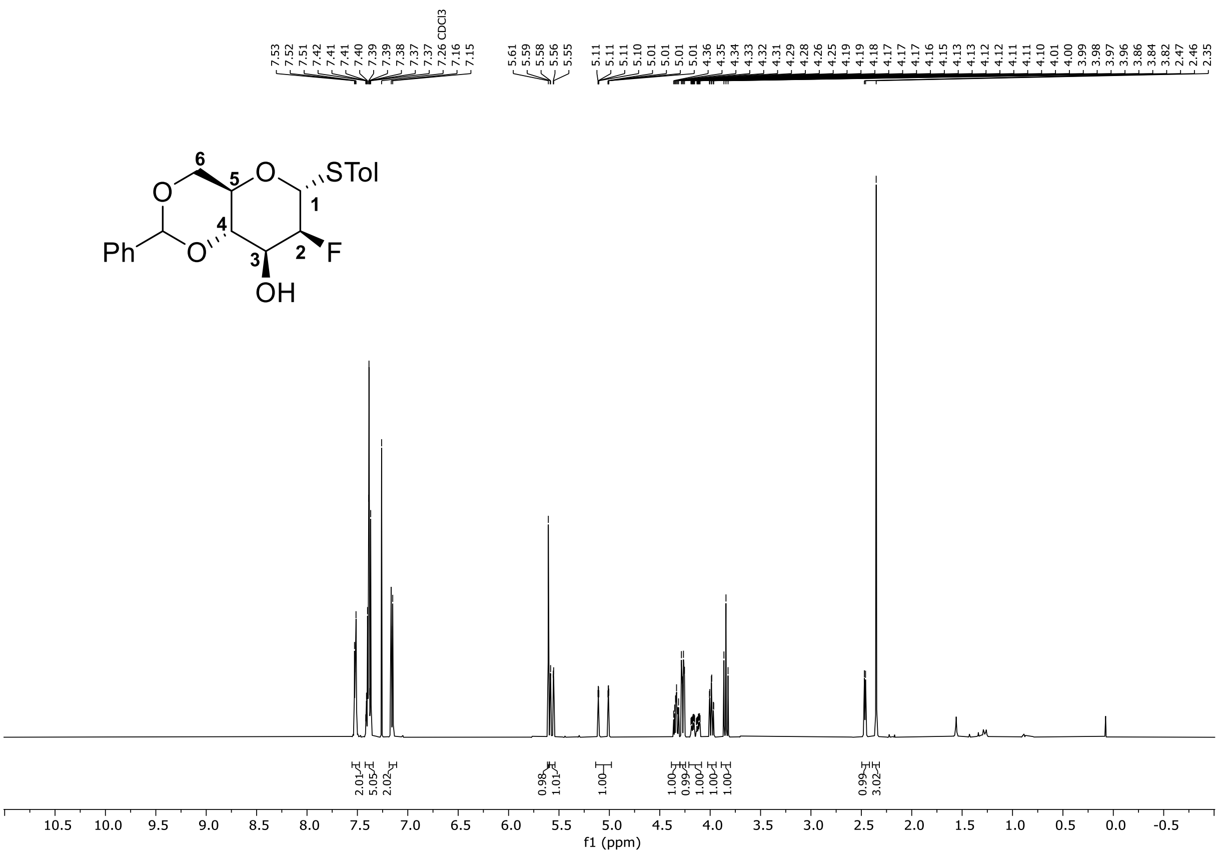

^{13}C NMR, 126 MHz, $CDCl_3$ – Compound 5

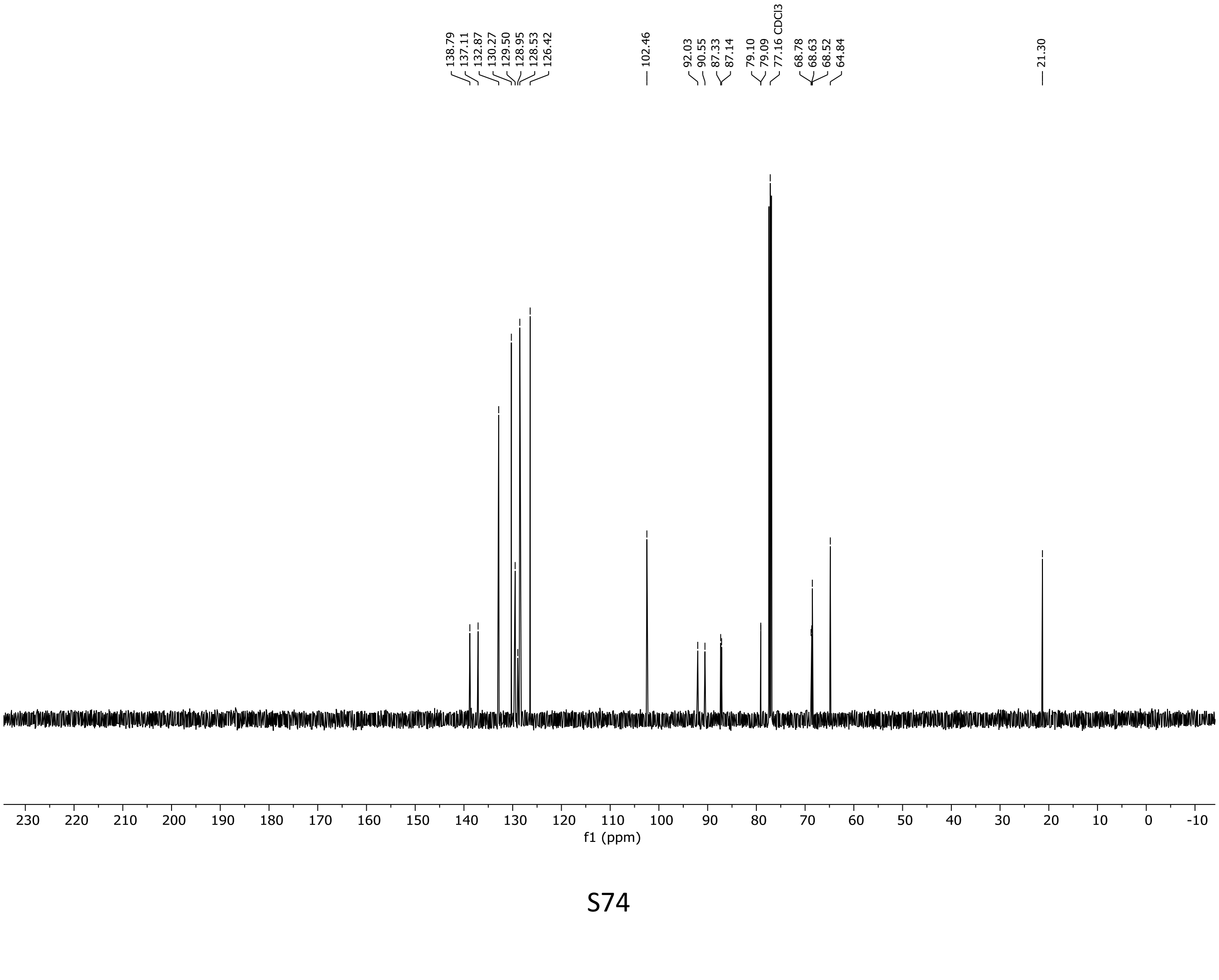

^{19}F NMR, 470 MHz, $CDCl_3$ – Compound 5

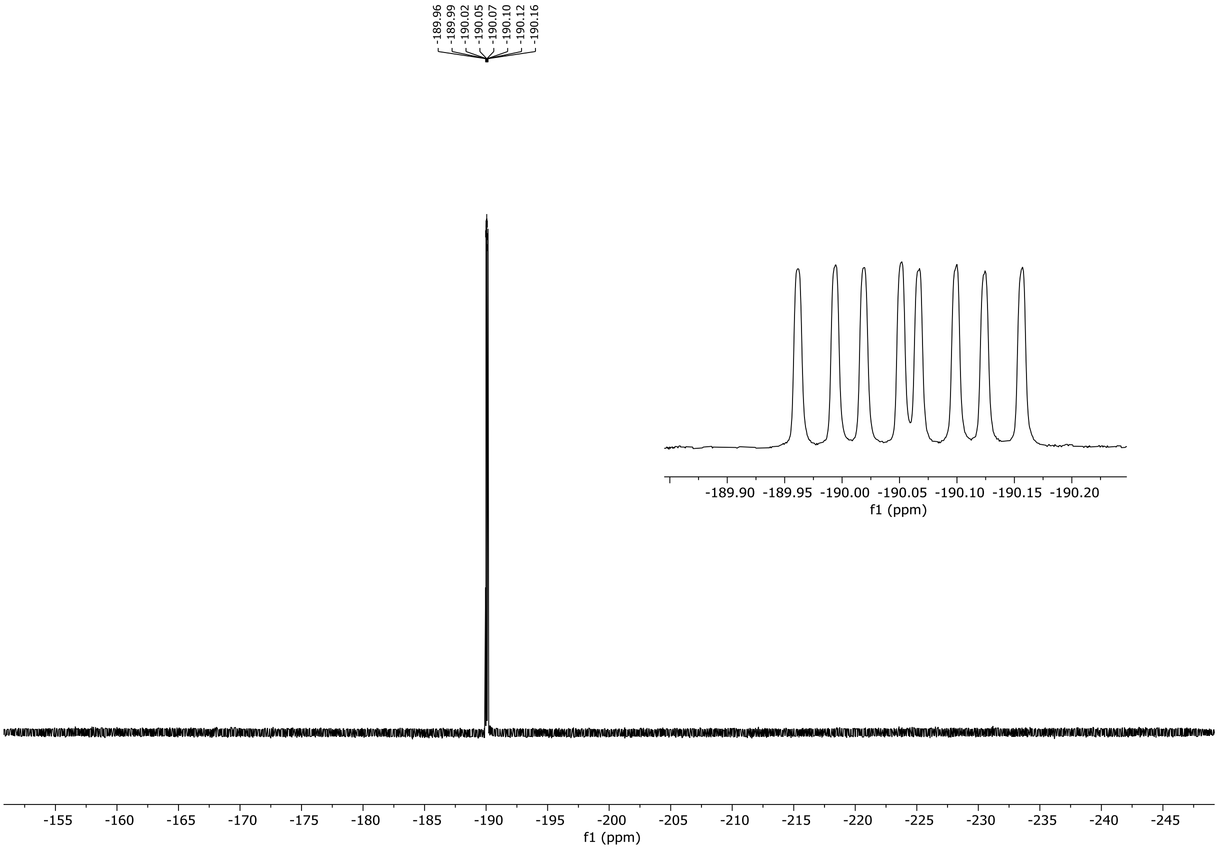

^{1}H NMR, 599 MHz, $CDCl_3$ – Compound 6

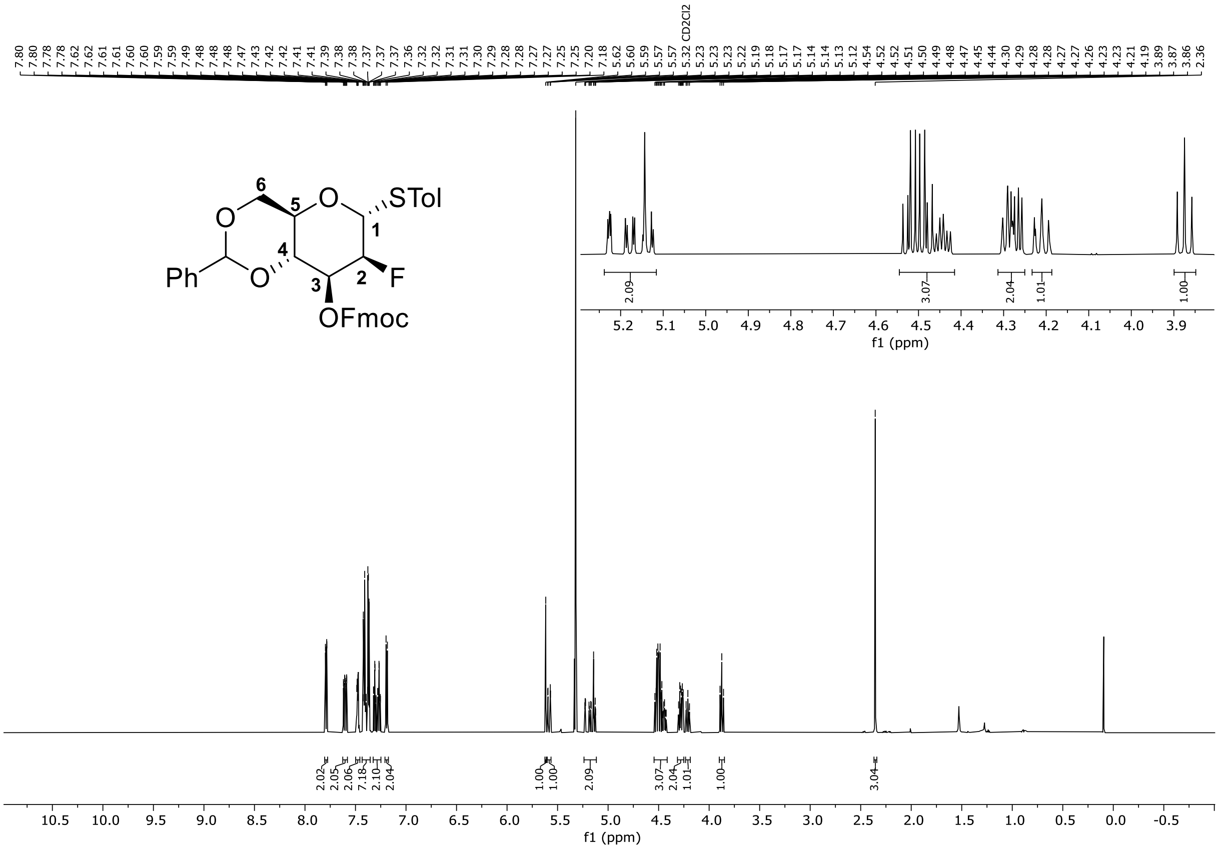

^{13}C NMR, 151 MHz, $CDCl_3$ – Compound 6

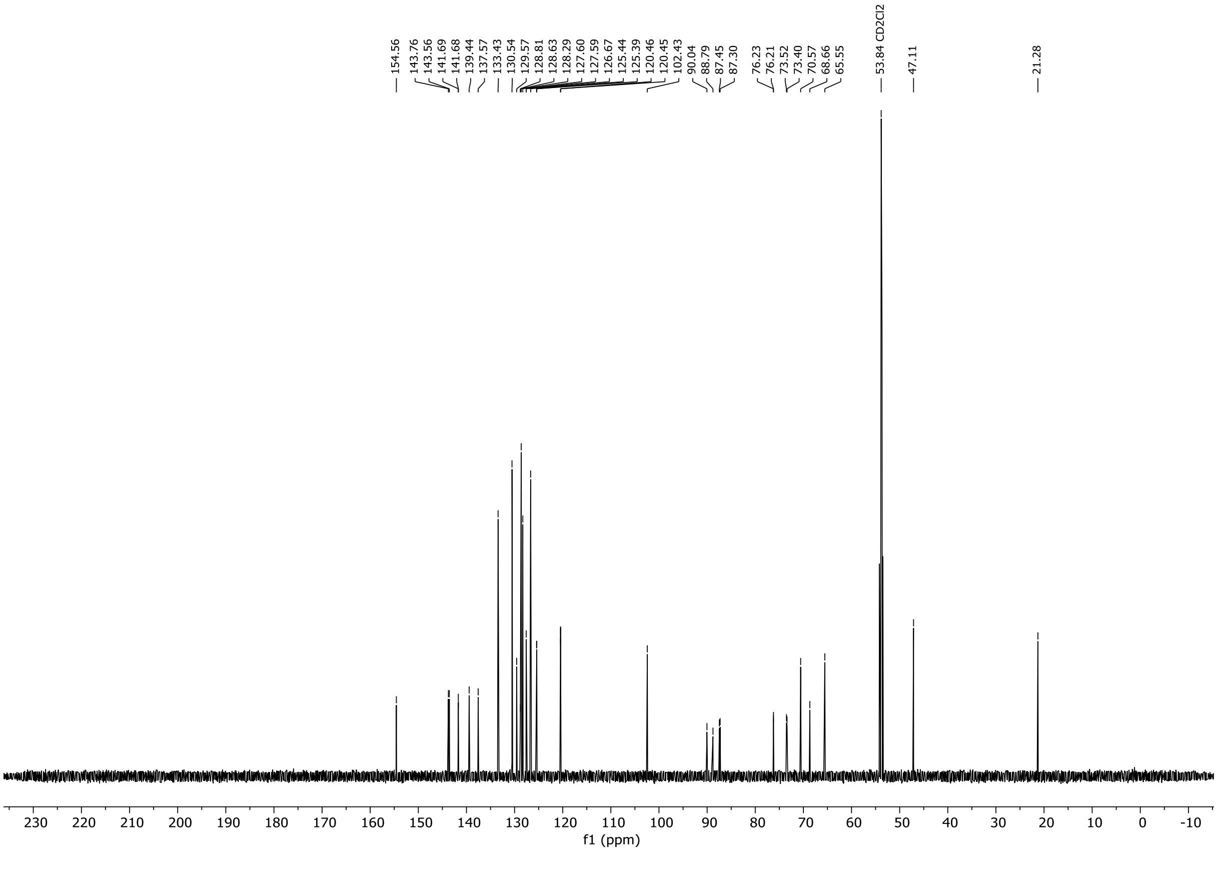

^{19}F NMR, 564 MHz, $CDCl_3$ – Compound 6

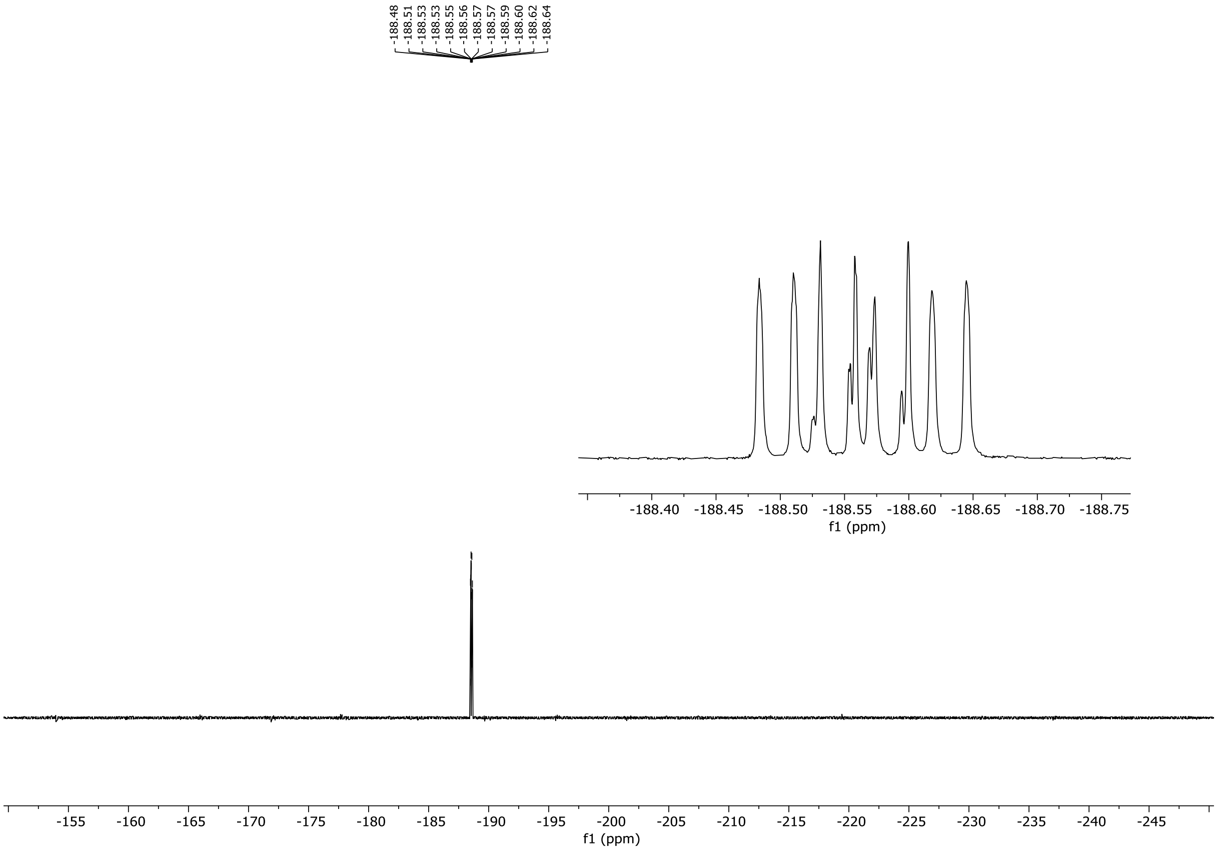

^{1}H NMR, 599 MHz, CDCl$_3$ – Compound 7

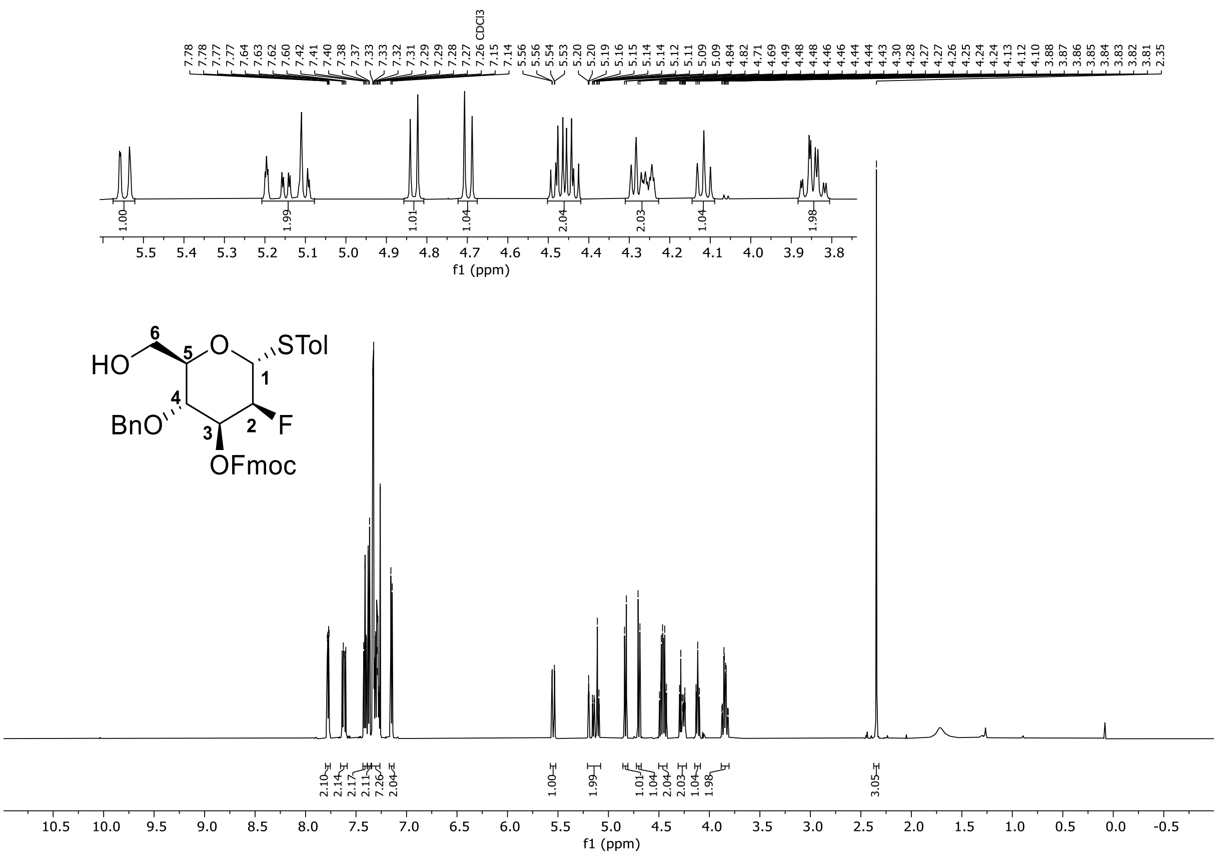

^{13}C NMR, 151 MHz, CDCl$_3$ – Compound 7

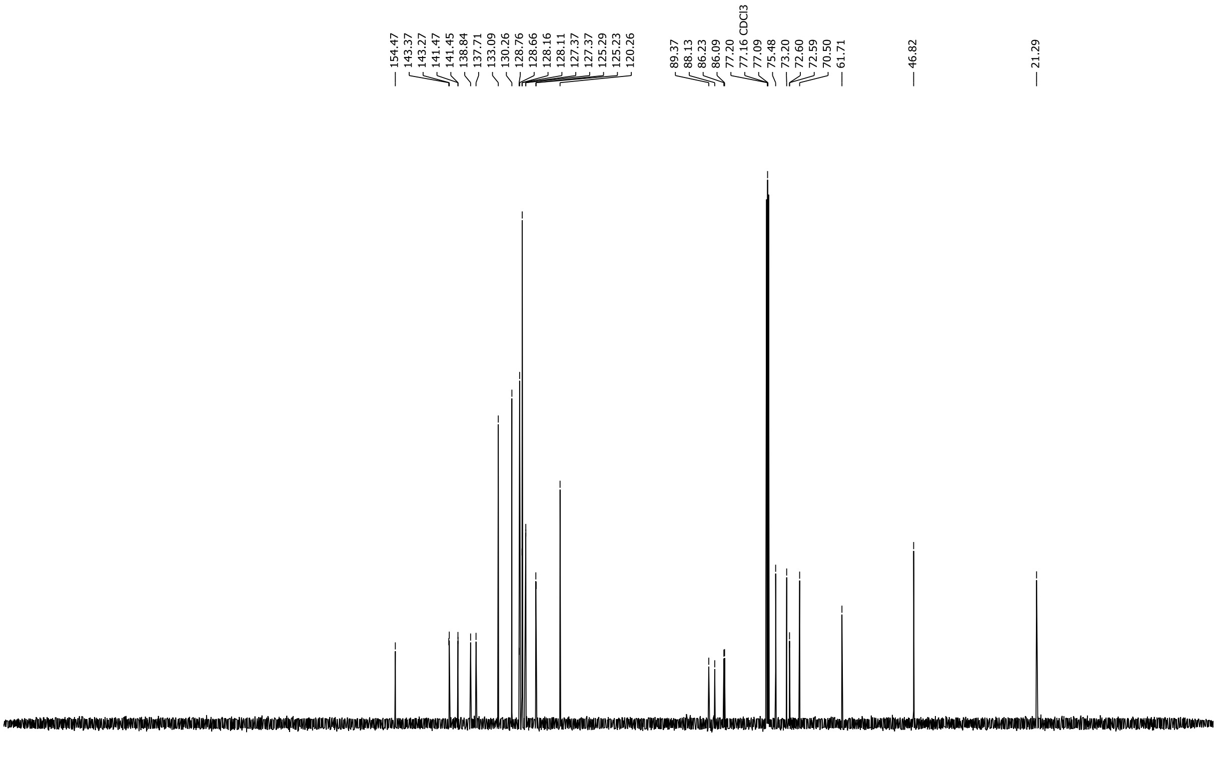

^{19}F NMR, 564 MHz, $CDCl_3$ – Compound 7

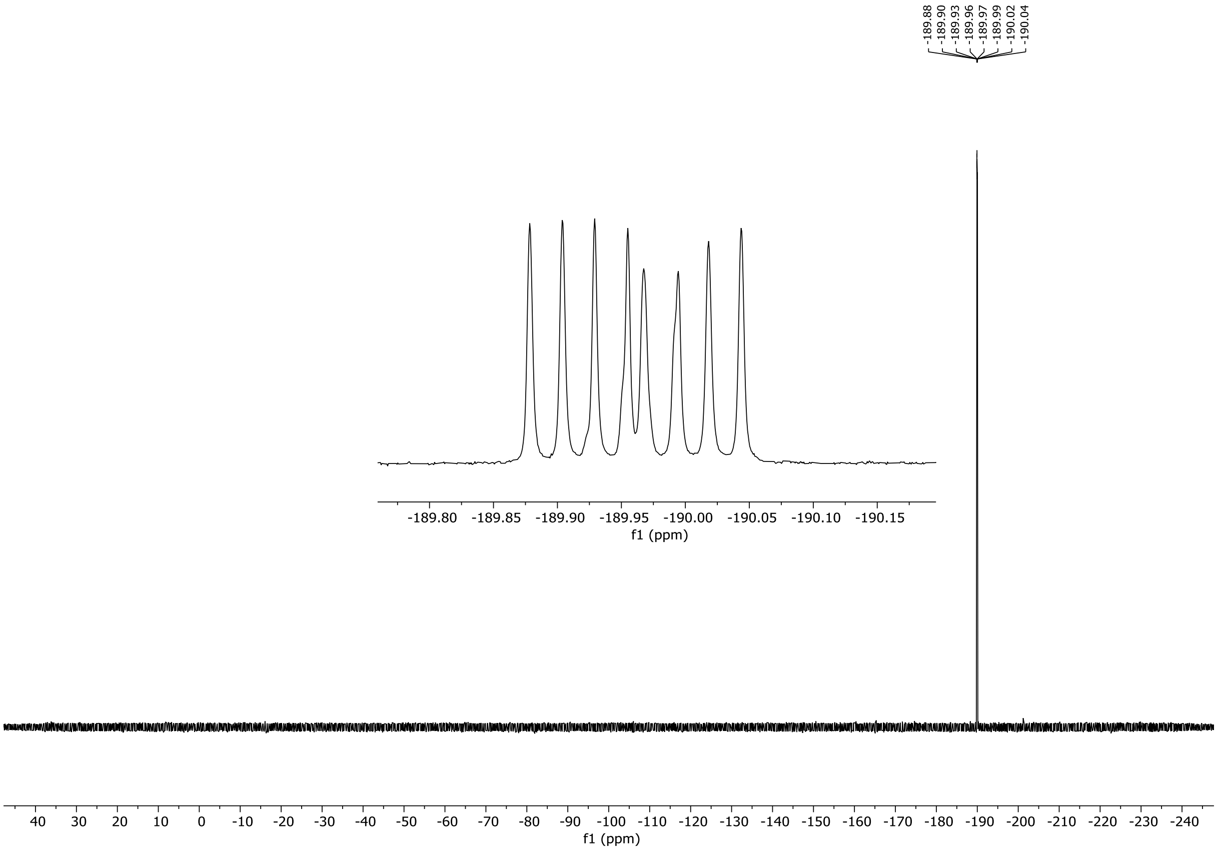

^{1}H NMR, 500 MHz, CDCl$_3$ – Compound 8

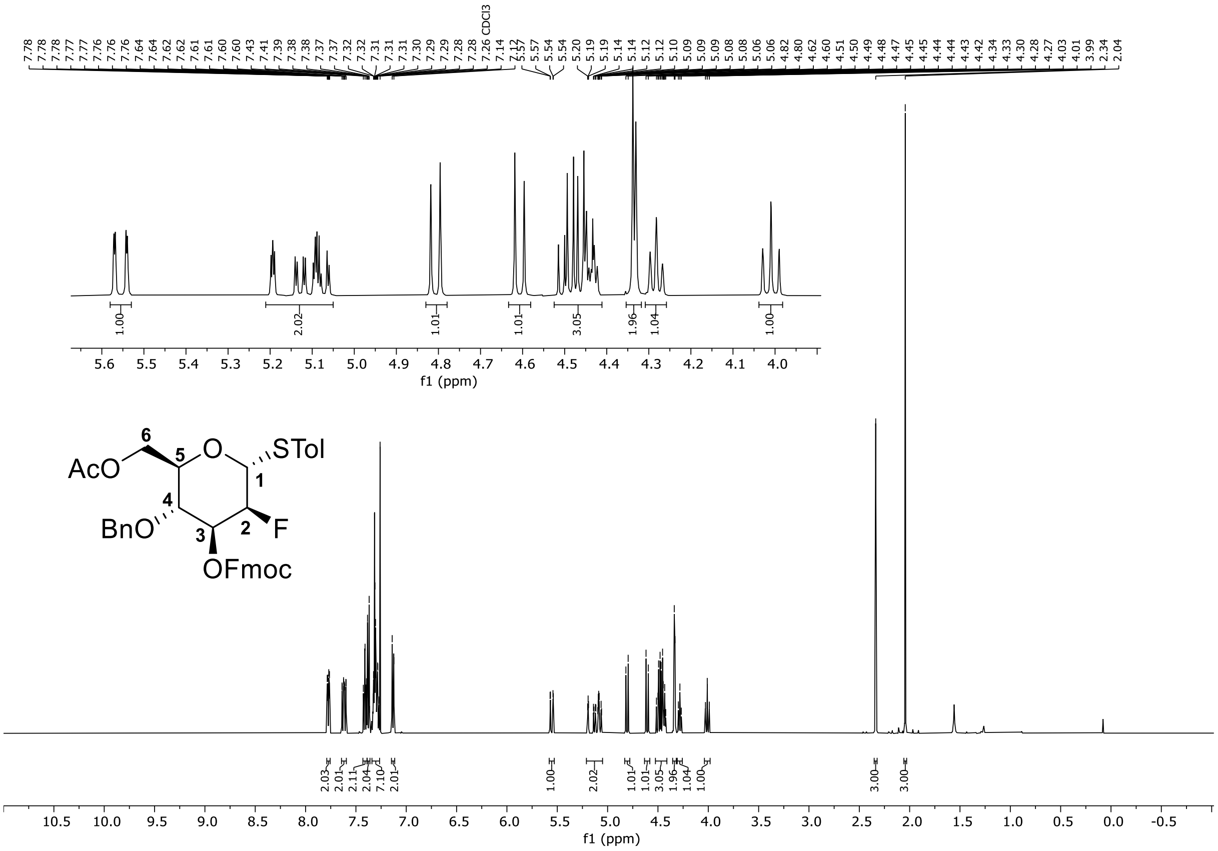

^{13}C NMR, 126 MHz, CDCl$_3$ – Compound 8

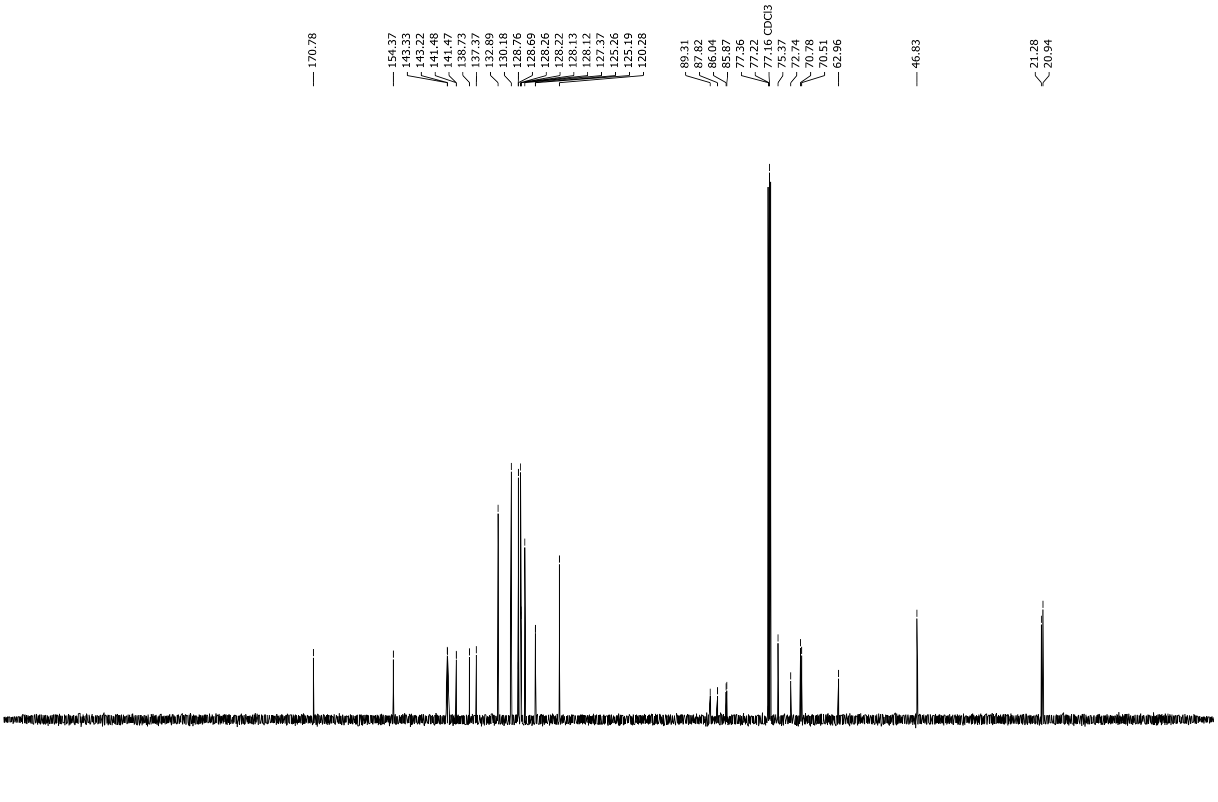

^{19}F NMR, 470 MHz, $CDCl_3$ – Compound 8

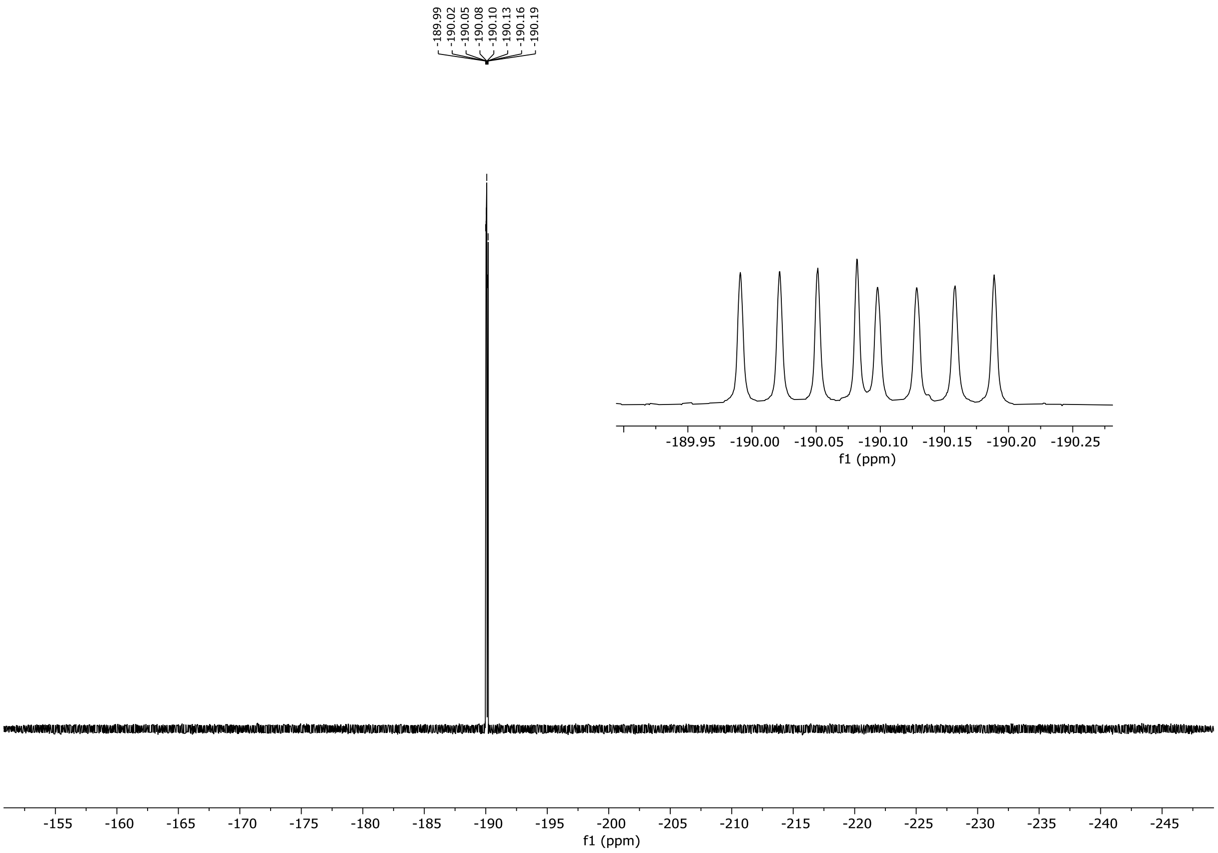

^{1}H NMR, 599 MHz, $CDCl_3$ – Compound 10

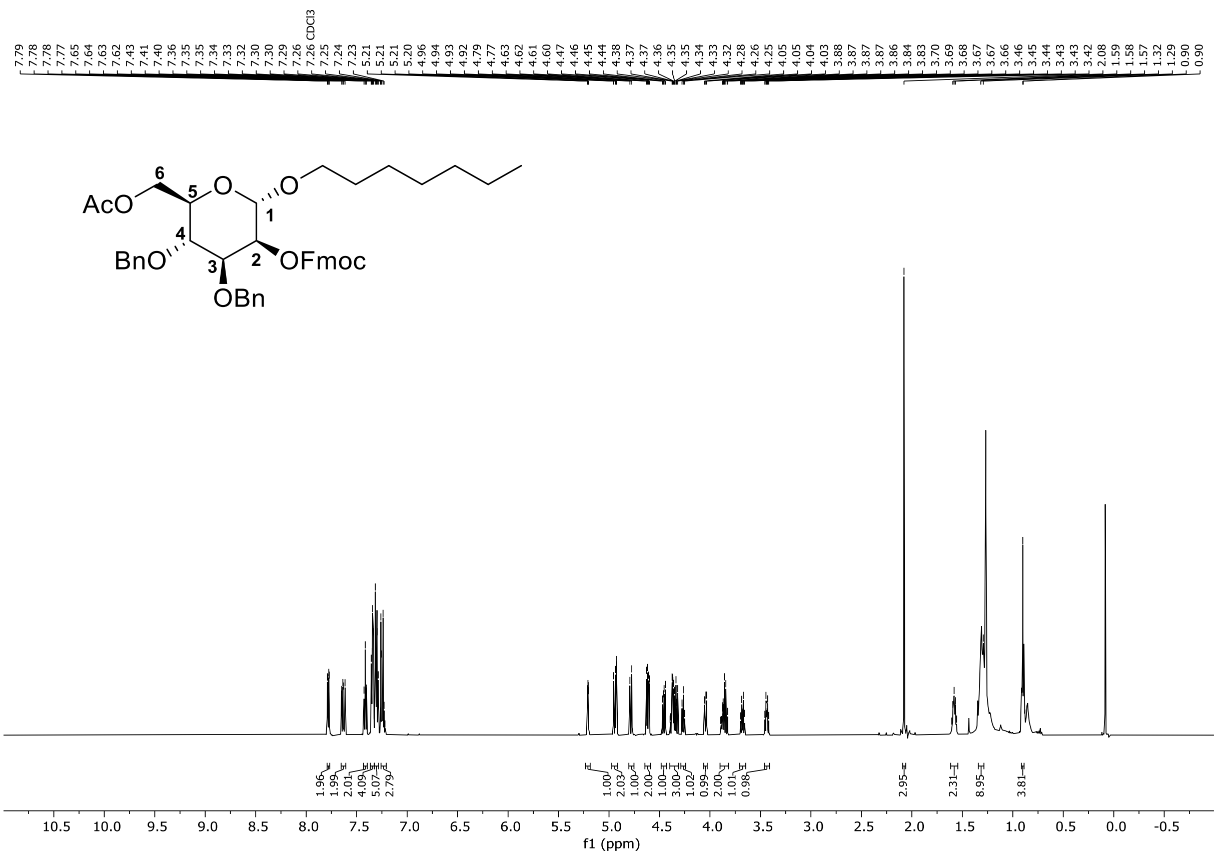

^{13}C NMR, 151 MHz, $CDCl_3$ – Compound 10

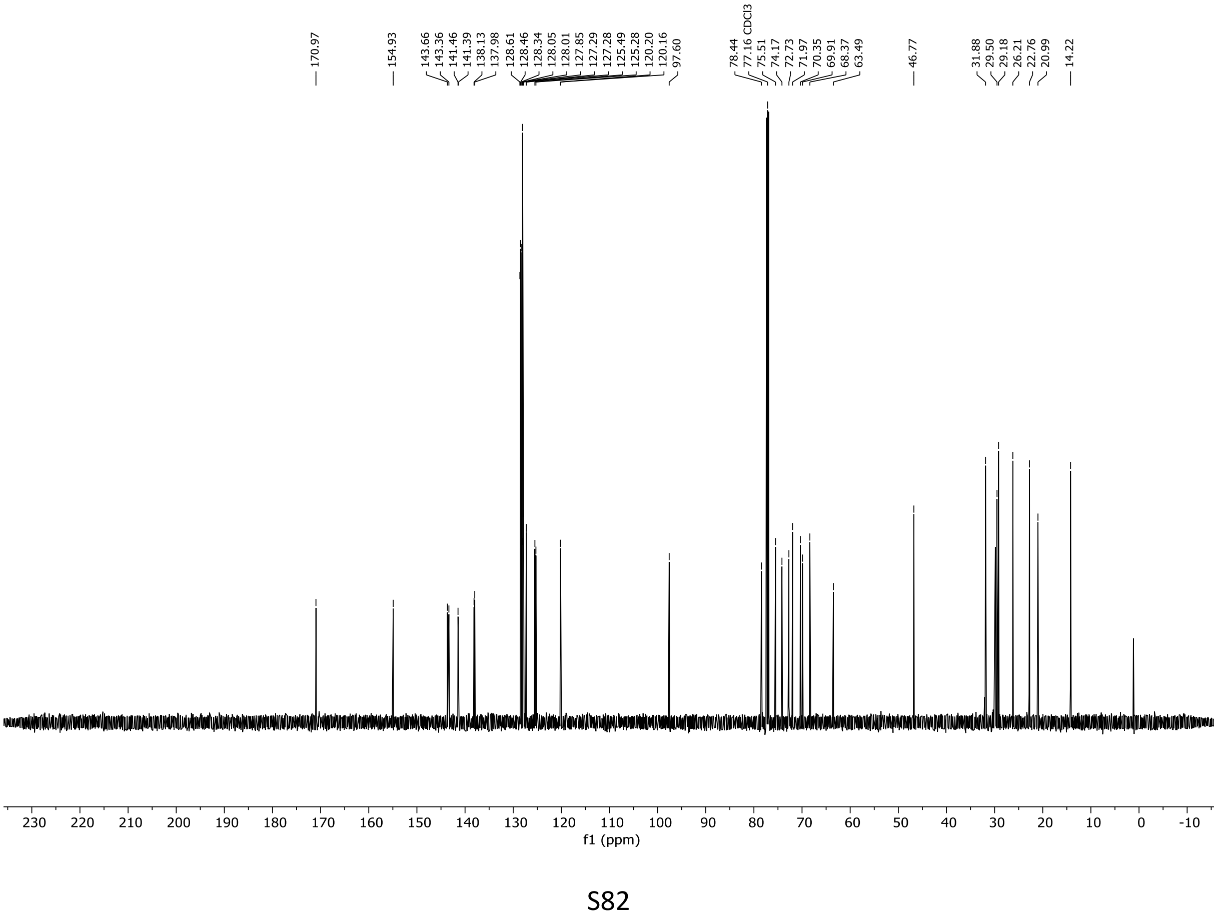

^{1}H-^{13}C gHSQC NMR, $CDCl_3$ – Carbohydrate Region, Compound 10

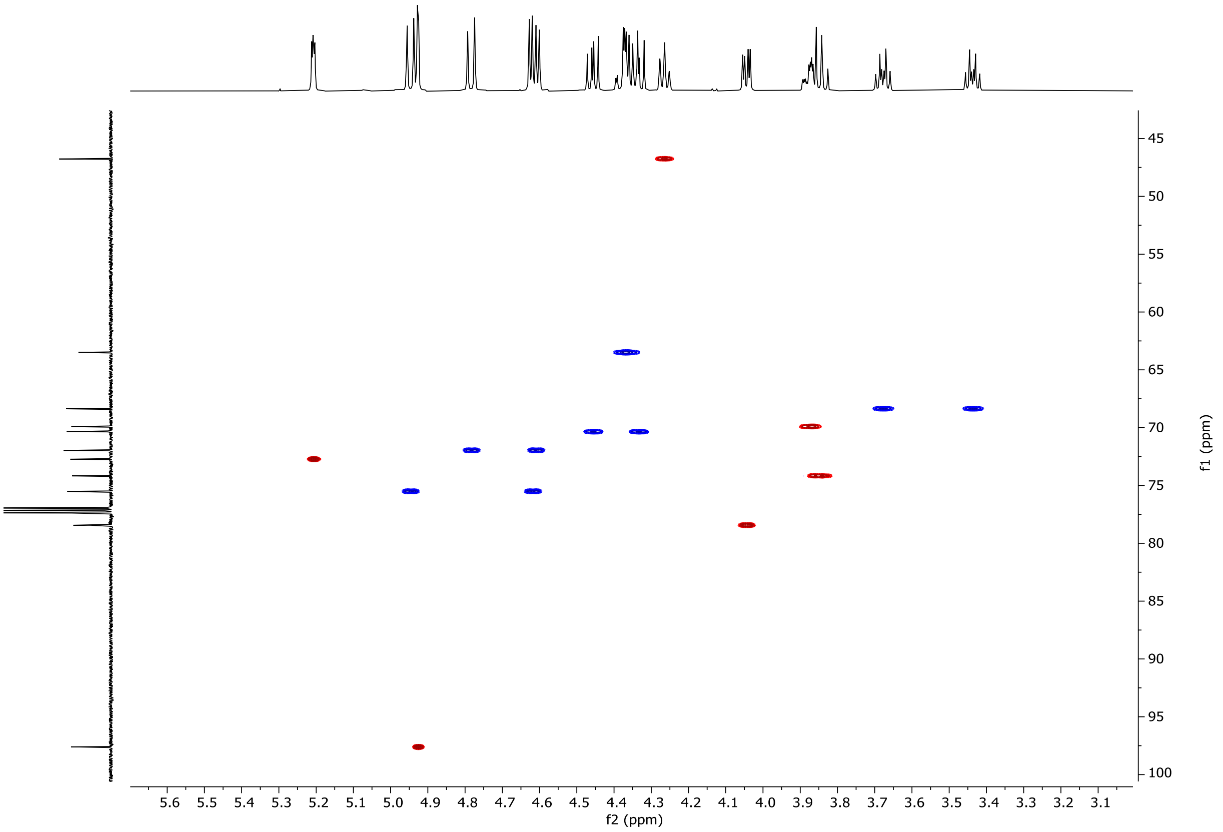

^{1}H NMR, 599 MHz, $CDCl_3$ – Compound 11

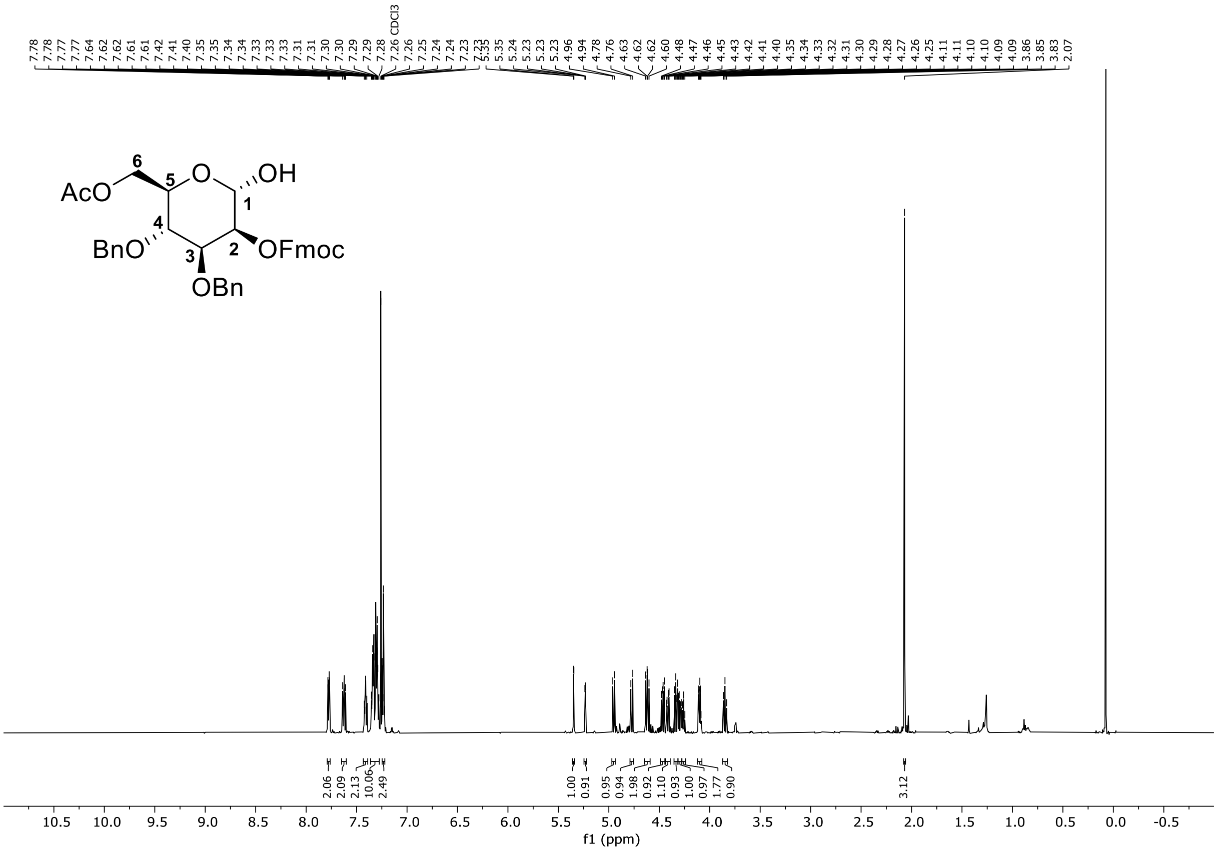

^{13}C NMR, 151 MHz, $CDCl_3$ – Compound 11

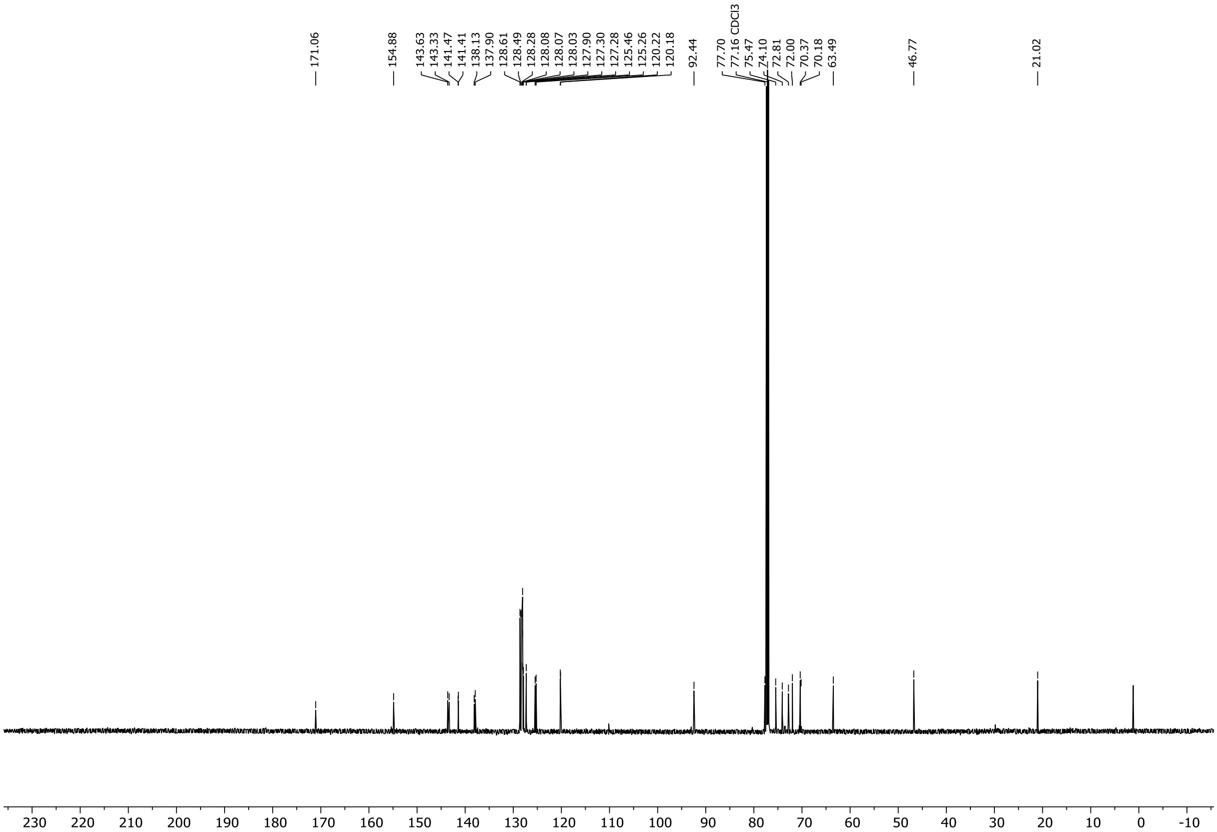

^{1}H-^{13}C gHSQC NMR, $CDCl_3$ – Carbohydrate Region, Compound 11

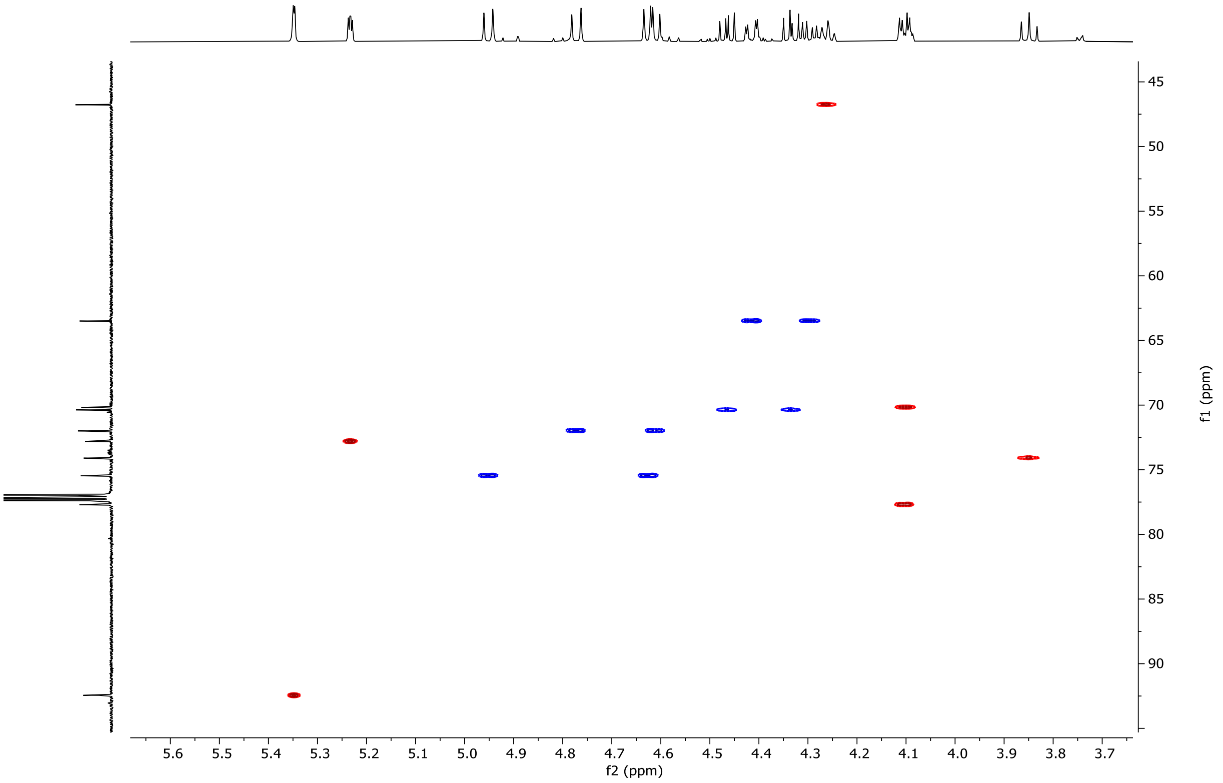

^{1}H NMR, 599 MHz, $CDCl_3$ – Compound 12

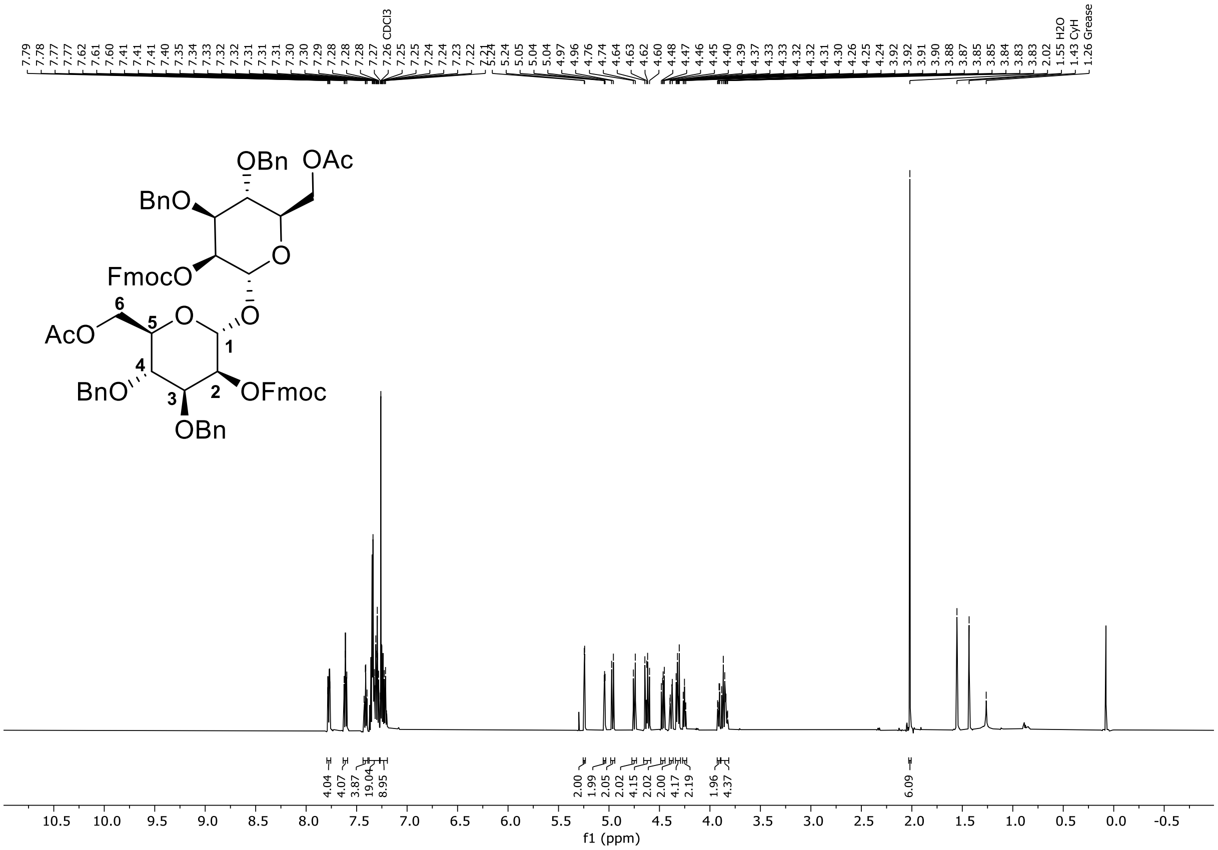

^{13}C NMR, 151 MHz, $CDCl_3$ – Compound 12

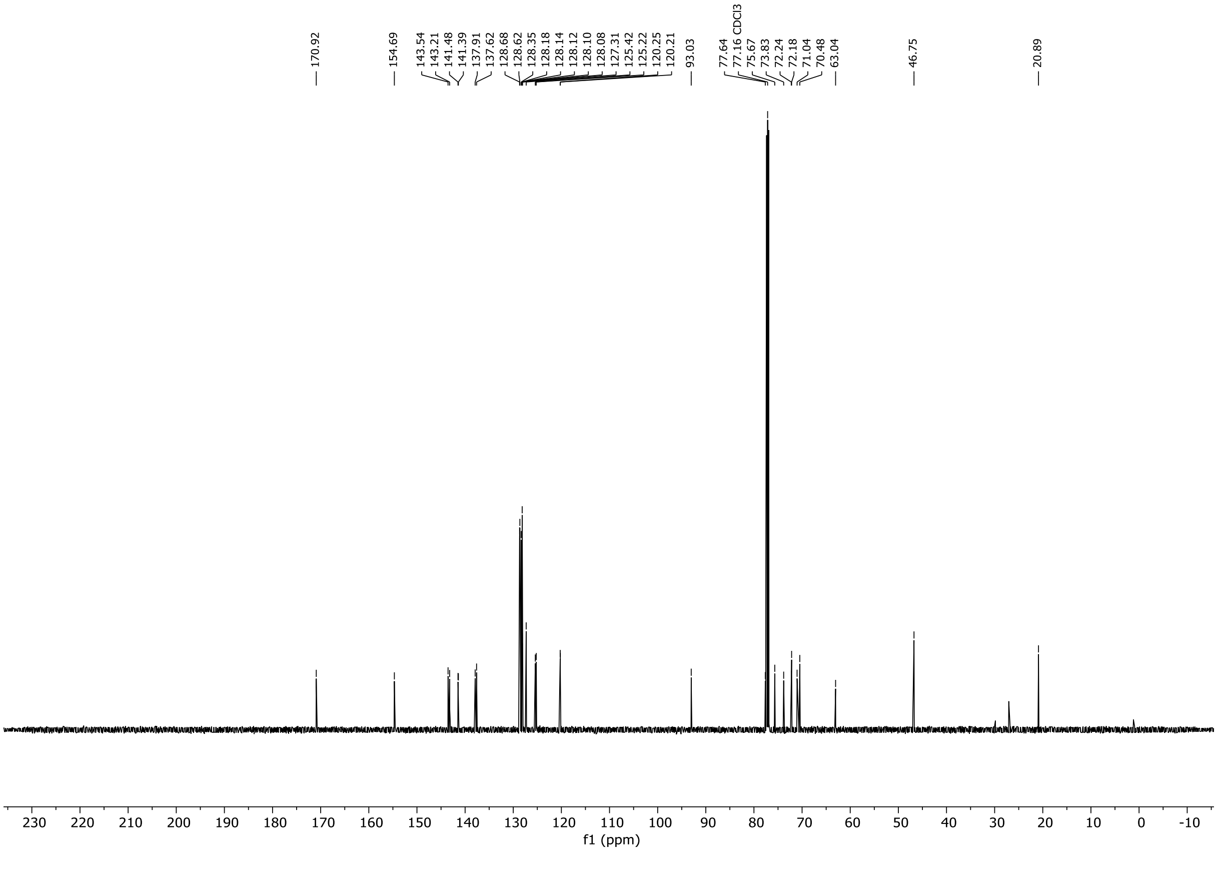

^{1}H-^{13}C gHSQC NMR, $CDCl_3$ – Carbohydrate Region, Compound 12

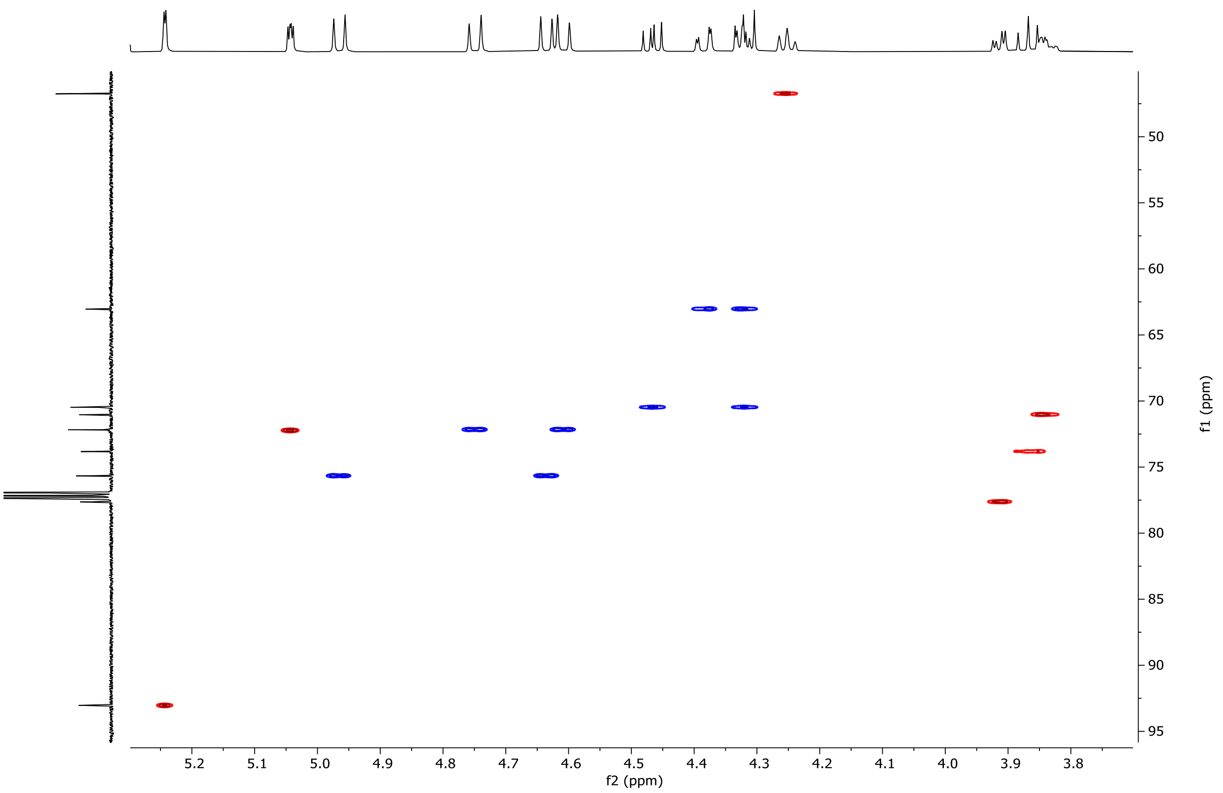

^{1}H NMR, 599 MHz, $CDCl_3$ – Compound 13

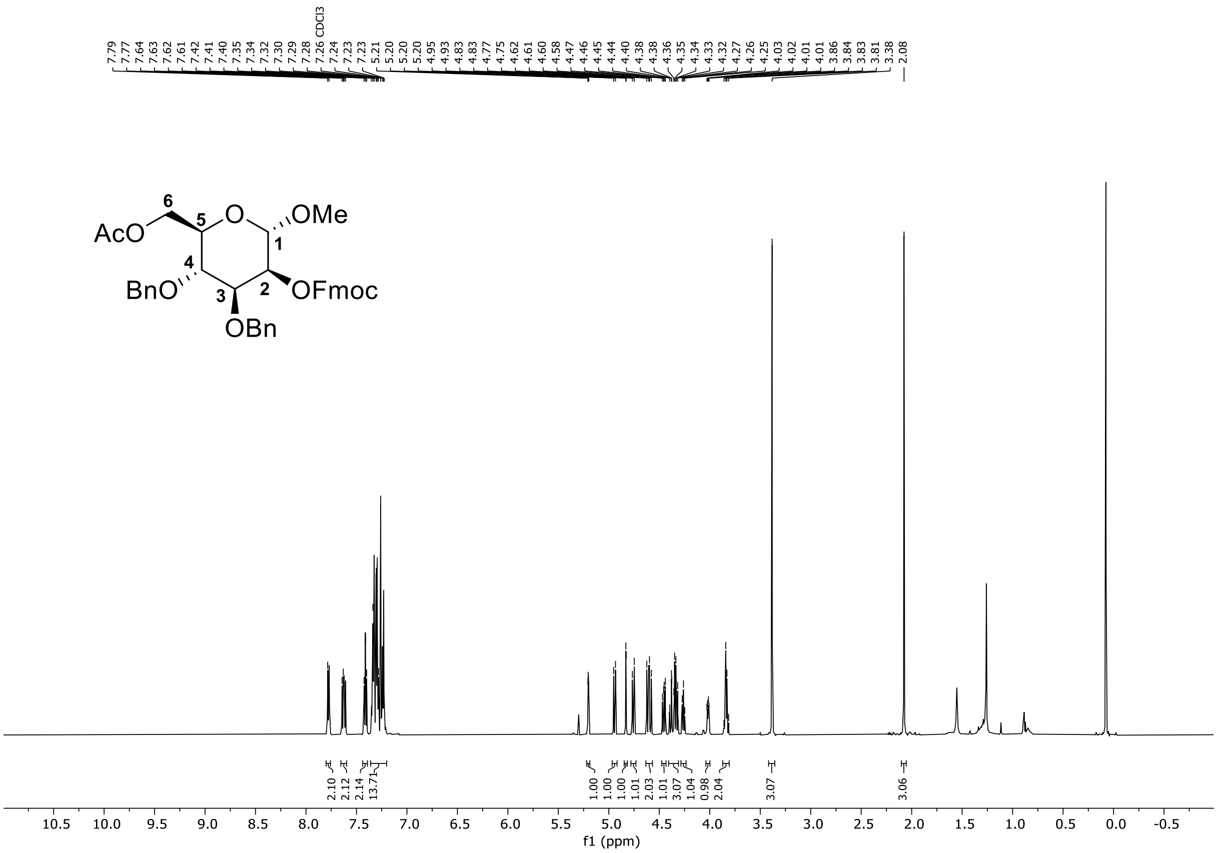

^{13}C NMR, 151 MHz, $CDCl_3$ – Compound 13

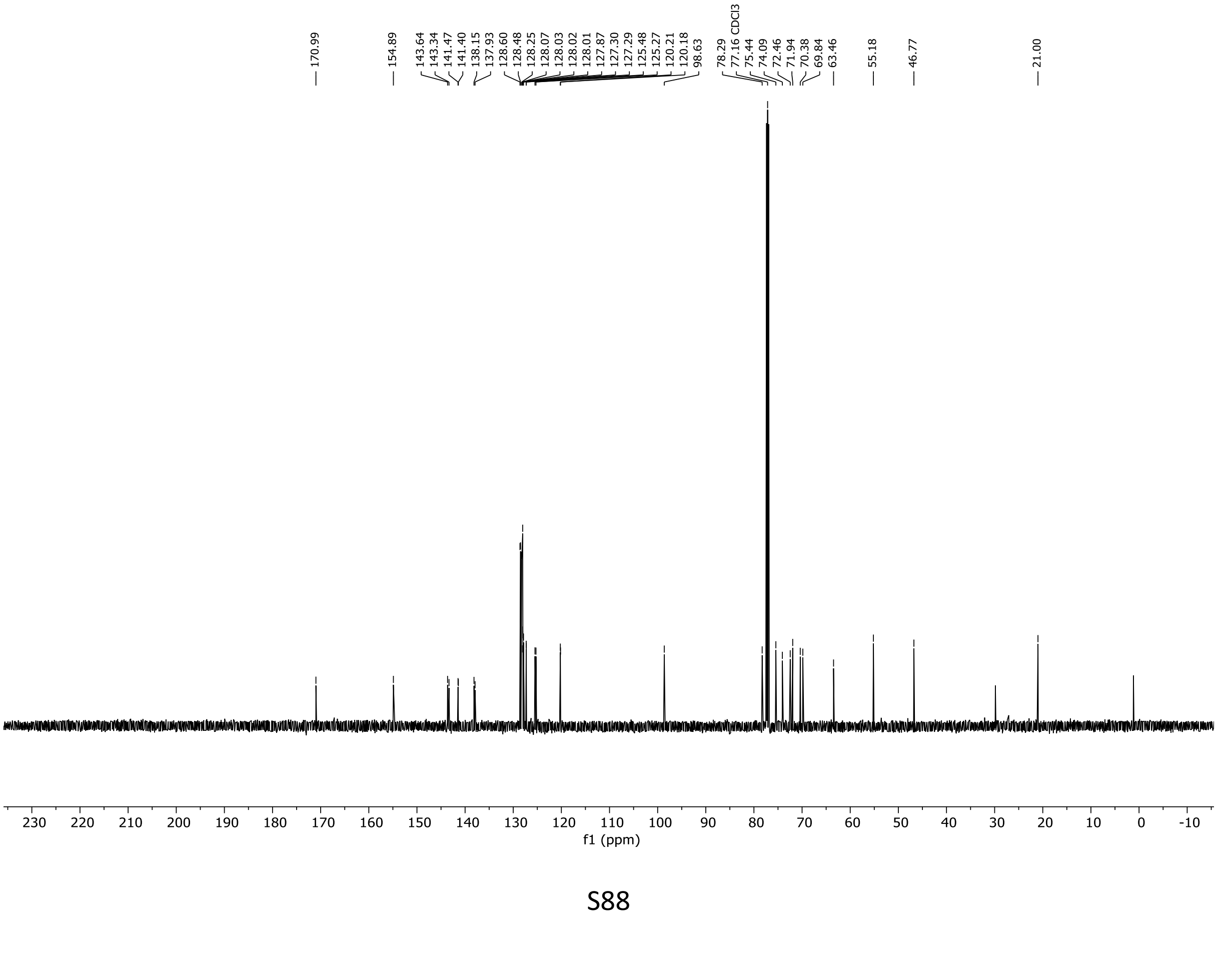

^{1}H-^{13}C gHSQC NMR, $CDCl_3$ – Carbohydrate Region Compound 13

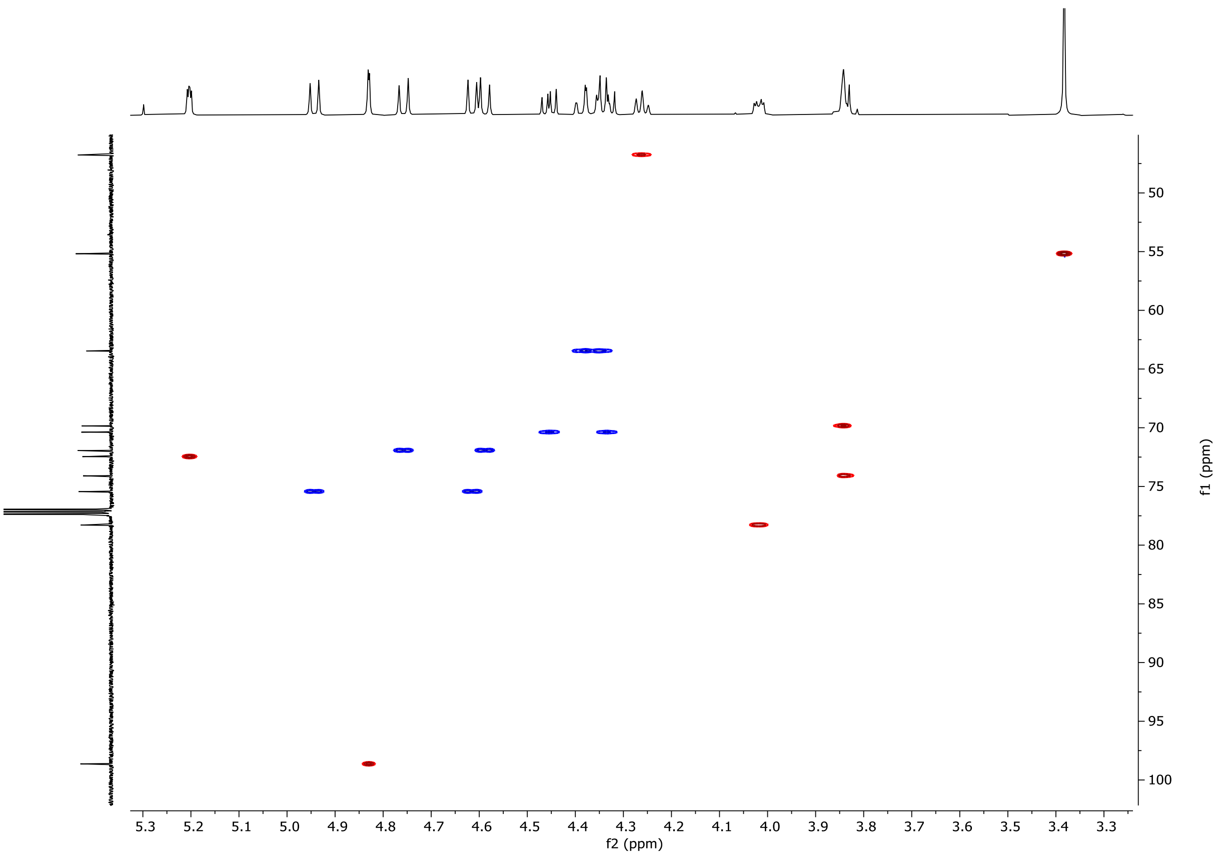

^{1}H NMR, 599 MHz, $CDCl_3$ – Compound 14

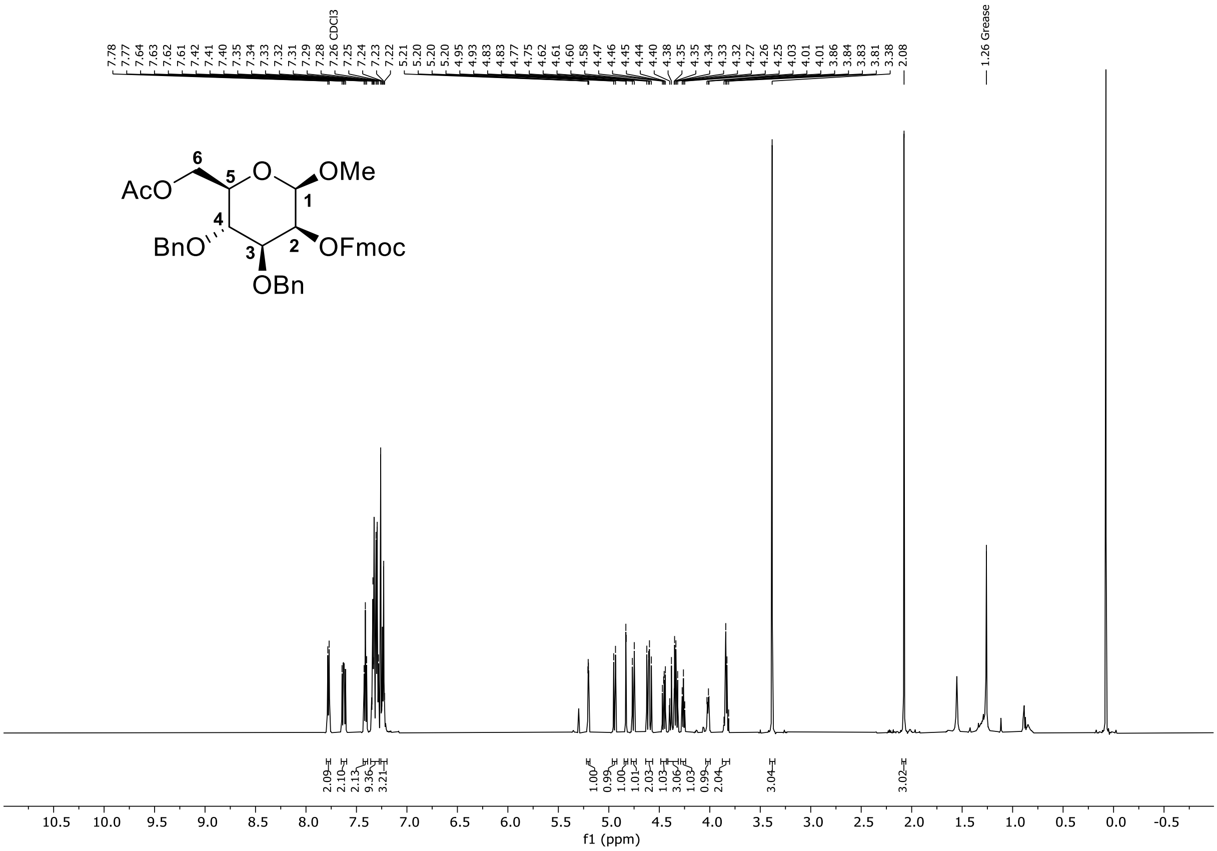

^{13}C NMR, 151 MHz, $CDCl_3$ – Compound 14

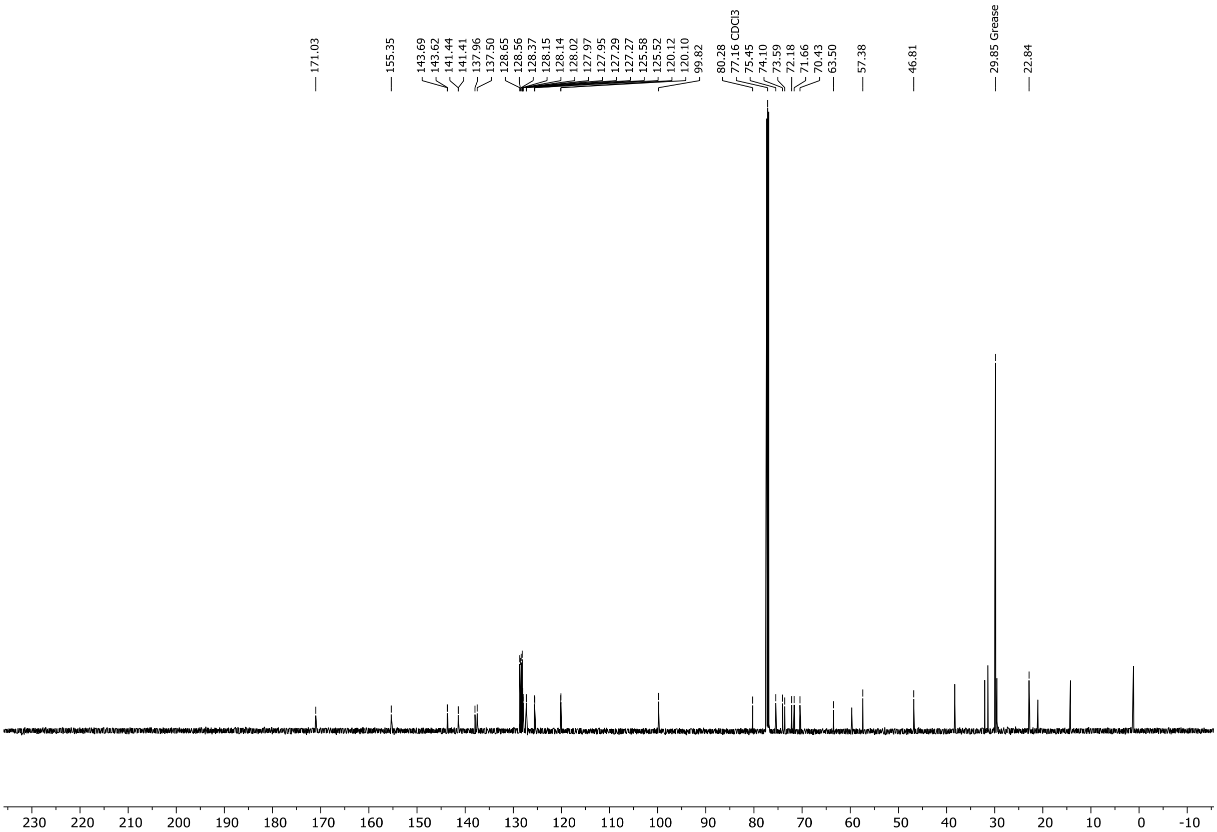

^{1}H-^{13}C gHSQC NMR, $CDCl_3$ – Carbohydrate Region, Compound 14

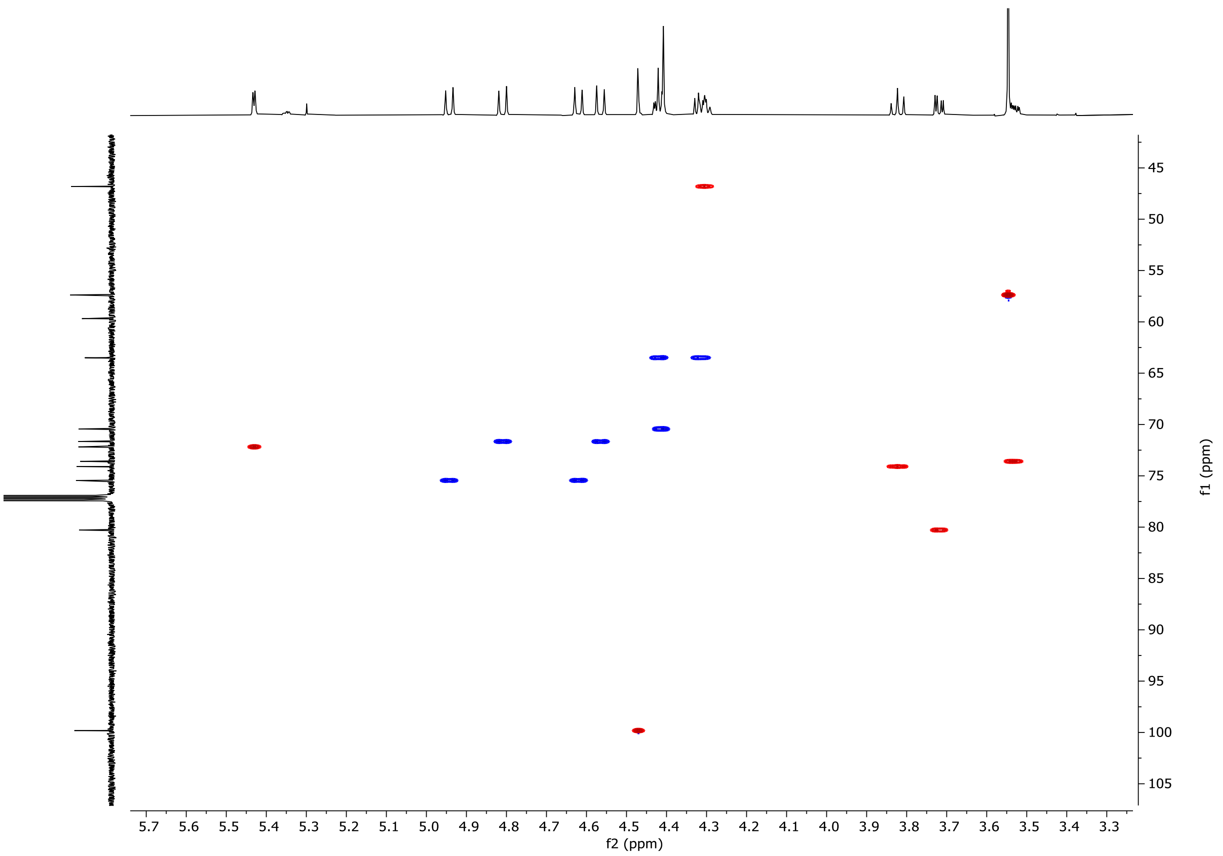

^{1}H NMR, 599 MHz, CDCl$_3$ – Crude Compound S10

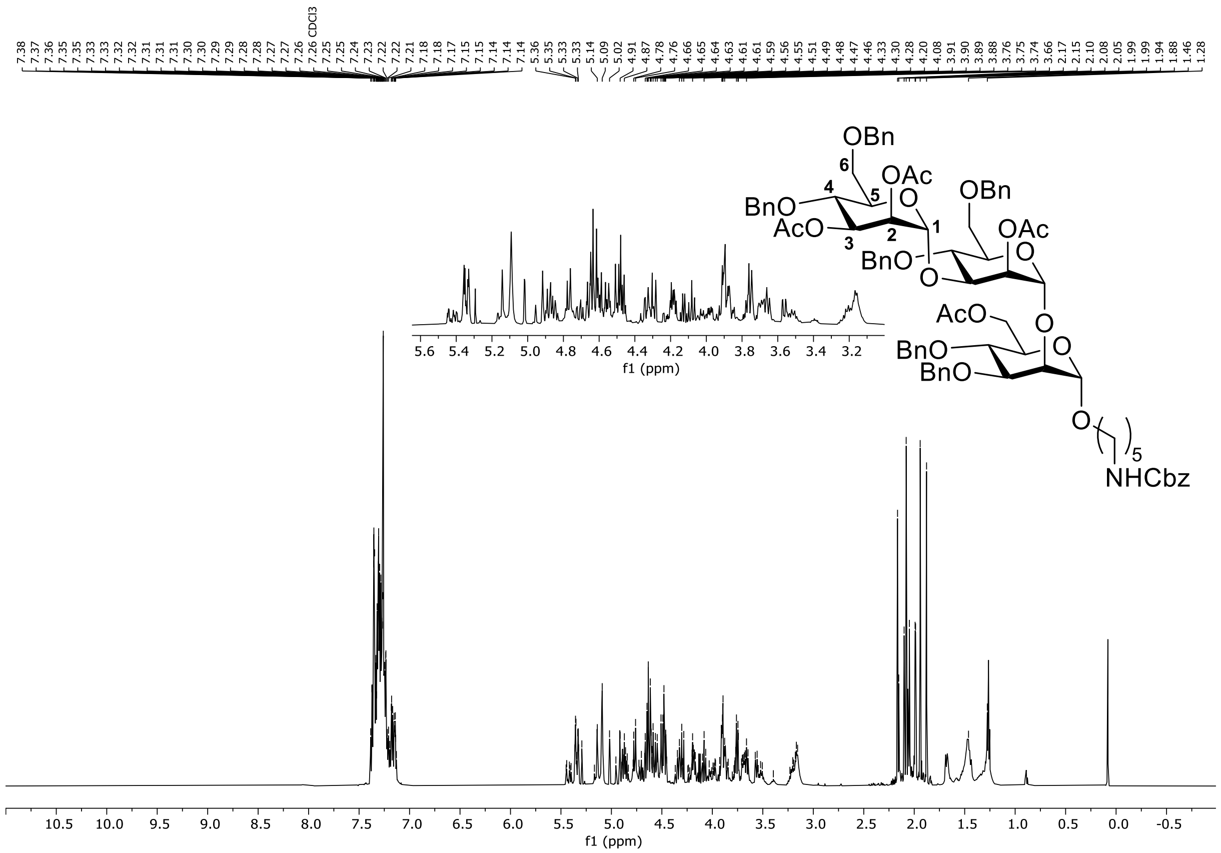

^{13}C NMR, 151 MHz, CDCl$_3$ – Crude Compound S10

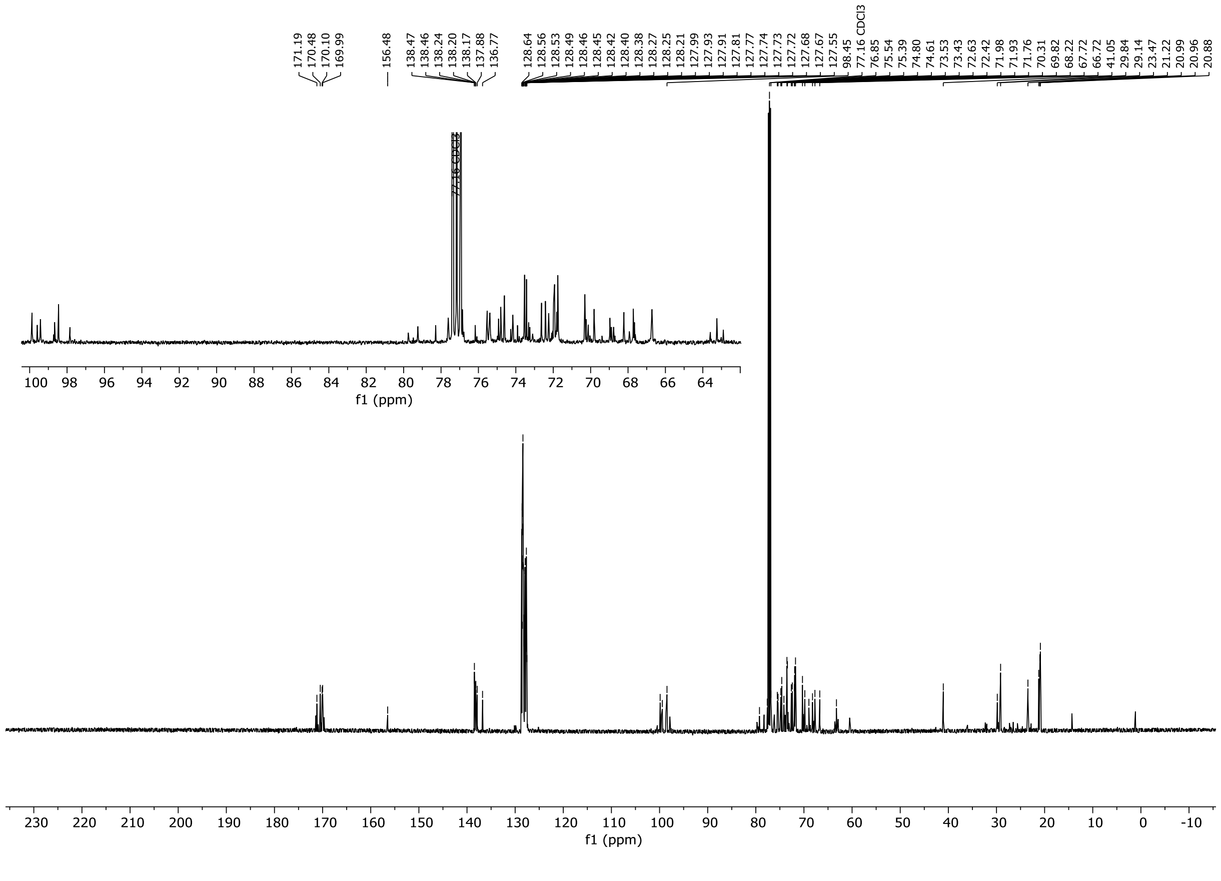

^{1}H-^{13}C gHSQC NMR, $CDCl_3$ – Carbohydrate Region, Crude Compound S10

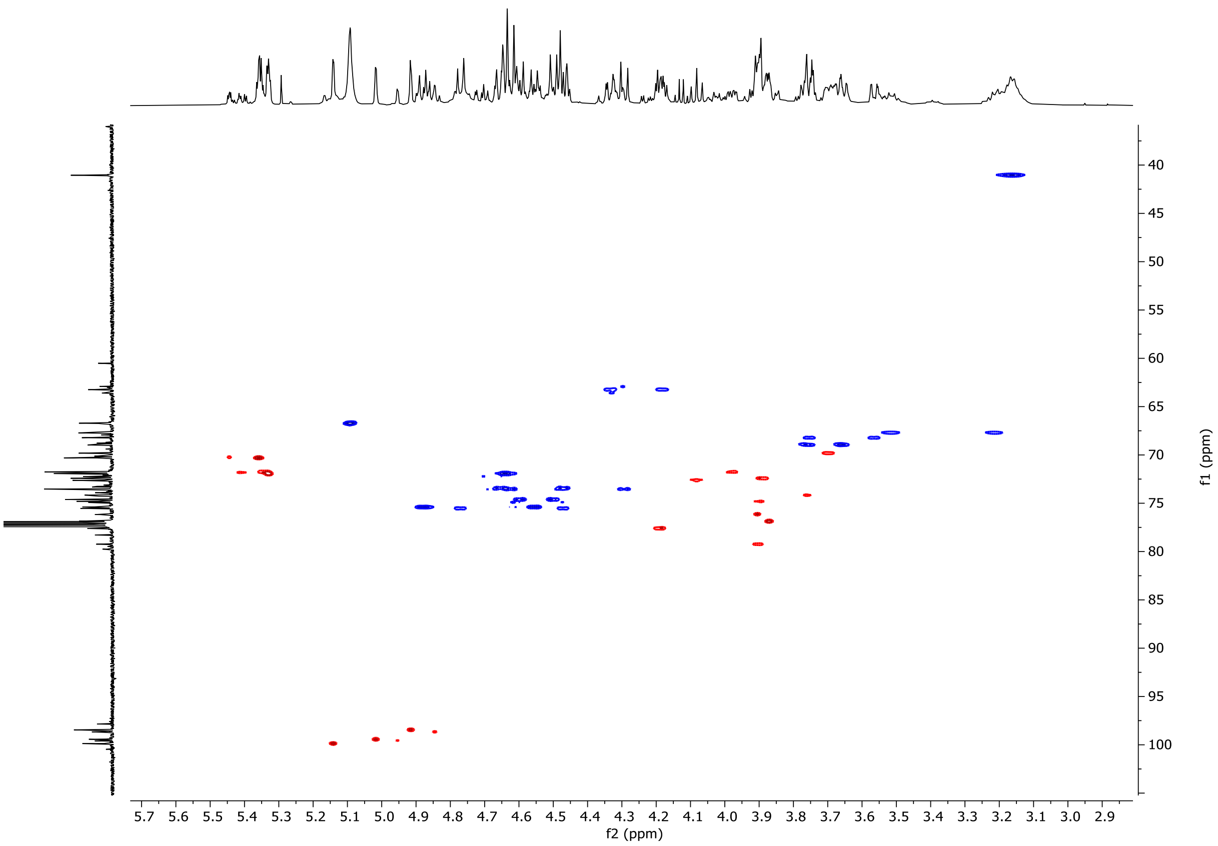

^{1}H NMR, 599 MHz, $CDCl_3$ – Compound 18

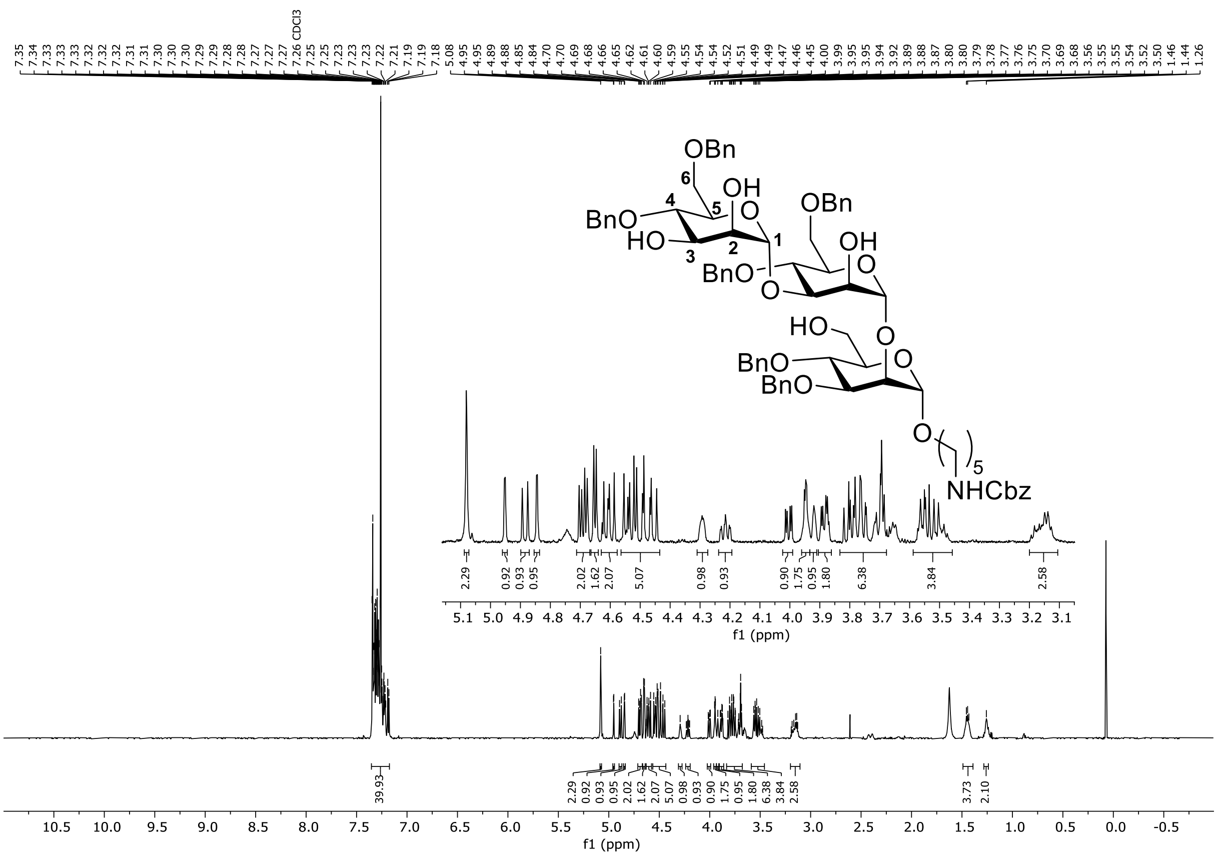

^{13}C NMR, 151 MHz, $CDCl_3$ – Compound 18

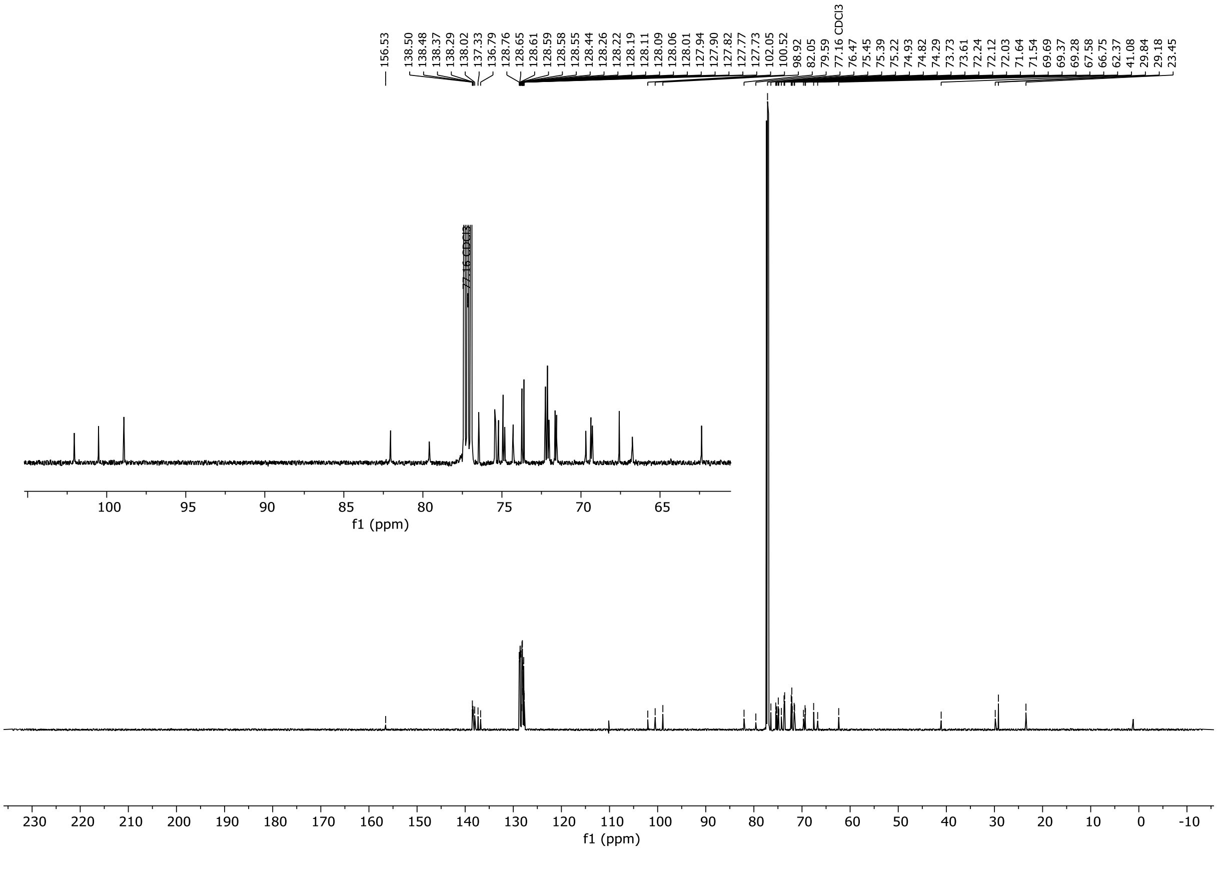

^{1}H-^{13}C gHSQC NMR, $CDCl_3$ – Carbohydrate Region, Compound 18

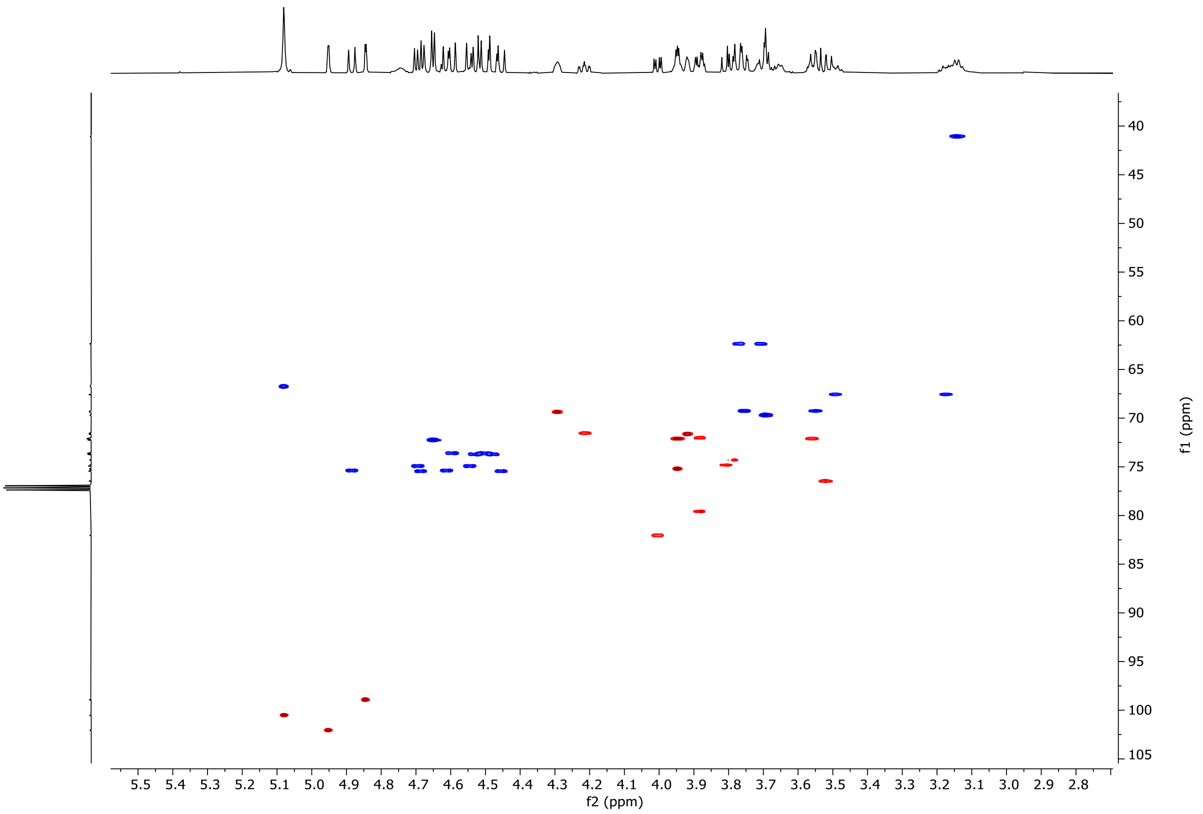

^{1}H NMR, 500 MHz, D_2O – Compound 24

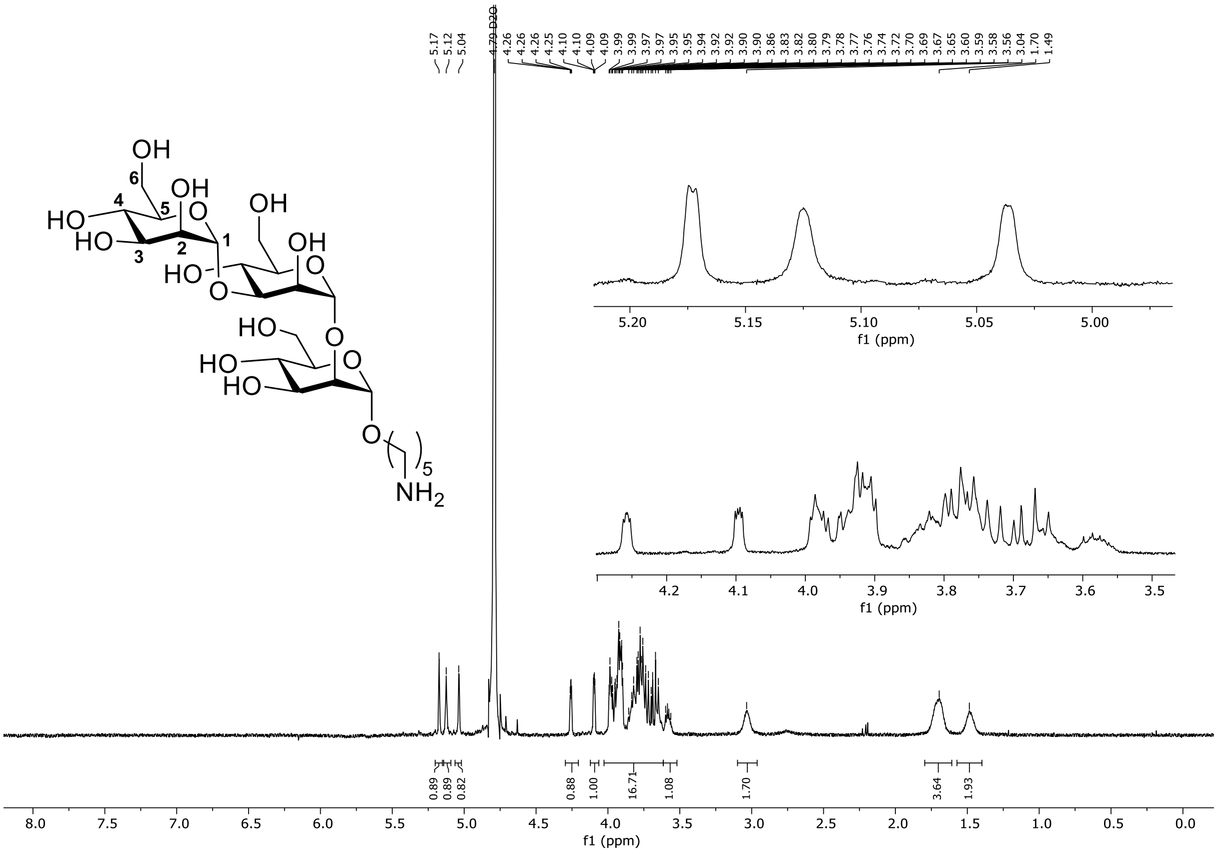

^{1}H-^{13}C gHSQC NMR, D_2O – Compound 24

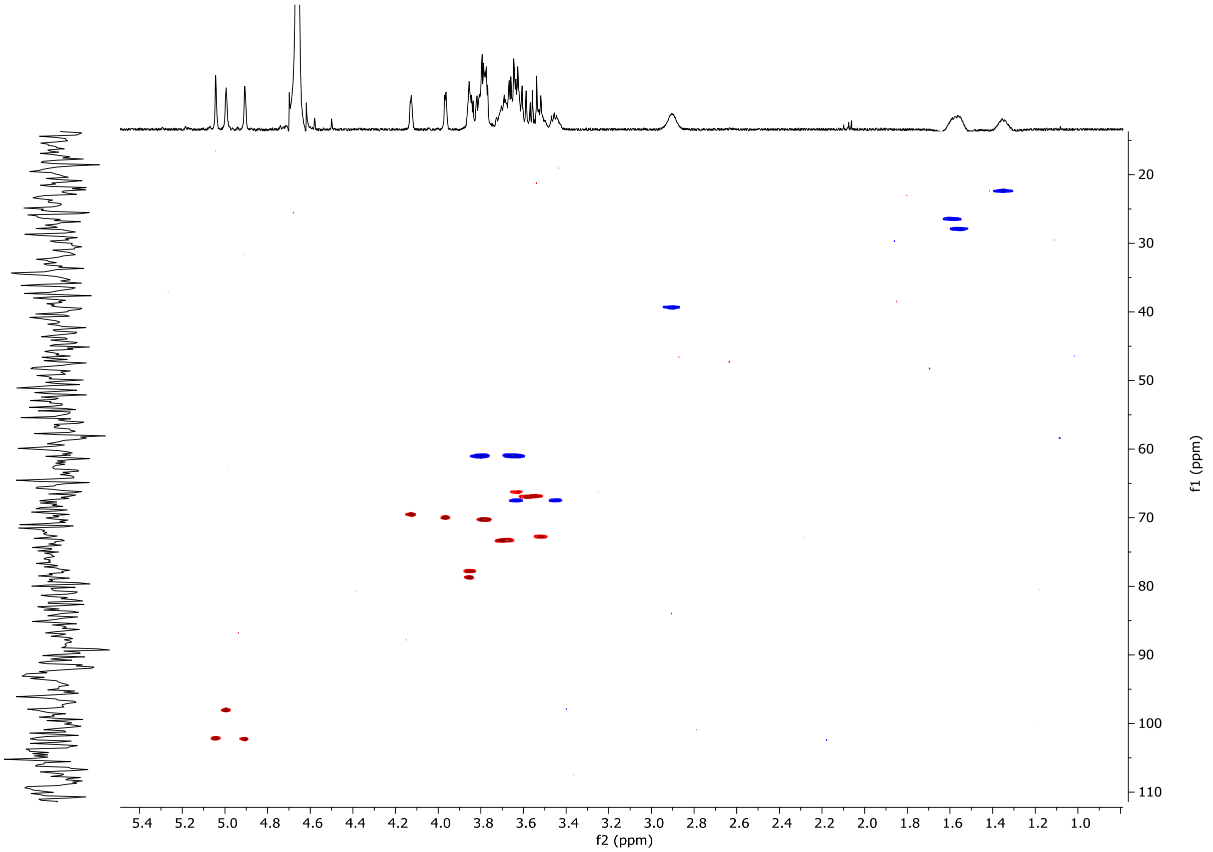

^{1}H NMR, 599 MHz, $CDCl_3$ – Crude Compound S11

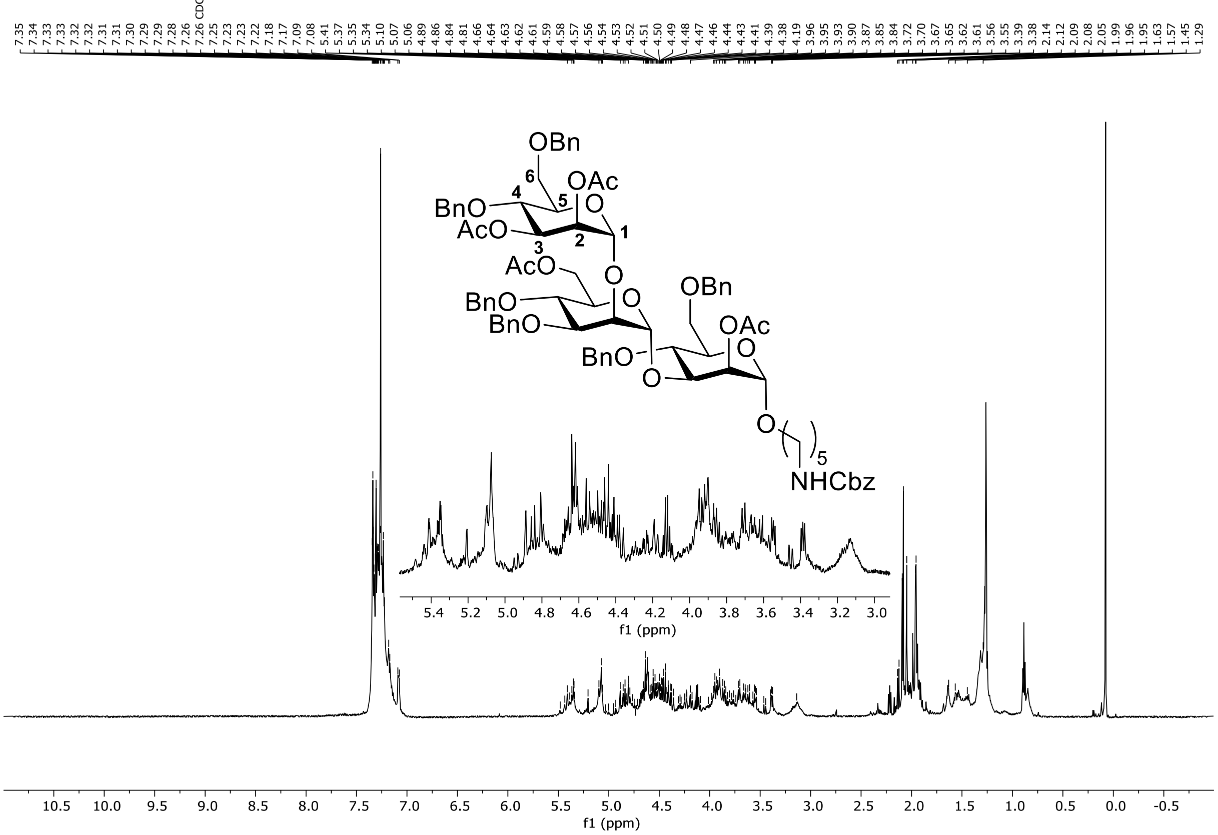

^{13}C NMR, 151 MHz, $CDCl_3$ – Crude Compound S11

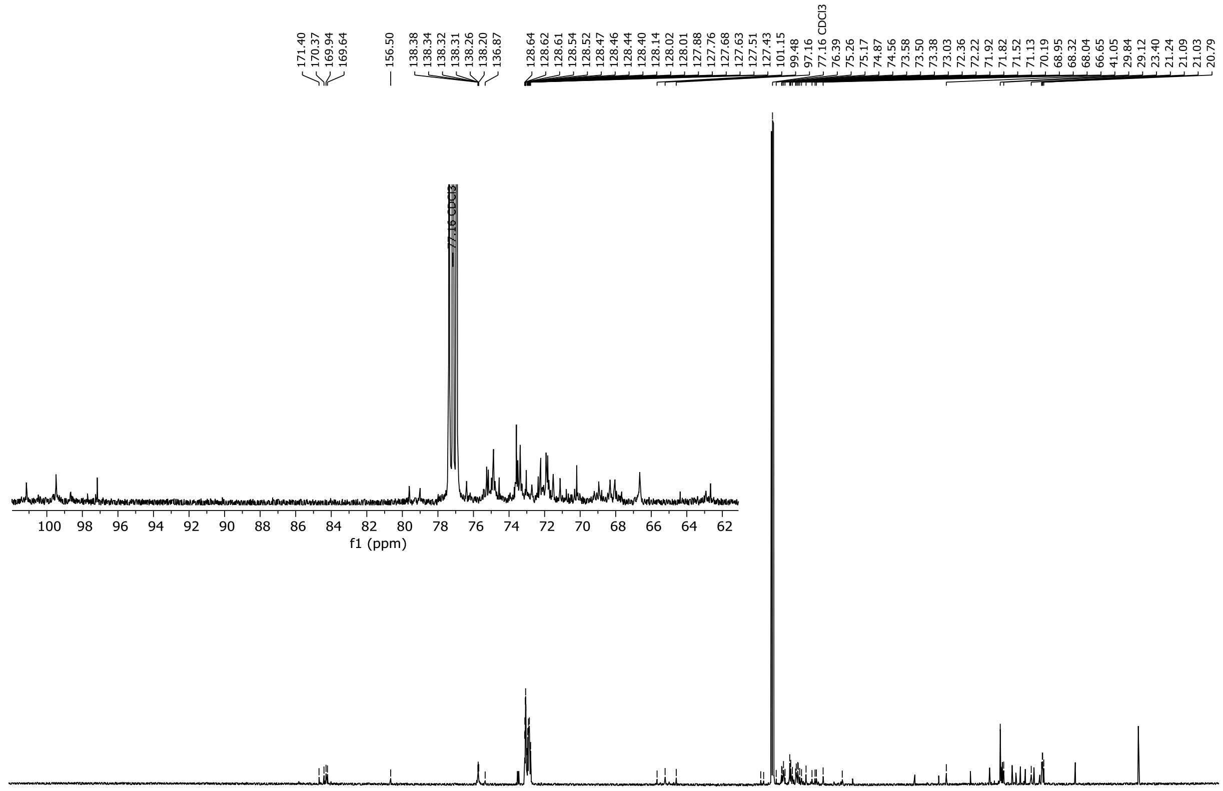

^{1}H-^{13}C gHSQC NMR, $CDCl_3$ – Carbohydrate Region, Crude Compound S11

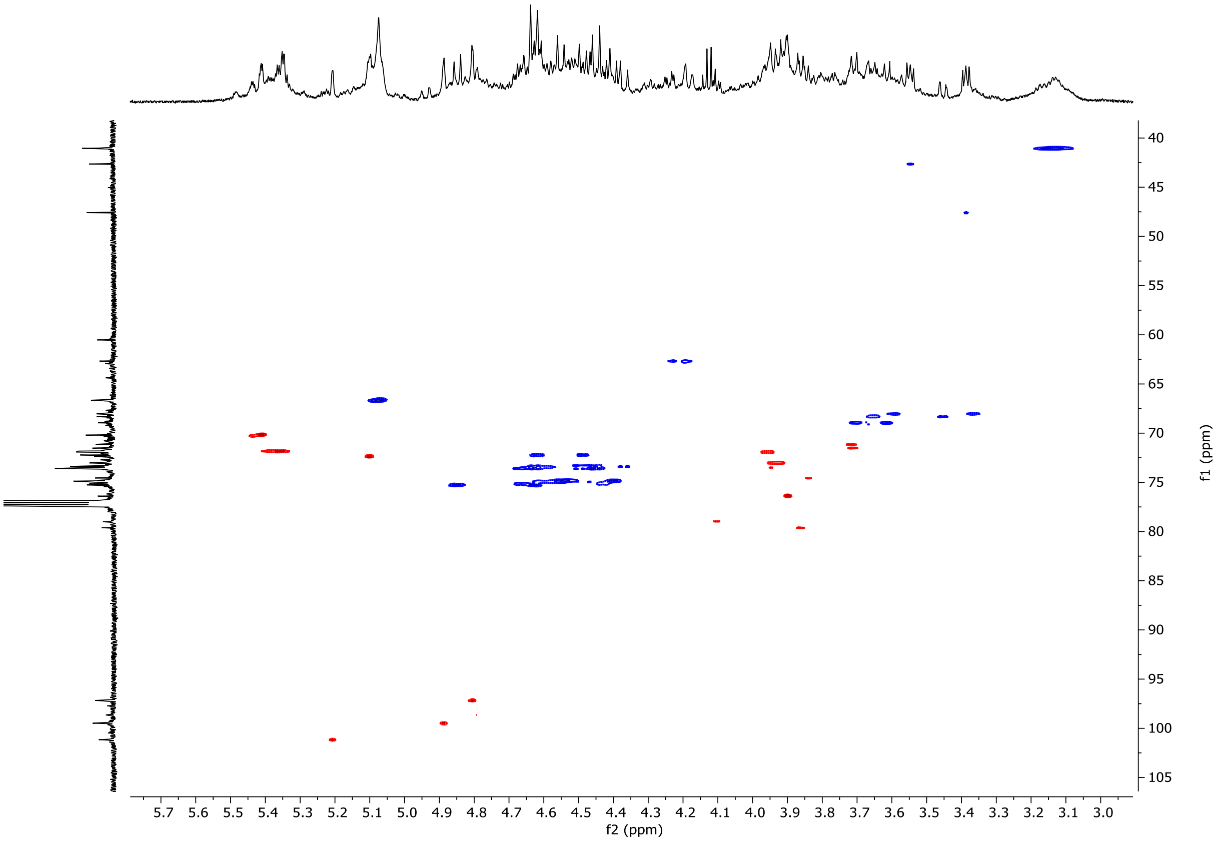

^{1}H NMR, 599 MHz, $CDCl_3$ – Compound 19

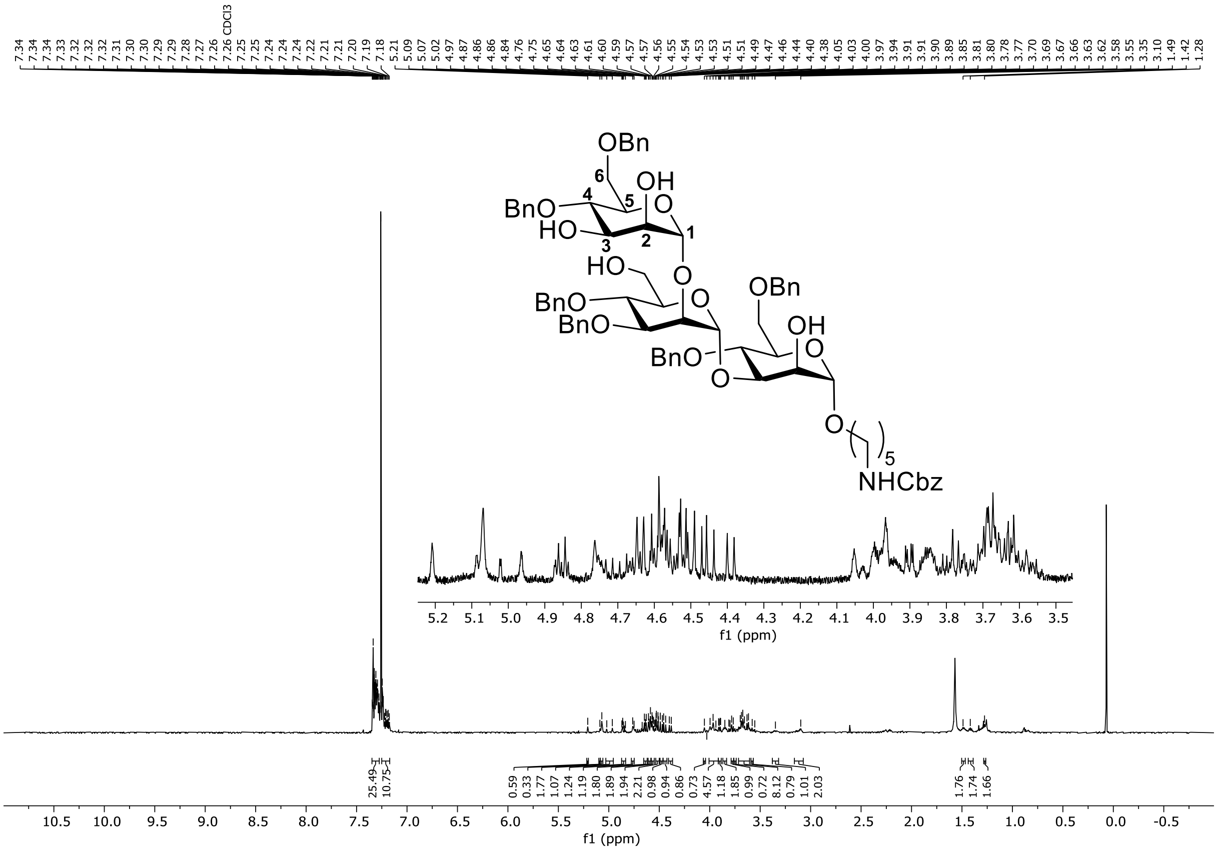

^{13}C NMR, 151 MHz, $CDCl_3$ – Compound 19

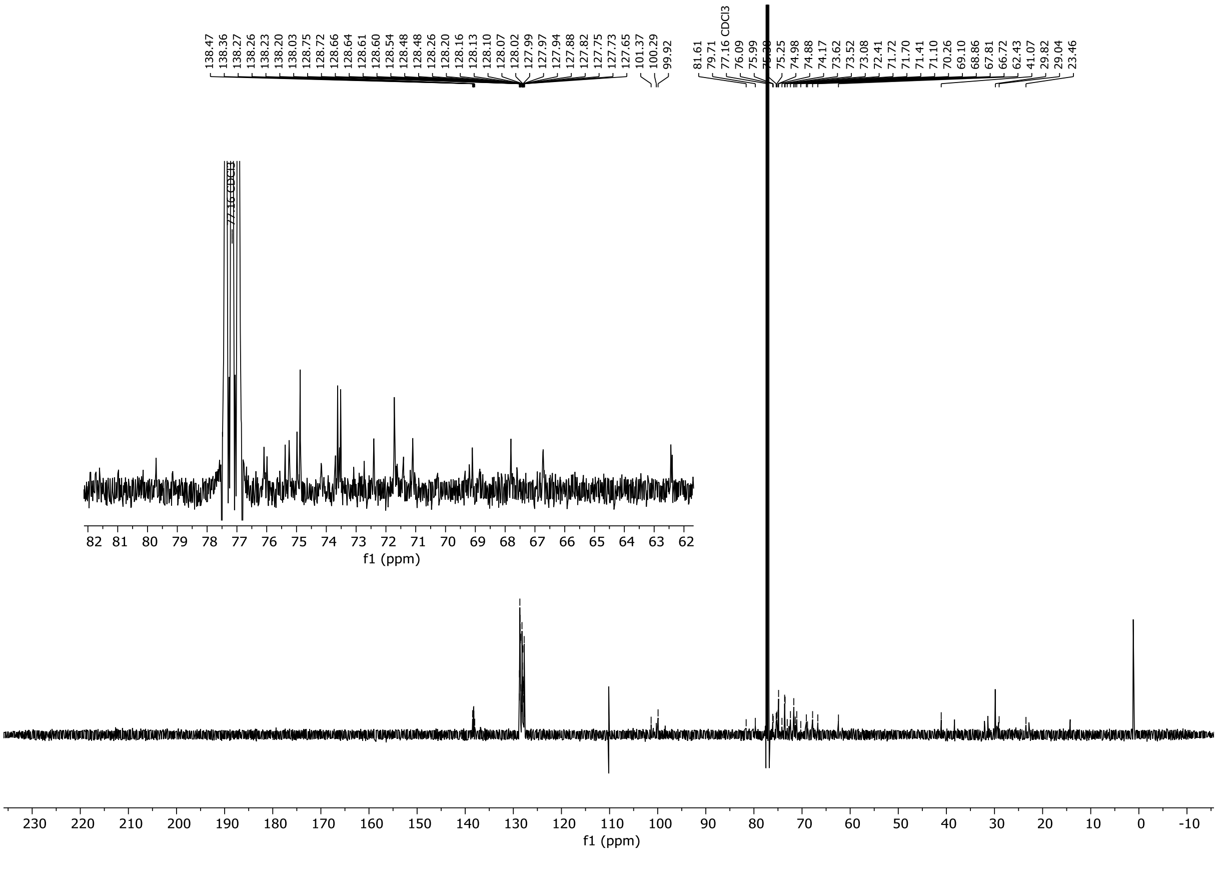

^{1}H-^{13}C gHSQC NMR, $CDCl_3$ – Carbohydrate Region, Compound 19

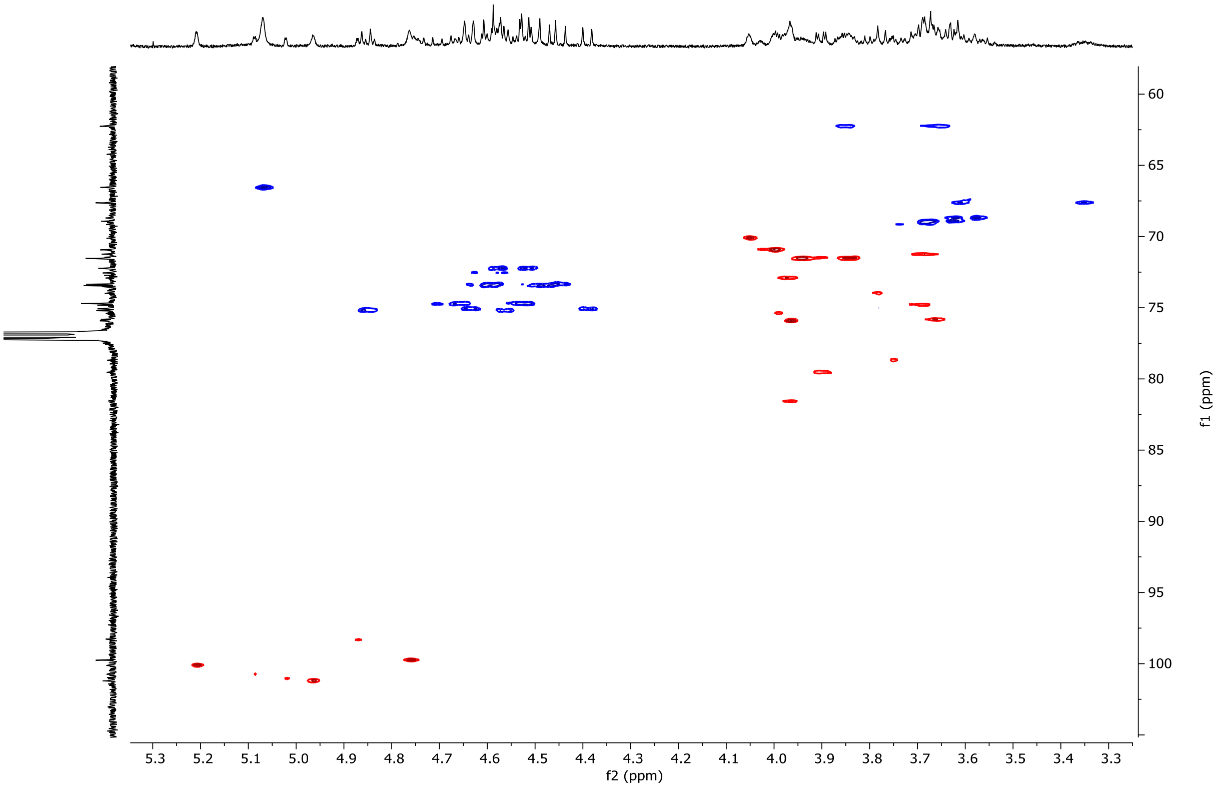

^1H NMR, 599 MHz, D_2O – Compound 25

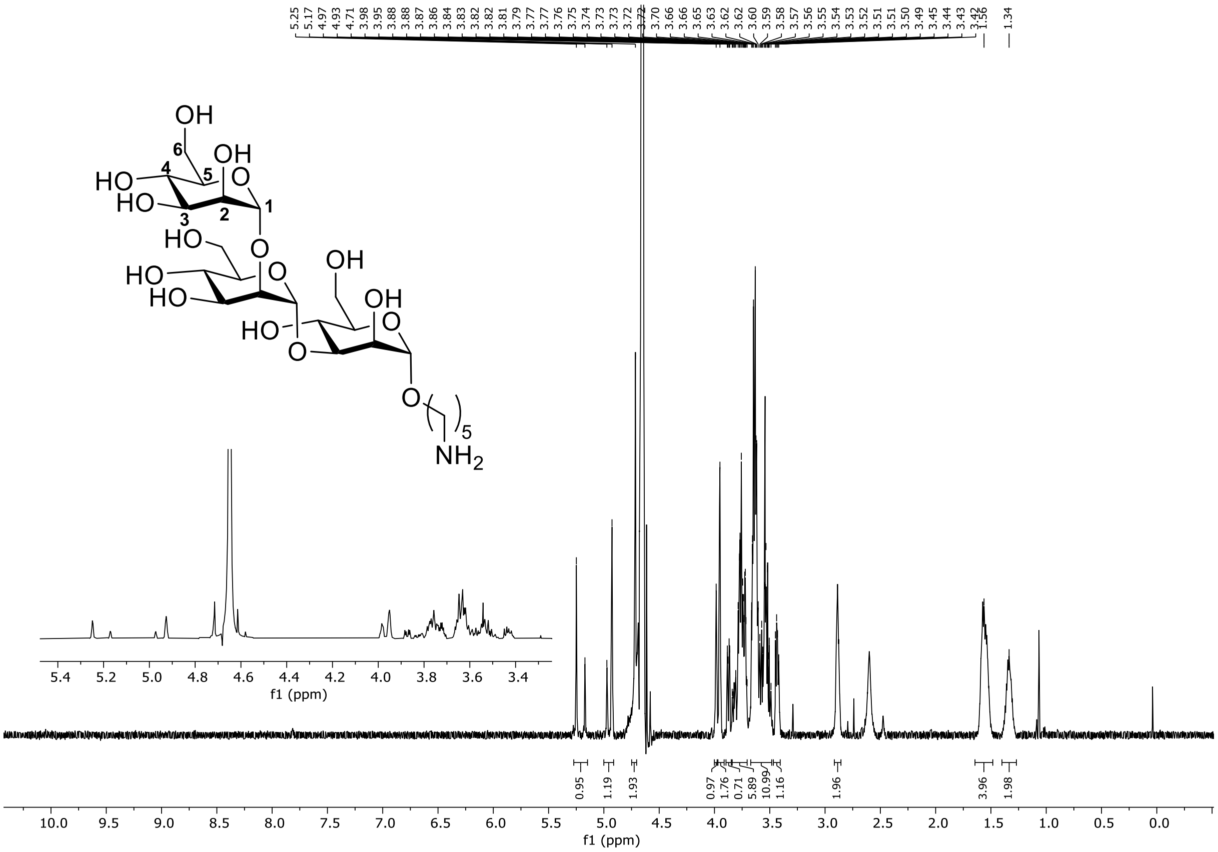

^1H-^{13}C gHSQC NMR, D_2O– Compound 25

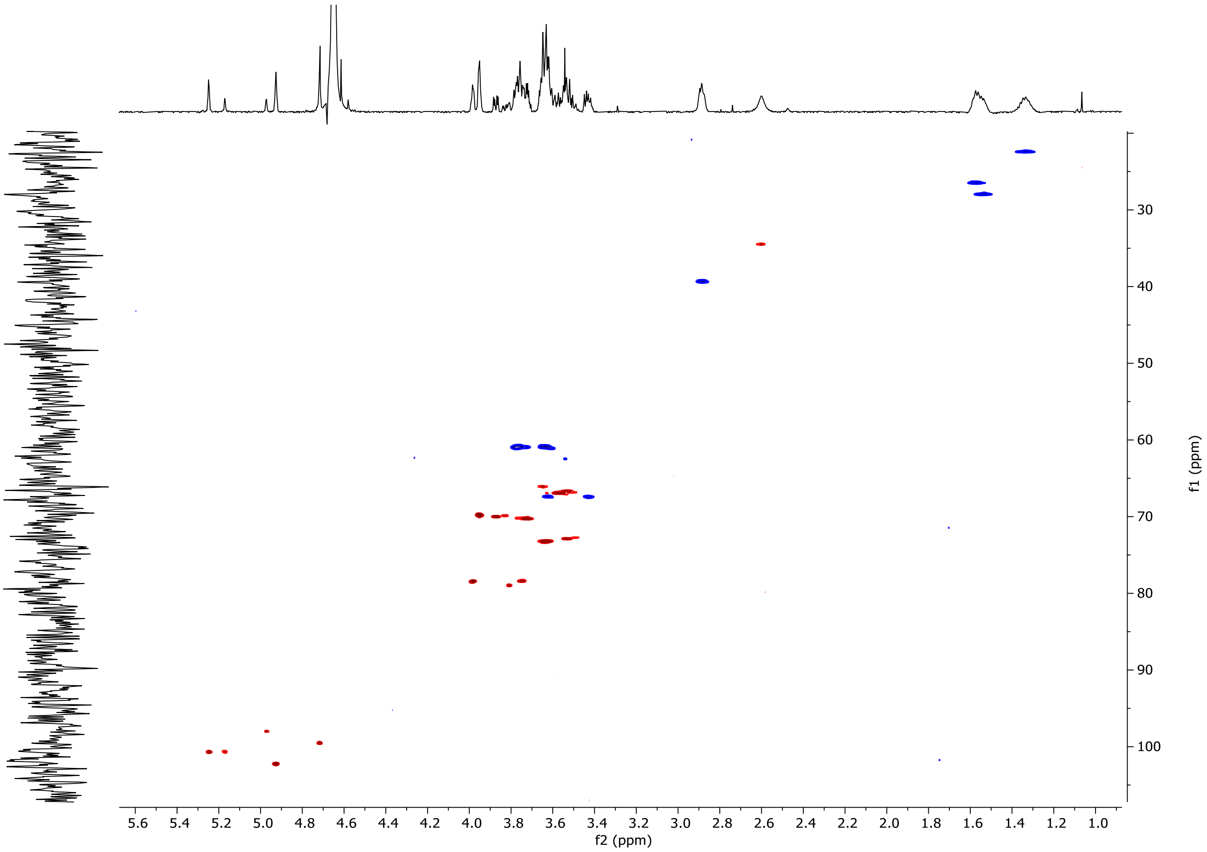

^{1}H NMR, 599 MHz, $CDCl_3$ – Compound 20

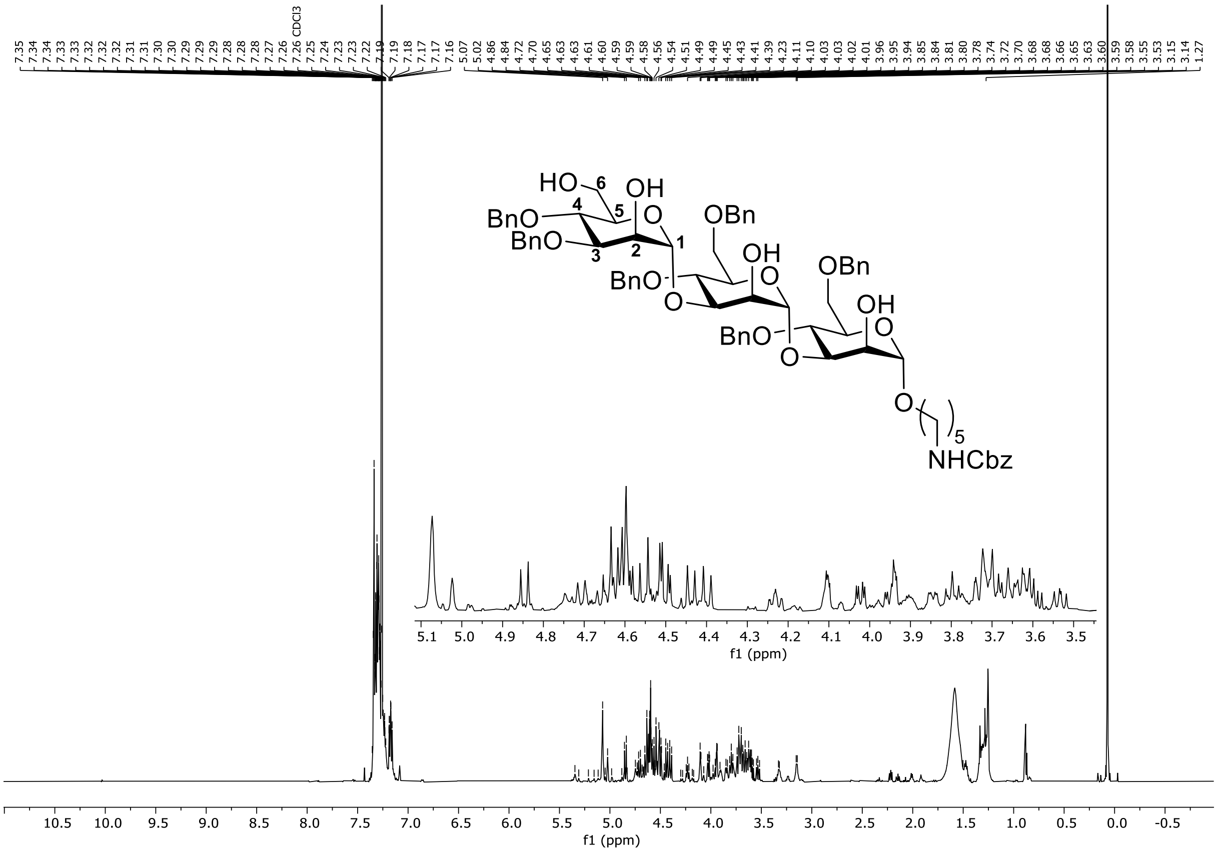

^{13}C NMR, 151 MHz, $CDCl_3$ – Compound 20

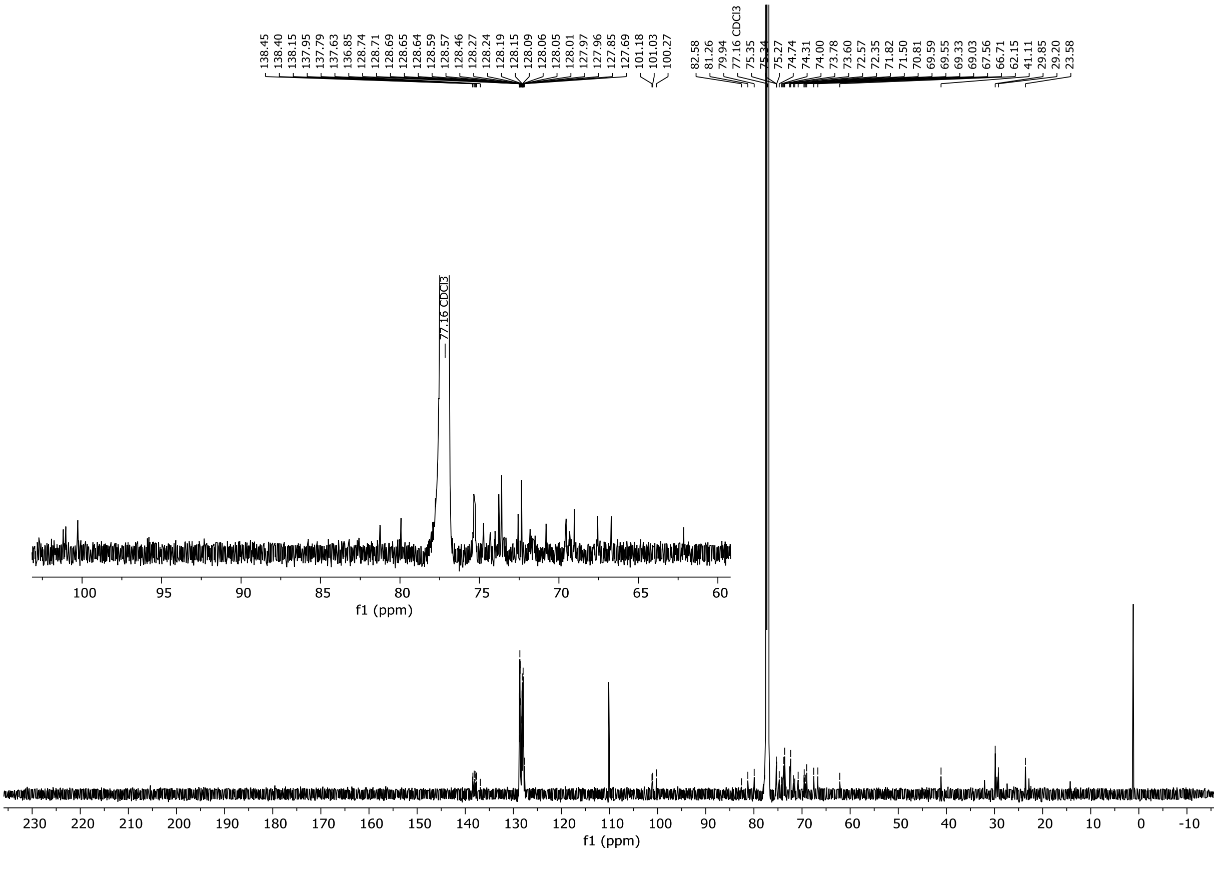

^{1}H-^{13}C gHSQC NMR, $CDCl_3$ – Carbohydrate Region Compound 20

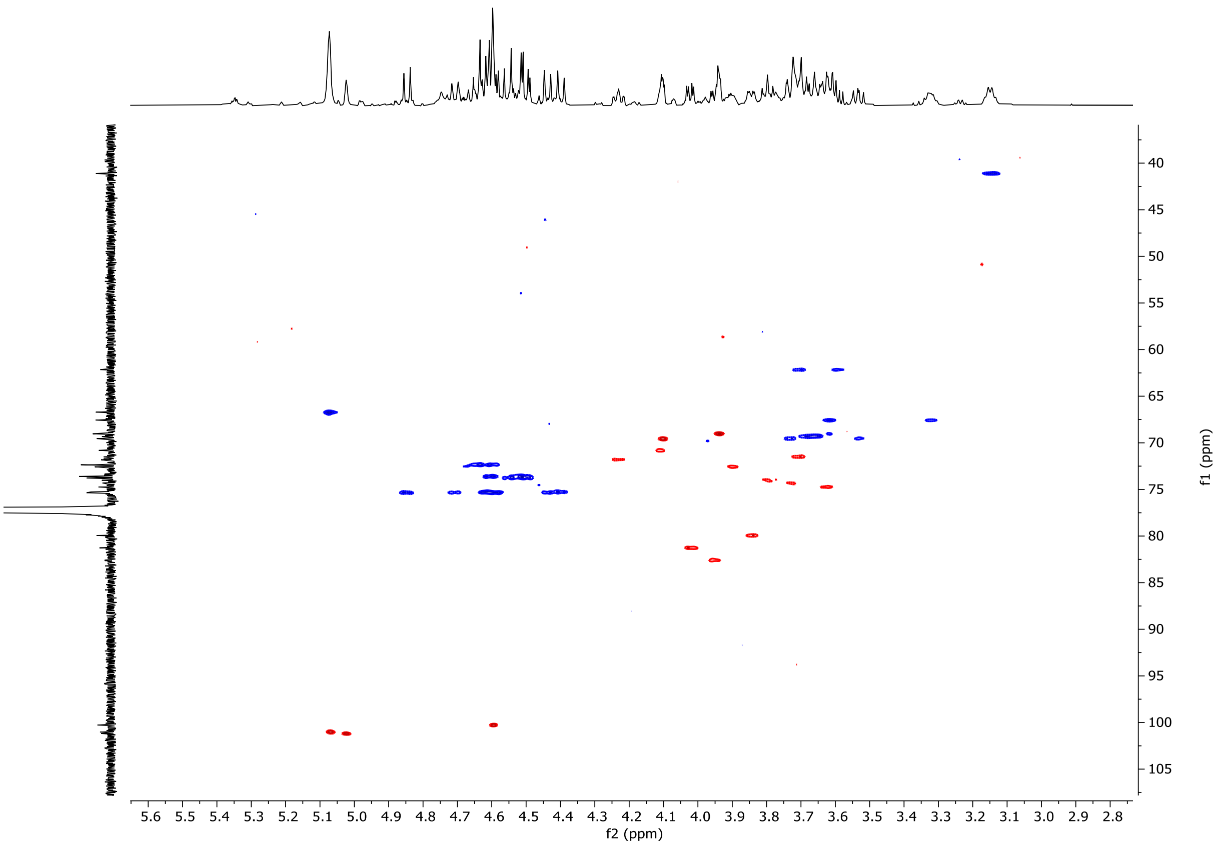

^{1}H NMR, 599 MHz, D_2O – Compound 26

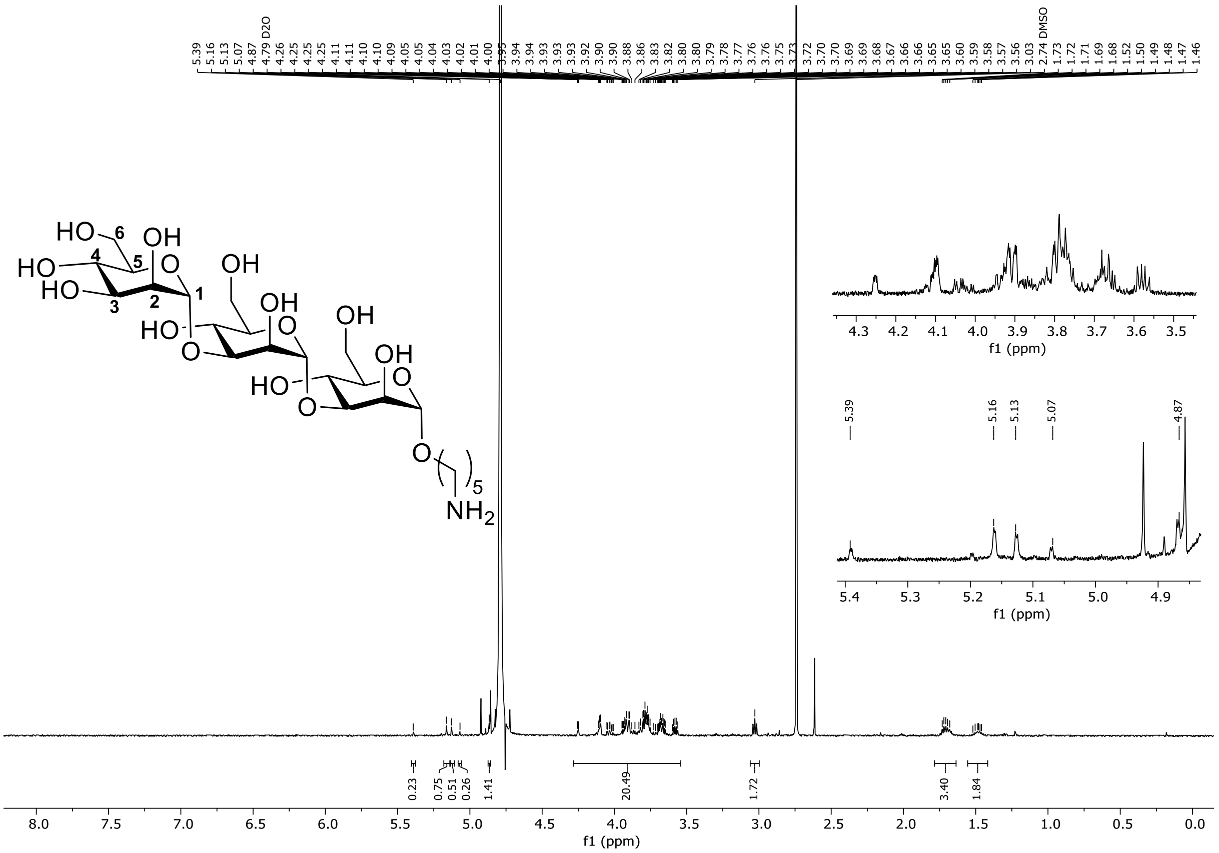

^{1}H-^{13}C gHSQC NMR, D_2O – Compound 26

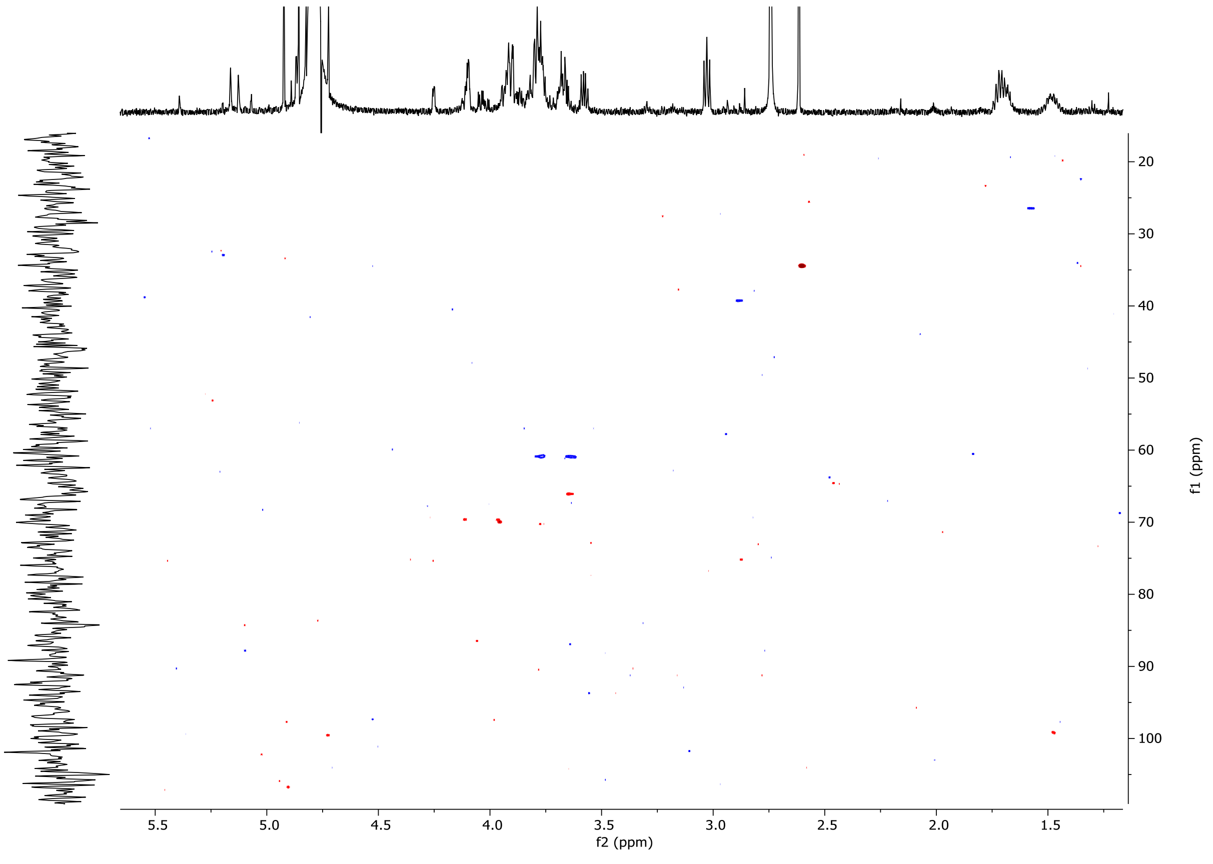

^{1}H NMR, 599 MHz, $CDCl_3$ – Crude Compound S13

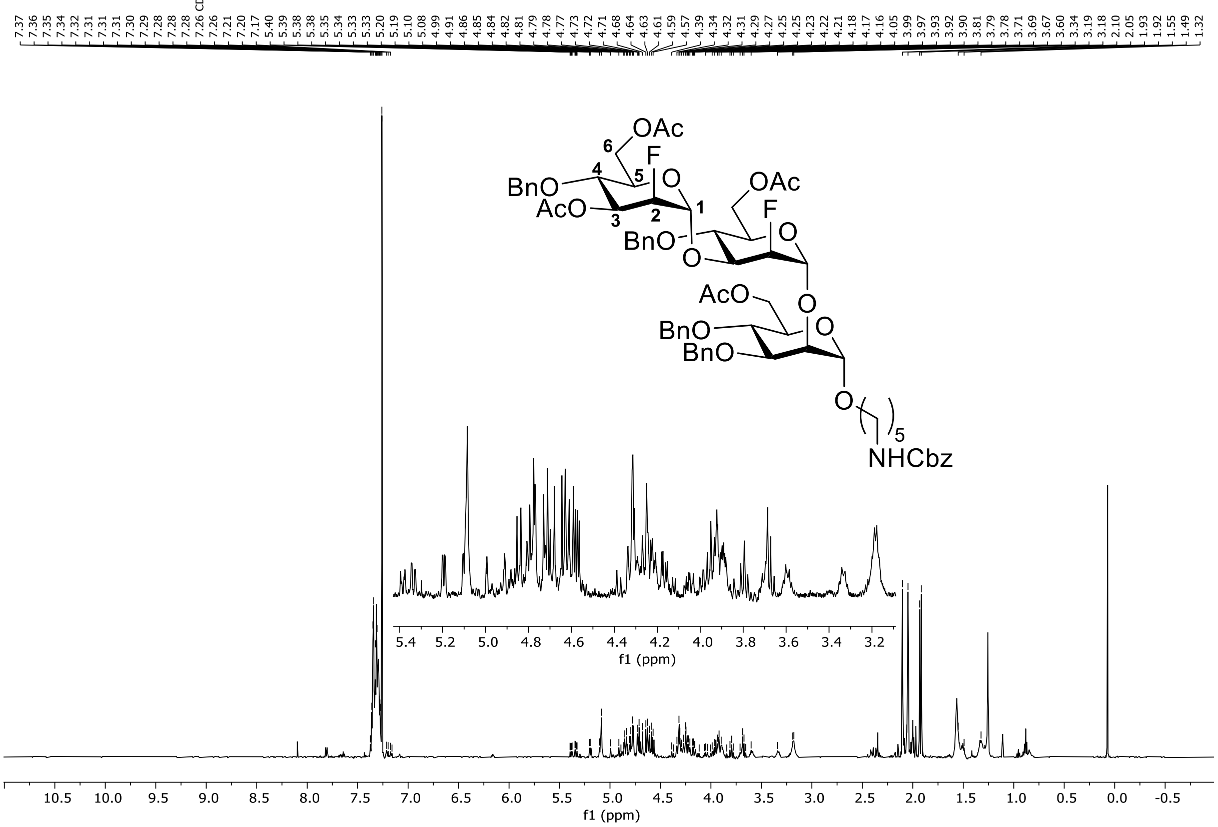

^{13}C NMR, 101 MHz, $CDCl_3$ – Crude Compound S13

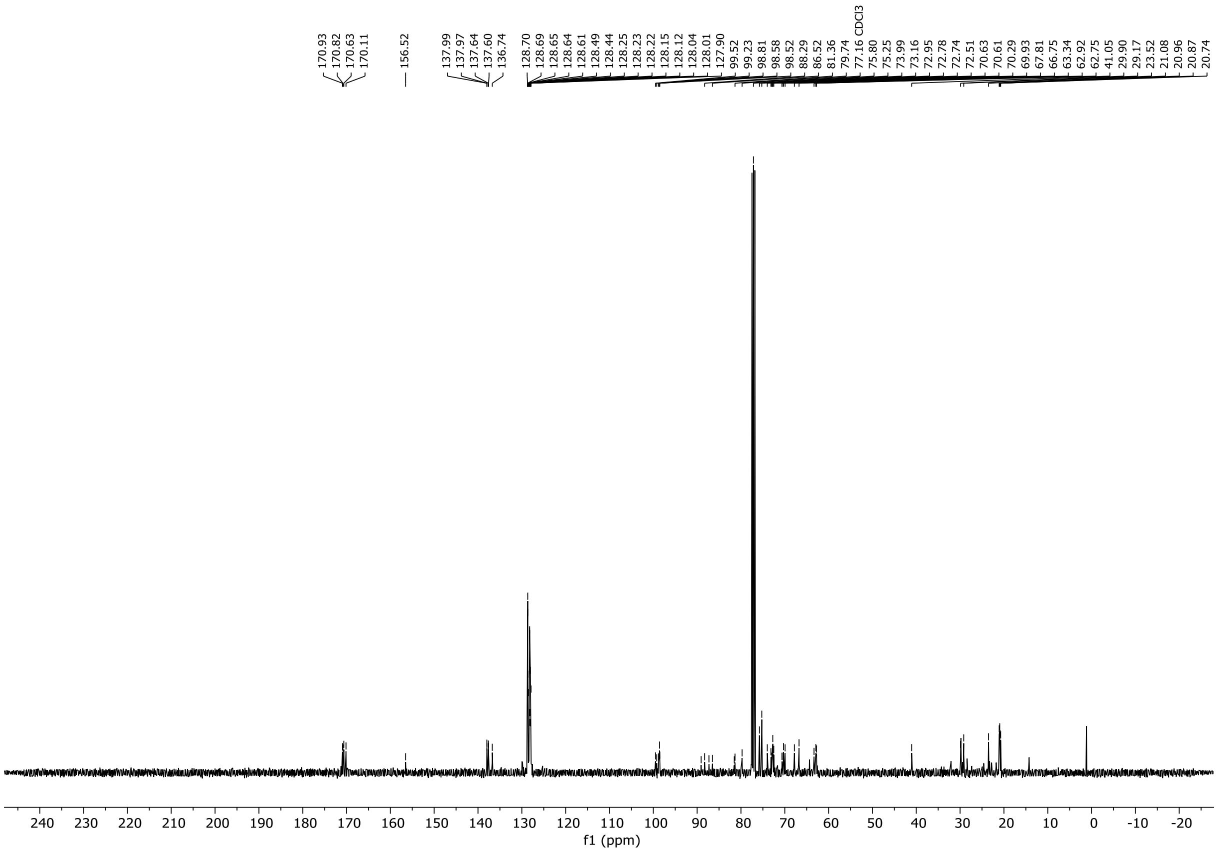

^{19}F NMR, 564 MHz, $CDCl_3$ – Crude Compound S13

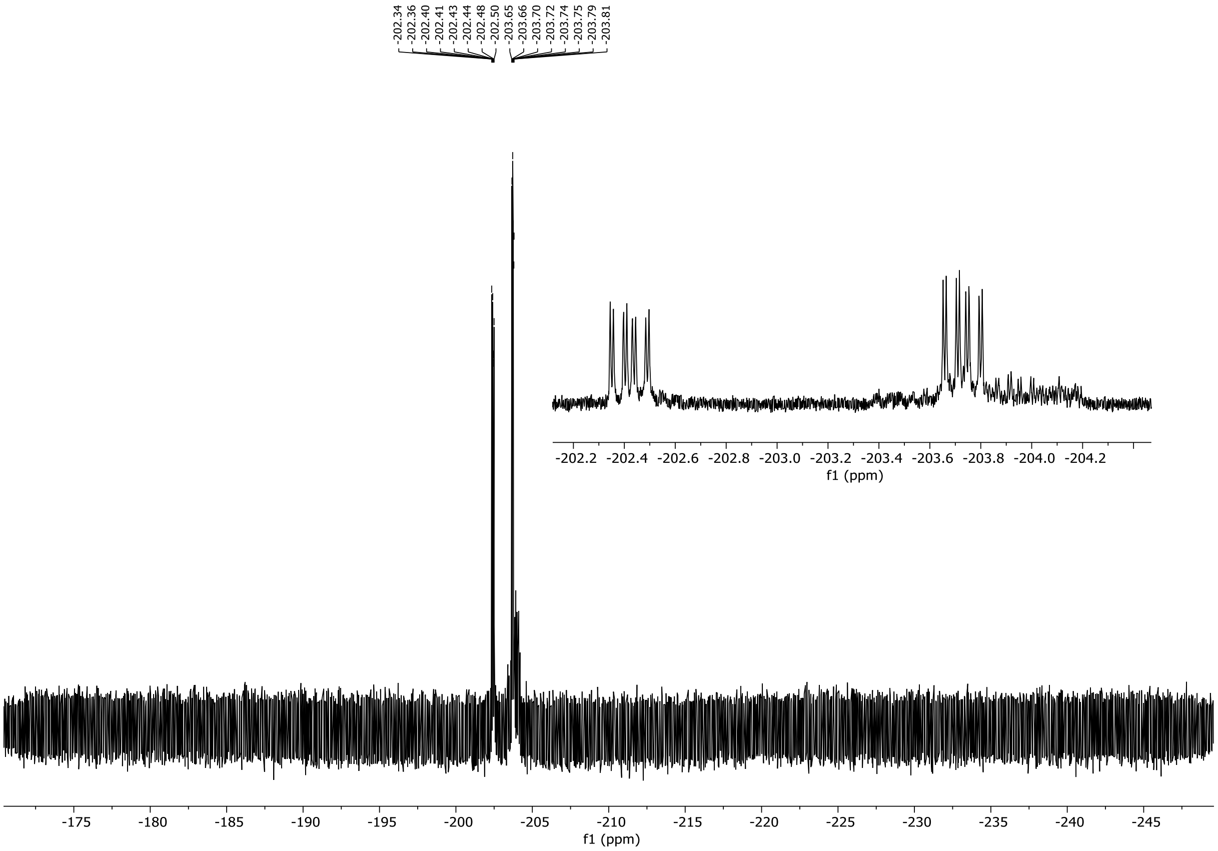

^{1}H-^{13}C gHSQC NMR, $CDCl_3$ – Carbohydrate Region, Crude Compound S13

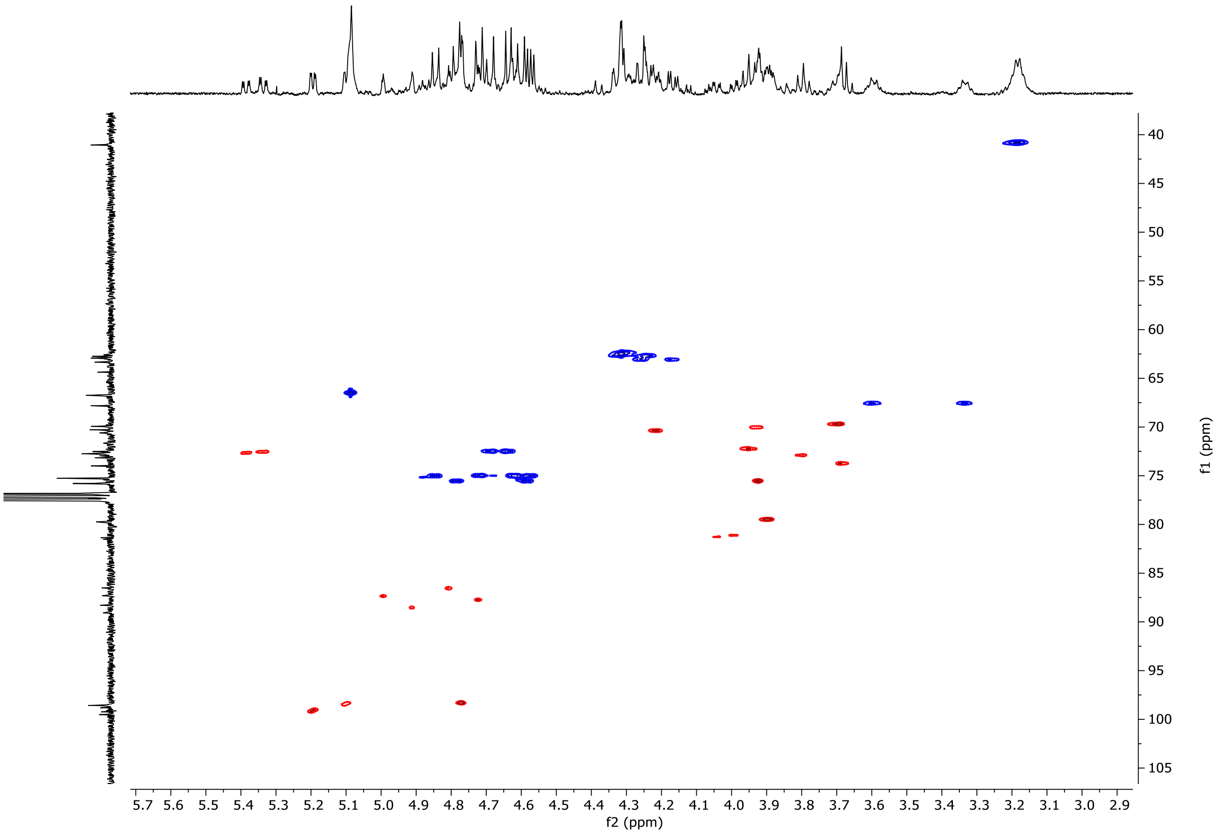

^{1}H NMR, 500 MHz, $CDCl_3$ – Compound 21

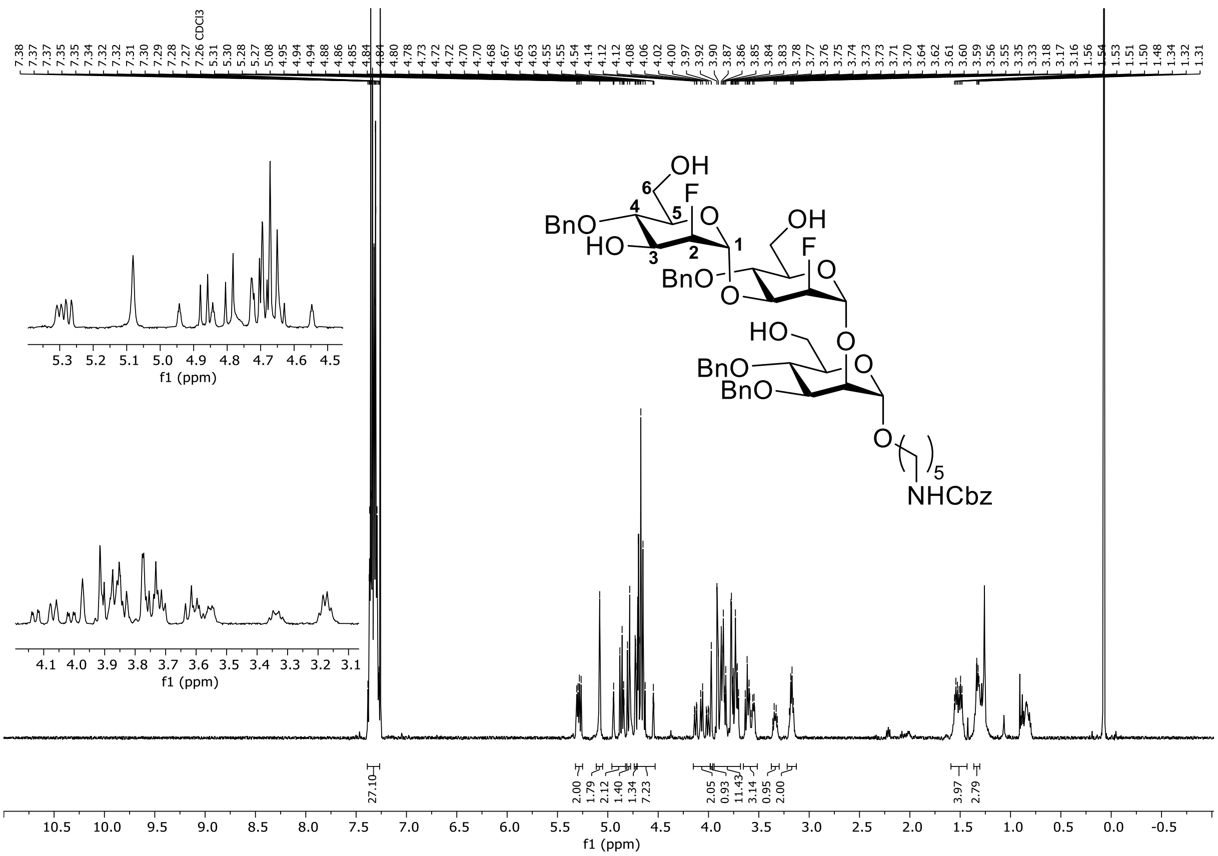

^{13}C NMR, 126 MHz, $CDCl_3$ – Compound 21

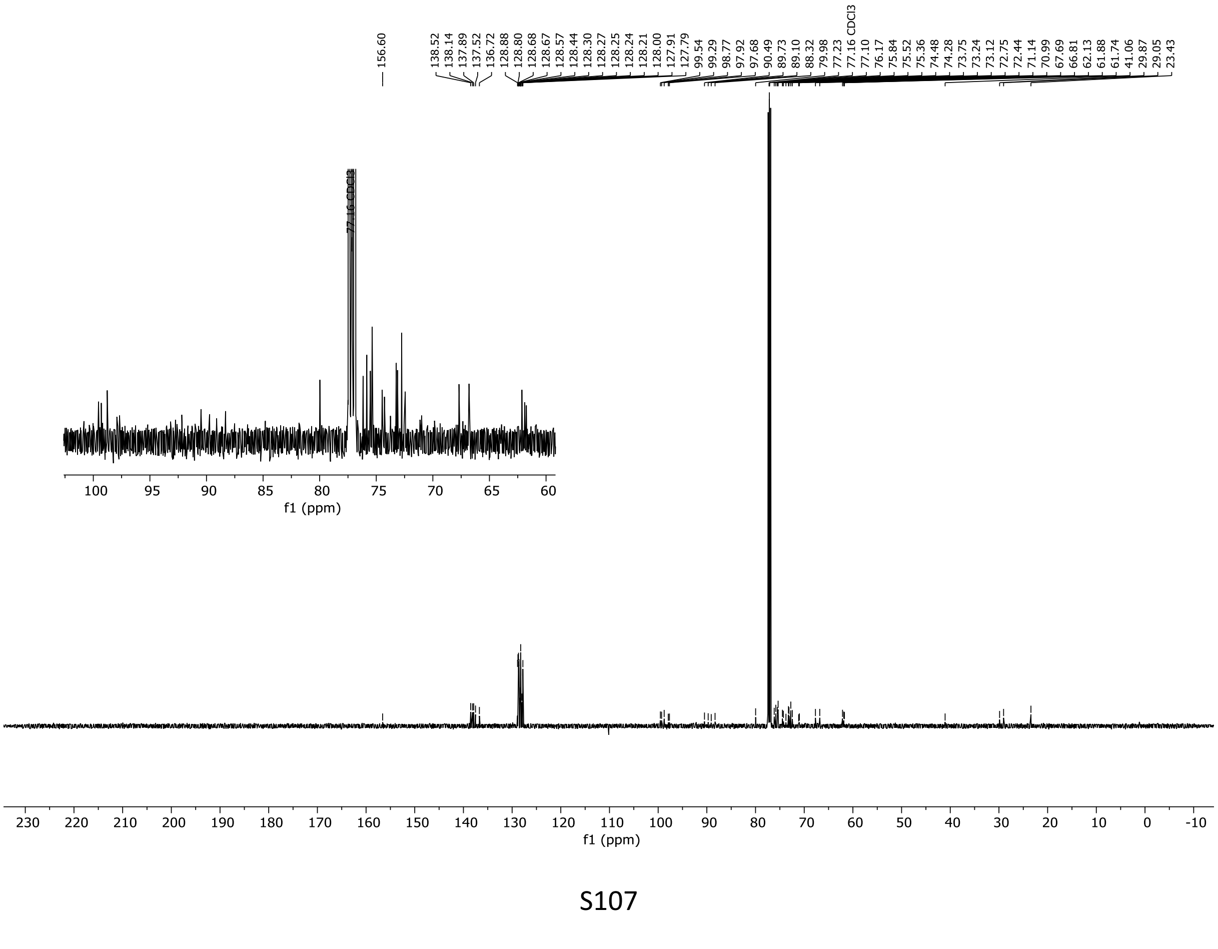

^{19}F NMR, 470 MHz, $CDCl_3$ – Compound 21

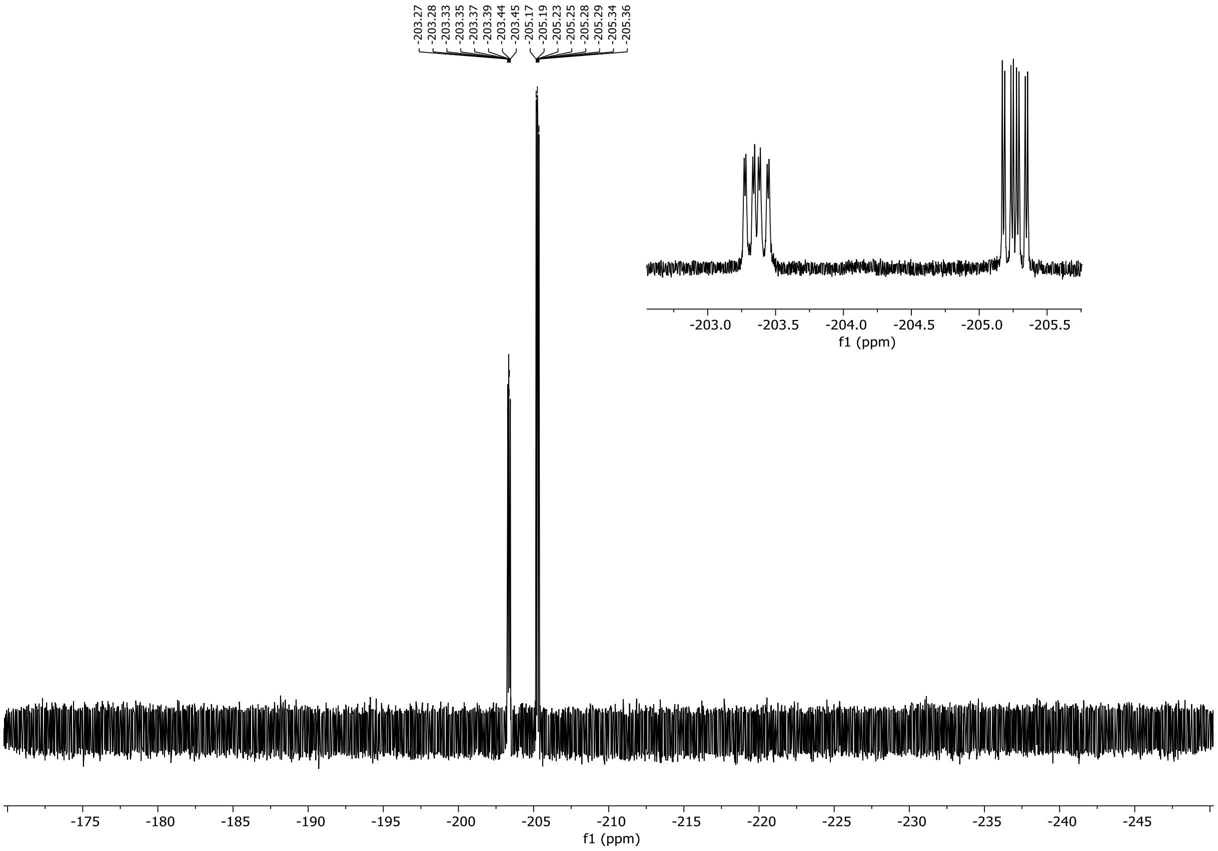

^{1}H-^{13}C gHSQC NMR, $CDCl_3$ – Carbohydrate Region, Compound 21

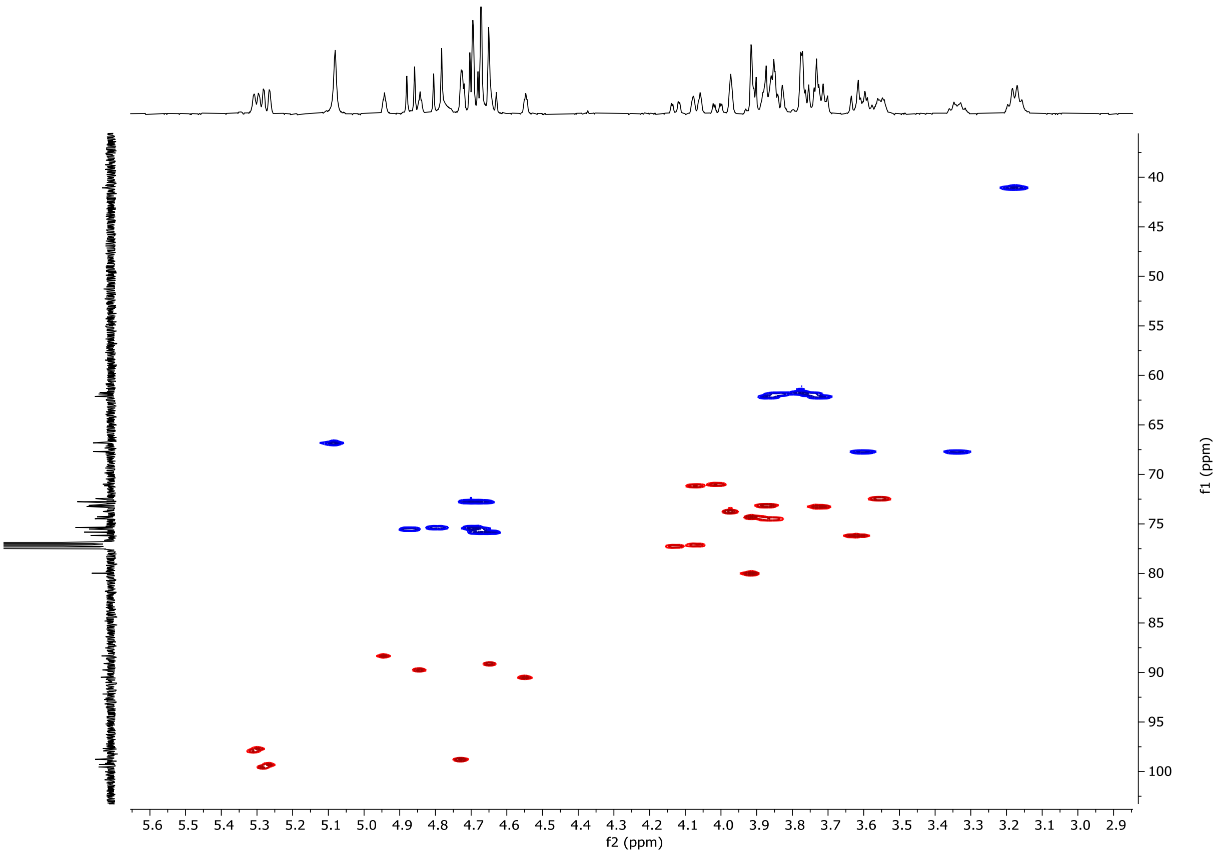

^{1}H NMR, 599 MHz, D_2O – Compound 27

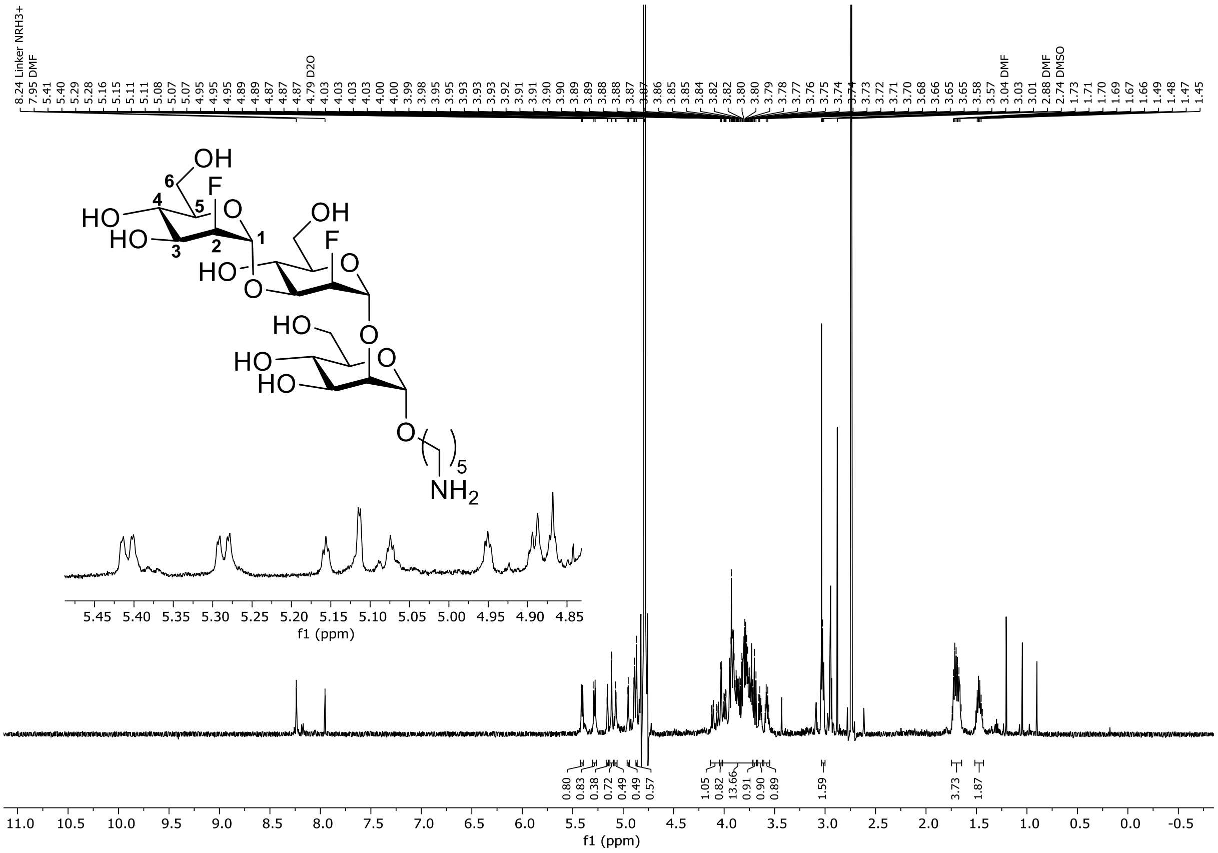

^{13}C {^{1}H,^{19}F} NMR, 151 MHz, D_2O – Compound 27

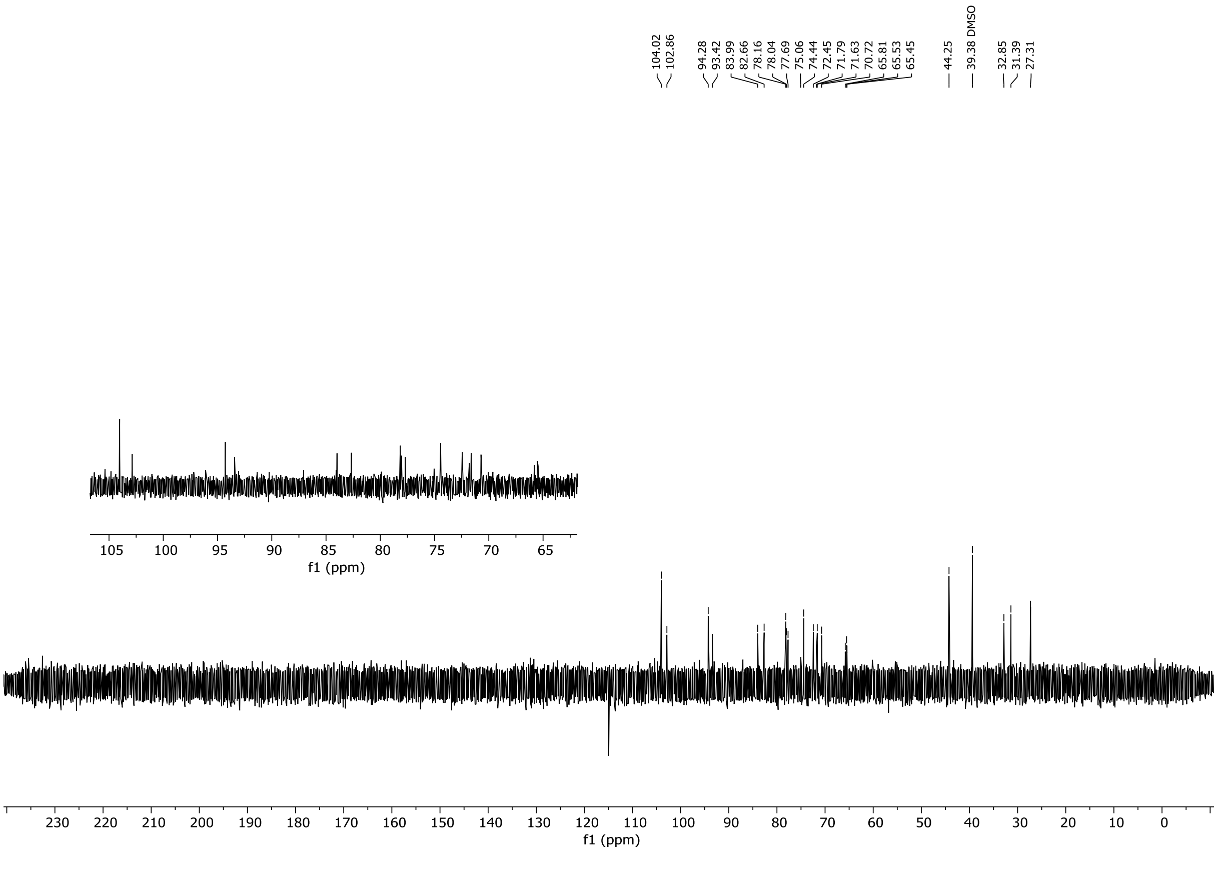

^{19}F NMR, 564 MHz, D_2O – Compound 27

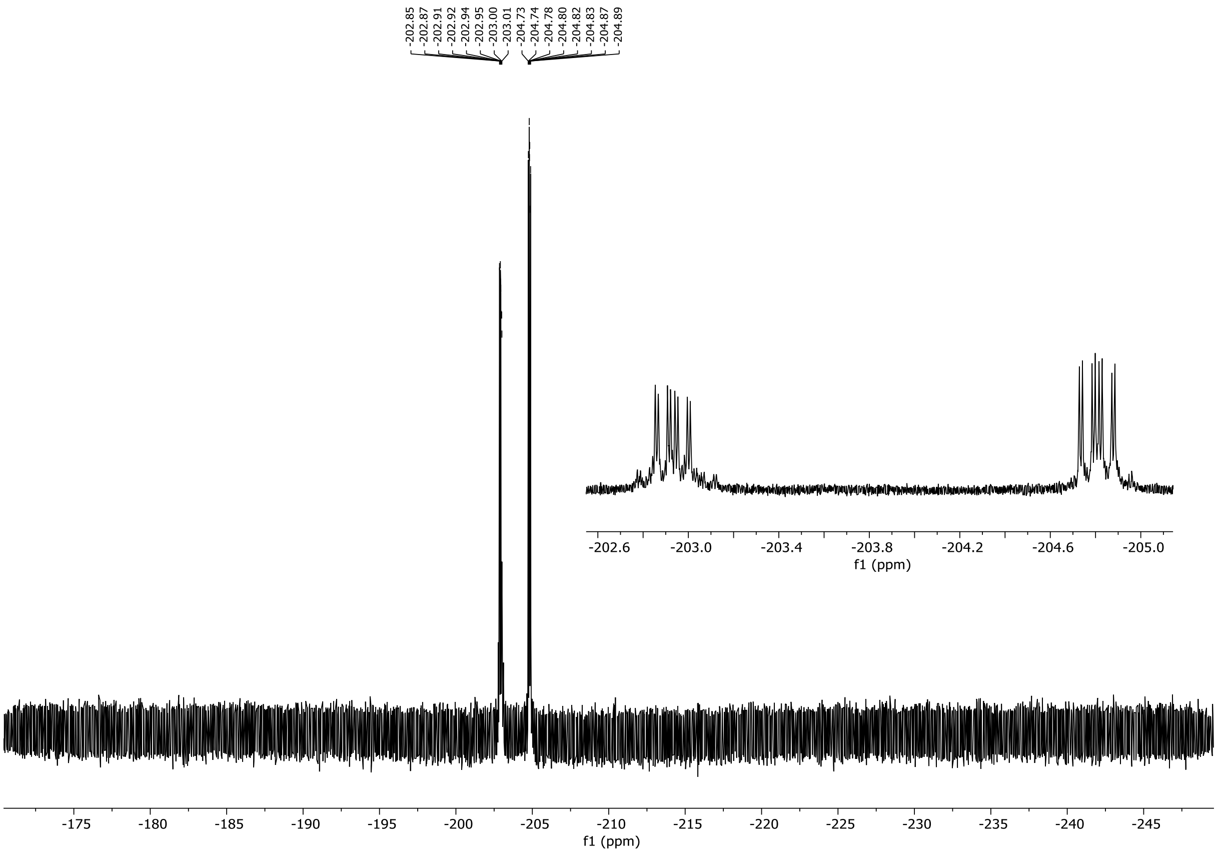

^{1}H-^{13}C gHSQC NMR, D_2O – Carbohydrate Region, Compound 27

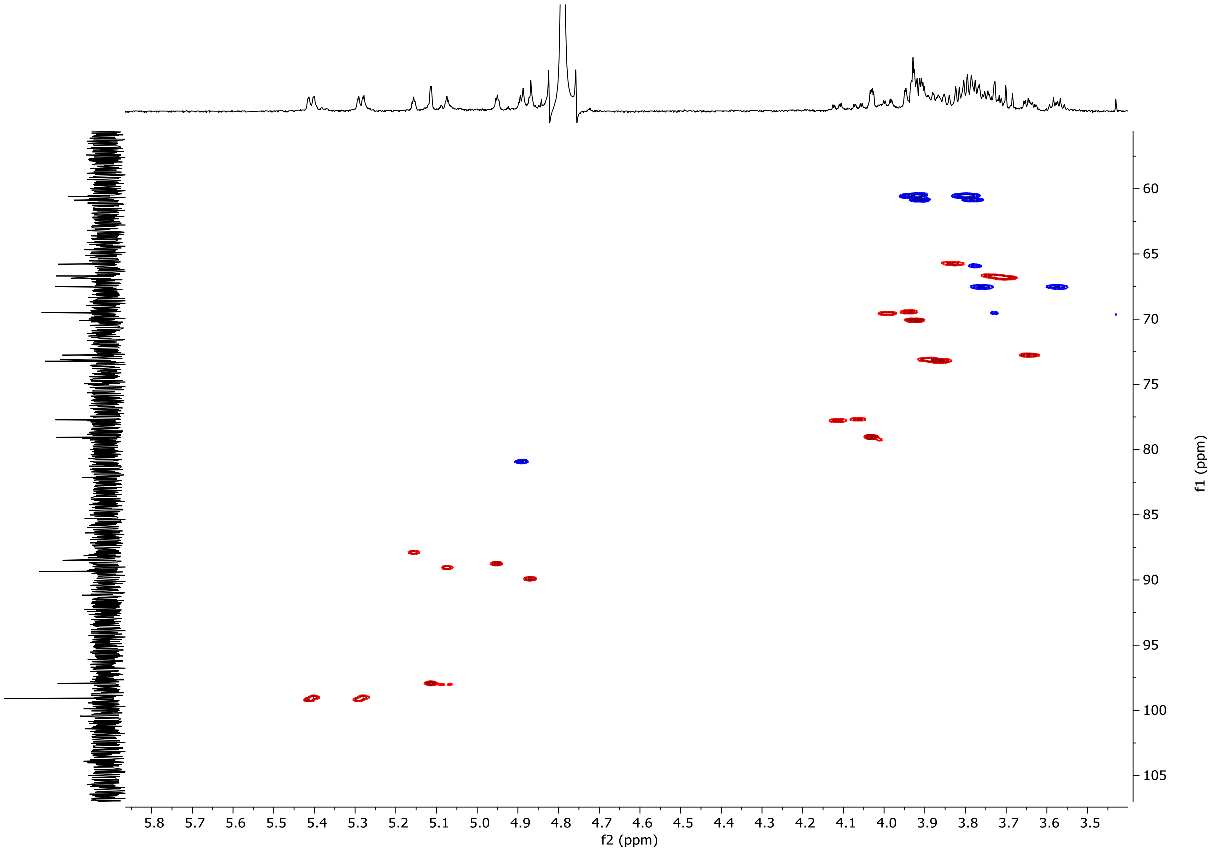

^{1}H NMR, 500 MHz, $CDCl_3$ – Crude Compound S14

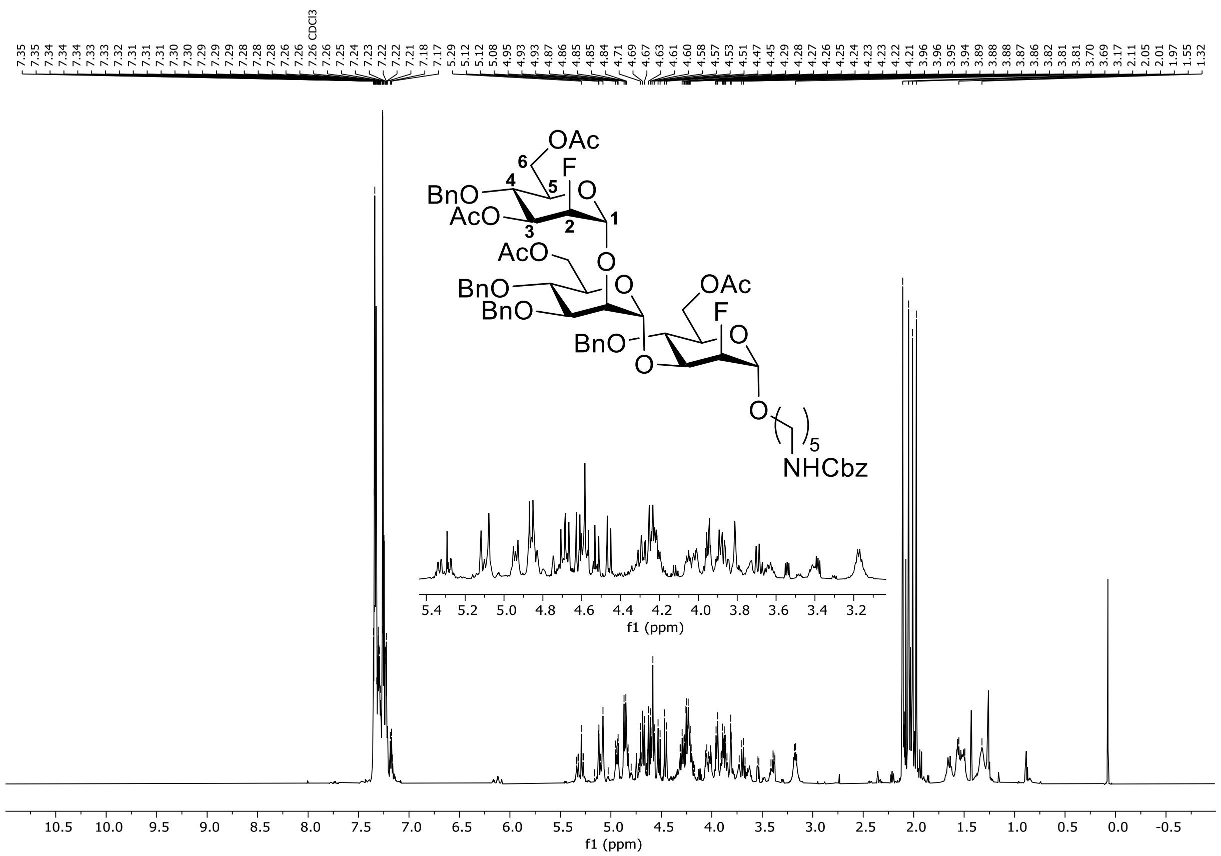

^{13}C NMR, 101 MHz, $CDCl_3$ – Crude Compound S14

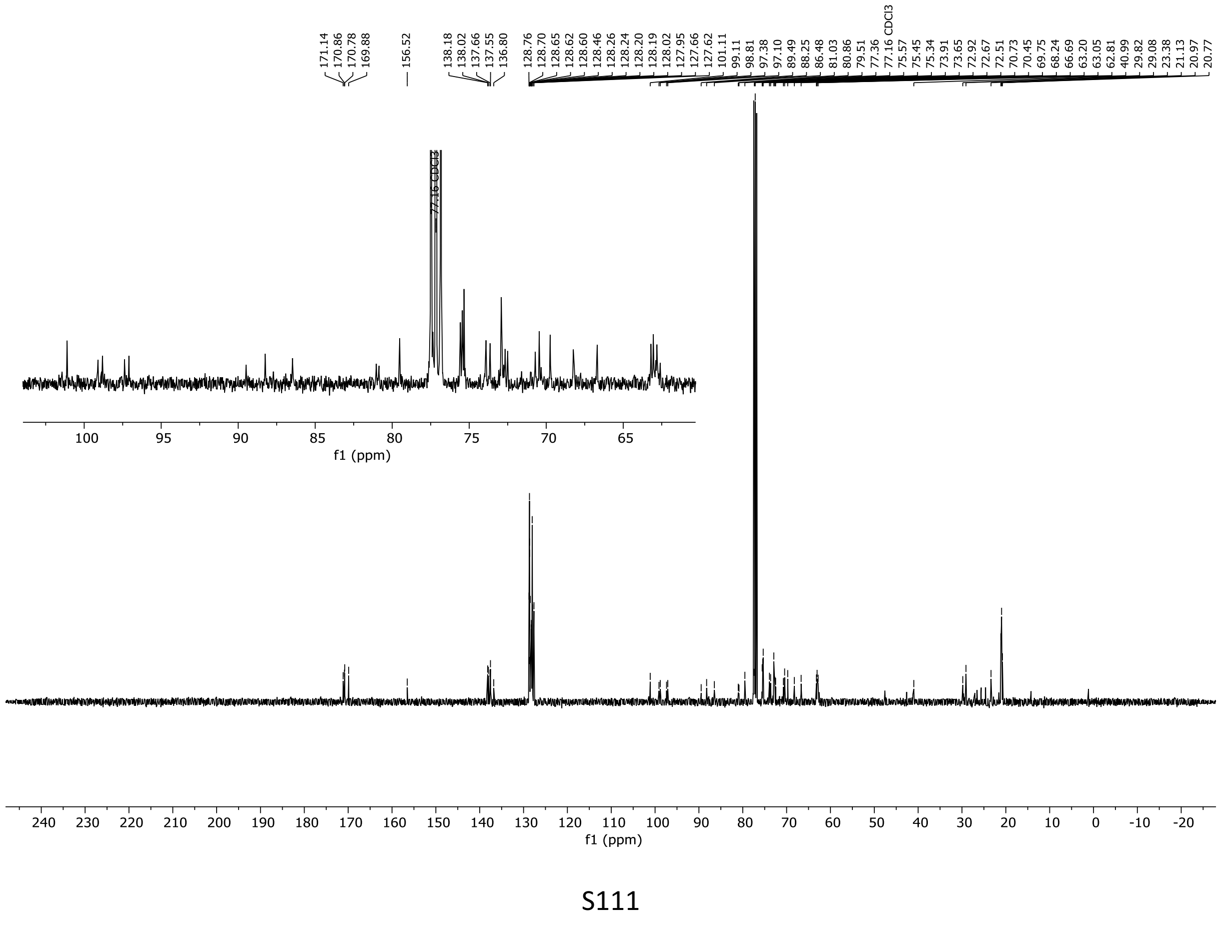

^{19}F NMR, 470 MHz, $CDCl_3$ – Crude Compound S14

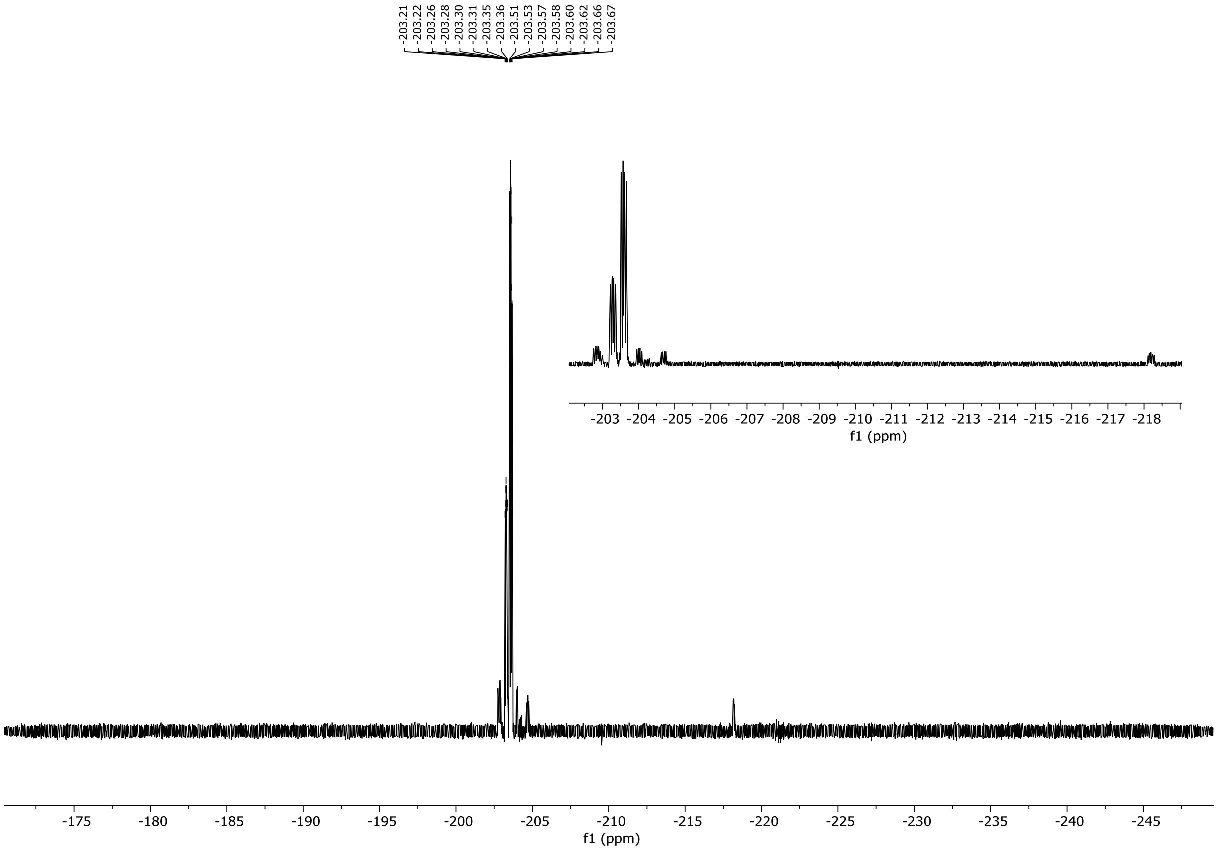

^{1}H-^{13}C gHSQC NMR, $CDCl_3$ – Carbohydrate Region, Crude Compound S14

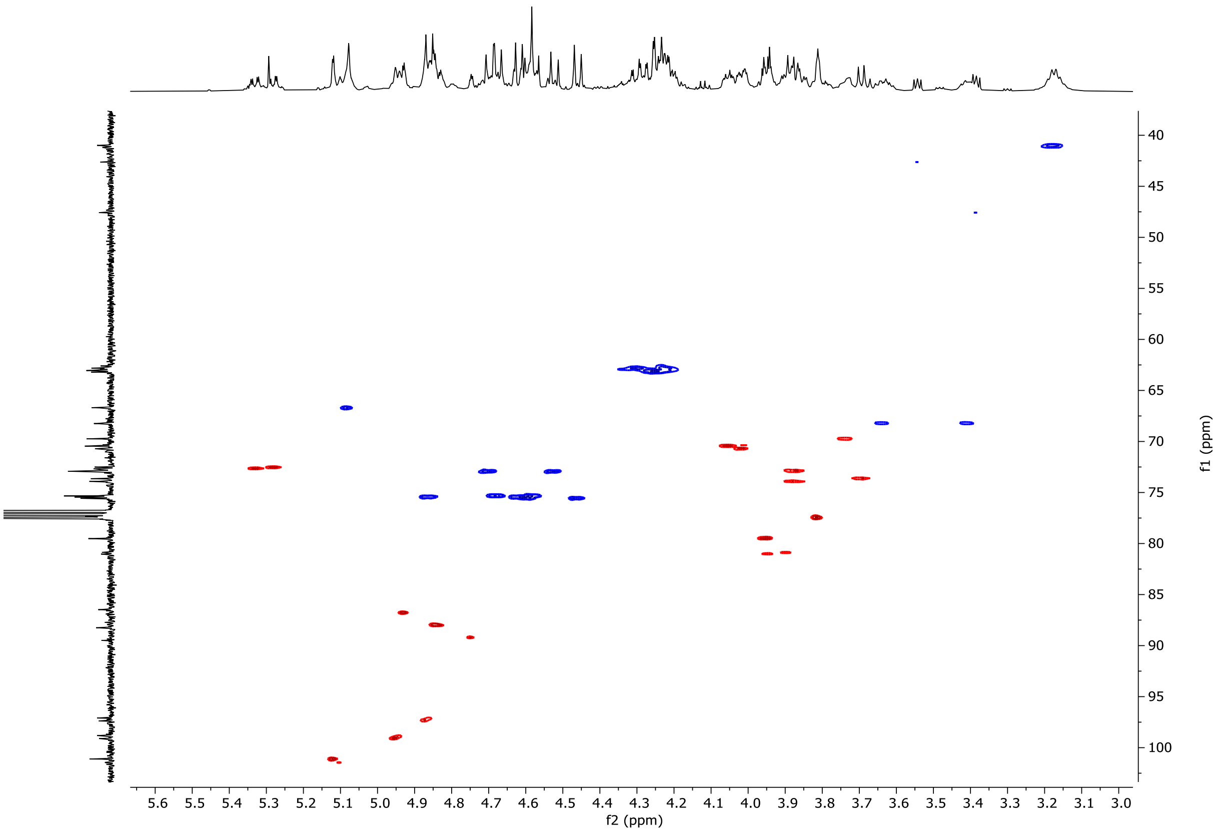

^{1}H NMR, 599 MHz, $CDCl_3$ – Compound 22

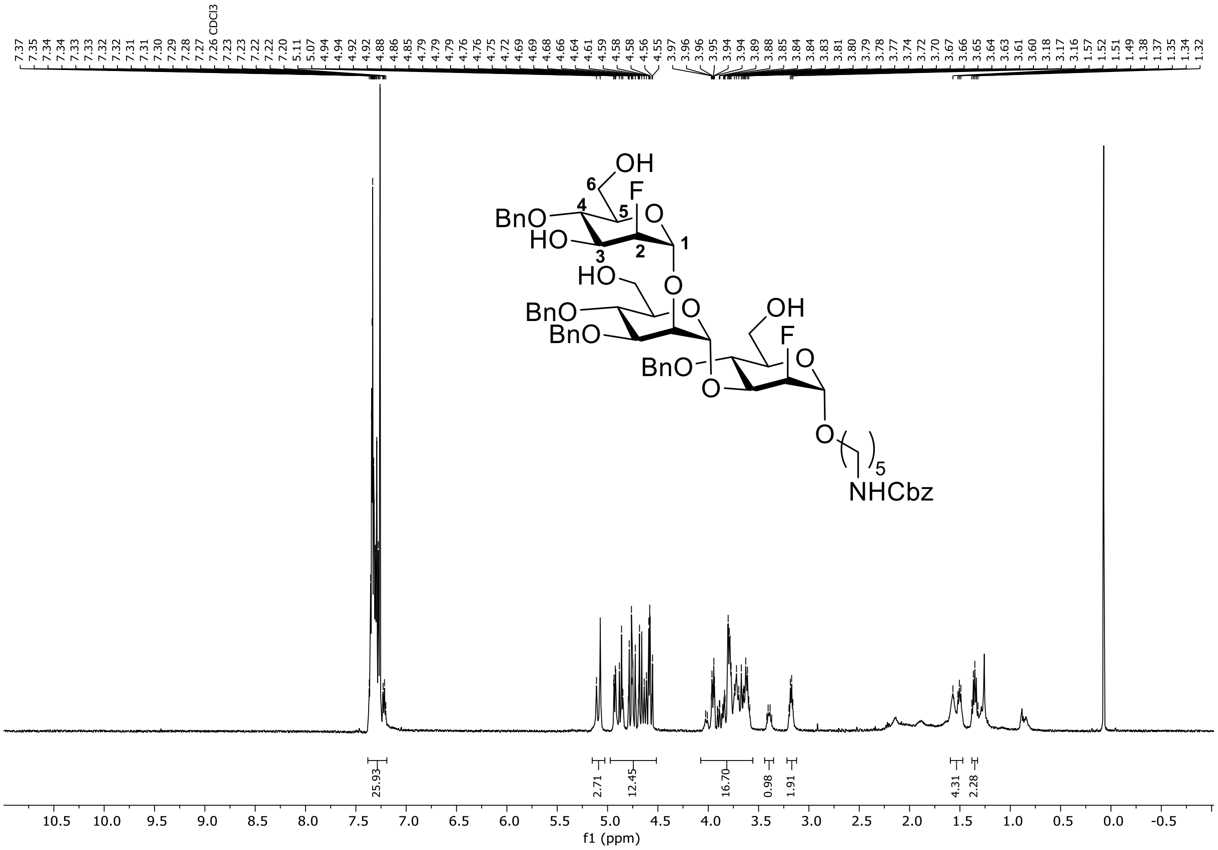

^{13}C NMR, 151 MHz, $CDCl_3$ – Compound 22

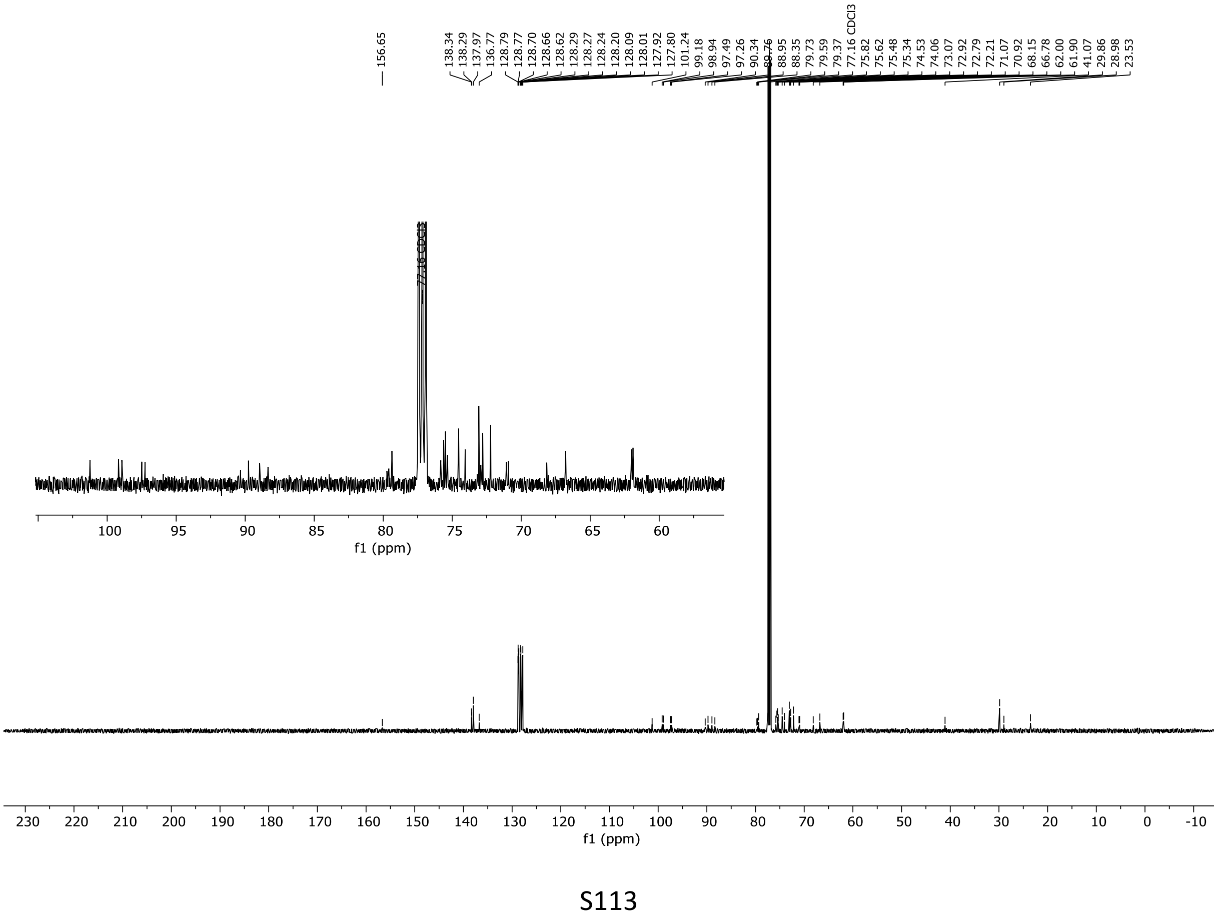

^{19}F NMR, 564 MHz, $CDCl_3$ – Compound 22

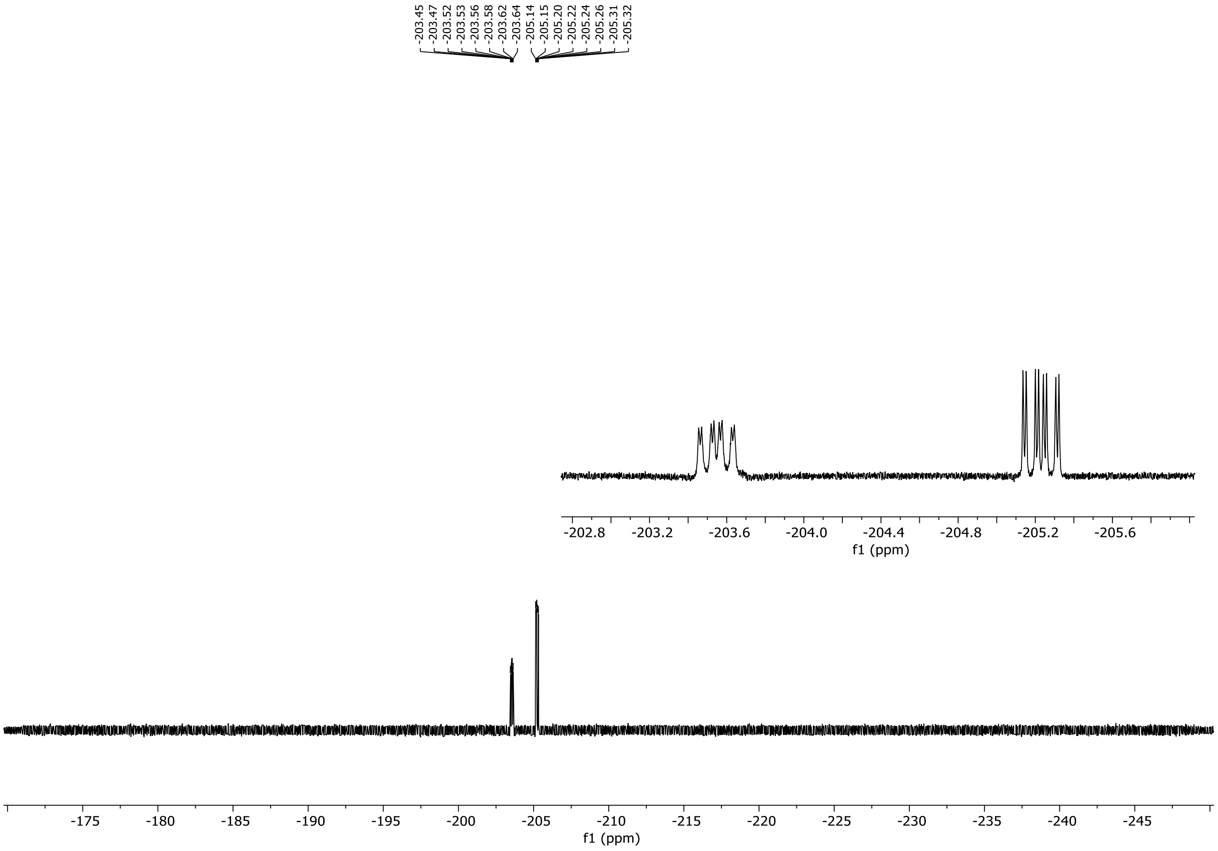

^{1}H-^{13}C gHSQC NMR, $CDCl_3$ – Carbohydrate Region, Compound 22

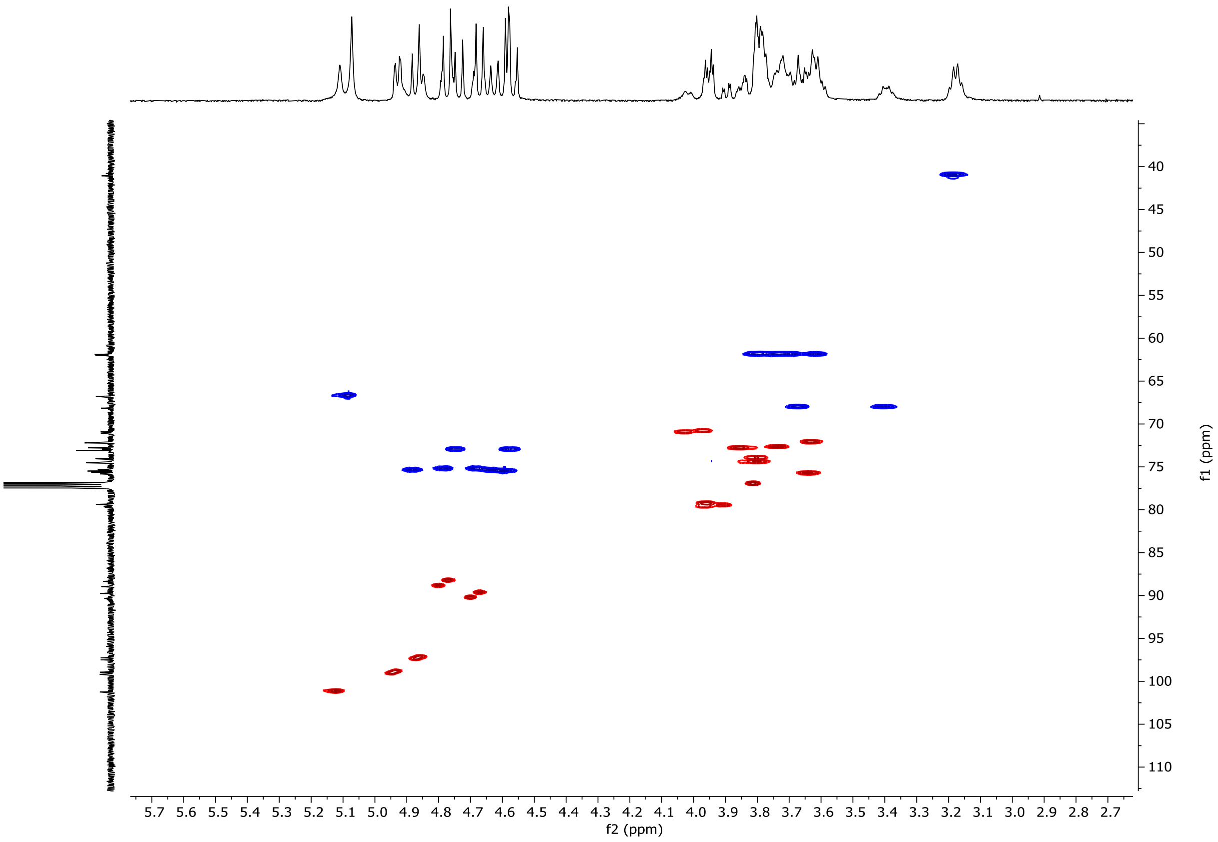

^{1}H NMR, 599 MHz, $CDCl_3$ – Crude Compound S15

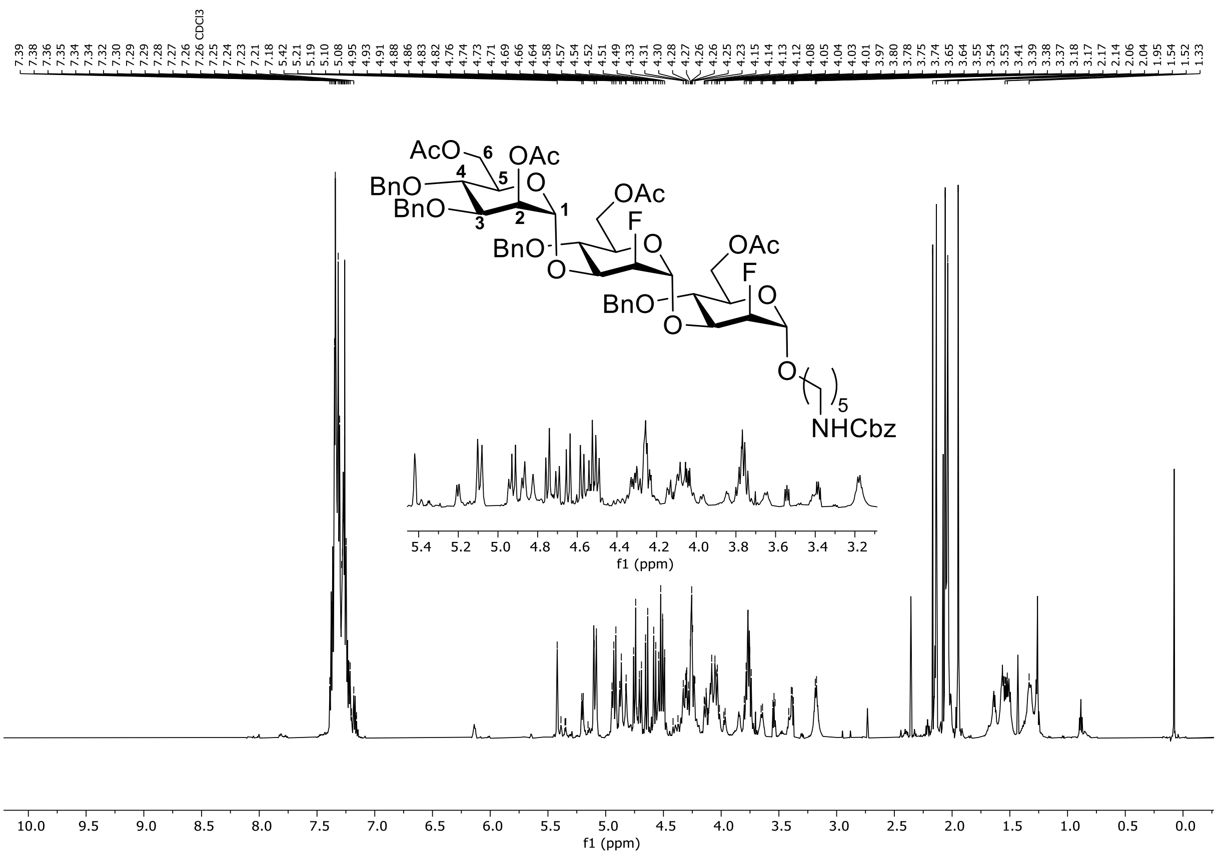

^{13}C NMR, 151 MHz, $CDCl_3$ – Crude Compound S15

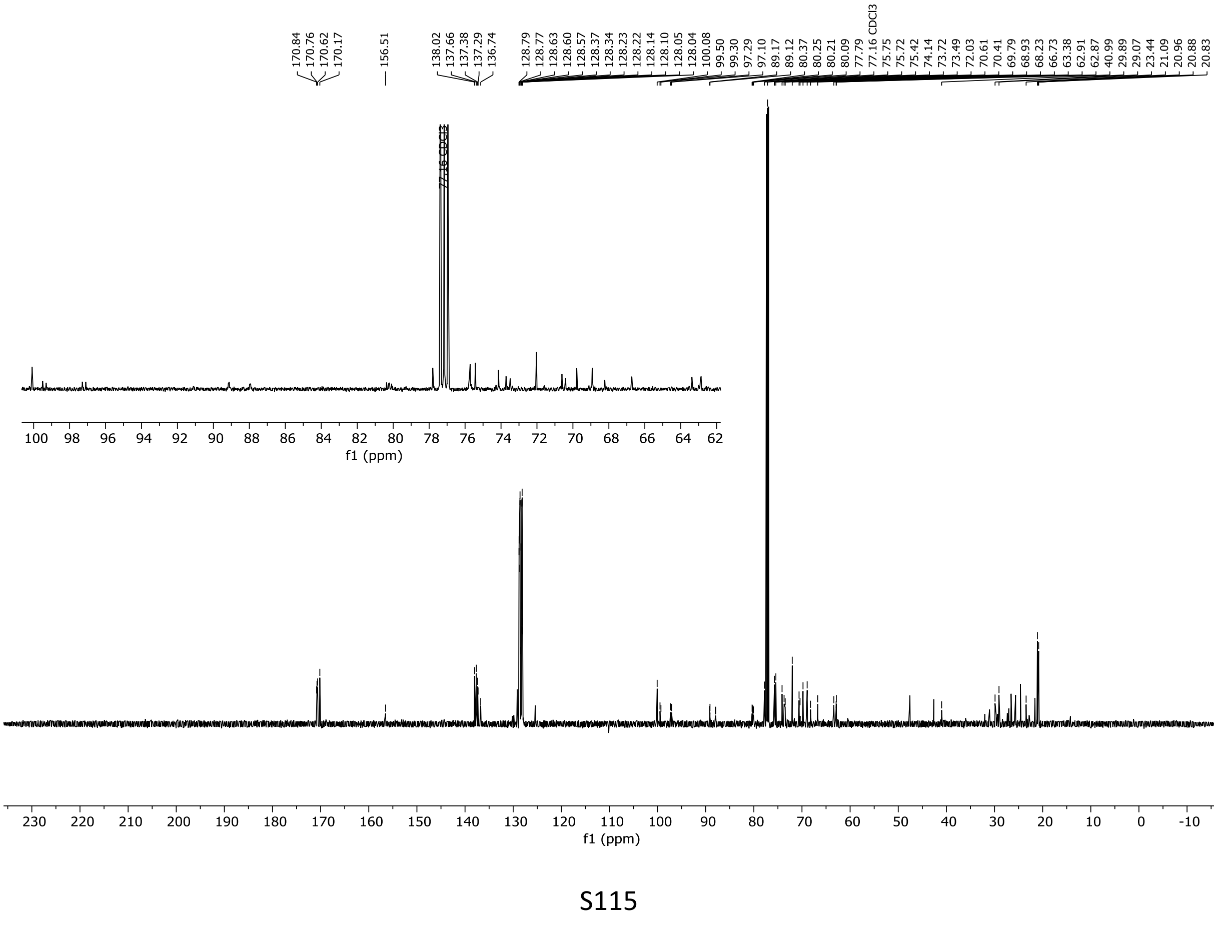

^{19}F NMR, 564 MHz, $CDCl_3$ – Crude Compound S15

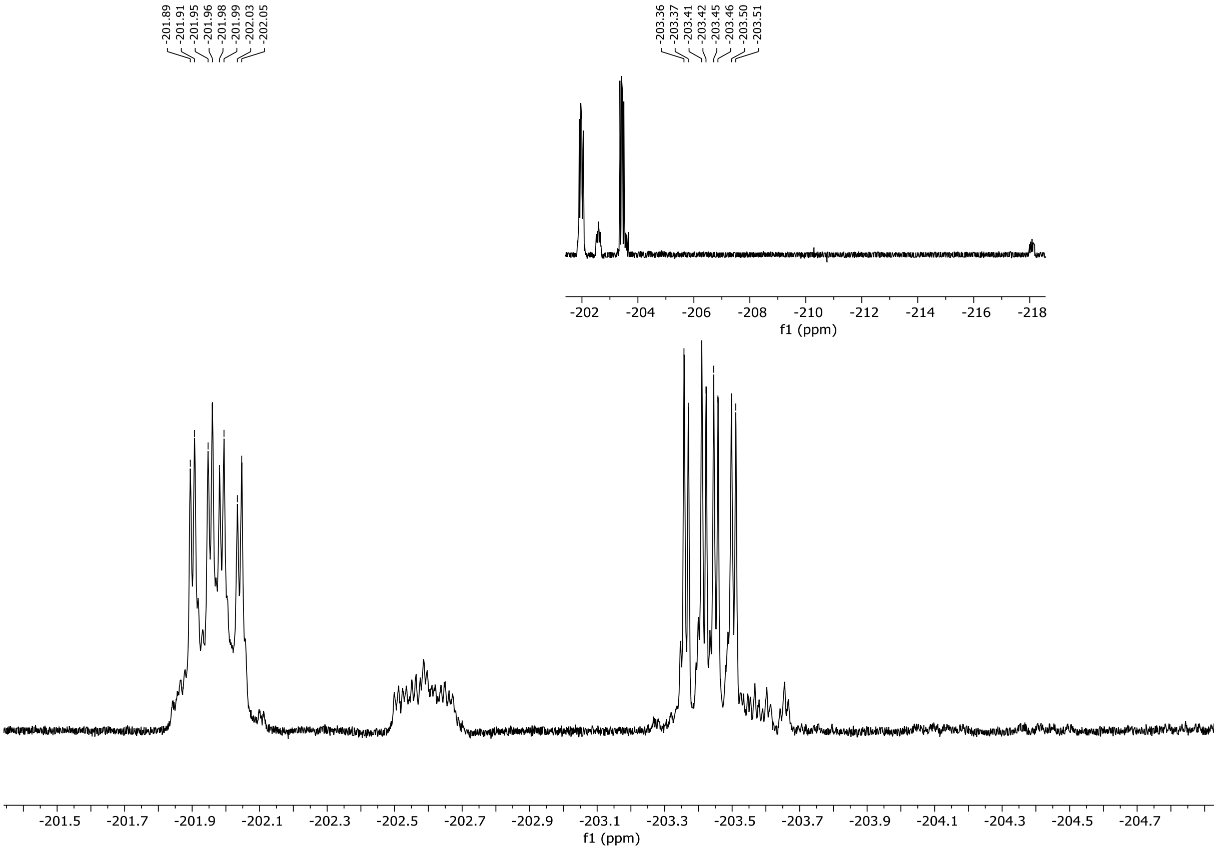

^{1}H-^{13}C gHSQC NMR, $CDCl_3$ – Carbohydrate Region, Crude Compound S15

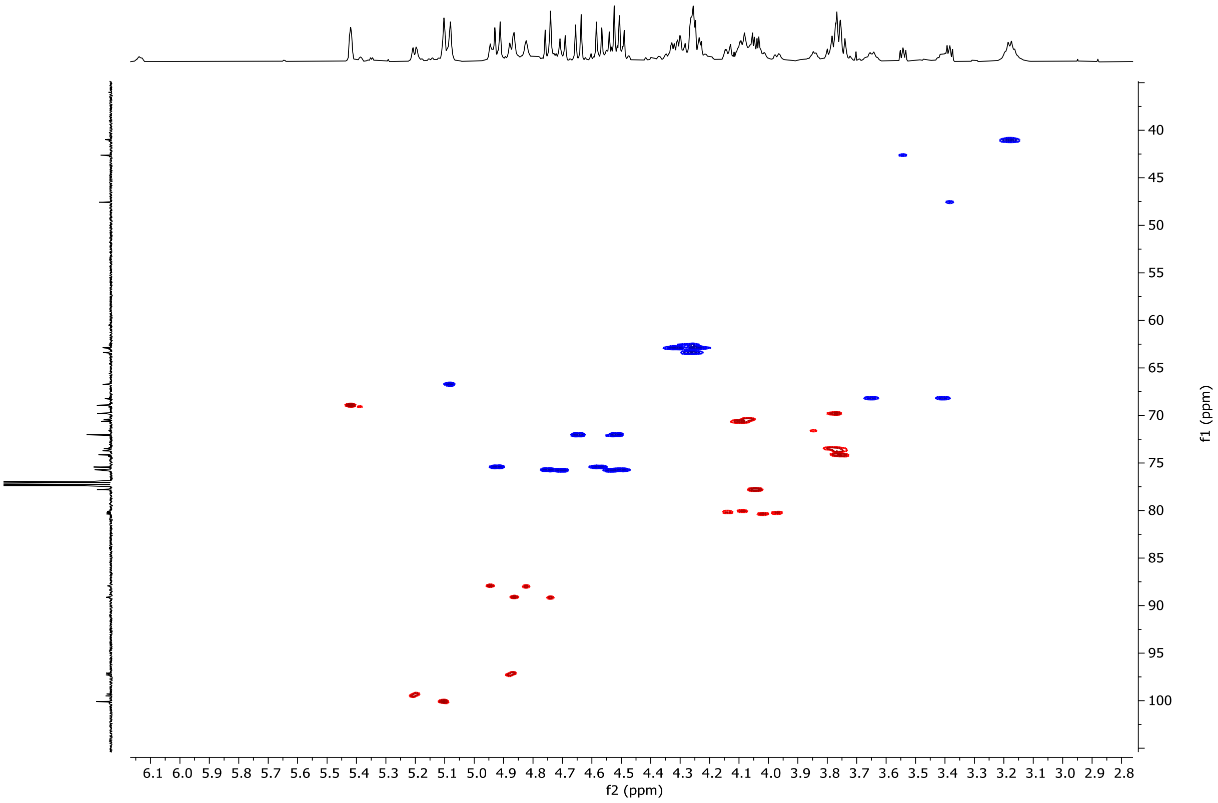

^{1}H NMR, 599 MHz, $CDCl_3$ – Compound 23

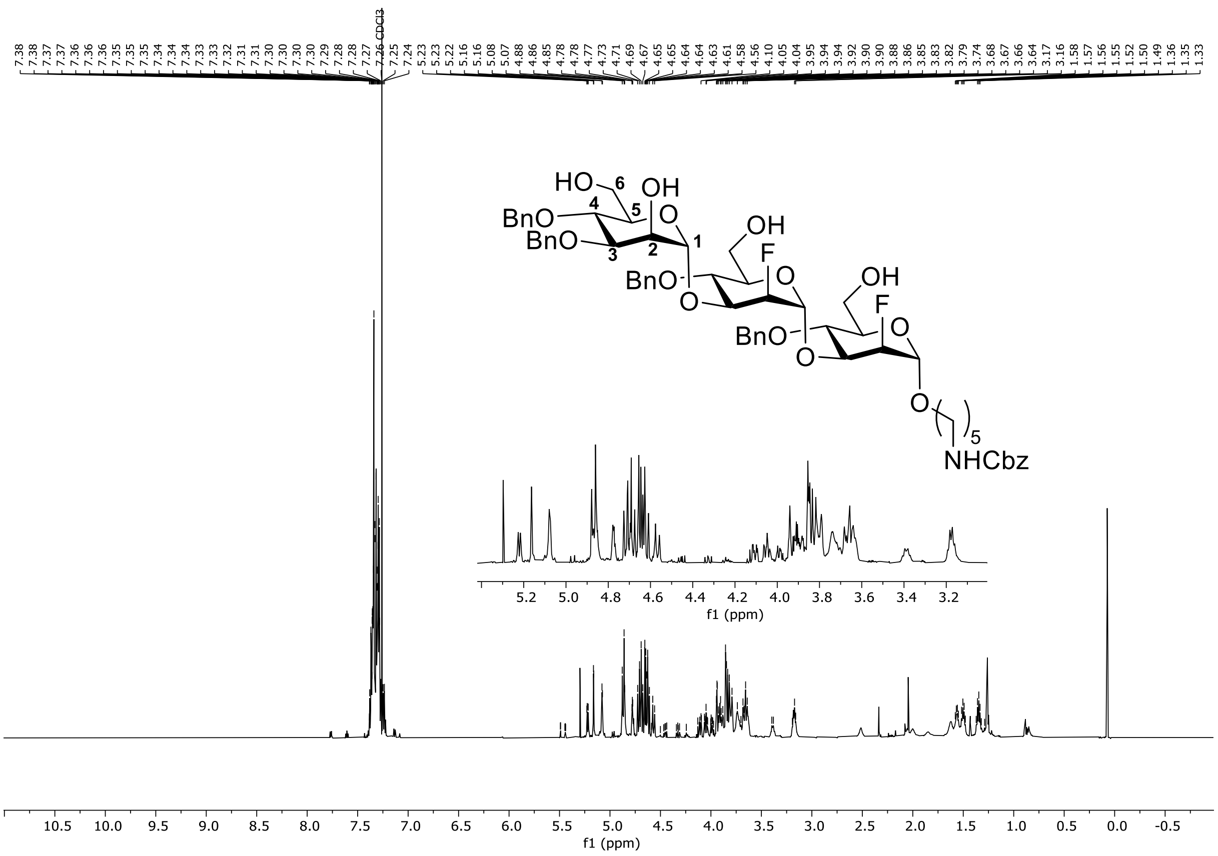

^{13}C NMR, 151 MHz, $CDCl_3$ – Compound 23

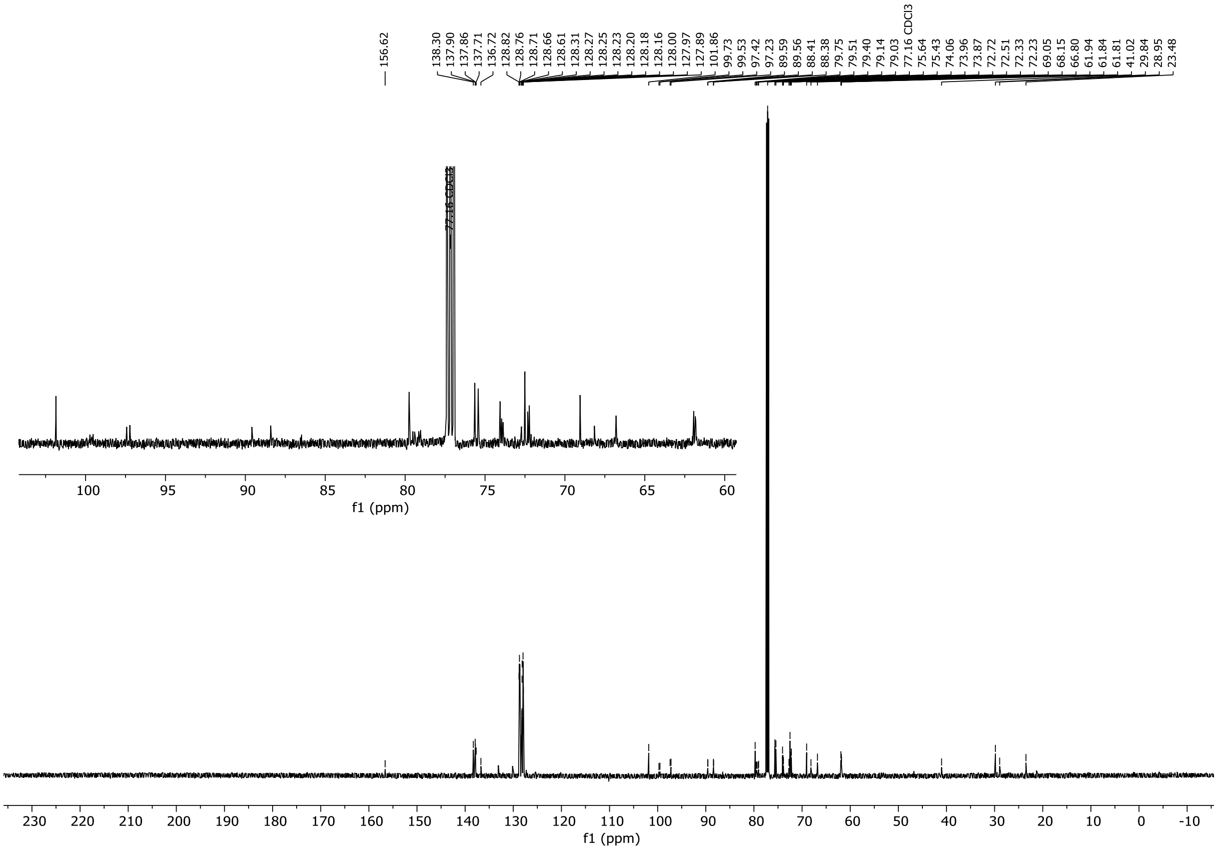

^{19}F NMR, 564 MHz, $CDCl_3$ – Compound 23

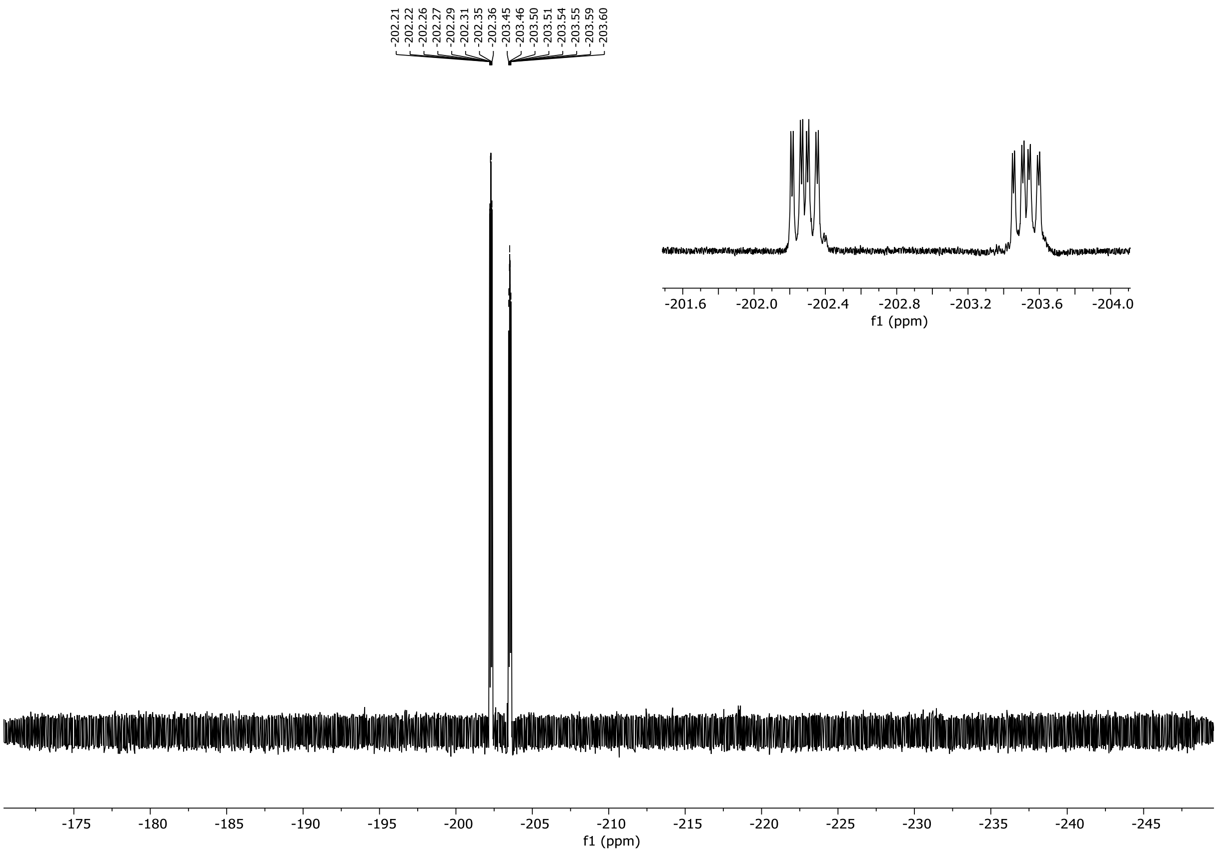

^{1}H-^{13}C gHSQC NMR, $CDCl_3$ – Carbohydrate Region, Compound 23

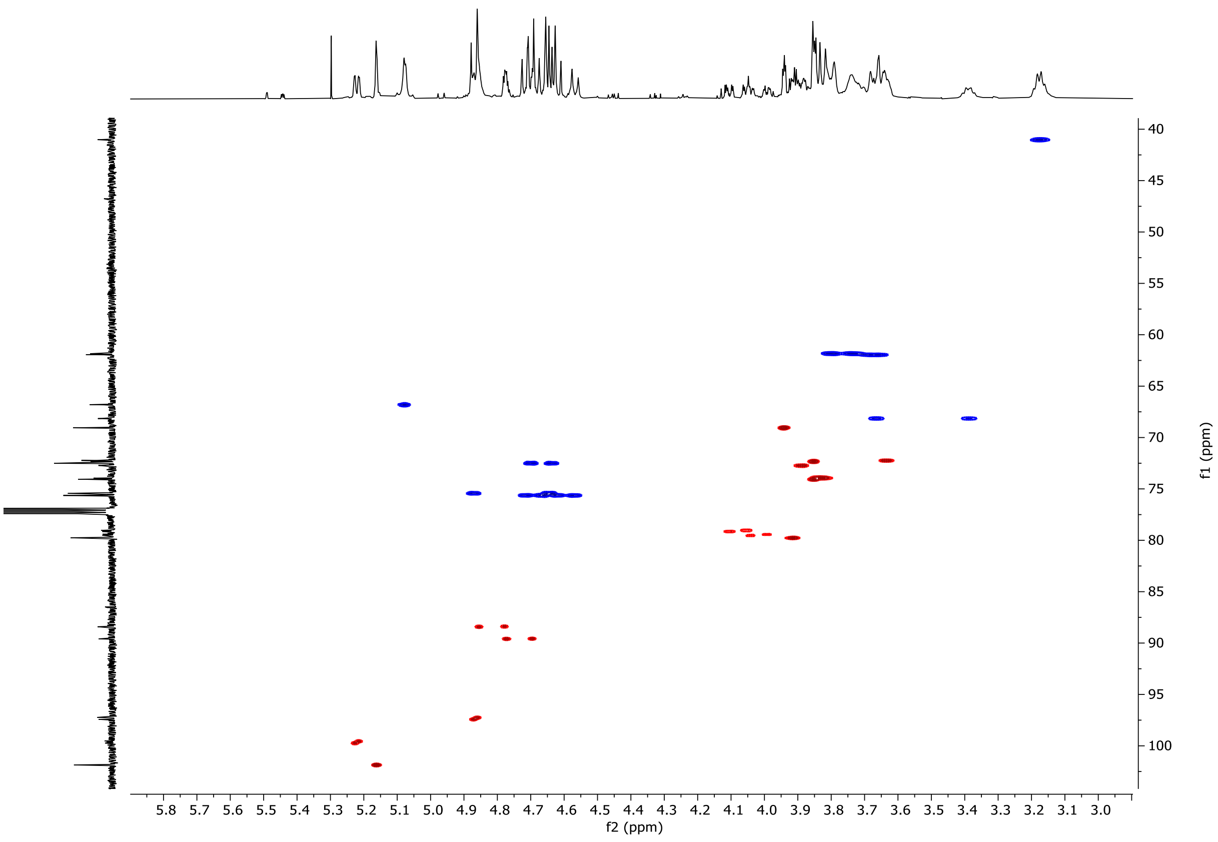

^{1}H NMR, 599 MHz, $CDCl_3$ – Compound S16

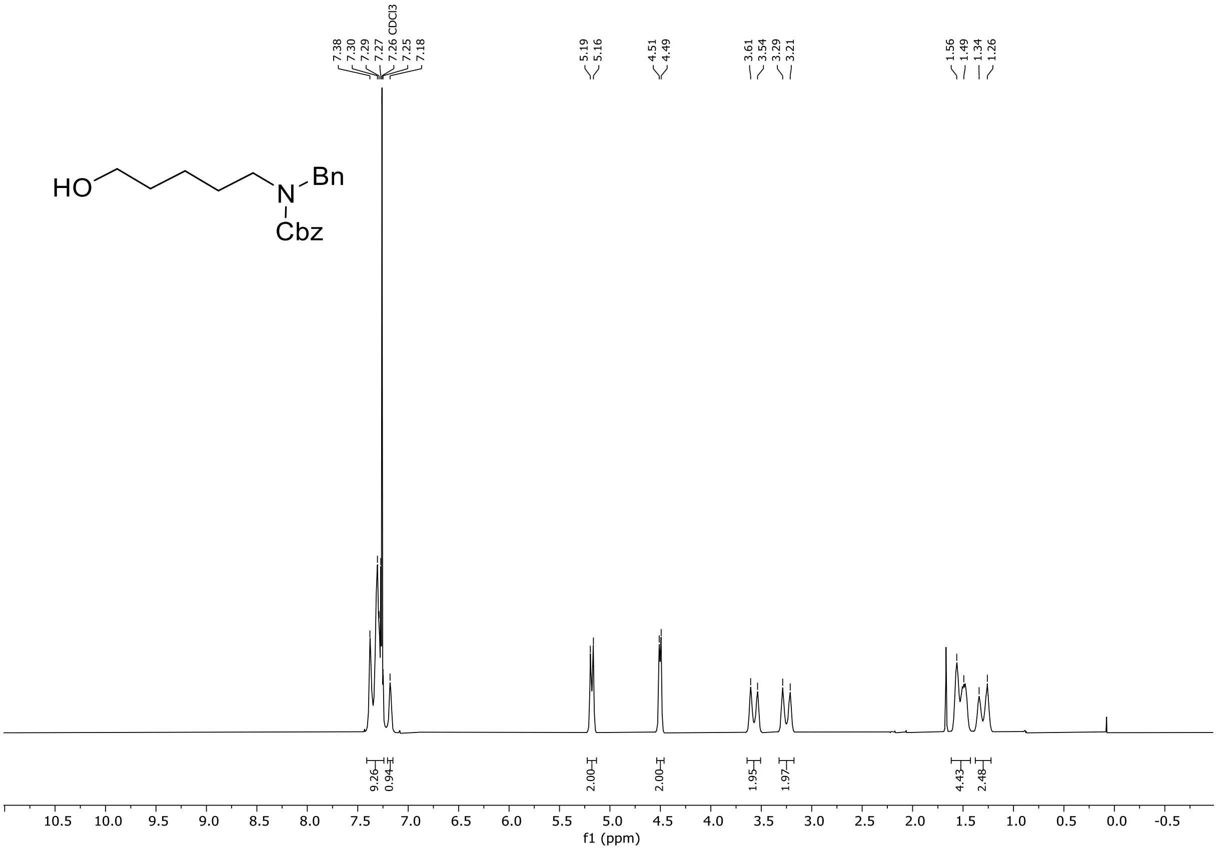

^{13}C NMR, 151 MHz, $CDCl_3$ – Compound S16

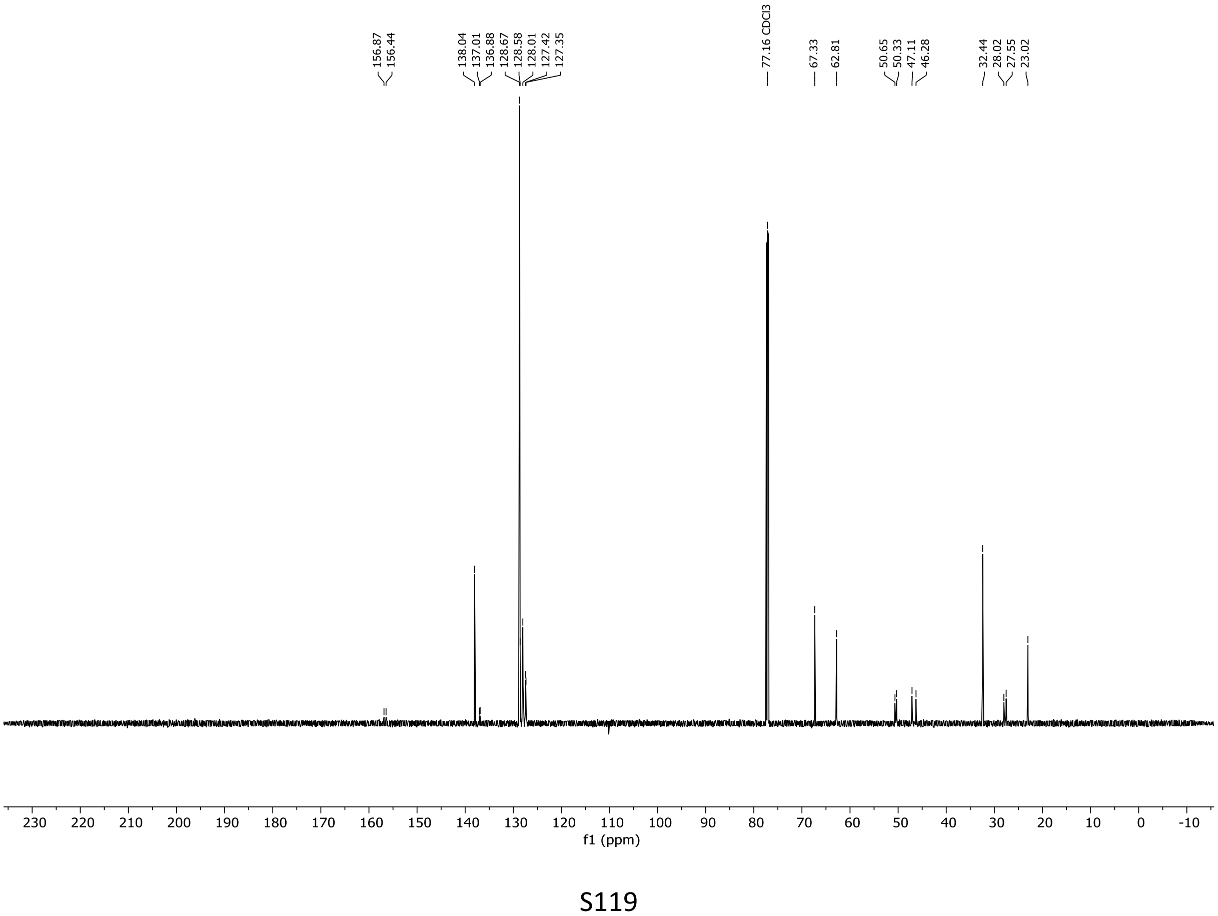

^{1}H NMR, 599 MHz, $CDCl_3$ – Compound S17

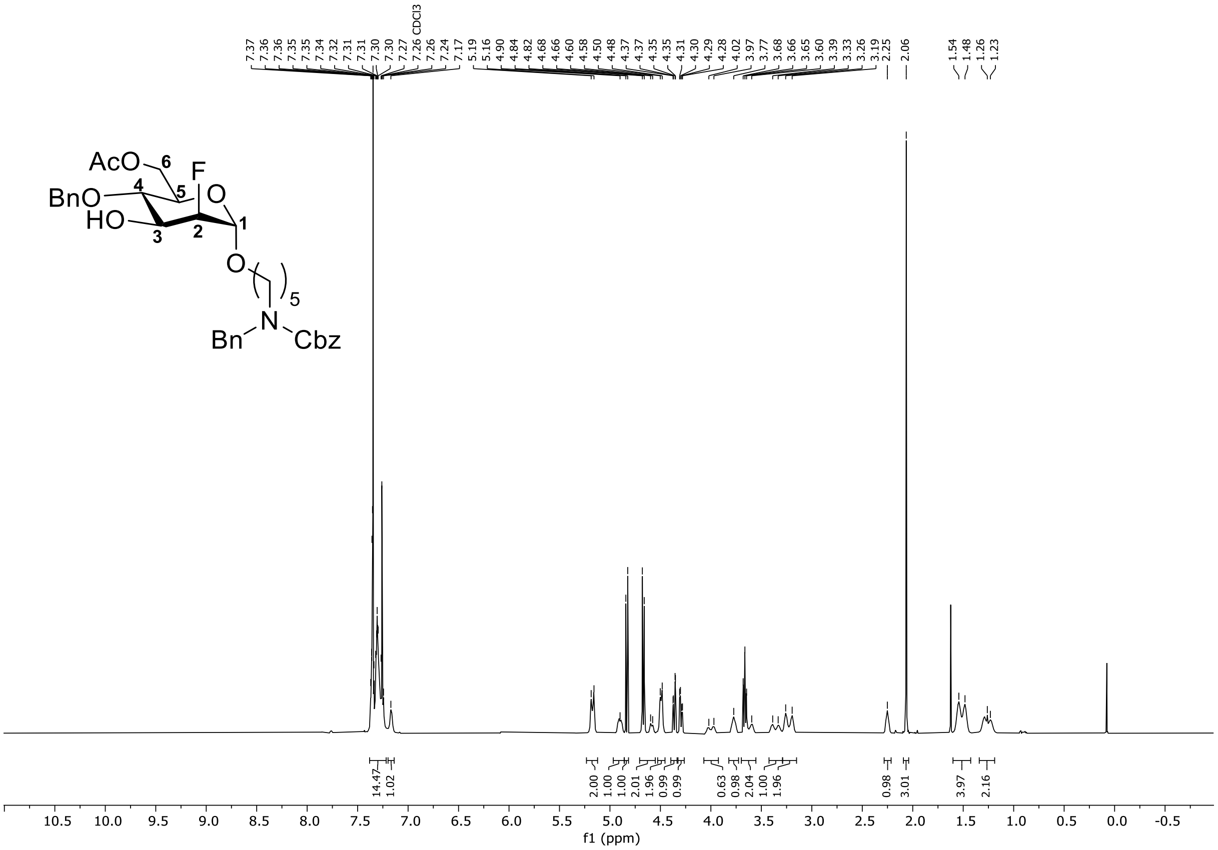

^{13}C NMR, 151 MHz, $CDCl_3$ – Compound S17

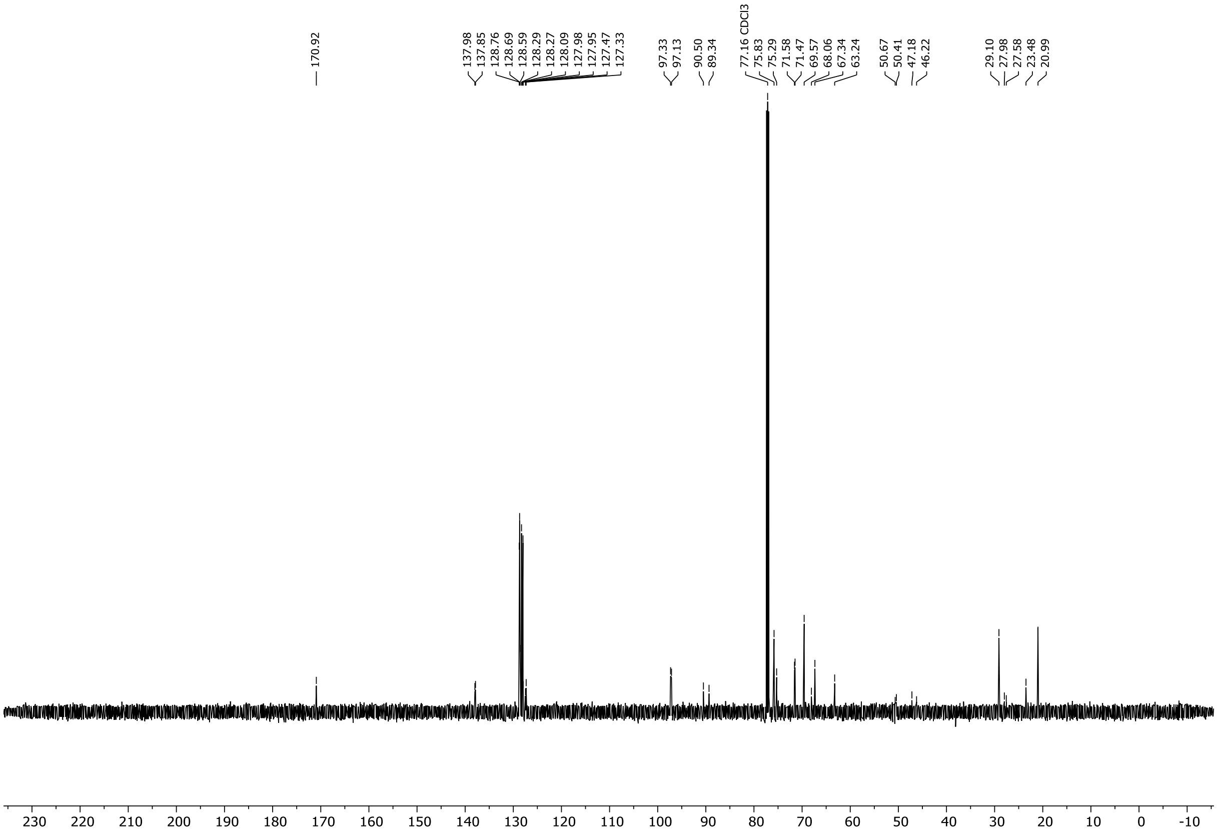

^{19}F NMR, 564 MHz, CDCl$_3$ – Compound S17

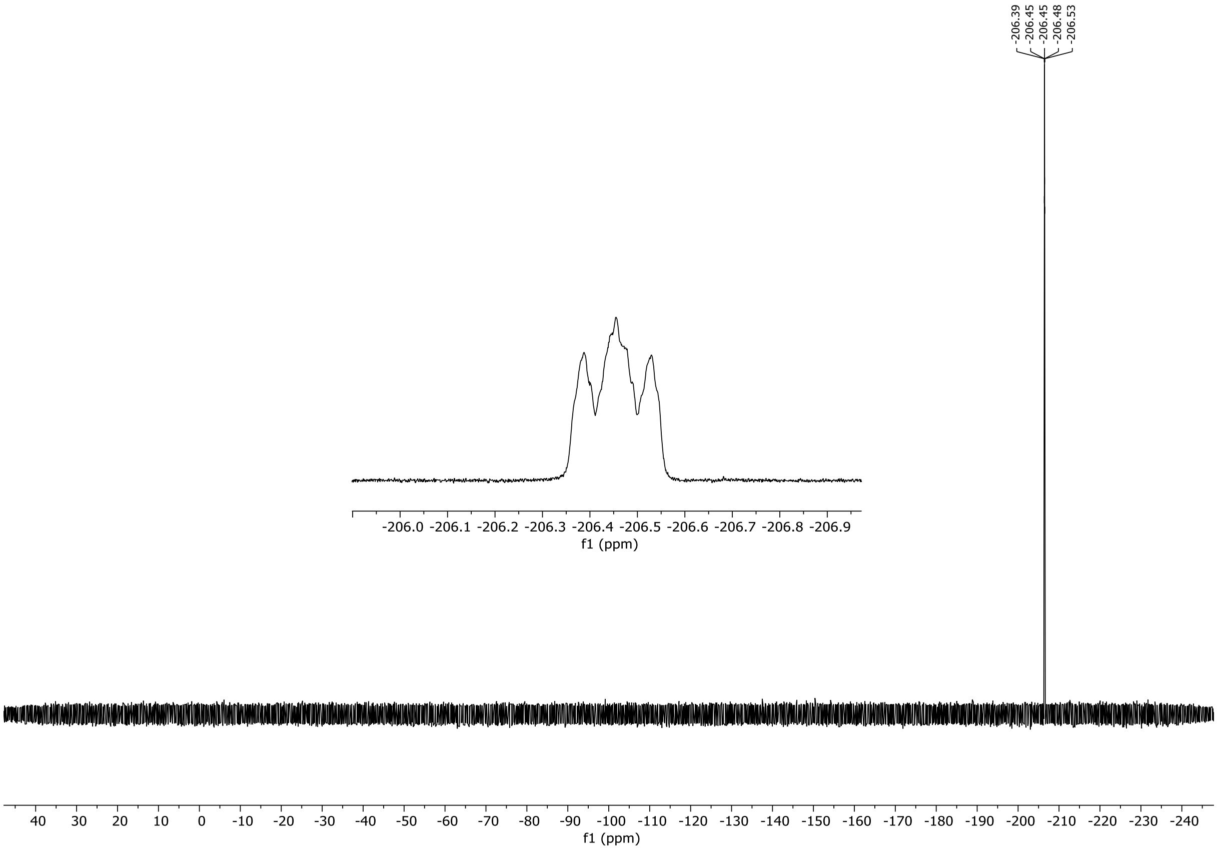

Variable Temperature ^{19}F NMR, 470 MHz, CDCl$_3$ – Compound S17

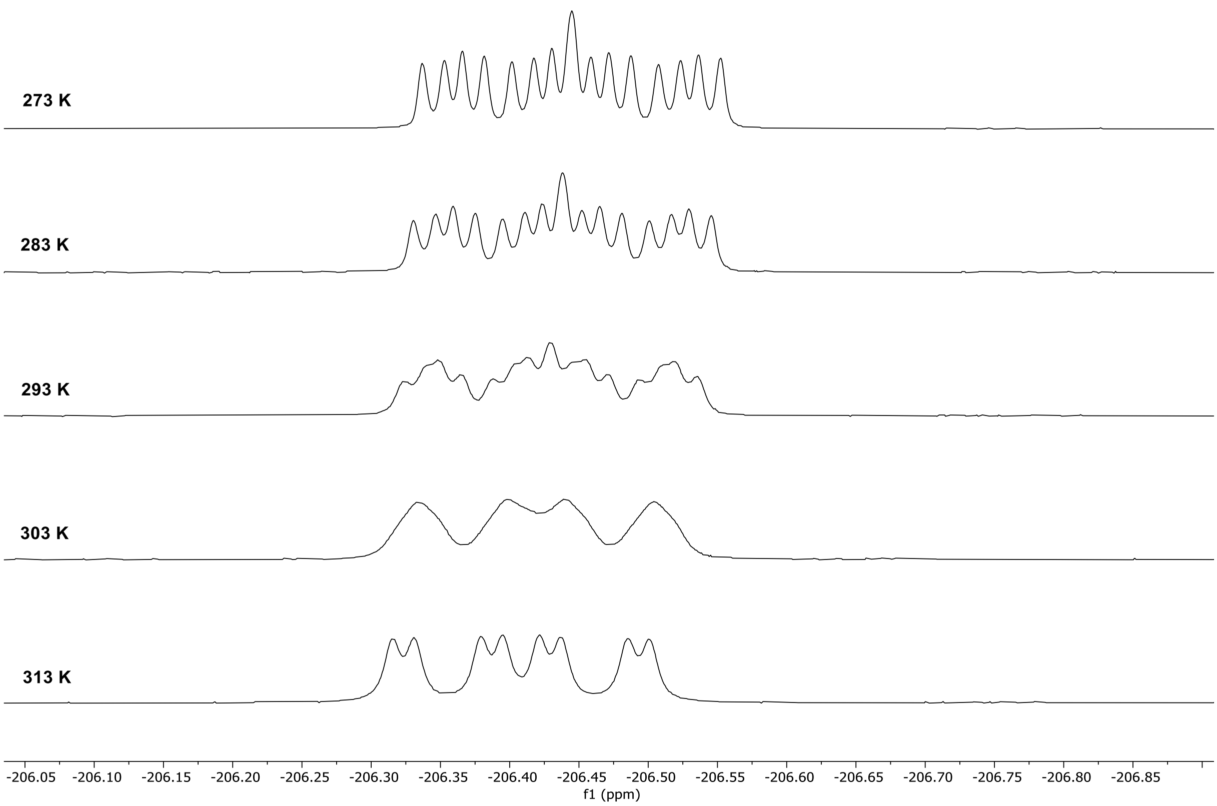

^{1}H-^{13}C gHSQC NMR, $CDCl_3$ – Carbohydrate Region, Compound S17

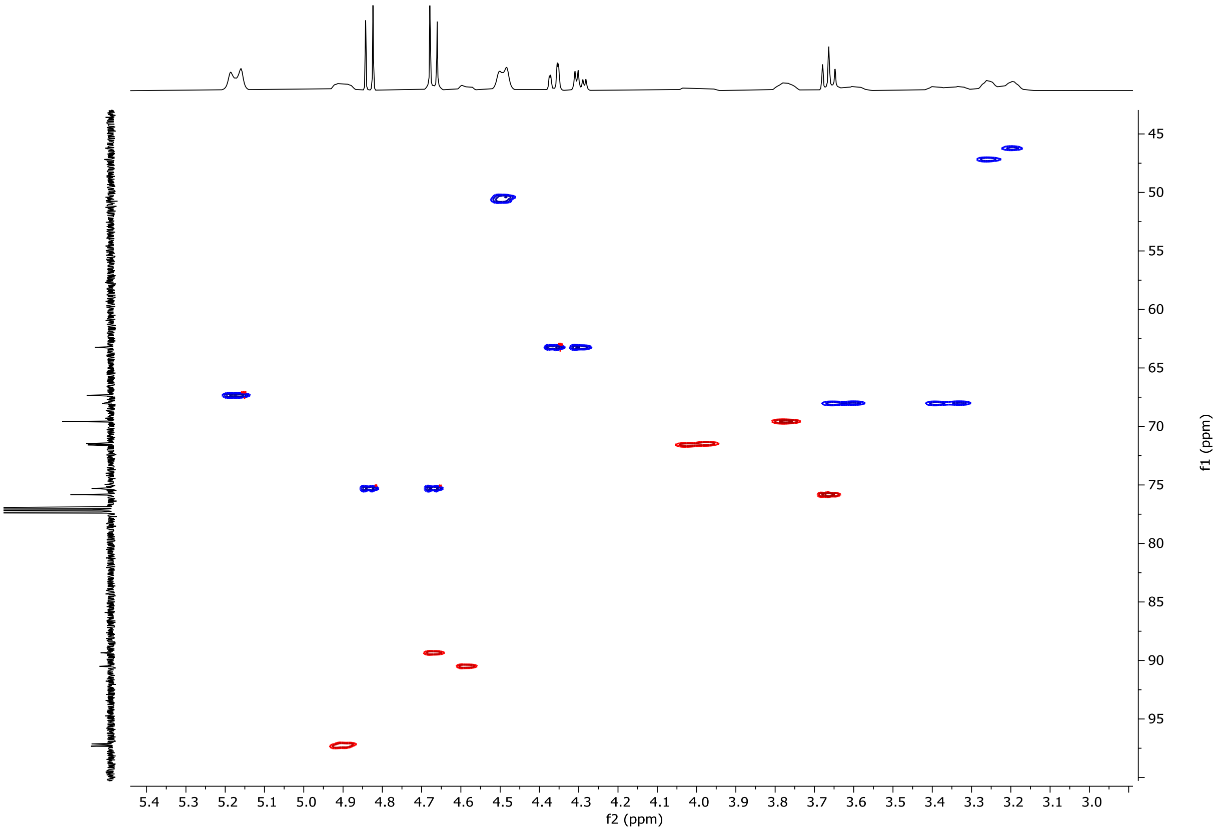

^{1}H NMR, 599 MHz, $CDCl_3$ – Compound S16a

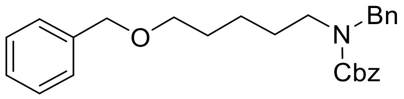

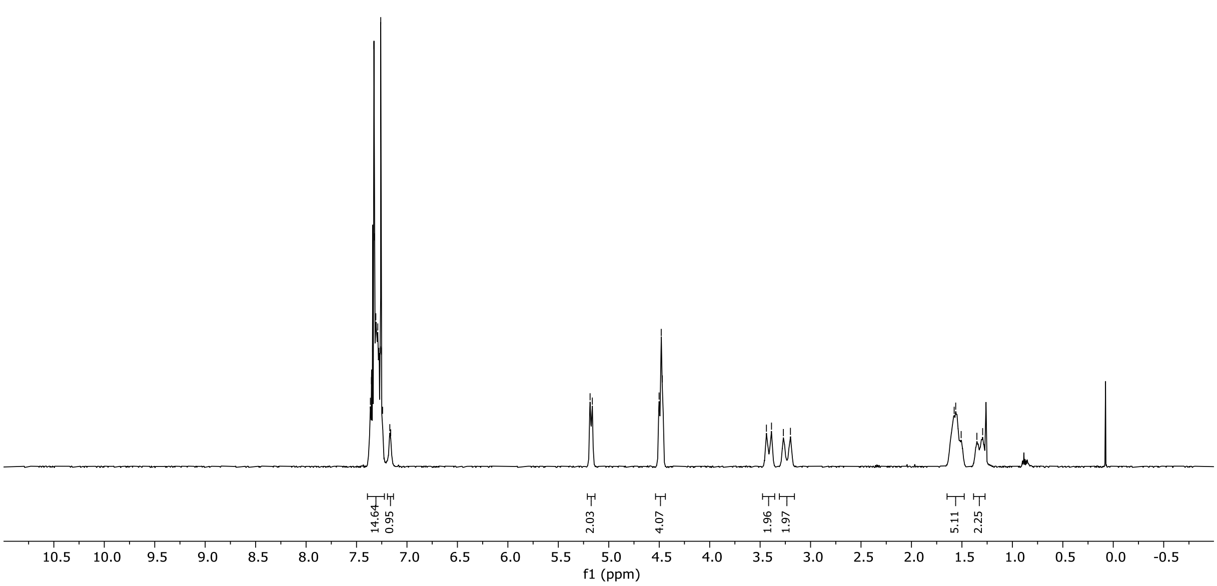

^{13}C NMR, 151 MHz, $CDCl_3$ – Compound S16a

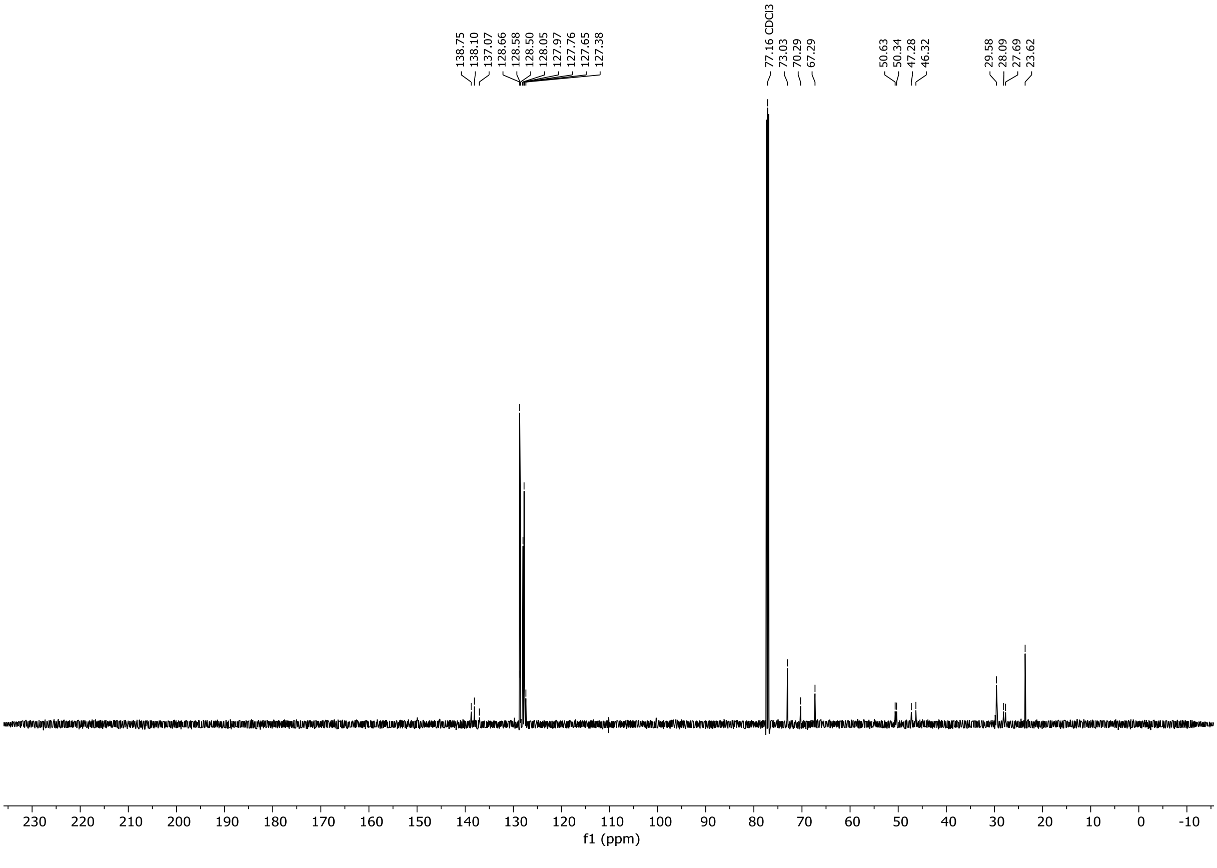

^{1}H NMR, 500 MHz, $CDCl_3$ – Compound S16b

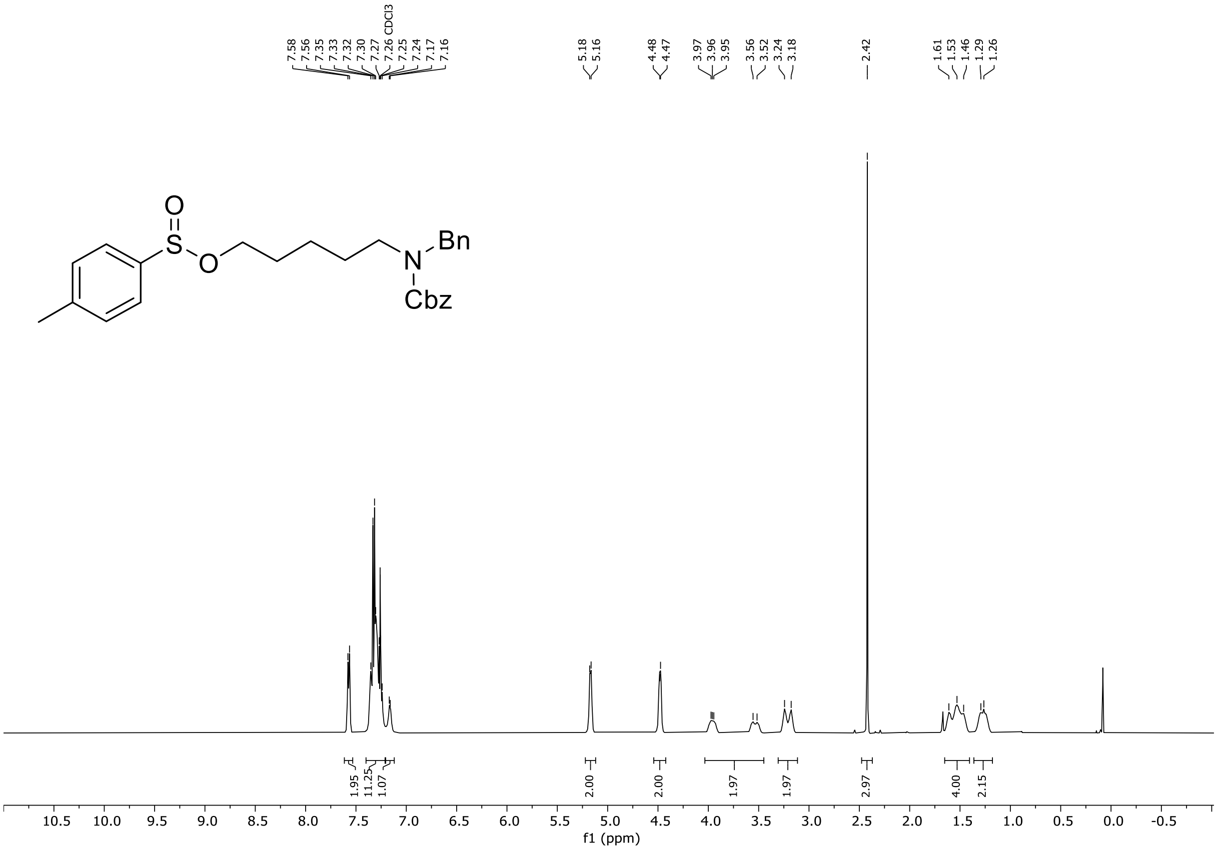

^{13}C NMR, 151 MHz, $CDCl_3$ – Compound S16b

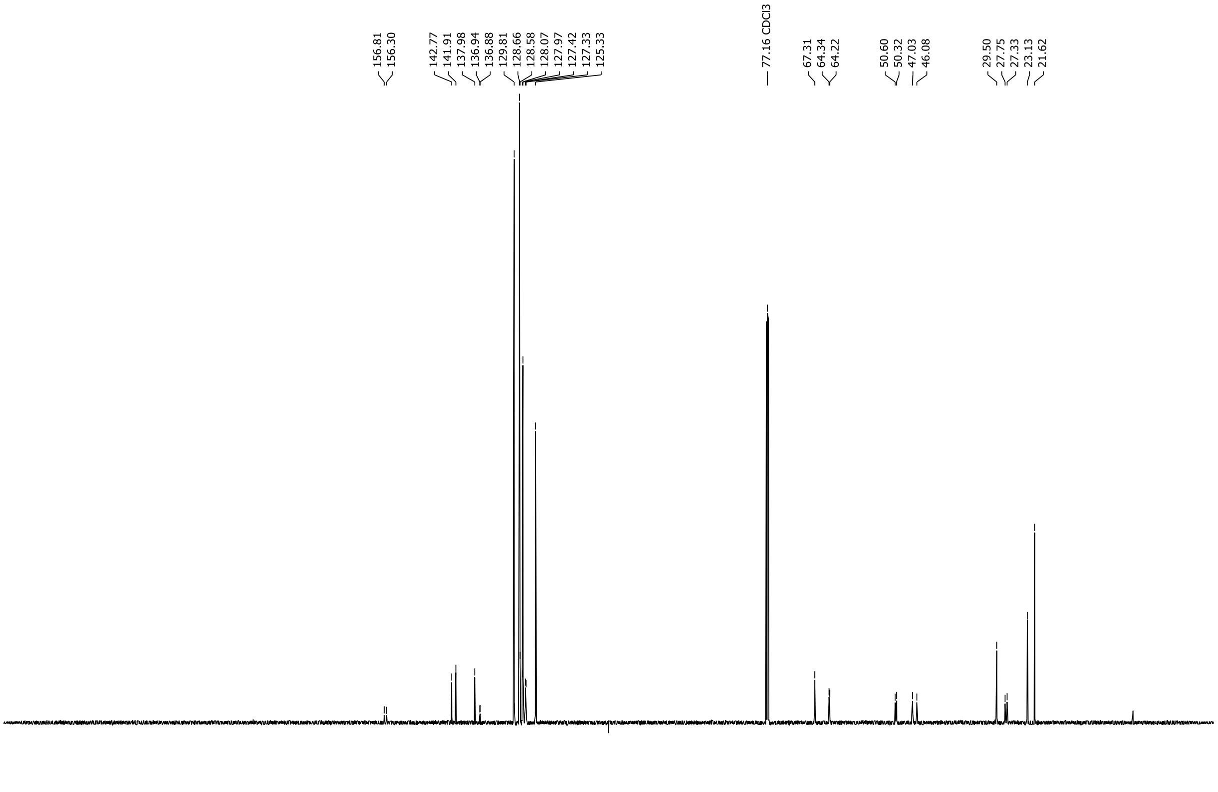

^{1}H NMR, 500 MHz, $CDCl_3$ – Compound S16c

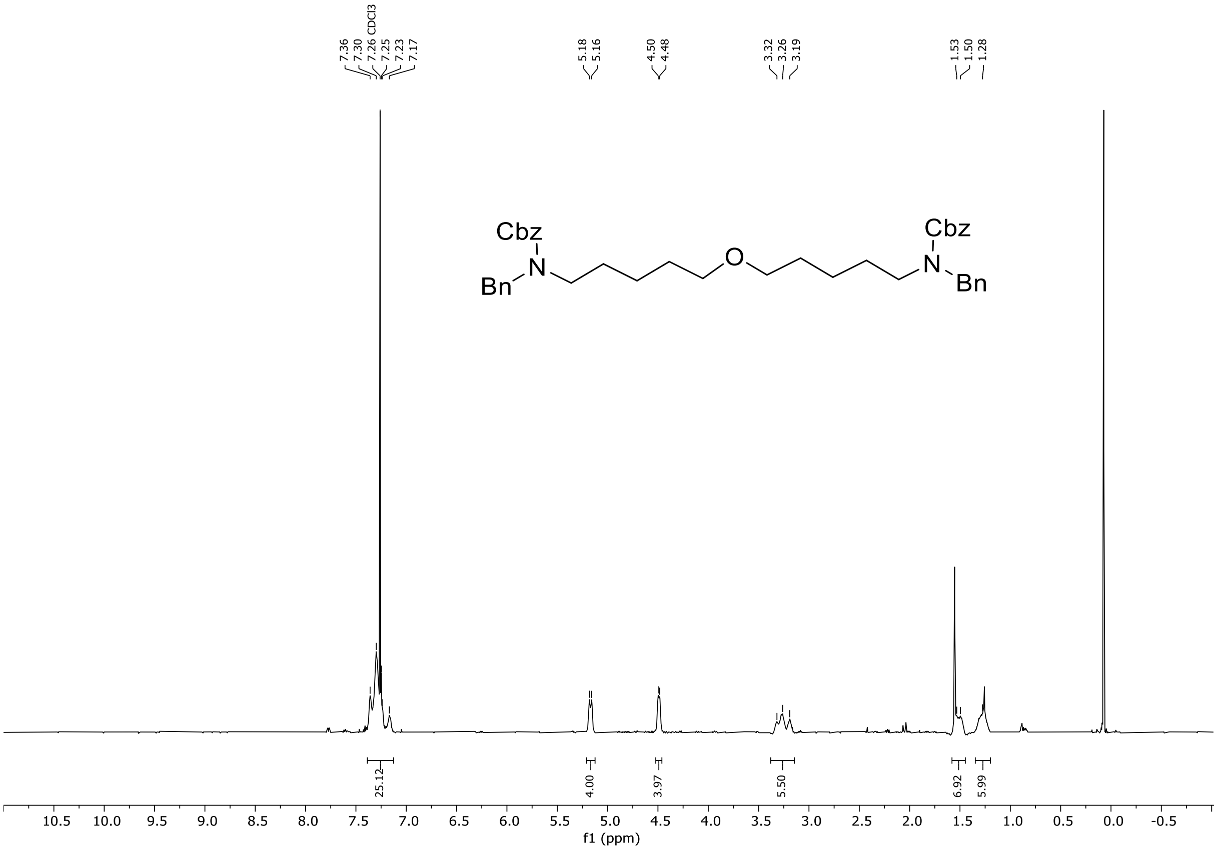

^{13}C NMR, 151 MHz, $CDCl_3$ – Compound S16c

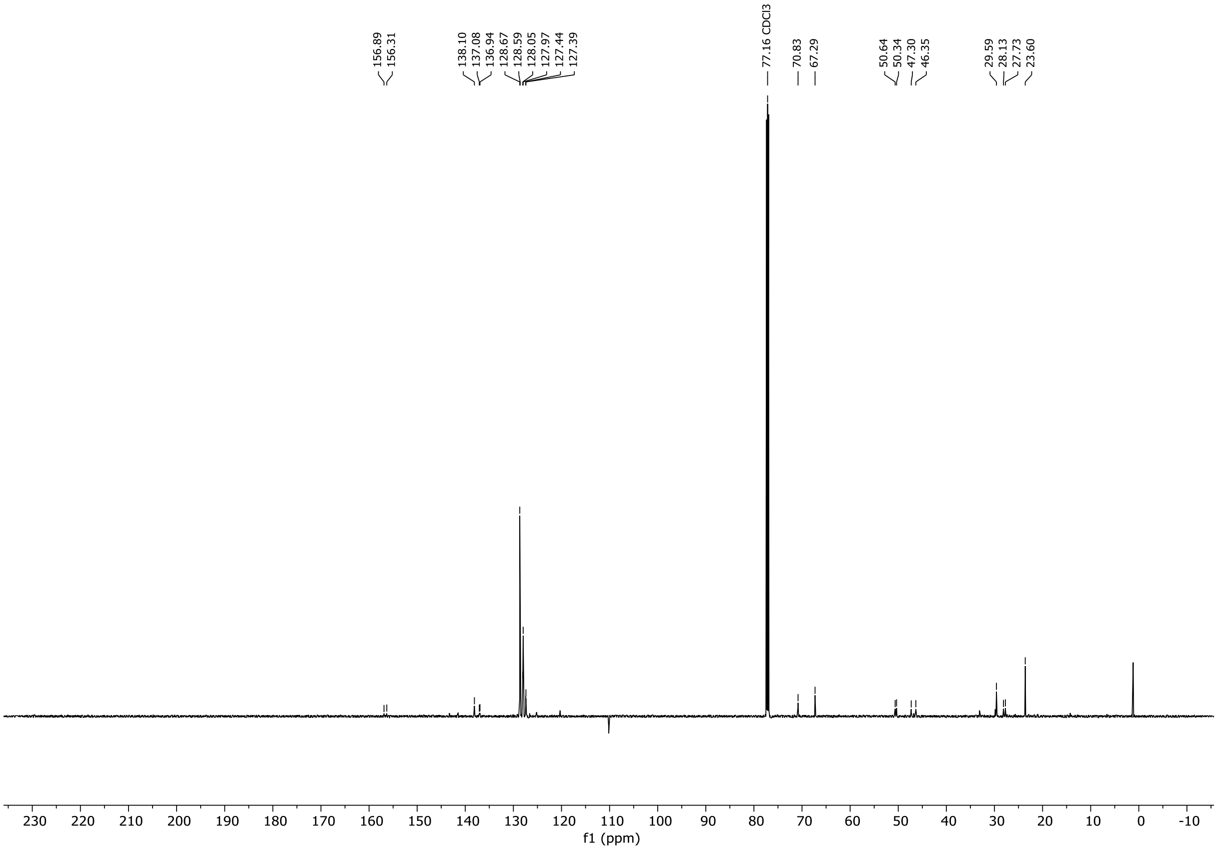

^{1}H NMR, 599 MHz, $CDCl_3$ – Compound S18

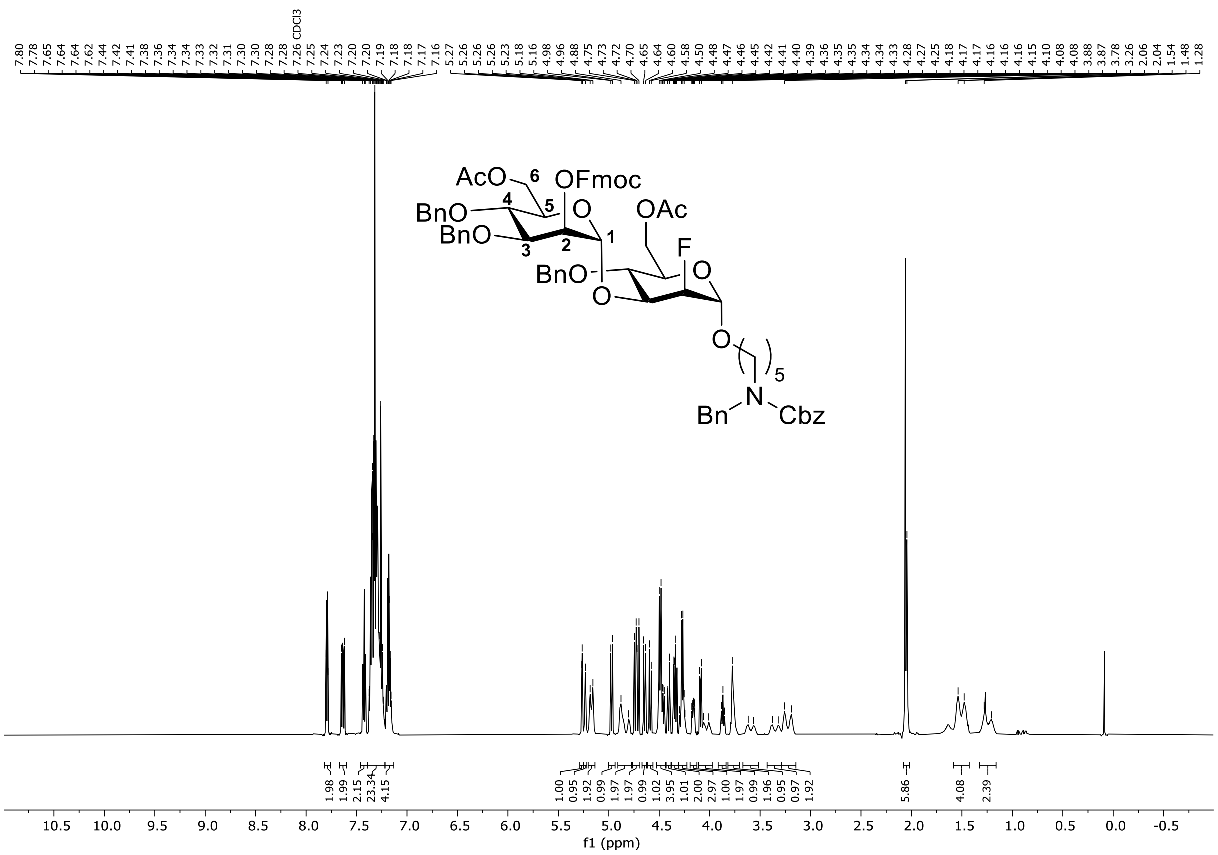

^{13}C NMR, 151 MHz, $CDCl_3$ – Compound S18

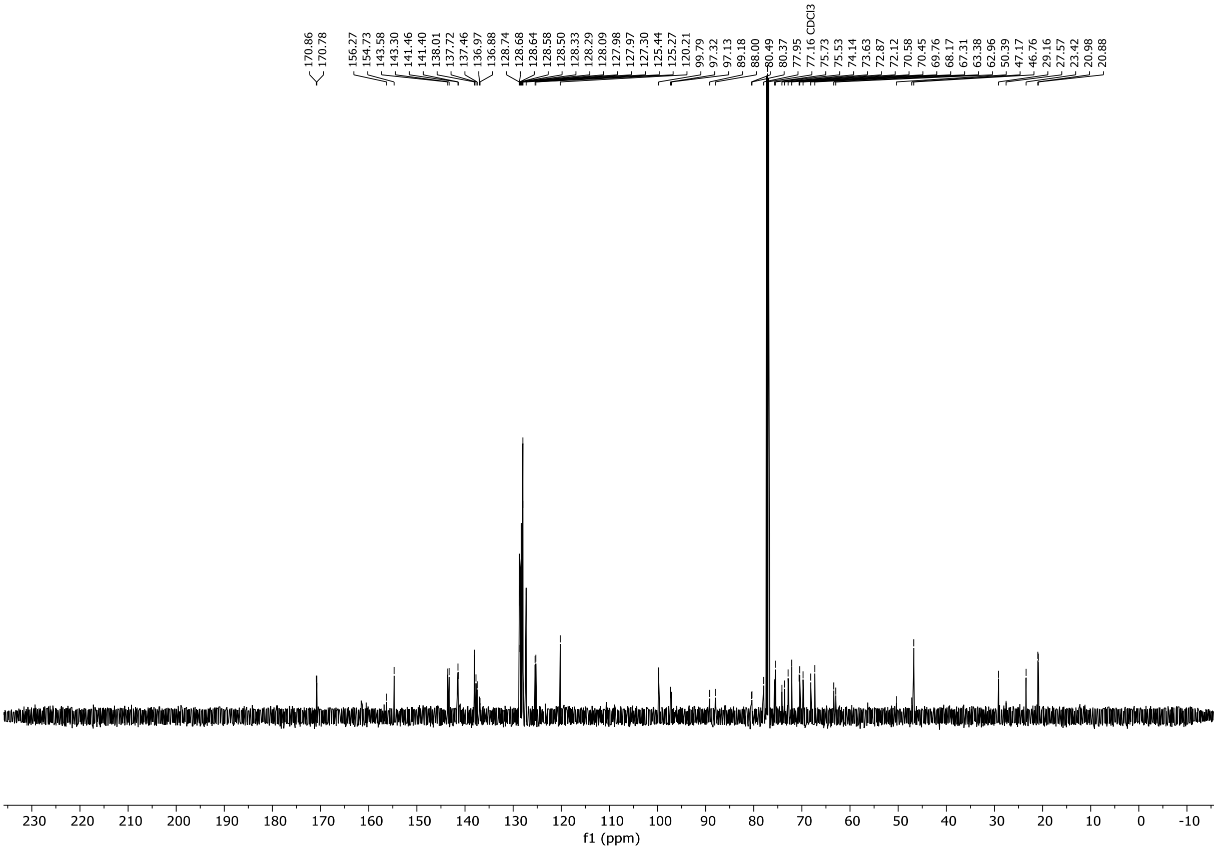

^{19}F NMR, 564 MHz, $CDCl_3$ – Compound S18

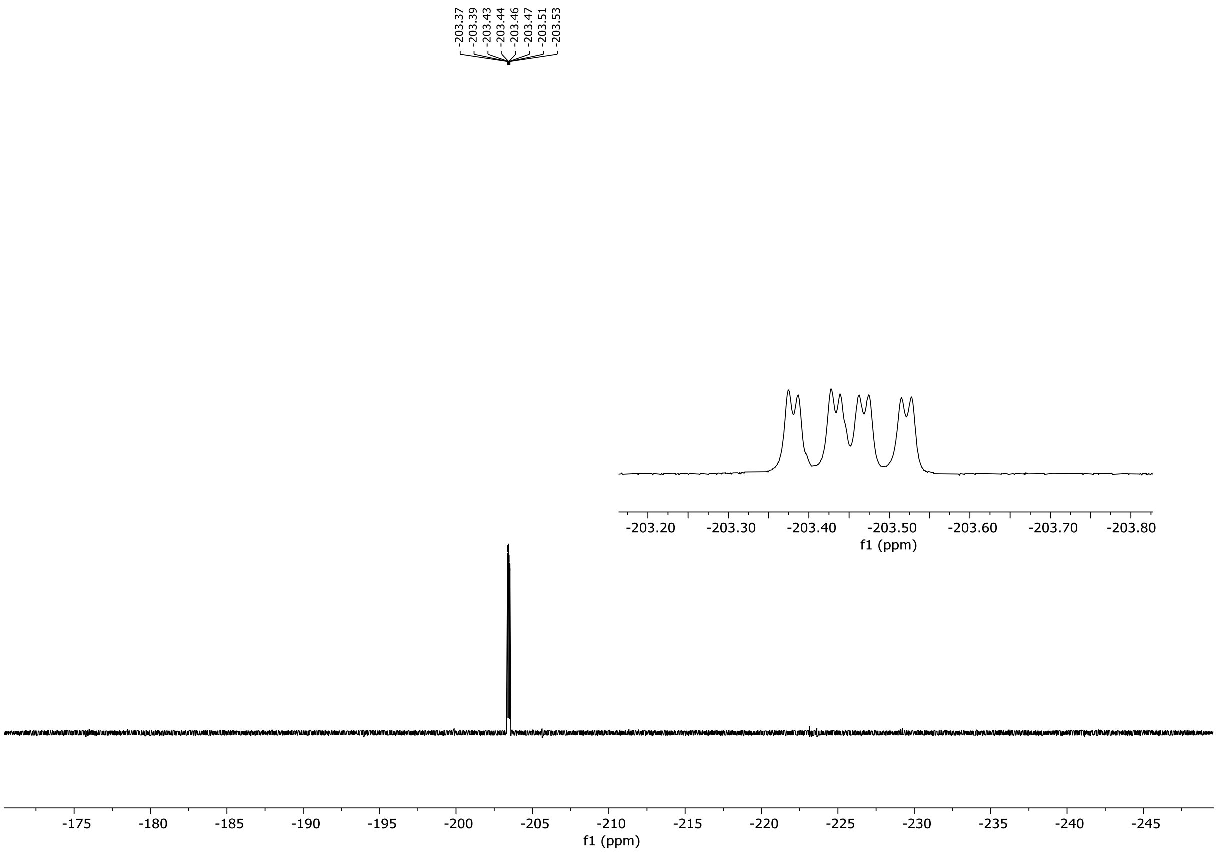

^{1}H-^{13}C gHSQC NMR, $CDCl_3$ – Carbohydrate Region, Compound S18

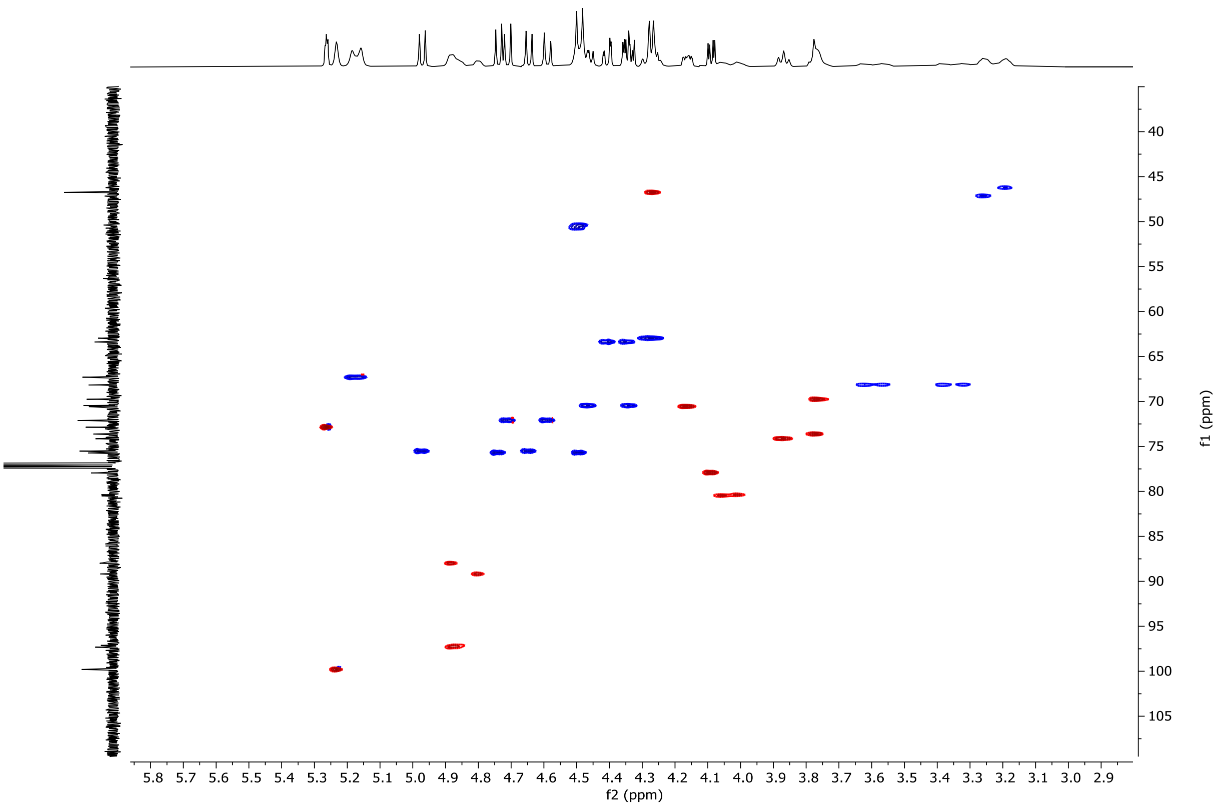

^{1}H NMR, 599 MHz, $CDCl_3$ – Compound S19

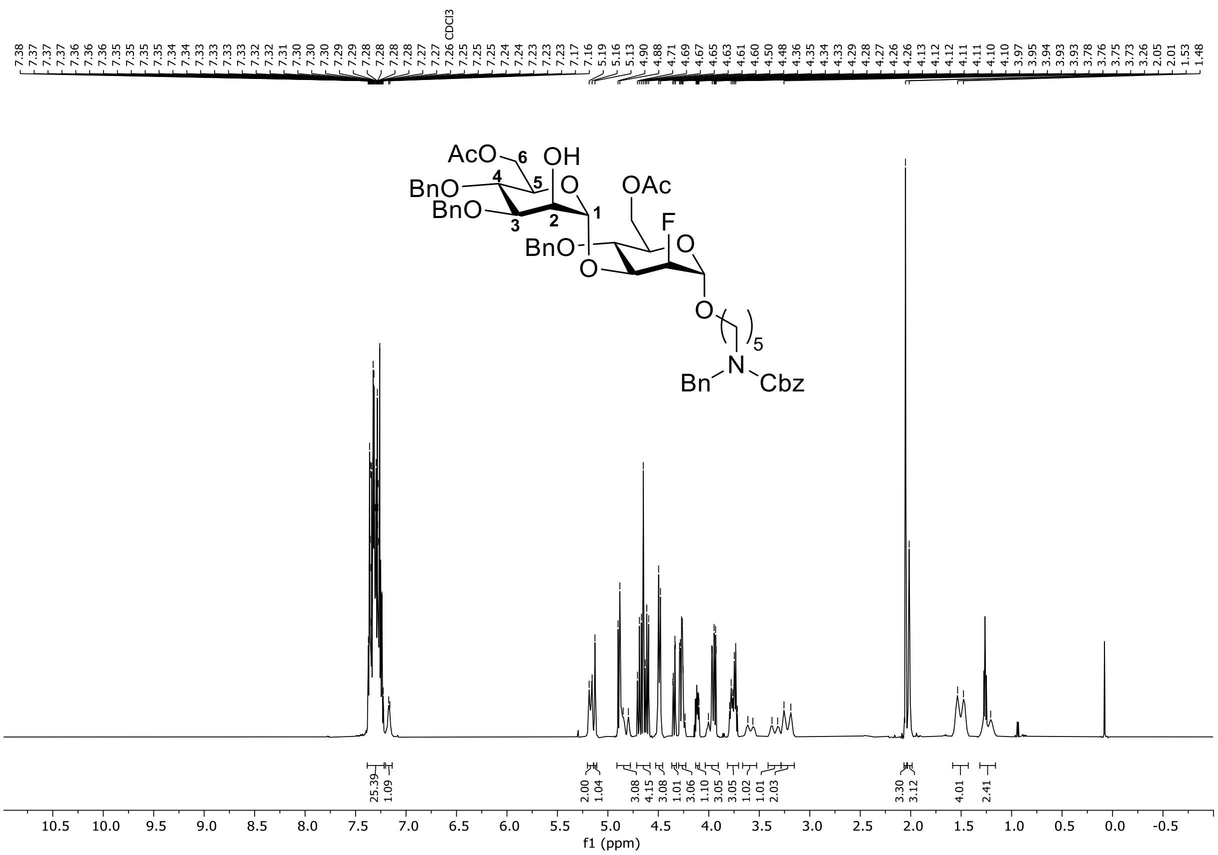

^{13}C NMR, 151 MHz, $CDCl_3$ – Compound S19

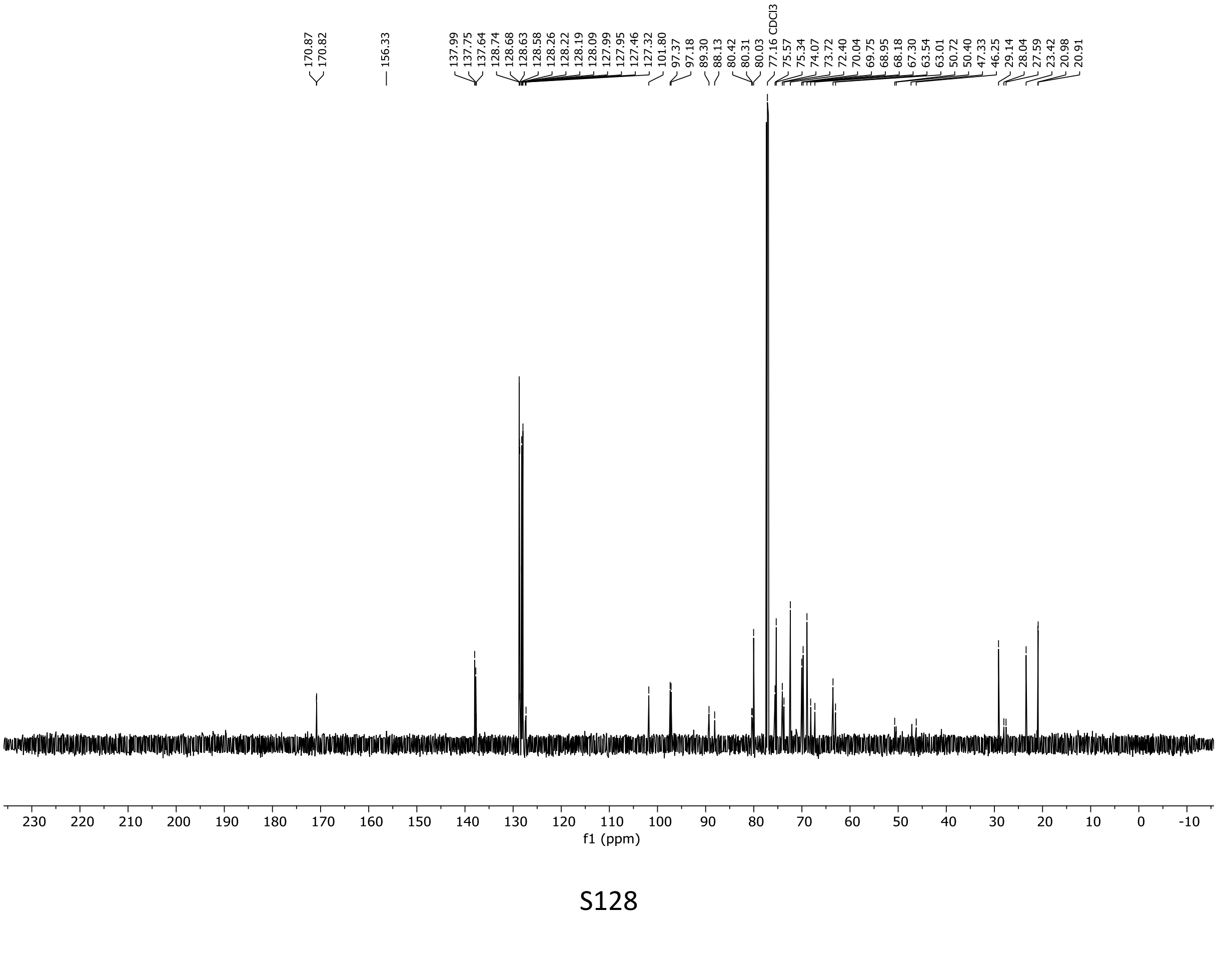

^{19}F NMR, 564 MHz, CDCl$_3$ – Compound S19

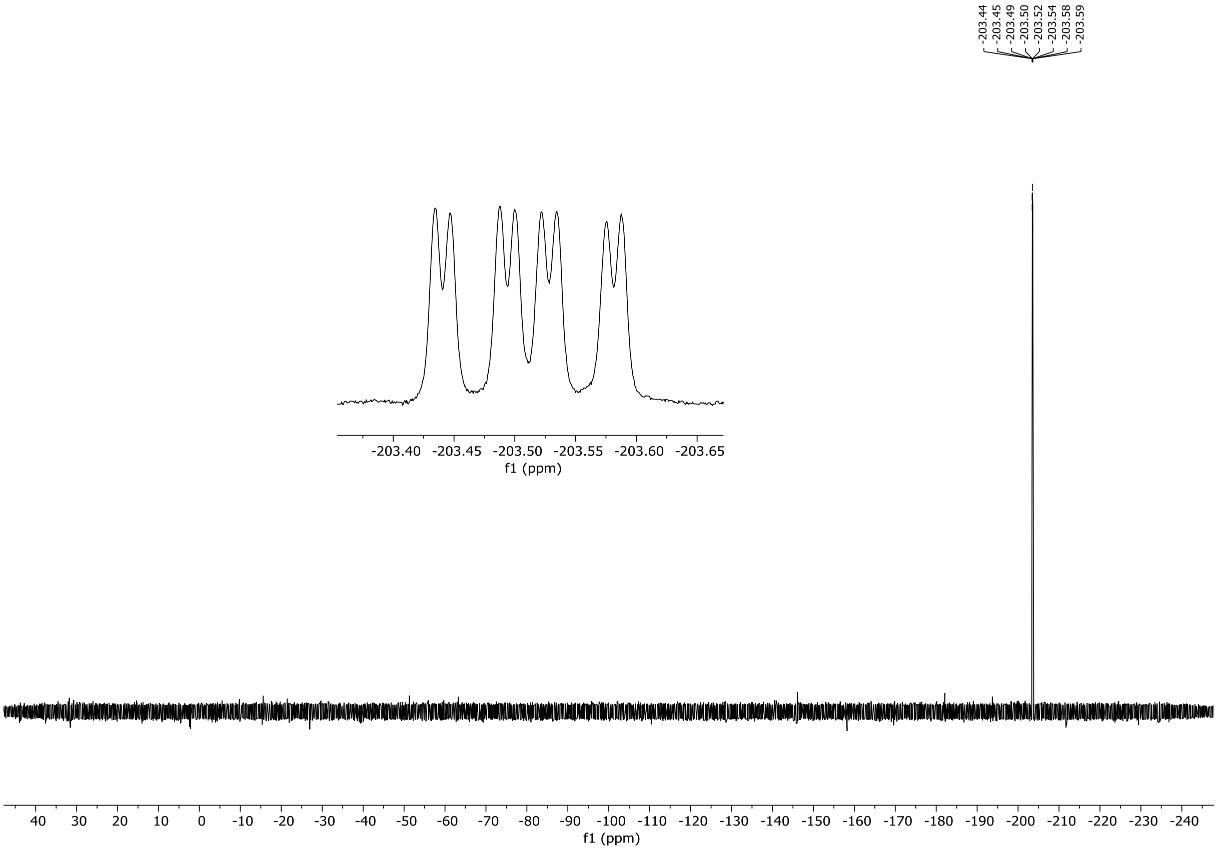

^{1}H-^{13}C gHSQC NMR, CDCl$_3$ – Carbohydrate Region, Compound S19

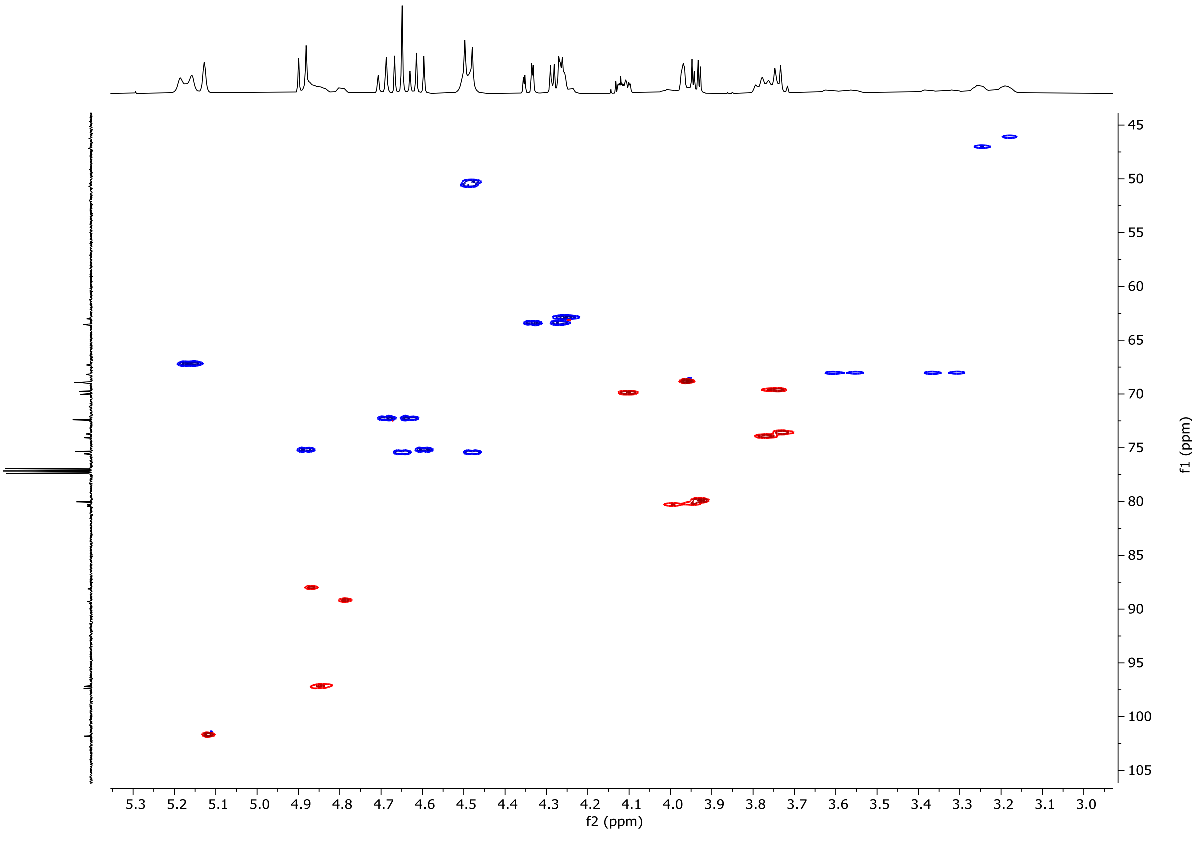

^{1}H NMR, 599 MHz, $CDCl_3$ – Compound S20

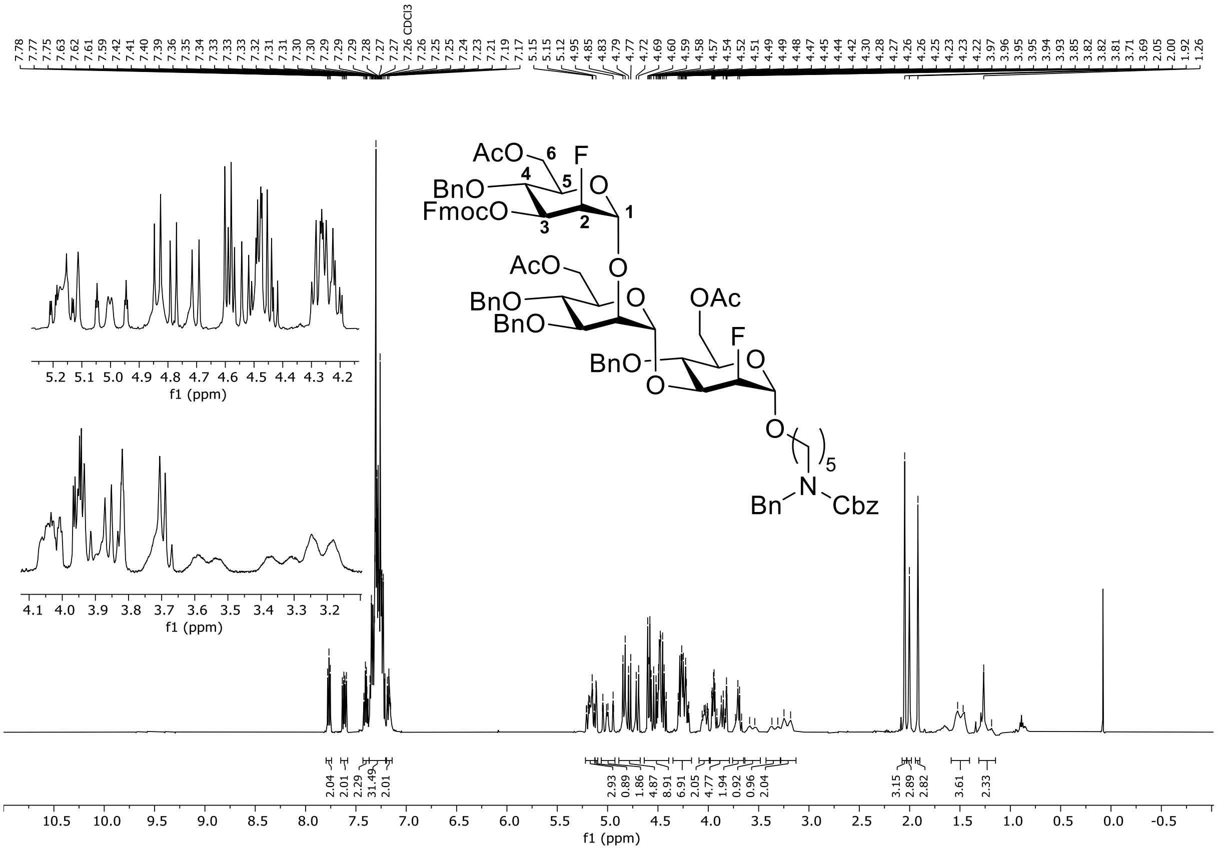

^{13}C NMR, 151 MHz, $CDCl_3$ – Compound S20

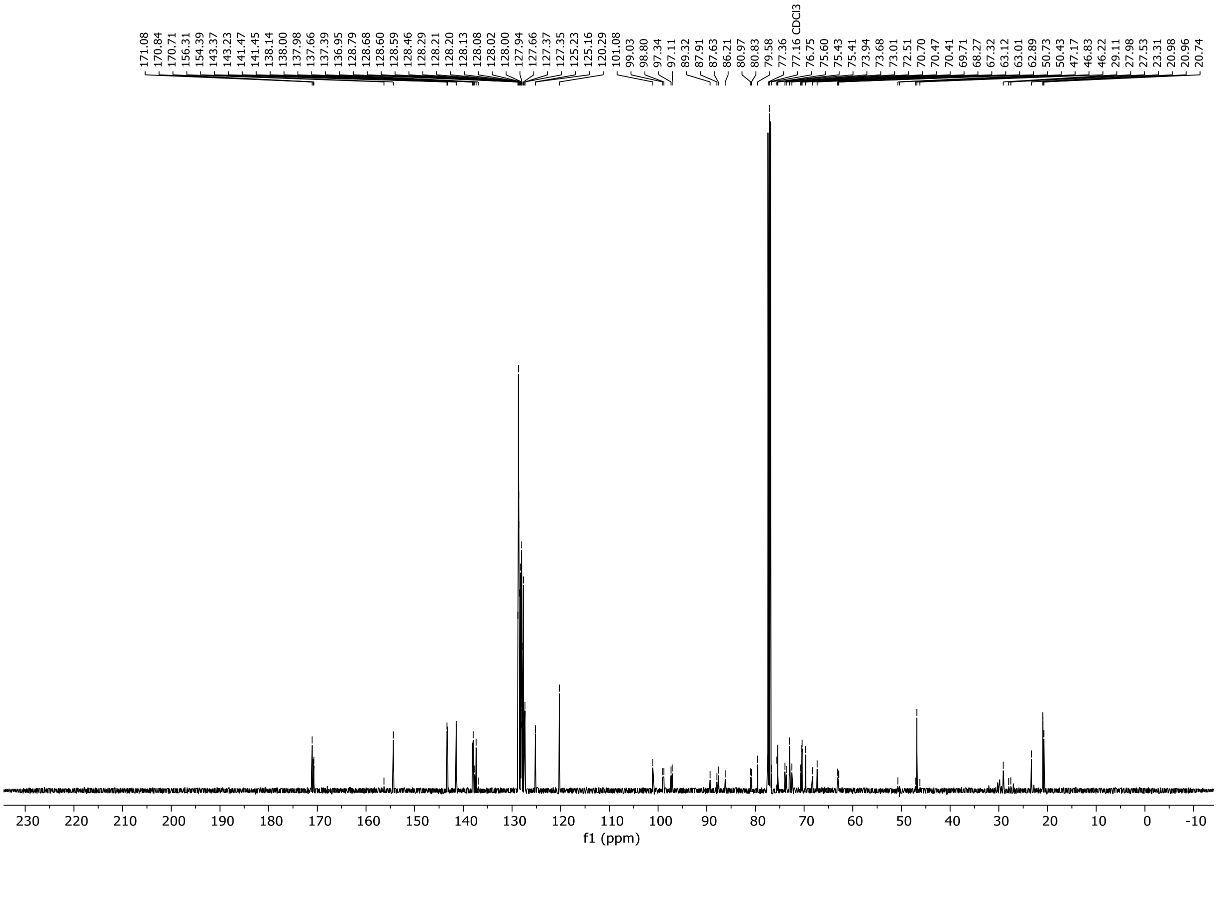

^{19}F NMR, 563 MHz, $CDCl_3$ – Compound S20

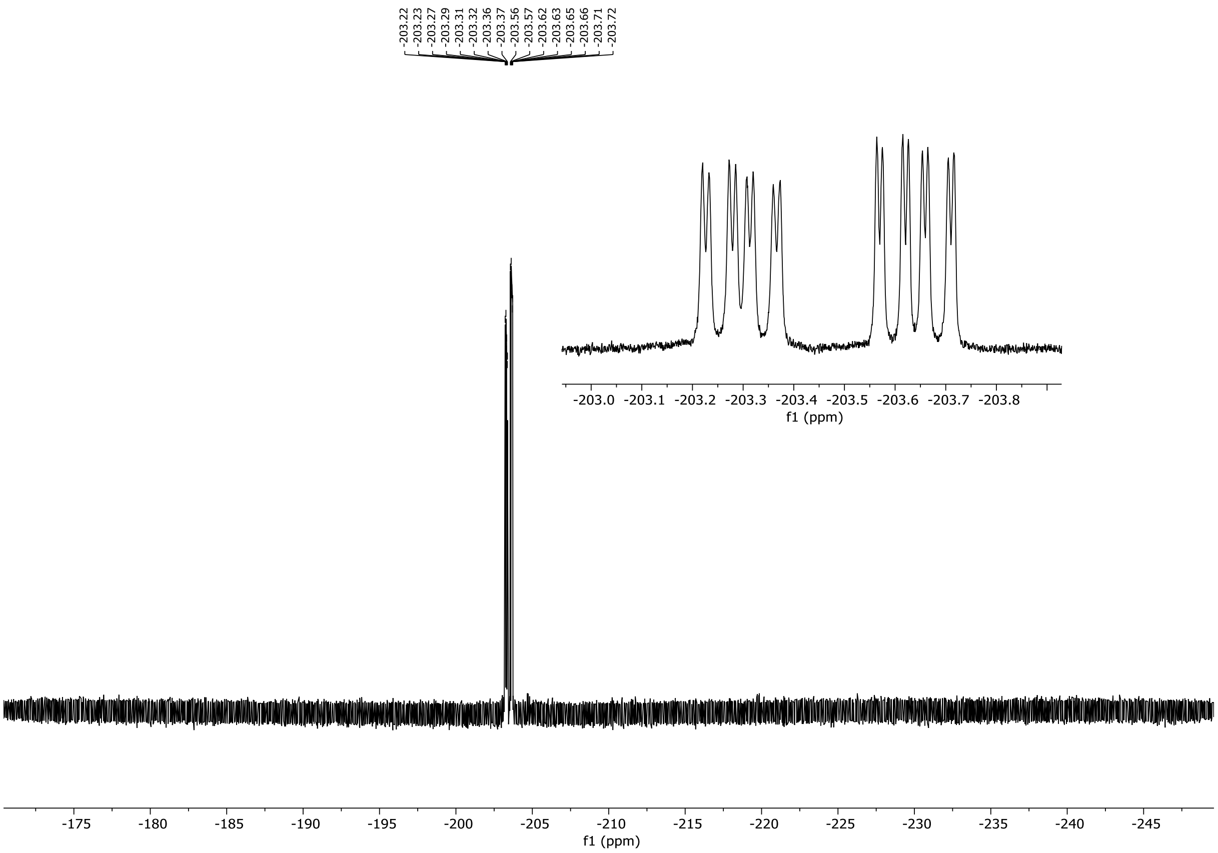

^{1}H-^{13}C gHSQC NMR, $CDCl_3$ – Carbohydrate Region, Compound S20

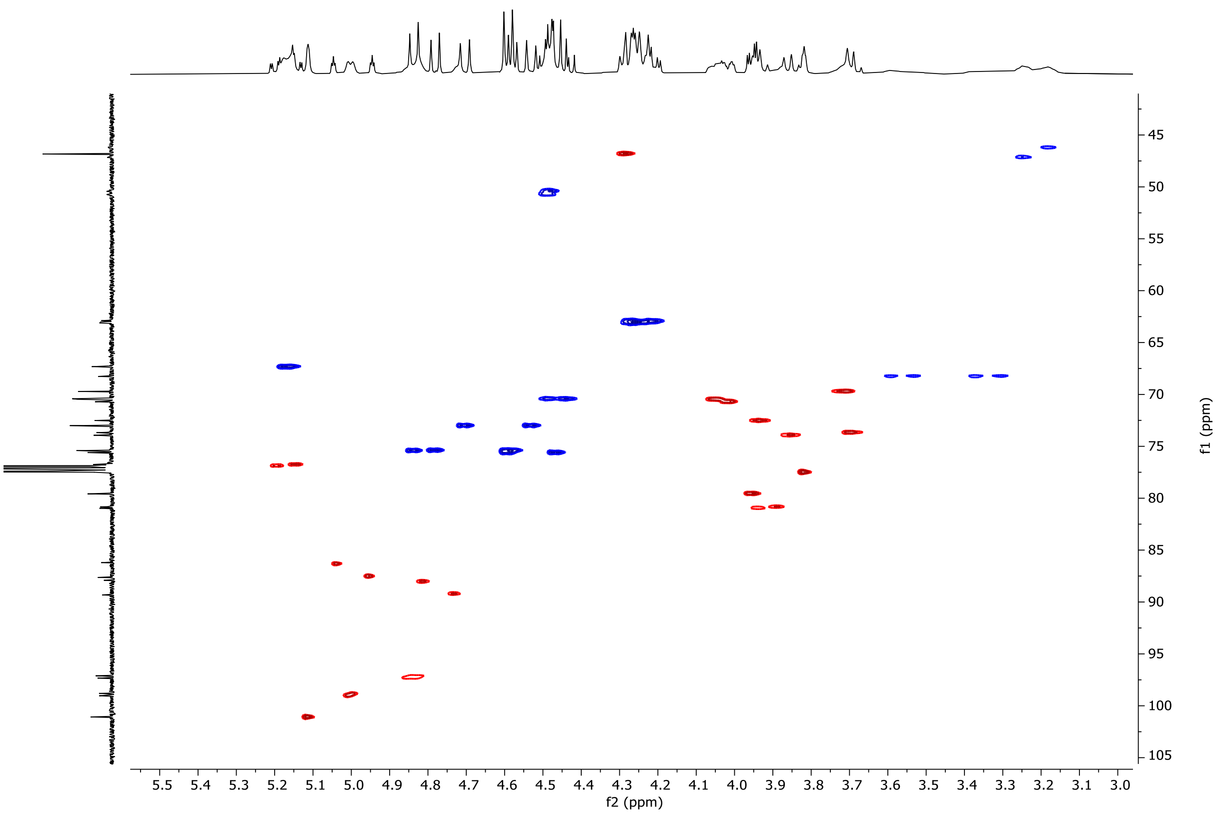

^{1}H NMR, 500 MHz, $CDCl_3$ – Compound S21

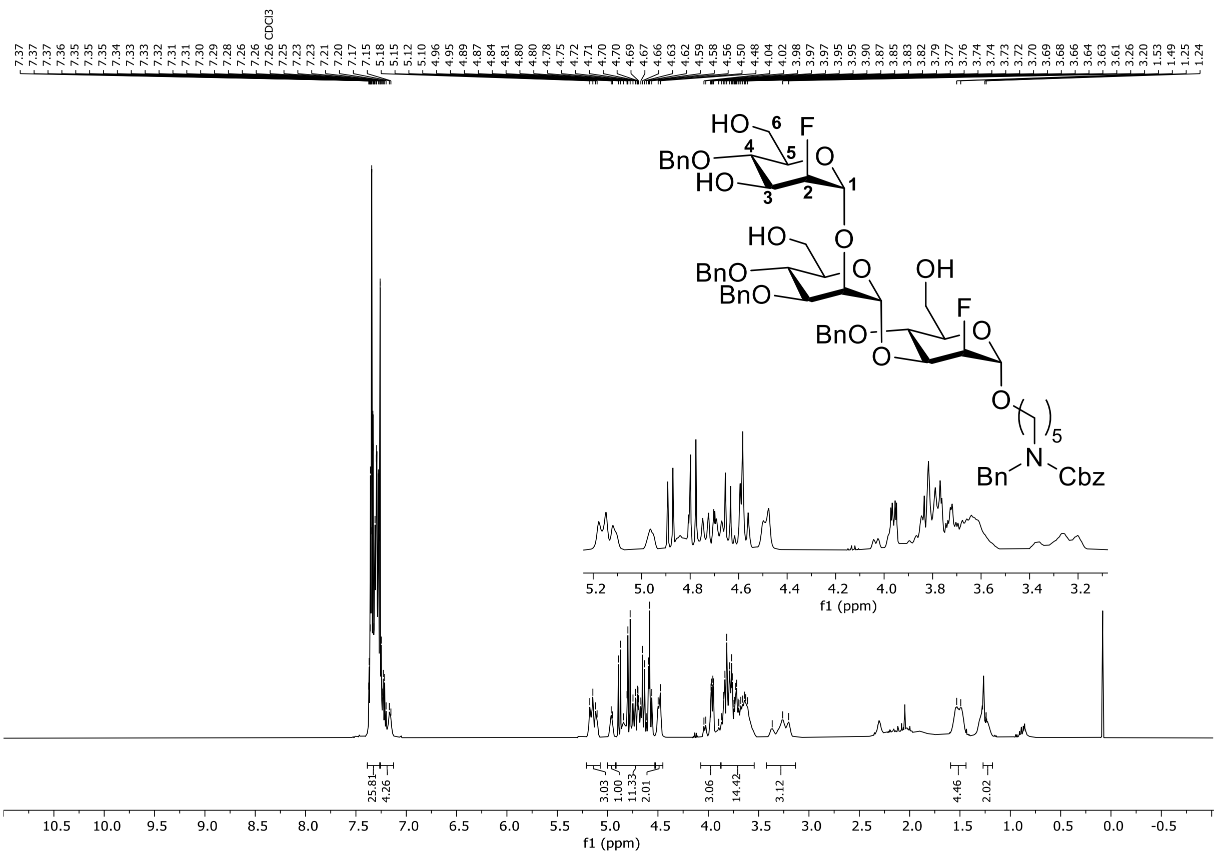

^{13}C NMR, 126 MHz, $CDCl_3$ – Compound S21

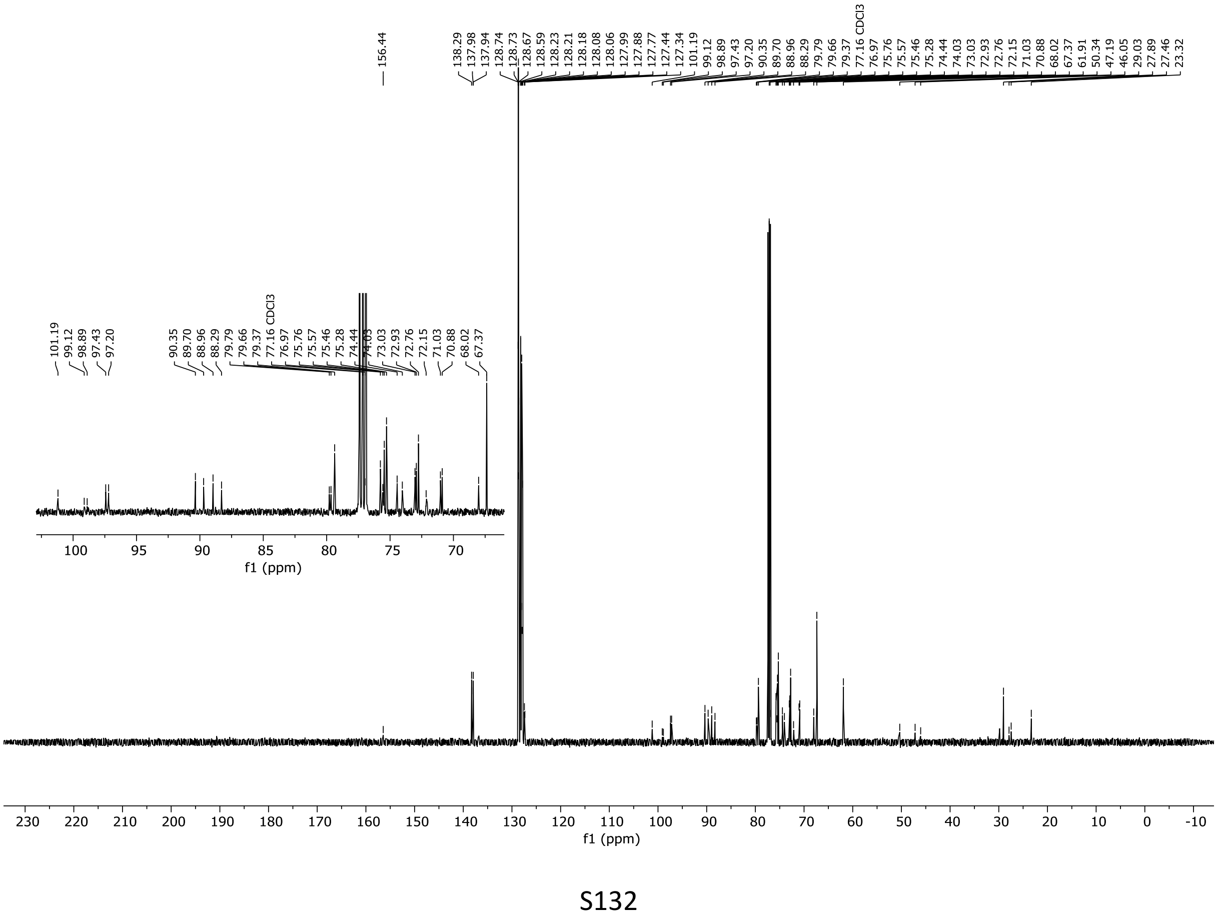

^{19}F NMR, 470 MHz, $CDCl_3$ – Compound S21

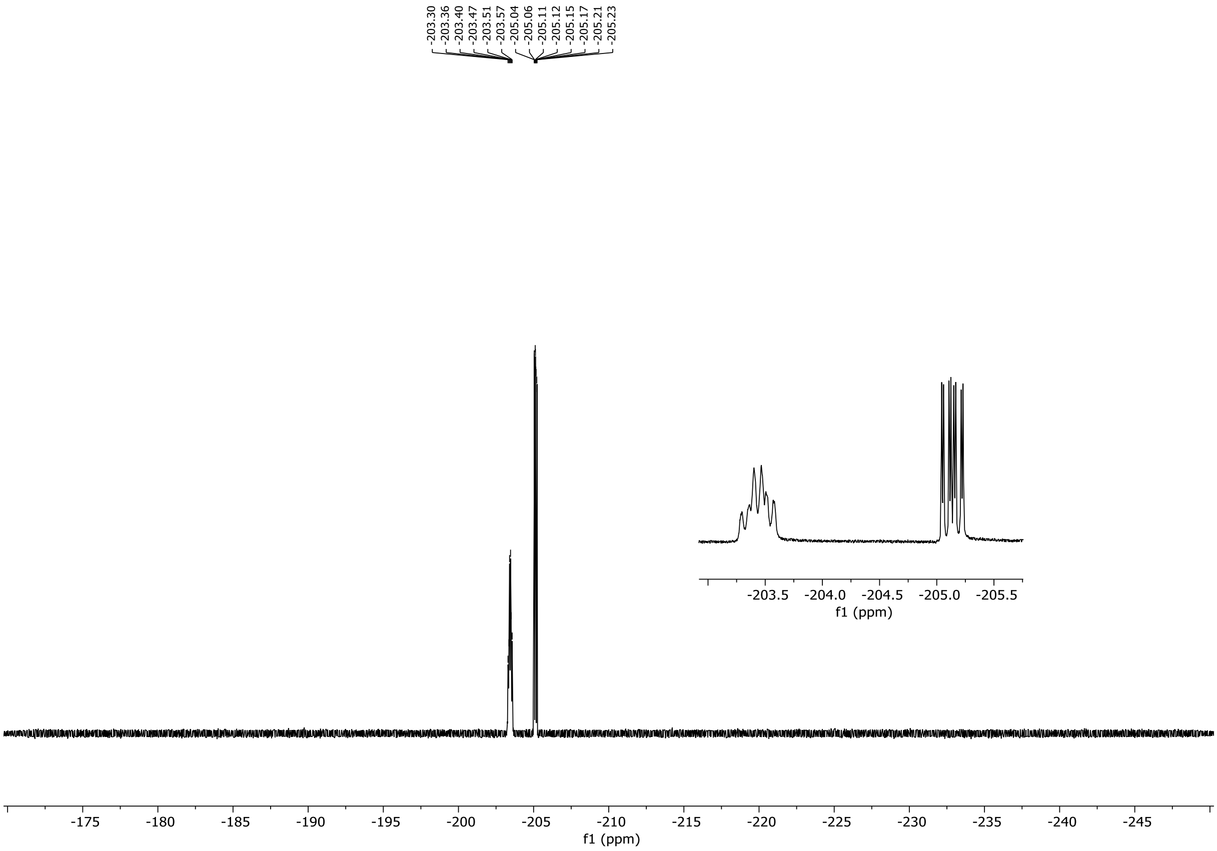

^{1}H-^{13}C gHSQC NMR, $CDCl_3$ – Carbohydrate Region, Compound S21

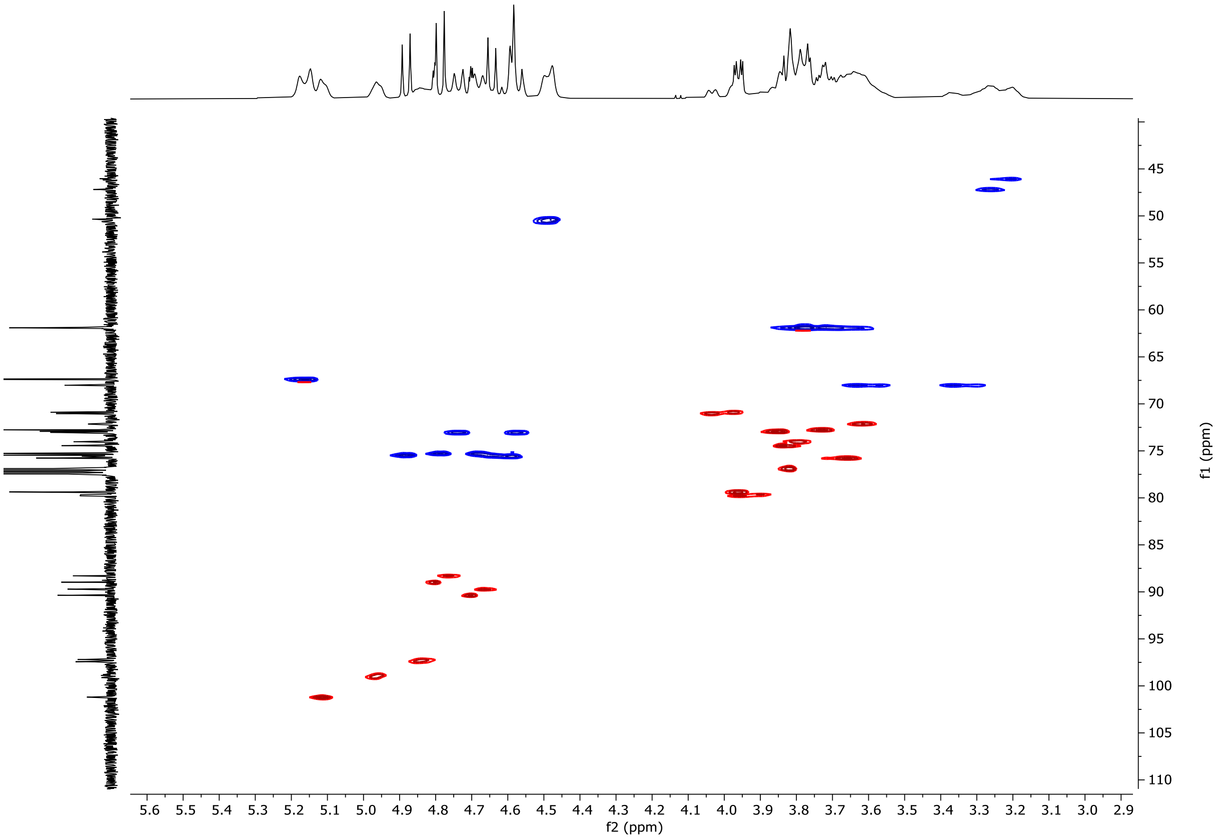

^{1}H NMR, 599 MHz, D_2O – Compound 28

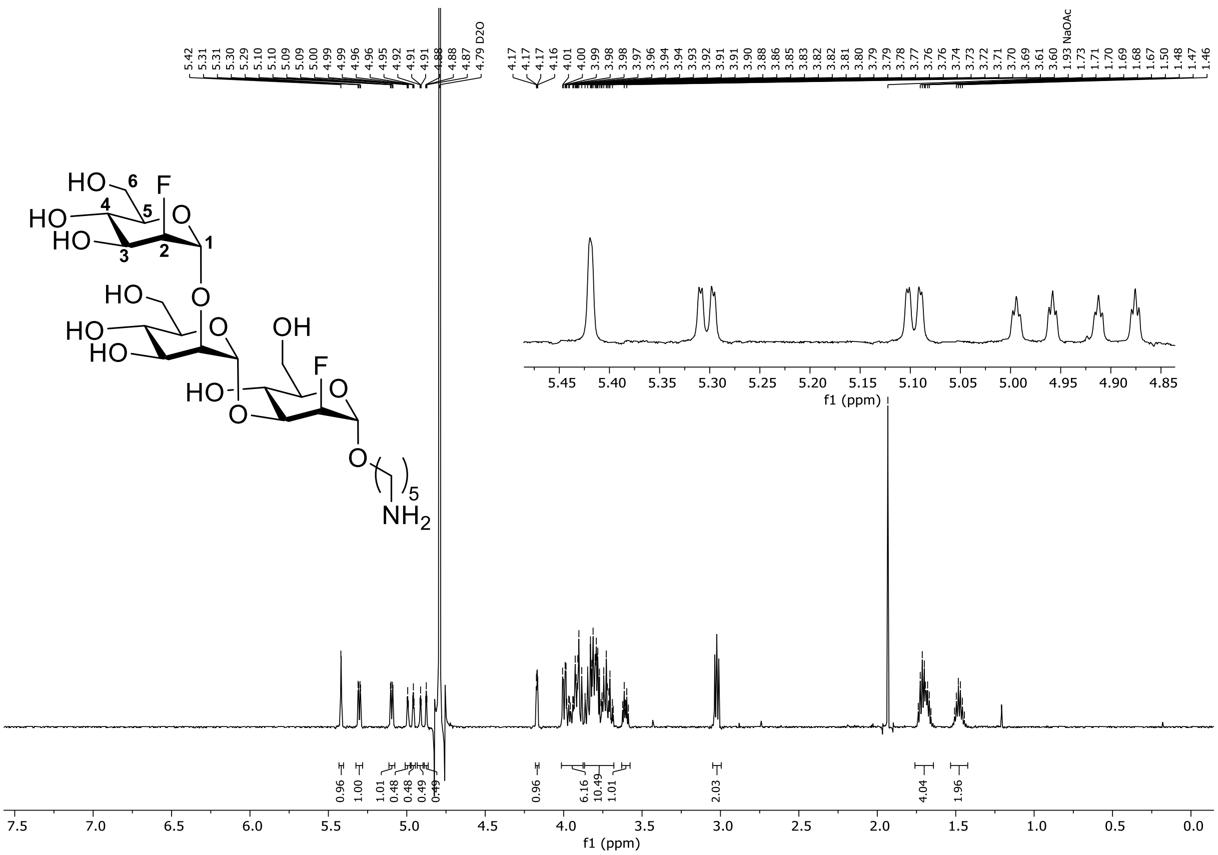

^{13}C NMR, 151 MHz, D_2O – Compound 28

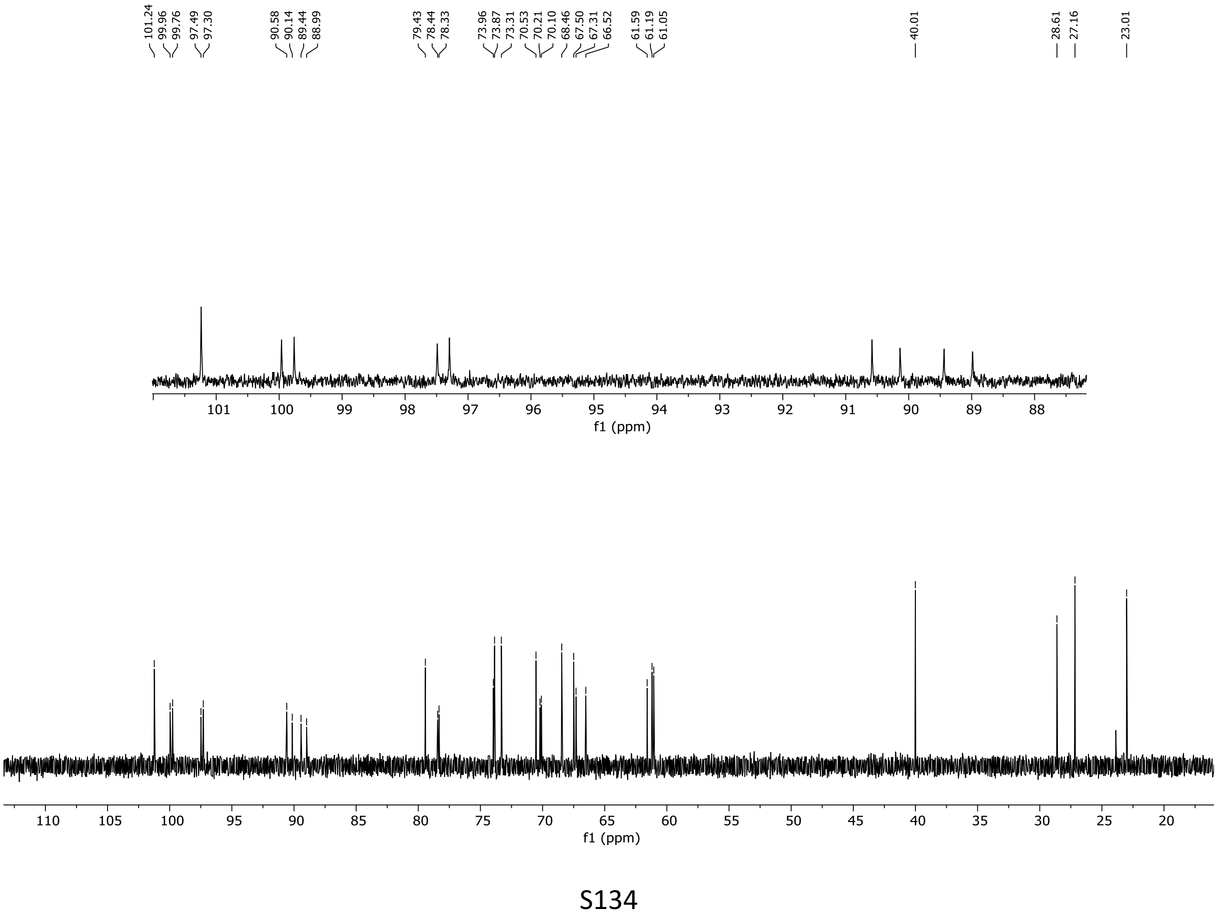

^{19}F NMR, 564 MHz, D_2O – Compound 28

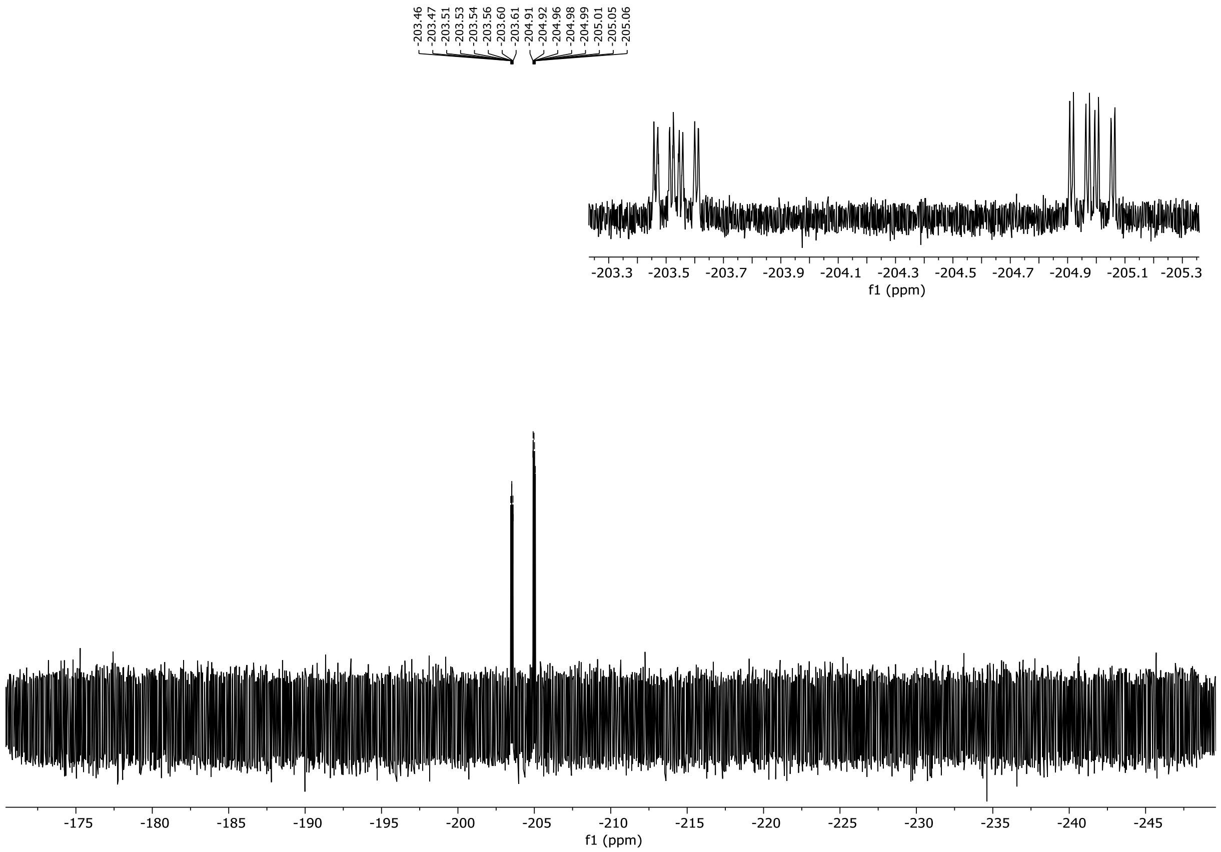

^{1}H-^{13}C gHSQC NMR, D_2O – Compound 28

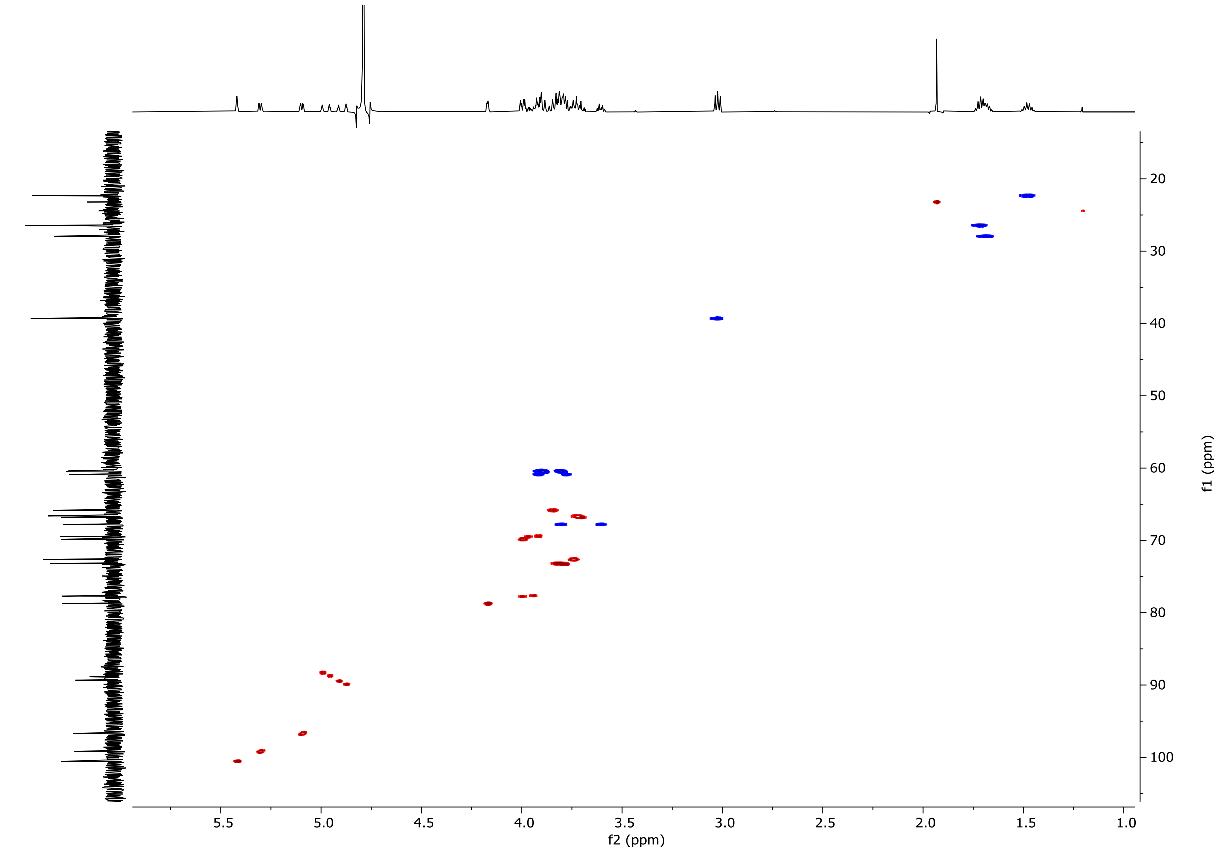

^{1}H NMR, 500 MHz, $CDCl_3$ – Compound S23

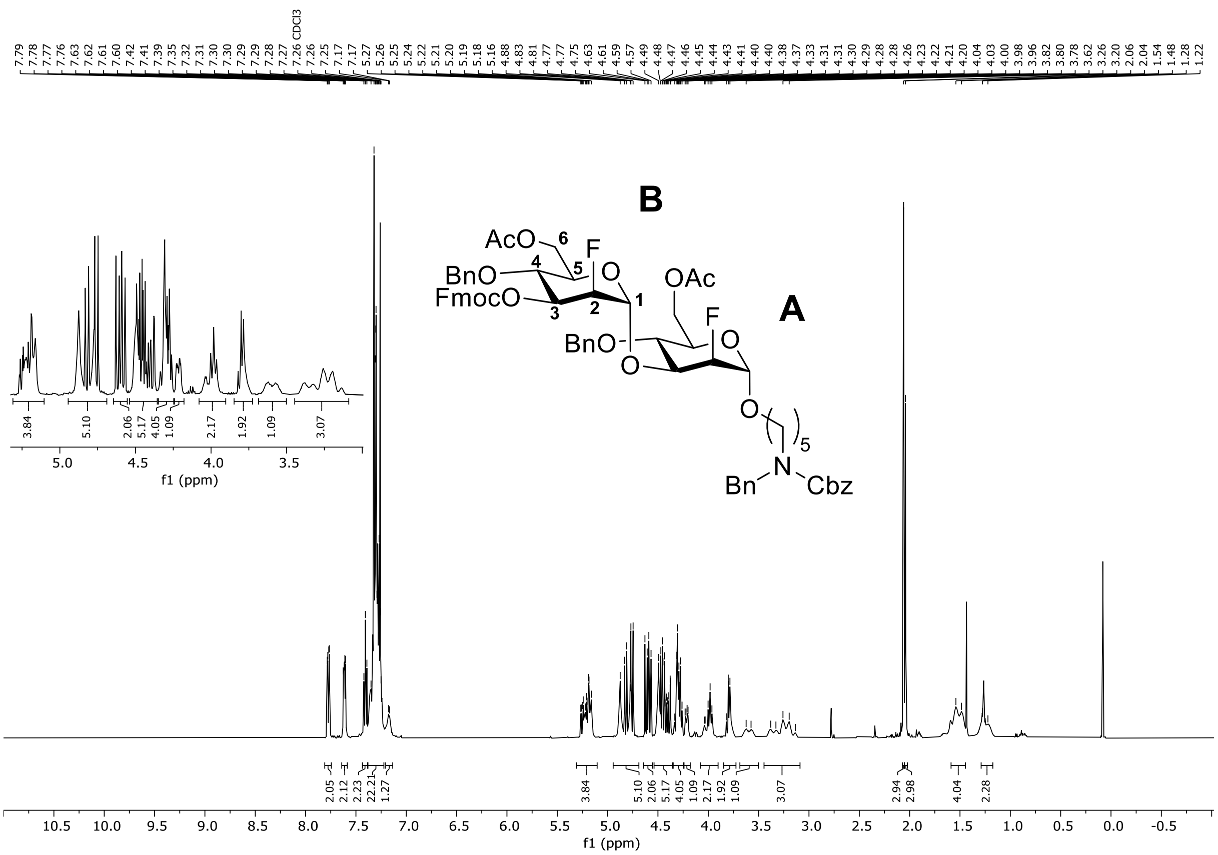

^{13}C NMR, 126 MHz, $CDCl_3$ – Compound S23

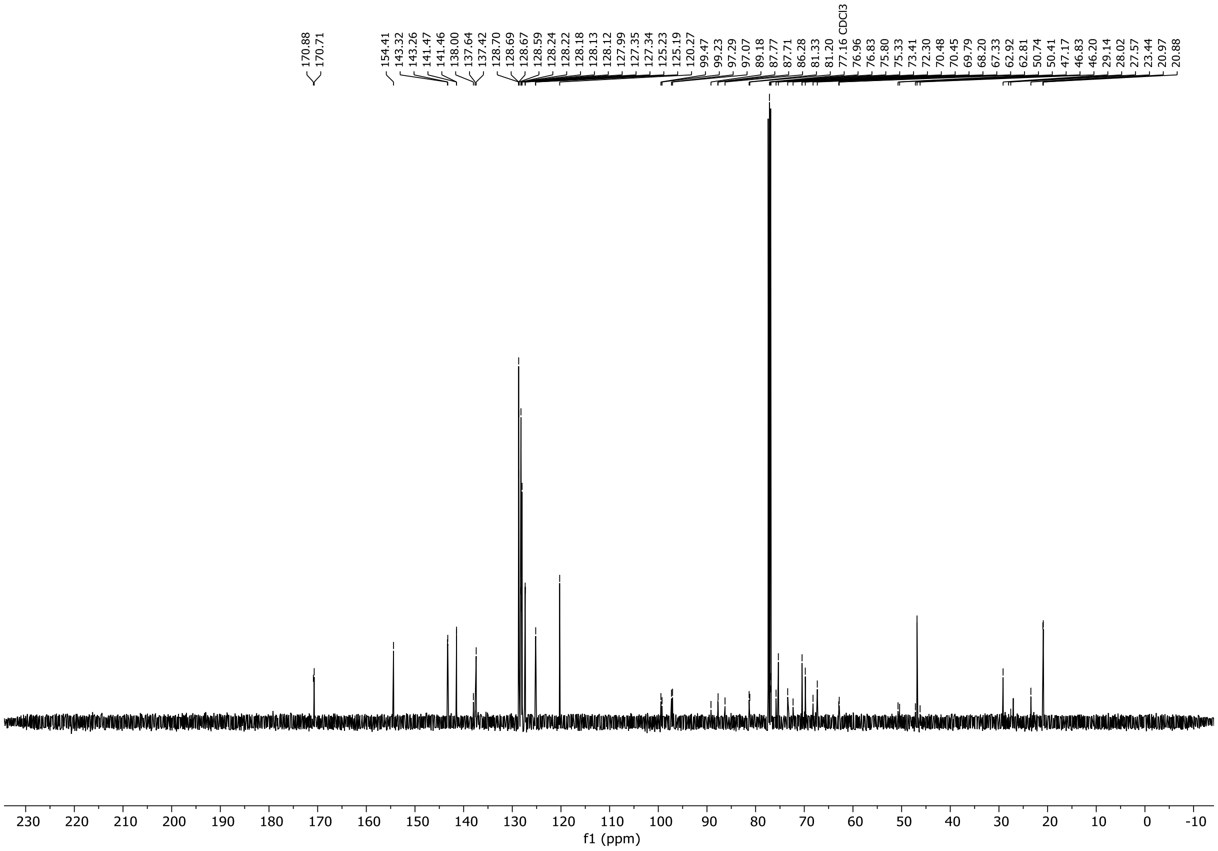

^{19}F NMR, 470 MHz, $CDCl_3$ – Compound S23

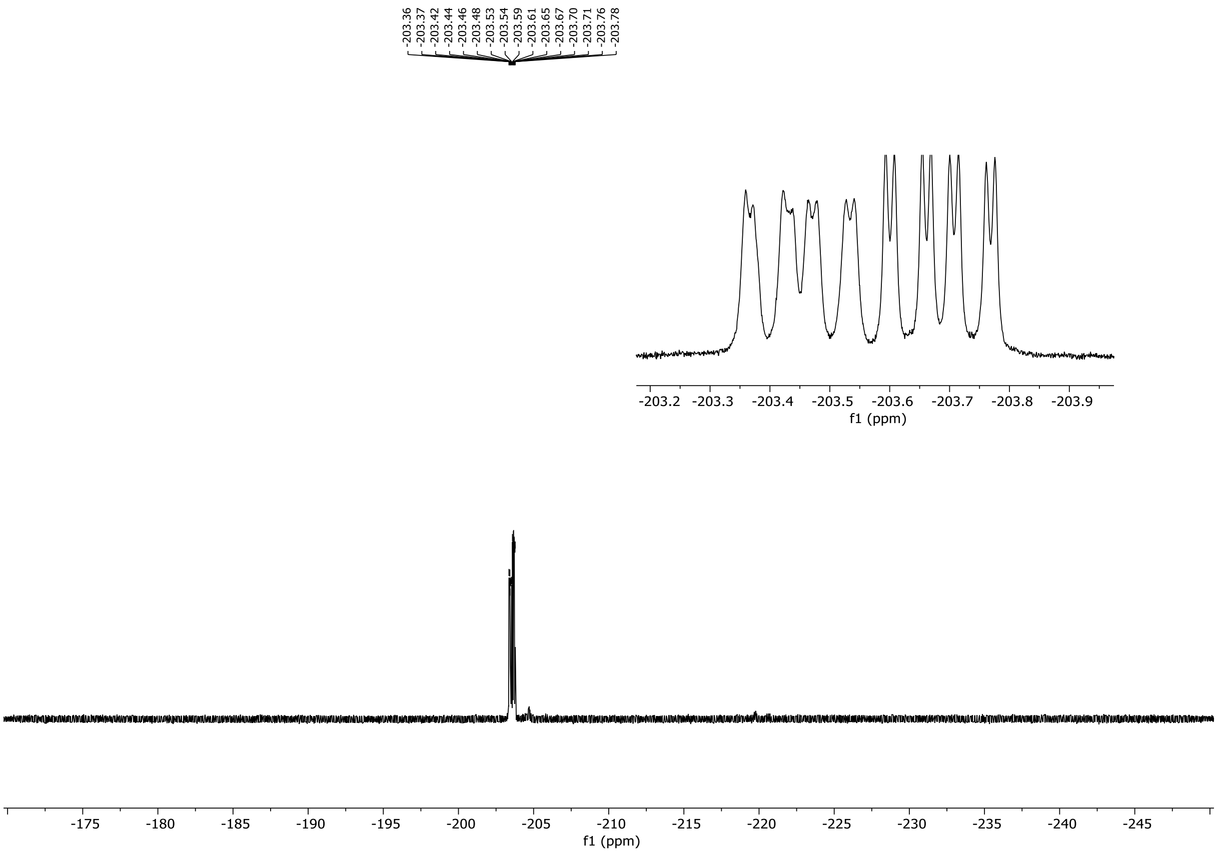

^{1}H-^{13}C gHSQC NMR, $CDCl_3$ – Carbohydrate Region, Compound S23

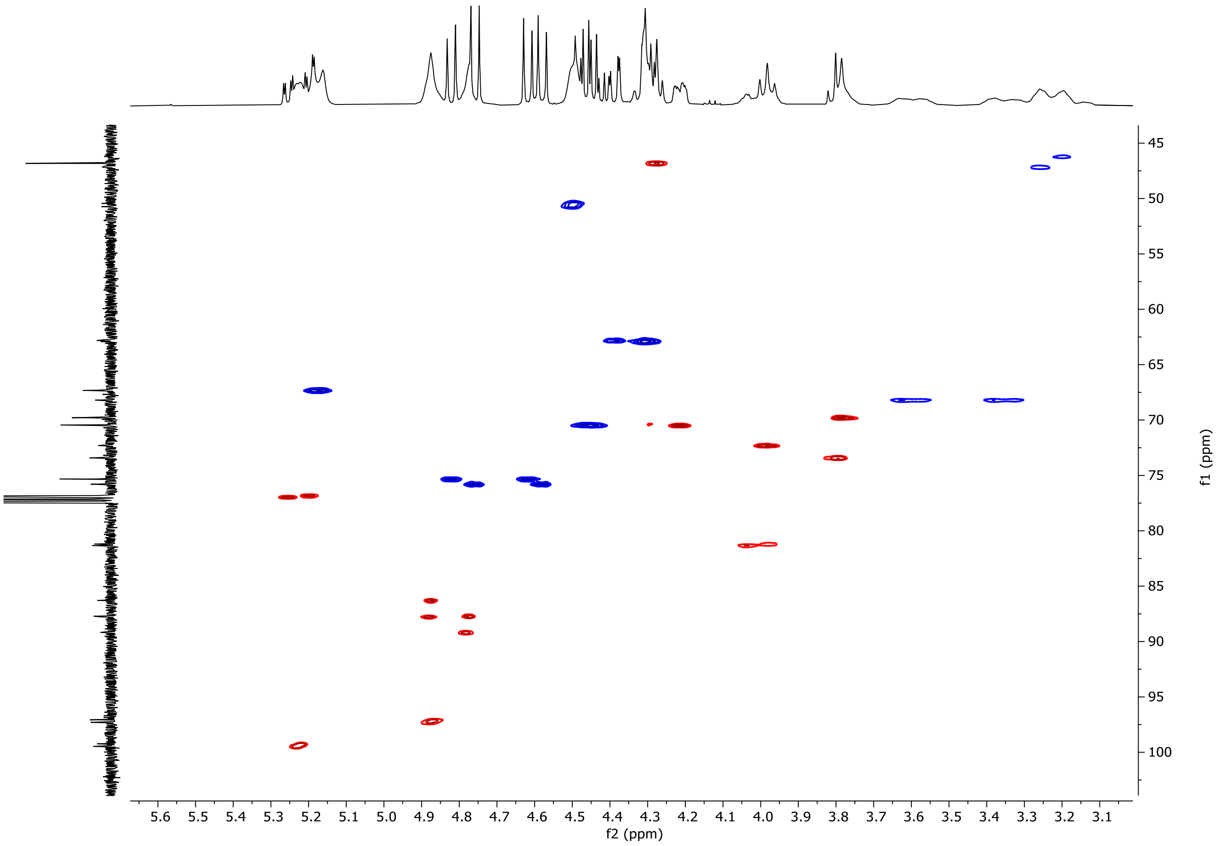

^{1}H NMR, 500 MHz, $CDCl_3$ – Compound S24

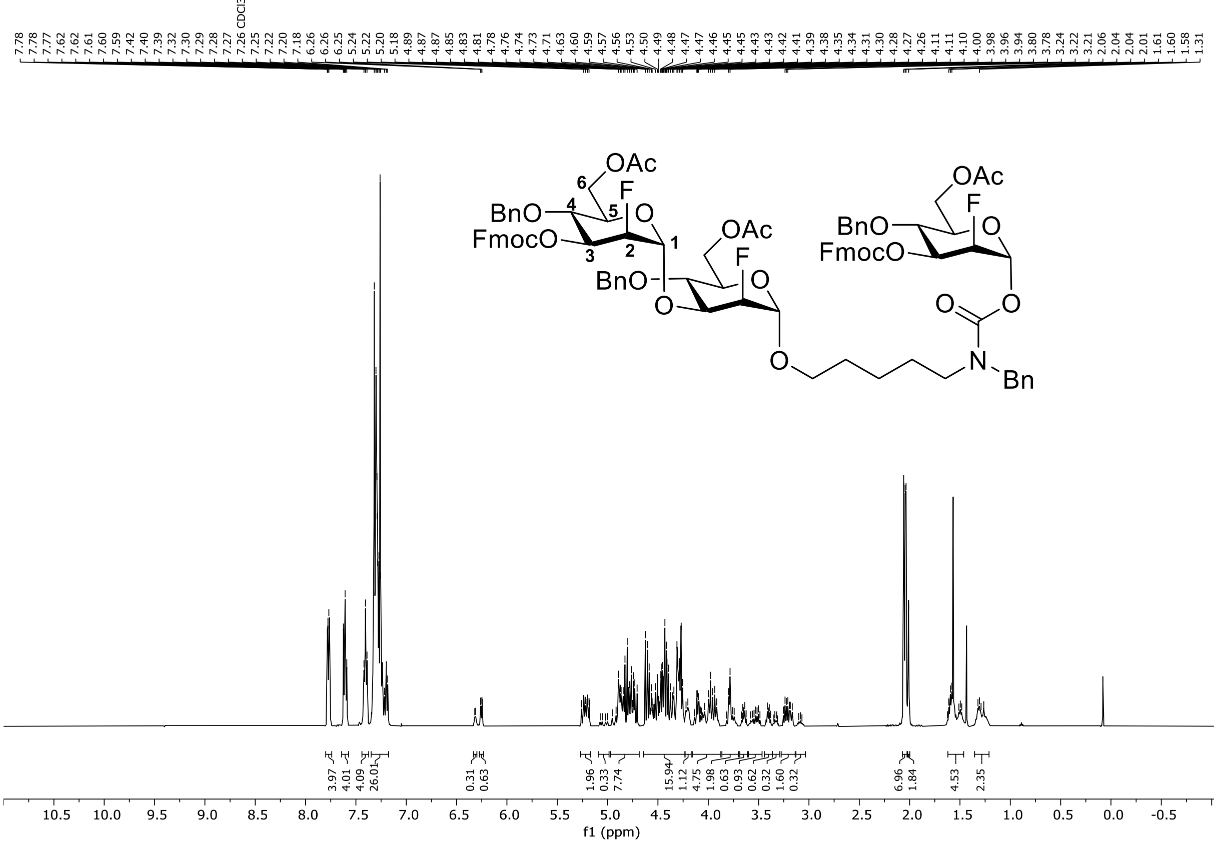

^{13}C NMR, 126 MHz, $CDCl_3$ – Compound S24

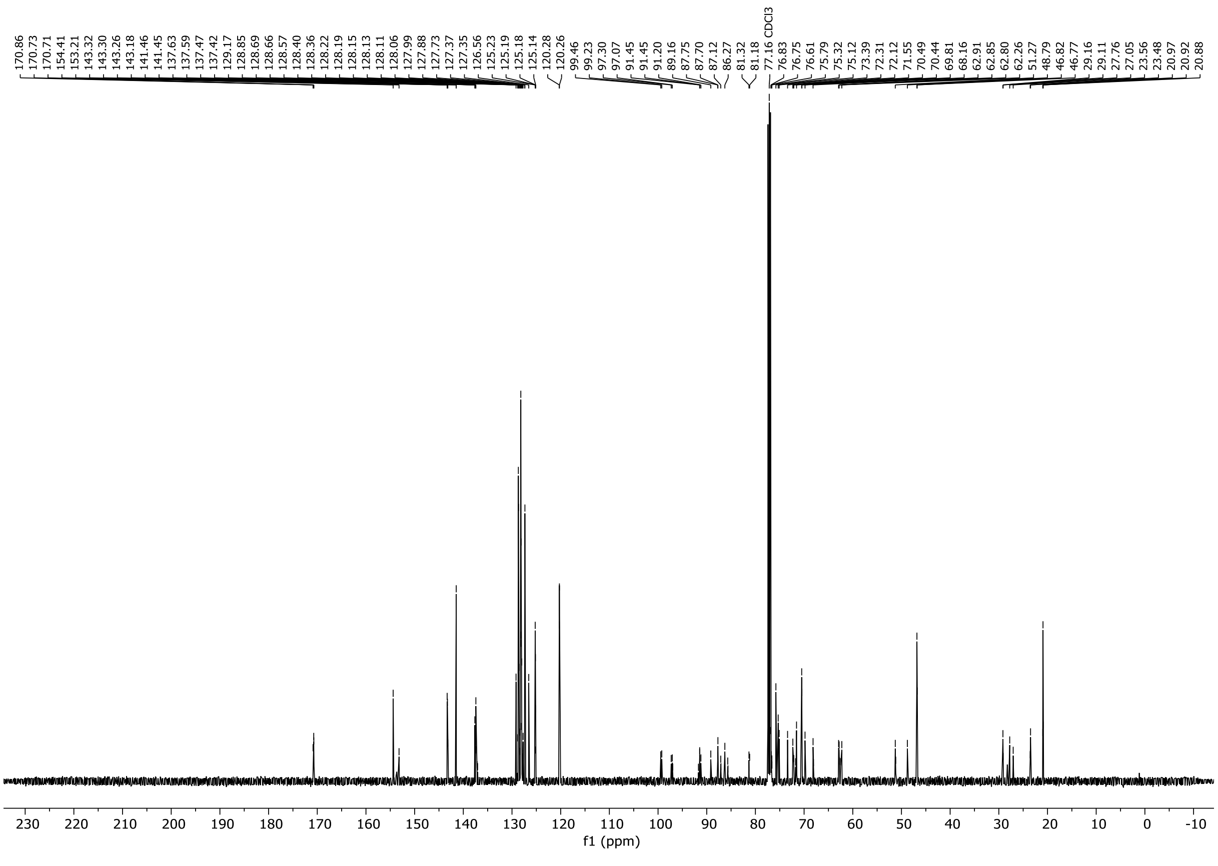

^{19}F NMR, 470 MHz, $CDCl_3$ – Compound S24

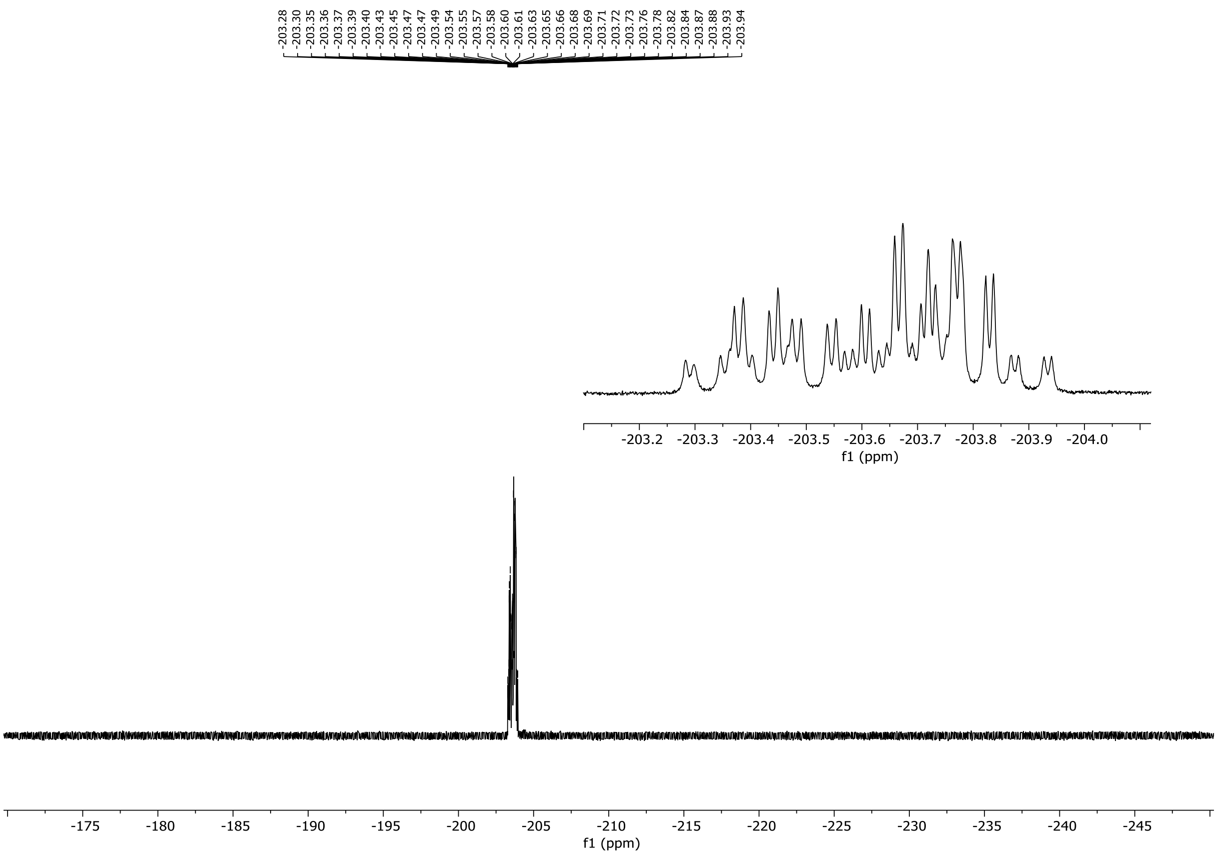

^{1}H-^{13}C gHSQC NMR, $CDCl_3$ – Carbohydrate Region, Compound S24

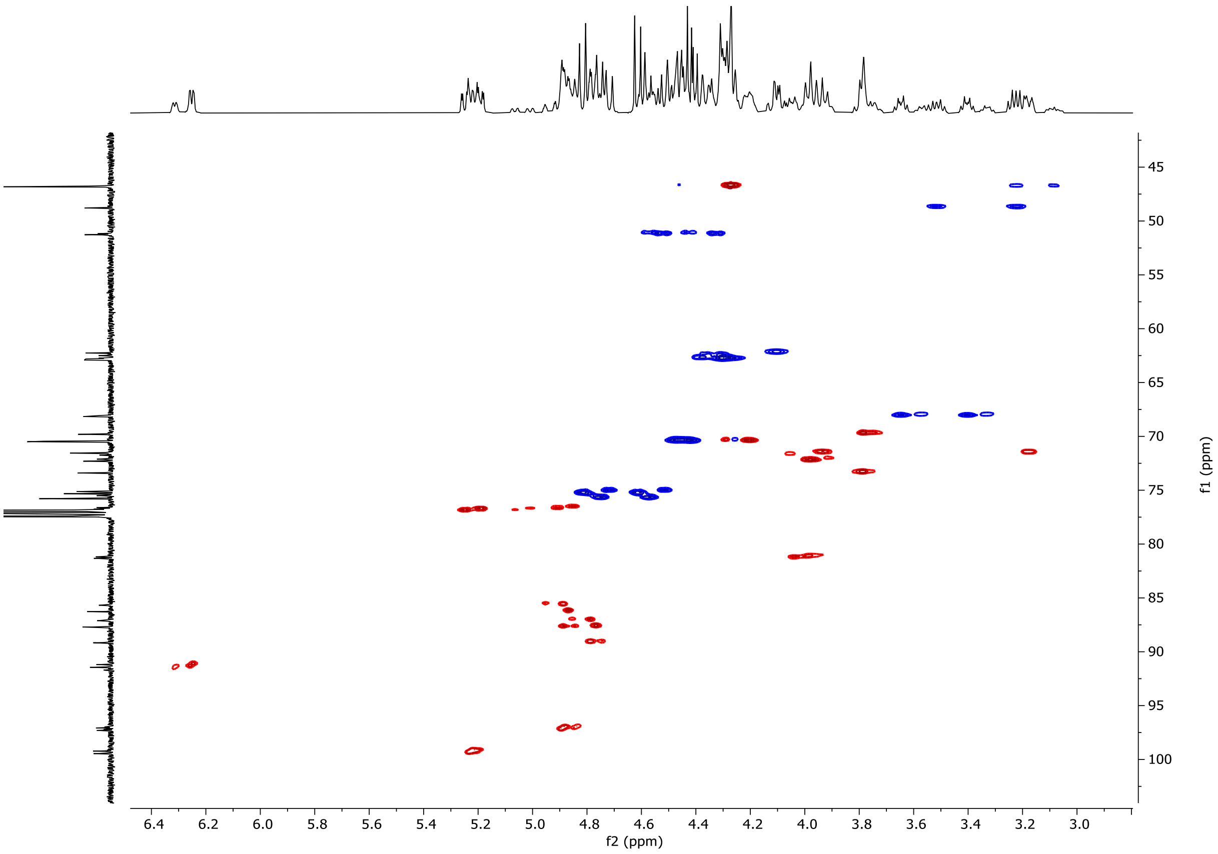

$^{19}F\{^1H\}$ -$^{19}F\{^1H\}$ NOESY/EXSY, 470 MHz, $CDCl_3$, 303 K – Compound S24

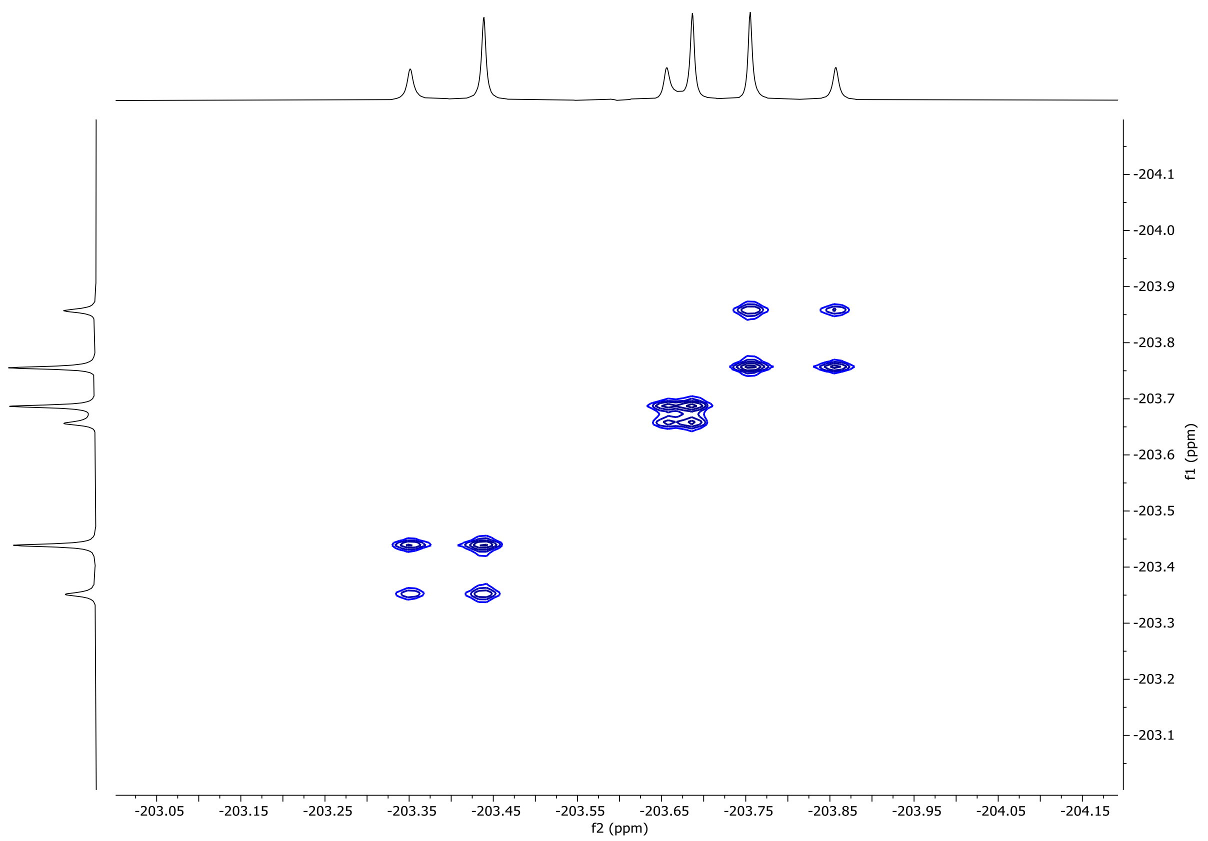

^{19}F-^{13}C gc2hsqcse NMR, $CDCl_3$ – Compound S24

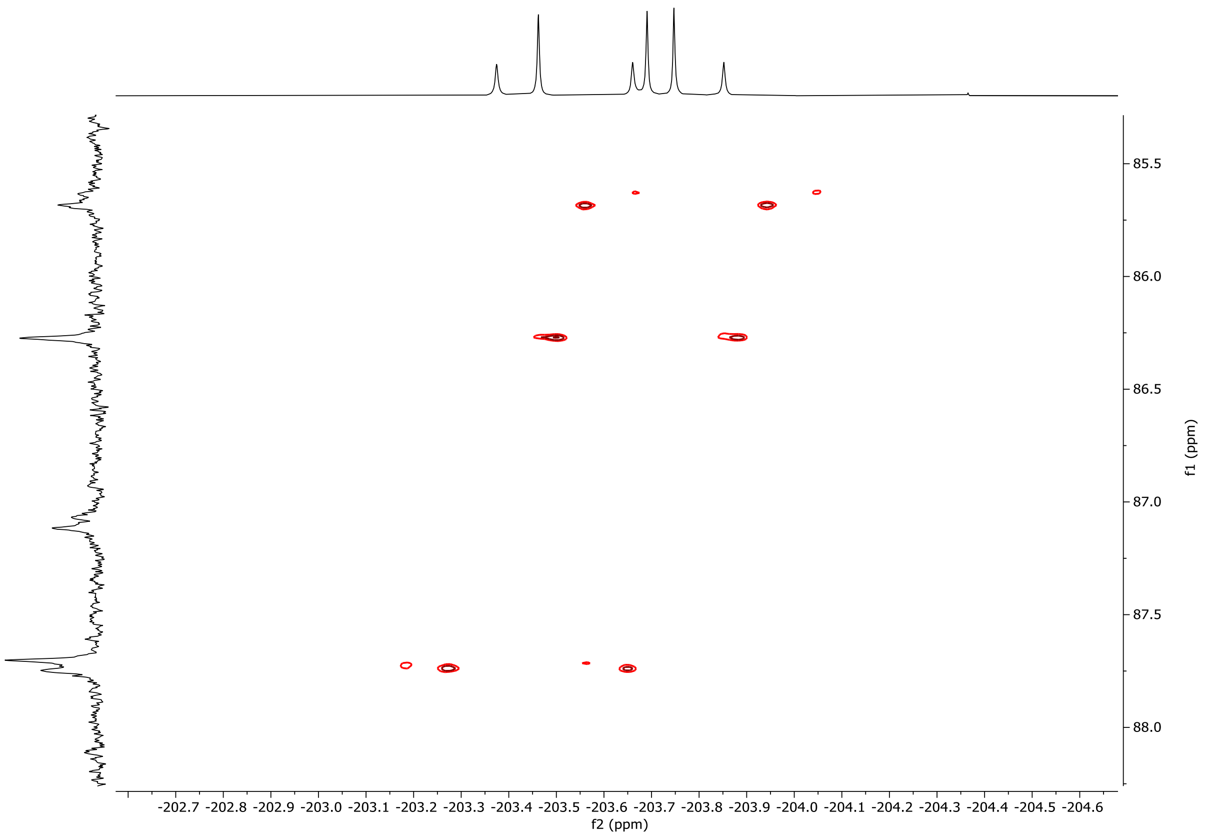

References

[1] C. J. Crawford, Y. Qiao, Y. Liu, D. Huang, W. Yan, P. H. Seeberger, S. Oscarson, S. Chen, *Org. Process Res. Dev.* **2021**, *25*, 1573–1578.

[2] H. E. Gottlieb, V. Kotlyar, A. Nudelman, *J. Org. Chem.* **1997**, *62*, 7512–7515.

[3] C. S. Teschers, R. Gilmour, *Org. Process Res. Dev.* **2020**, *24*, 2234–2239.

[4] C. S. Teschers, R. Gilmour, *Angew. Chem. Int. Ed.* **2023**, *62*, e202213304.

[5] M. Gude, J. Ryf, P. D. White, *Lett. Pept. Sci.* **2002**, *9*, 203–206.

[6] D. L. Jensen, H. H. Trinderup, F. Skovbo, H. H. Jensen, *Org. Biomol. Chem.* **2022**, *20*, 4915–4925.

[7] P. S. Patil, C.-C. Lee, Y.-W. Huang, M. M. L. Zulueta, S.-C. Hung, *Org. Biomol. Chem.* **2013**, *11*, 2605.

[8] L. Legentil, Y. Cabezas, O. Tasseau, C. Tellier, F. Daligault, V. Ferrières, *J. Org. Chem.* **2017**, *82*, 7114–7122.

[9] A. J. Thompson, R. J. Williams, Z. Hakki, D. S. Alonzi, T. Wennekes, T. M. Gloster, K. Songsrirote, J. E. Thomas-Oates, T. M. Wrodnigg, J. Spreitz, A. E. Stütz, T. D. Butters, S. J. Williams, G. J. Davies, *Proc. Nat. Acad. Sci.* **2012**, *109*, 781–786.

[10] C. Bucher, R. Gilmour, *Angew. Chem. Int. Ed.* **2010**, *49*, 8724–8728.

[11] O. T. Tuck, E. T. Sletten, J. Danglad-Flores, P. H. Seeberger, *Angew. Chem. Int. Ed.* **2022**, *61*, DOI 10.1002/anie.202115433.

[12] M. Lin, Y. Kuo, J. Danglad-Flores, E. T. Sletten, P. H. Seeberger, *Chem. Eur. J.* **2024**, *30*, e202400479.

[13] J.-G. Delcros, S. Tomasi, S. Carrington, B. Martin, J. Renault, I. S. Blagbrough, P. Uriac, *J. Med. Chem.* **2002**, *45*, 5098–5111.
